# One-Pot Two-Step Synthesis of 2-Aryl benzimidazole *N*-oxides Using Microwave Heating as a Tool

**DOI:** 10.3390/molecules24203639

**Published:** 2019-10-09

**Authors:** Fabrizio Politano, Ana K. Gran-Magano, Nicholas E. Leadbeater

**Affiliations:** Department of Chemistry, University of Connecticut, 55 North Eagleville Road, Storrs, CT 06269-3060, USA; fabrizio.politano@uconn.edu (F.P.); ana.magano@uconn.edu (A.K.G.-M.)

**Keywords:** microwave heating, benzimidazole, benzimidazole *N*-oxide, green chemistry

## Abstract

A methodology is reported for the preparation of 2-aryl-benzimidazole-3-oxide derivatives. By means of a one-pot two-step protocol, a library of 42 new compounds has been prepared. Reactions were performed in a total time of 40 min using microwave heating as a tool. A streamlined work-up process was also developed, allowing for facile isolation of the products. The methodology offers a more sustainable approach than previous routes, with only water and ethanol being used as solvents, and the products being isolated by means of a simple filtration without the need for any further purification.

## 1. Introduction

**Figure 1:**
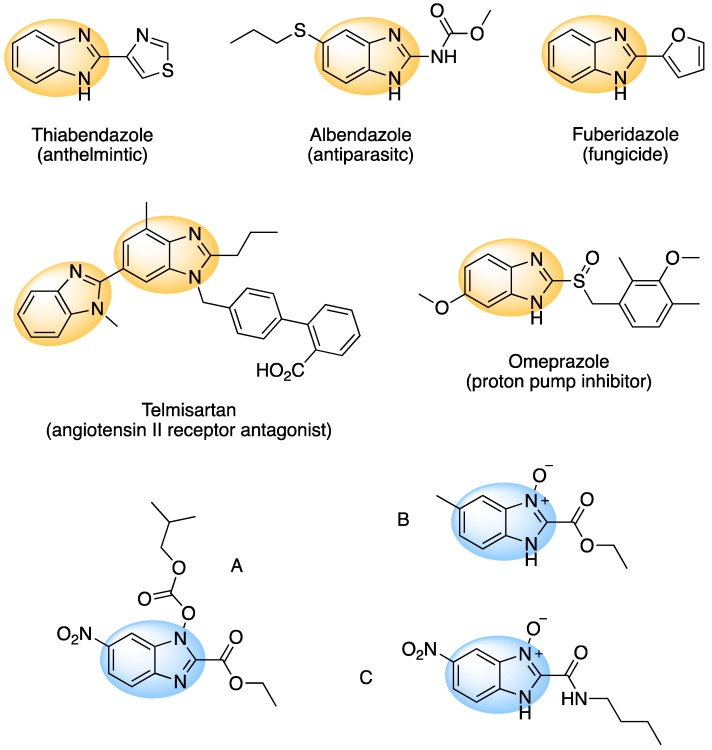
A range of pharmaceuticals containing the benzimidazole motif (highlighted in yellow). A, B, and C are compounds bearing the benzimidazole *N*-oxide motif (highlighted in blue) that show anti-protozoa activity.

Nitrogen-containing heterocyclic compounds play a vital role in drug development because they can increase binding efficiency and solubility as well as facilitate the formation of salts. These properties improve oral absorption and bioavailability of the drug [1,2]. The benzimidazole scaffold falls into this category. This motif is present in several commercial available pharmaceuticals that are used for the treatment of different diseases and disorders, such as ulcers, high blood pressure, tumors, and allergies, as well as fungal, bacterial, and viral infections (Figure 1) [3,4,5,6]. Some 1-alkoxy-2-alkylbenzimidazoles obtained from 2-nitroanilines have shown anti-human immunodeficiency virus (HIV) activity [7]. Other benzimidazoles have shown antagonist activity of the chemokine receptor responsible for autoimmune diseases such as multiple sclerosis and rheumatoid arthritis [8]. More recently, the lithium salts of benzimidazoles have found use in the development of lithium-ion batteries [9,10,11]. The wide range of applications of benzimidazole derivatives has reignited interest in the development of improved synthetic routes.

Benzimidazole *N*-oxide derivatives have a unique functionality, due to the N-O moiety that can act effectively as a push-pull electron donor/acceptor group; the N^+^-O^−^ group being strongly polarizable in both directions [12]. In addition, the N-O moiety can improve the solubility of the compounds in polar solvents [13,14]. These features furnish benzimidazole *N*-oxides with unique and interesting chemical [15] and biological [16] properties. For example, some imidazole and benzimidazole *N*-oxides show anti-protozoa activity when tested against *Trypanosoma cruzi* and *Trichomonas vaginalis* [17]. Despite the potential applicability of these compounds, there are relatively few synthetic routes to reach them.

The synthesis of benzimidazole *N*-oxides is usually carried on through base-mediated cyclization reactions of o-nitroanilines [18] instead of direct oxidation of benzimidazoles [19]. One approach involves a two-step process [20,21,22]. In the first step, 2-nitroanilines are prepared through an S_N_Ar reaction between a suitably-functionalized haloarene and an amine. This is followed by a cyclization. Both steps employ solvents such as 1,4-dioxane and dimethylformamide (DMF), which are undesirable from a sustainability viewpoint [23,24,25,26,27,28]. In our research, we have attempted to address this drawback by developing a continuous-flow process using ethanol, 2-propanol, and water as solvents [29]. This procedure works well with substrates bearing short-chain alkyl substituents, but shows some drawbacks when more non-polar substituents, due to precipitation of the 2-nitroaniline intermediates with concommitant clogging of the flow reactor. In order to prepare a broader range of benzimidazole-*N*-oxides for screening in a biological assay, we have turned our attention to developing an improved protocol for their preparation. Microwave heating has proven an excellent tool in this endeavor.

Since the first reports in 1986 [30,31], microwave heating has found application in a wide range of organic transformations. Indeed, it can be said that if a reaction requires heating, microwave irradiation proves to be a valuable tool. With the advent of microwave units designed with the synthetic chemist in mind, the number of reports has increased rapidly [32]. Reactions can be performed safely in sealed vessels and a range of parameters such as temperature, pressure, and stirring speed can be monitored in real-time. This leads to robust, reproducible methodologies [33,34]. Here, we report the rapid and efficient synthesis of 42 new benzimidazole *N*-oxide derivatives by a one-pot two-step methodology facilitated by microwave heating.

## 2. Results and Discussion

### 2.1. Synthesis

**Scheme 1:**
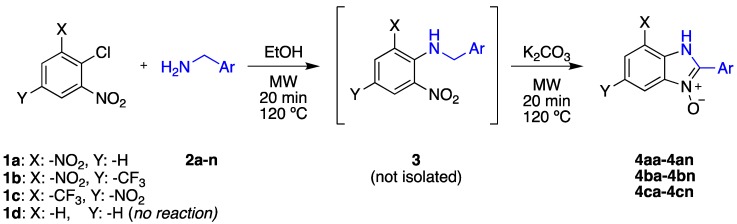
Synthesis of 2-aryl-benzimidazole *N*-oxide derivatives.

Our starting point for developing our methodology was to build on the previously reported two-step route [29,35]. Our objective was to use ethanol as the solvent for the S_N_Ar reaction between *o*-nitrochlorobenzene derivatives (**1a-c**) and a range of different aryl amines (**2a-n**) (Scheme 1).

Optimal conditions were found to involve heating the reaction mixture for 20 min at 120 °C. This led to the formation of the desired aniline product, **3**, in good to excellent yield. Rather than isolate this intermediate, our objective was to convert the 2-nitroanilines directly to the corresponding benzimidazole *N*-oxides. We achieved this by adding a quantity of 0.5 M aqueous potassium carbonate K_2_CO_3_ and subjecting the reaction mixture to a further heating cycle of 20 min at 120 °C. After this time, the crude product mixture was assayed using ^1^H-NMR spectroscopy. In all cases, conversion to the desired benzimidazole *N*-oxide, **4**, was greater than 90%, the remaining material being identified as unreacted intermediate **3**.

### 2.2. Product Isolation

With a synthetic methodology in hand, our attention turned to isolation of the benzimidazole *N*-oxides in high purity. This proved to be the most challenging aspect of the study. We initially followed a literature protocol [29]. This consisted of an initial liquid-liquid extraction with ethyl acetate in order to remove any unreacted aniline, **3**, since this is preferentially soluble in the organic layer. Acidification of the aqueous phase in order to protonate the benzimidazole derivative, followed by extraction with ethyl acetate, gave the desired 2-aryl-benzimidazole N-oxide derivative, **4**, in pure form. We followed this work-up protocol for four representative examples, but in each case, the isolated yield obtained was unacceptably low (Table 1). Therefore, we decided to develop a more effective product isolation workflow.

**Scheme 2:**
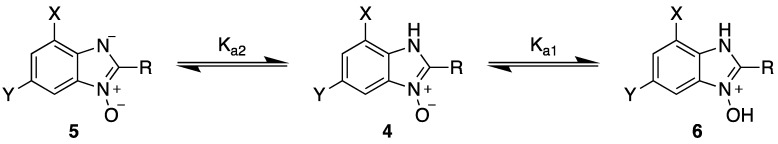
Acid-base equilibria present in benzimidazole *N*-oxide derivatives.

In the acidification step of the literature work-up protocol, we observed the formation of a precipitate. This was not something that had been reported when using simple substrates. We believed that in our family of compounds the hydrophobic groups (aryl moieties) attached to the 2-position of the benzimidazole ring played a key role in the precipitation process. This made us probe the acid-base characteristics of benzimidazole *N*-oxides. These compounds are involved in a double acid-base equilibrium (Scheme 2) and the pK_a_ values of some examples have been reported in the literature [21,22]. The averages of these values are approximately 2.2 for pK_a1_ and 6.3 for pK_a2_. We posited that although the products formed in our study deviated from the examples that had been studied previously in that they bore different substituents, the pKa values would be analogous. With this in mind, we modified our work-up such that we directly acidified the crude product mixture to a pH of between 2 and 6 in the reaction vessel directly after the second heating step. When the pH was in this range, precipitation was observed, but outside of this pH range, the species formed (**5** and **6**) are soluble in the reaction medium, meaning that product isolation is challenging. Cooling the product mixture in the vessel using an ice bath led to further precipitation. Filtering the solid and allowing it to dry furnished us with the desired product in pure form. Not only does this approach allow us to obtain the products in far superior isolated yield as compared to the previous purification protocol (Table 1), it also negates the need for extraction and hence the use of organic solvents. With this work-up protocol in hand, we proceeded to isolate 42 benzimidazole *N*-oxide derivatives bearing aryl substituents in the 2-position of the heterocycle ring. Excellent to good yields were obtained in each case (Table 2). The synthetic methodology was applicable to a range of benzylamine substrates as well as heterocyclic amines, and a range of functional groups on the aromatic ring of the amine were well tolerated. Extending the method to use with phenethylamine was possible, albeit at the expense of isolated yield. We attribute this to the difference in the physical properties of the product. We observed full conversion from **1** to **3** and also a high conversion of **3** to **4**. Therefore, a loss of product is occurring in the precipitation process.

### 2.3. Expanding the Substrate Scope, Applicability, and Scale of the Reaction

In an attempt to broaden the substrate scope of the reaction, we first turned attention to the nitrochlorobenzene component. We attempted the reaction using 2-nitrochlorobenzene (**1d**) as a substrate, but no benzimidazole *N*-oxide formation was observed. This follows the trend previously noted for similar substrates [20,22,36]. The lack of highly electron-withdrawing groups near the NH on **3** reduces the acidity of the NH proton. The deprotonation of **3** is proposed as the first step of the cyclization mechanism, and thus, reducing the propensity for this impacts the efficacy of the reaction [22,37]. Moving to the amine component, while we have previously shown that the reaction can be performed using alkylamines [29], product isolation using our new approach proved challenging.

Reactions were performed using a 1:2 stoichiometric ratio of nitrochlorobenzene derivative to benzylamine, the second equivalent of amine serving as a sacrificial scavenger of the HCl formed in the S_N_Ar step. In the case of the benzylamines used in our study, the fact that a second equivalent of amine was employed did not prove to be an issue; the amines are readily commercially available and inexpensive. However, should the amine be challenging to source or prepare, an alternative approach would be to use a cheap tertiary amine as the scavenger. To this end, we performed the reaction of **1a** with **2a** using a 1:1 stoichiometric ratio of the two components and added one equivalent of triethylamine as an acid scavenger. We obtained an 85% isolated yield of **4aa** as compared to 95% when using two equivalents of **2a**. This shows the applicability of the modified approach, albeit with a slight diminution in product yield.

We attempted to scale up the reaction by process intensification. Our methodology was developed and the substrate scope probed at the 0.3 mmol scale using a 10-mL capacity microwave vial. On increasing the scale five-fold to 1.5 mmol, an identical product yield of **4aa** was obtained in the case of the reaction of **1a** with **2a**. However, performing the reaction at the 3 mmol scale led to a significant drop in yield. We have shown previously that for the preparation of this class of heterocycle, continuous-flow processing offers a valuable approach for further scale-up [29].

## 3. Materials and Methods 

### 3.1. General

All reactions were performed using a CEM Discover SP monomode microwave unit, in a closed vessel. Temperature was measured by means of an infrared (IR) temperature sensor located in the floor of the microwave unit. Nuclear magnetic resonance (NMR) spectra (^1^H, ^13^C, ^19^F) were performed at 300 K using either a Brüker Avance Ultra Shield 300 MHz, Brüker DRX-400 400 MHz, or Brüker Avance 500 MHz spectrometer (Billerica, MA, USA). ^1^H NMR spectra were referenced to residual non-deuterated dimethylsulfoxide (2.50 ppm) in deuterated dimethylsulfoxide (*d*_6_-DMSO). ^13^C NMR spectra were referenced to DMSO (39.52 ppm). ^19^F NMR spectra were referenced to hexafluorobenzene (−164.9 ppm). Melting points were determined with a Barnstead Electrotherm 1001D Mel-Temp capillary melting point apparatus in open capillaries and are uncorrected. High-resolution mass spectra were performed on a JEOL AccuTOF-DART SVP 100 unit employing a positive direct analysis in real time (DART) ionization method, using polyethylene glycol (PEG) as the internal standard. Reactions were monitored by ^1^H NMR spectroscopy.

### 3.2. Chemicals

Deuterated dimethylsulfoxide (*d*_6_-DMSO) was purchased from Cambridge Isotope Laboratories. The *o*-nitro-chlorobenzene derivatives were purchased from Oakwood Chemicals or Alfa Aesar. Ethanol was obtained from Pharmaco. Potassium carbonate was purchased from J. T. Baker. Hexafluorobenzene was purchased from Oakwood Chemicals. All amines employed were purchased from Oakwood Chemicals and used without purification.

### 3.3. Representative Procedure for the Synthesis of Benzimidazole-N-oxides

7-Nitro-2-phenyl-1H-benzimidazole 3-oxide (**4aa**): 2-Chloro-1,3-dinitrobenzene (0.3 mmol, 1 eq), benzylamine (0.6 mmol, 2 eq) and ethanol (3 mL) were added to a 10 mL-capacity glass tube equipped with a magnetic stir bar. The reaction mixture was sealed with a septum and placed into a CEM Discover SP microwave unit. The contents of the vessel was heated to 120 °C using an initial microwave power of 100 W and held at this temperature for 20 min, the microwave power automatically fluctuating to hold the reaction mixture at the desired temperature. The reaction mixture was stirred constantly. After the allotted time, the reaction mixture was allowed to cool to below 50 °C before taking the vessel out of the microwave unit. The septum was removed, 0.5 M aqueous potassium carbonate (2 mL) was added, and the septum was then replaced. The mixture was then replaced into the microwave unit and heated at 120 °C for 20 min using an identical protocol to that for the first step of the reaction. After cooling, removing from the microwave unit, and de-capping, water (5 mL) was added directly into the glass reaction vessel. The product mixture was acidified with 2 M hydrochloric acid to a pH of between 2 and 5 at which point a yellow solid precipitated from the solution. The stir bar was removed and the reaction vessel placed into an ice bath for 1 h to promote further product precipitation. After this time, the solid product was removed by filtration and dried at 80 °C. Analytically pure **4aa** was obtained as a yellow powder (73 mg, 95%); characterized by melting point determination, ^1^H- and ^13^C-NMR spectroscopy, and high-resolution mass spectrometry (see the Supplementary Materials). 

## 4. Conclusions

Using a one-pot-two step protocol, a library of 42 novel 2-aryl benzimidazole-3-oxide derivatives were prepared. The use of microwave heating as a tool greatly facilitated the procedure, a total reaction time of 40 min being all that was needed. Ethanol and water were employed as solvents. This, in conjunction with a work-up protocol that involves a simple precipitation and filtration, thereby obviating the need for aqueous-organic extraction, offers a sustainable route to the synthesis and isolation of this class of compound. The products obtained bear diverse structural characteristics, thereby furnishing them with potentially interesting biological activity; this is an area of ongoing study in our group. In addition, post-synthesis modification of the benzimidazole N-oxide products could further yield compounds of biological and chemical interest.

## Figures and Tables

**Table 1:** Comparative isolated yields obtained for four representative products using the previous and improved work-up protocols.

Product	Isolated Yield (%)	
	Previously Reported Work-Up	New Work-Up
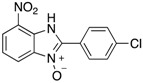	31	86
**4ai**		
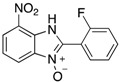	14	84
**4ac**		
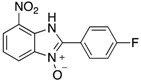	31	85
**4ah**		
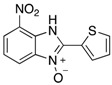	20	79
**4al**		

**Table 2:** The library of 2-aryl-benzimidazole 3-oxides prepared in this study ^a^.

Amine	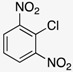	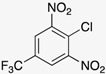	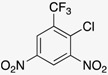
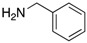	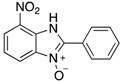	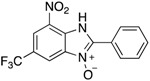	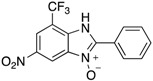
**2a**	**4aa** (95%; 95% *)	**4ba** (88%)	**4ca** (88%)
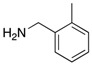	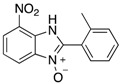	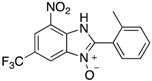	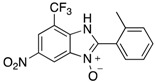
**2b**	**4ab** (88%)	**4bb** (98%)	**4cb** (90%)
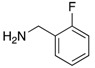	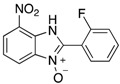	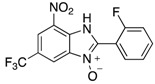	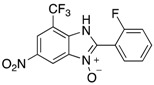
**2c**	**4ac** (76%)	**4bc** (93%)	**4cc** (83%)
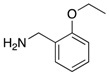	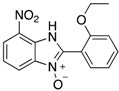	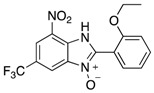	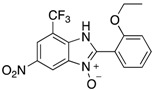
**2d**	**4ad** (99%)	**4bd** (86%)	**4cd** (76%)
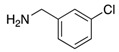	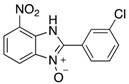	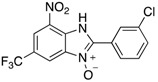	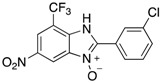
**2e**	**4ae** (99%)	**4be** (90%)	**4ce** (78%)
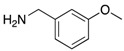	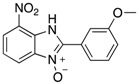	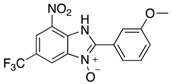	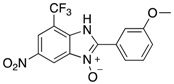
**2f**	**4af** (98%)	**4bf** (91%)	**4cf** (84%)
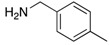	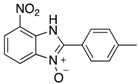	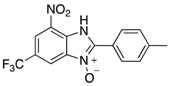	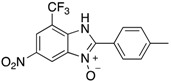
**2g**	**4ag** (51%)	**4bg** (99%)	**4cg** (92%)
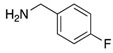	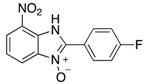	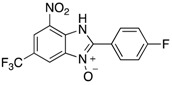	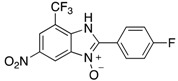
**2h**	**4ah** (77%)	**4bh** (96%)	**4ch** (67%)
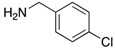	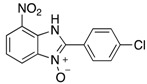	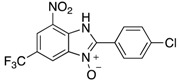	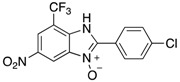
**2i**	**4ai** (86%)	**4bi** (89%)	**4ci** (99%)
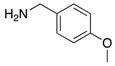	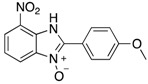	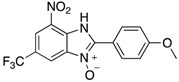	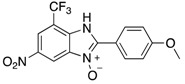
**2j**	**4aj** (88%)	**4bj** (85%)	**4cj** (80%)
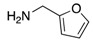	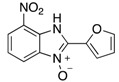	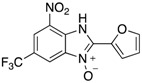	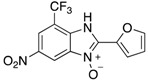
**2k**	**4ak** (74%)	**4bk** (96%)	**4ck** (94%)
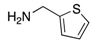	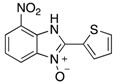	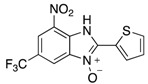	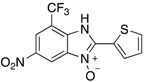
**2l**	**4al** (79%)	**4bl** (98%)	**4cl** (84%)
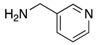	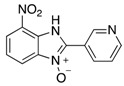	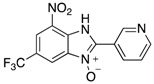	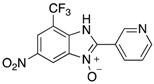
**2m**	**4am** (61%)	**4bm** (87%)	**4cm** (84%)
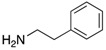	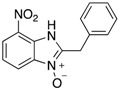	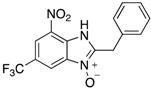	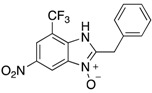
**2n**	**4an** (42%)	**4bn** (52%)	**4cn** (73%)

^a^ Isolated yields in parentheses. * Isolated yield of reaction performed on 1.5 mmol scale.

## Supplementary Materials

The following are available online, NMR spectra and high-resolution mass spectra of products.
