# Supplementary material for: One-Pot Two-Step Synthesis of 2-Aryl benzimidazole N-oxides Using Microwave Heating as a Tool
Source: Molecules. 2019 Oct 9;24(20):3639. doi: 10.3390/molecules24203639 (PMC6832536; doi:10.3390/molecules24203639)

One-pot two-step synthesis of 2-aryl benzimidazole *N*-oxides using microwave heating as a tool

Fabrizio Politano, Ana K. Gran-Magano and Nicholas E. Leadbeater*

Department of Chemistry, University of Connecticut, 55 North Eagleville Road, Storrs, CT 06269-3060, USA

* *Correspondence: nicholas.leadbeater@uconn.edu*

SUPPORTING INFORMATION

General Considerations S2

Representative Procedure for the Synthesis of Benzimidazole-*N*-oxides S3
General procedure for synthesis, isolation procedures, and spectral characterization information for 2-aryl-benzimidazole N-oxides

^{1}H-NMR Spectra of Synthesized Compounds S19

^{13}C-NMR Spectra of Synthesized Compounds S61

^{19}F-NMR Spectra of Synthesized Compounds S103

General Considerations

All reactions were performed using a CEM Discover SP microwave unit, in a closed vessel. Temperature was measured by means of an IR temperature sensor located in the floor of the microwave unit. NMR spectra (^{1}H, ^{13}C, ^{19}F) were performed at 300 K using either a Brüker Avance Ultra Shield 300 MHz, Brüker DRX-400 400 MHz, or Brüker Avance 500 MHz spectrometer. ^{1}H NMR spectra were referenced to residual non-deuterated dimethylsulfoxide (2.50 ppm) in d_6-DMSO. ^{13}C NMR spectra were referenced to DMSO (39.52 ppm). ^{19}F NMR spectra were referenced to hexafluorobenzene (-164.9 ppm). Melting points were determined with a Barnstead Electrotherm 1001D Mel-Temp capillary melting point apparatus in open capillaries and are uncorrected. High-resolution mass spectra were performed on a JEOL AccuTOF-DART SVP 100 unit employing a positive direct analysis in real time (DART) ionization method, using PEG as the internal standard. Reactions were monitored by ^{1}H NMR spectroscopy.

Chemicals

Deuterated dimethylsulfoxide (d_6-DMSO) was purchased from Cambridge Isotope Laboratories. The *o*-nitro-chlorobenzene derivatives were purchased from Oakwood Chemicals or Alfa Aesar. Ethanol was obtained from Pharmaco. Potassium carbonate was purchased from J. T. Baker. Hexafluorobenzene was purchased from Oakwood Chemicals. All amines employed were purchased from Oakwood Chemicals and used without purification.

Representative Procedure for the Synthesis of Benzimidazole-N-oxides

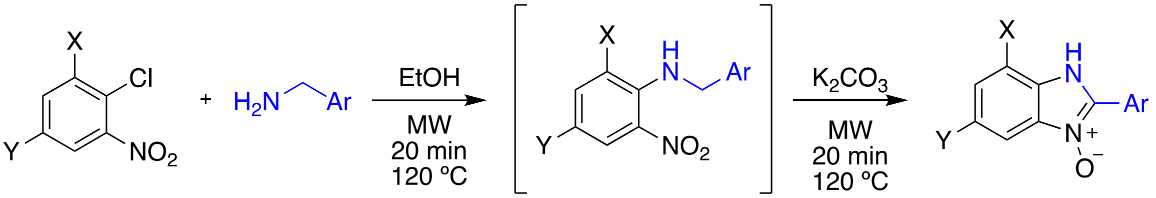

1a: X: $-NO_2$, Y: -H
1b: X: $-NO_2$, Y: $-CF_3$
1c: X: $-CF_3$, Y: $-NO_2$

2a-n

3
(not isolated)

4aa-4an
4ba-4bn
4ca-4cn

7-nitro-2-phenyl-1H-benzimidazole 3-oxide (**4aa**): 2-Chloro-1,3-dinitrobenzene (**1a**, 0.3 mmol, 1 eq), benzylamine (**2a**, 0.6 mmol, 2 eq) and ethanol (3 mL) were added to a 10 mL-capacity glass tube equipped with a magnetic stir bar. The reaction mixture was sealed with a septum and placed into a CEM Discover SP microwave unit. The contents of the vessel was heated to 120 °C using an initial microwave power of 100 W and held at this temperature for 20 min, the microwave power automatically fluctuating to hold the reaction mixture at the desired temperature. The reaction mixture was stirred constantly. After the allotted time, the reaction mixture was allowed to cool to below 50 °C before taking the vessel out of the microwave unit. The septum was removed, 0.5 M aqueous potassium carbonate (2 mL) added, and the septum then replaced. The mixture was then replaced into the microwave unit and heated at 120 °C for 20 min using an identical protocol to that for the first step of the reaction. After cooling, removing from the microwave unit, and de-capping, water (5 mL) was added directly into the glass reaction vessel. The product mixture was acidified with 2M hydrochloric acid to a pH of between 2 and 5 at which point a yellow solid precipitated from the solution. The stir bar was removed and the reaction vessel placed into an ice bath for 1 h to promote further product precipitation. After this time, the solid product was removed by filtration and dried at 80 °C to constant weight. Analytically pure **4aa** was obtained as a yellow powder (73 mg, 95%); characterized by melting-point determination, ^{1}H- and ^{13}C-NMR spectroscopy, and high-resolution mass spectrometry.

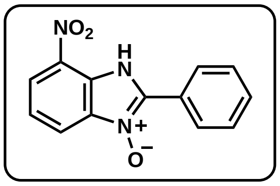

7-Nitro-2-phenyl-1*H*-benzimidazole 3-oxide (4aa). m.p.: decomposition at 238 °C. **^{1}H NMR:** (300 MHz, DMSO-d_6) δ 12.60 (s, 1H), 8.38 – 8.25 (m, 2H), 8.11 (dd, *J* = 8.0, 1.0 Hz, 1H), 8.01 (dd, *J* = 8.0, 0.9 Hz, 1H), 7.70 – 7.54 (m, 3H), 7.49 (t, *J* = 8.0 Hz, 1H). **^{13}C NMR:** (101 MHz, DMSO-d_6) δ 150.23, 138.13, 136.19, 131.32, 130.95, 128.87, 128.70, 127.70, 122.14, 119.21, 116.22. **HRMS (ESI):** calculated for $C_{13}H_{10}N_3O_3$ [M+H]$^+$ 256.0717, found 256.0733.

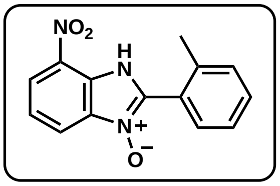

7-Nitro-2-(*o*-tolyl)-1*H*-benzimidazole 3-oxide (4ab). Synthesized following the procedure for **4aa**, starting from **1a** (0.3 mmol, 1 eq) and amine **2b** (0.6 mmol, 2 eq). Product **4ab** was obtained as a white powder (71 mg, 88%). **m.p.:** decomposition at 252 °C. **^{1}H NMR:** (400 MHz, DMSO-d6) δ 12.27 (s, 1H), 8.11 (d, J = 7.9 Hz, 1H), 8.00 (d, J = 8.0 Hz, 1H), 7.63 (d, J = 7.6 Hz, 1H), 7.50 (td, J = 7.7, 3.2 Hz, 2H), 7.47 – 7.34 (m, 2H), 2.38 (s, 3H). **^{13}C NMR:** (101 MHz, DMSO-d6) δ 151.85, 138.22, 138.03, 134.93, 131.26, 130.59, 130.53, 130.35, 127.50, 125.61, 122.03, 118.87, 116.18, 19.77. **HRMS (ESI):** calculated for $C_{14}H_{12}N_3O_3$ [M+H]$^+$ 270.0873, found 270.0883.

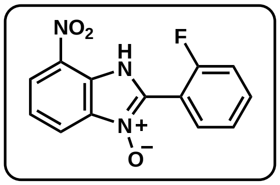

2-(2-Fluorophenyl)-7-nitro -1*H*-benzimidazole 3-oxide (4ac). Synthesized following the procedure for **4aa**, starting from **1a** (0.3 mmol, 1 eq) and amine **2c** (0.6 mmol, 2 eq). Product **4ac** was obtained as a pale yellow powder (62 mg, 76%). **m.p.:** decomposition at 292 °C. **^{1}H NMR** (300 MHz, DMSO-d_6) δ 12.45 (s, 1H), 8.14 (dd, *J* = 8.0, 1.0 Hz, 1H), 8.03 (dd, *J* = 8.1, 1.0 Hz, 1H), 7.85 (td, *J* = 7.5, 1.8 Hz, 1H), 7.70 (dddd, *J* = 8.9, 7.2, 5.3, 1.8 Hz, 1H), 7.54 (t, *J* = 8.0 Hz, 1H), 7.50 – 7.40 (m, 2H). **^{13}C NMR** (126 MHz, DMSO-d_6) δ 159.85 (d, *J* = 251.9 Hz), 147.52, 138.38, 135.20, 133.21 (d, *J* = 8.4 Hz), 132.20, 131.47, 124.76 (d, *J* = 3.5 Hz), 122.51, 119.23, 116.46, 116.30 (d, *J* = 21.0 Hz), 116.03 (d, *J* = 14.1 Hz). **^{19}F NMR** (376 MHz, DMSO-d_6) δ -102.85 (ddd, *J* = 10.7, 7.2, 5.4 Hz). **HRMS (ESI):** calculated for $C_{13}H_9FN_3O_3$ [M+H]+ 274.0622, found 274.0626.

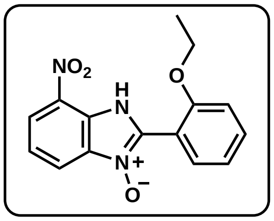

2-(2-Ethoxyphenyl)-7-nitro-1*H*-benzimidazole 3-oxide (4ad). Synthesized following the procedure for **4aa**, starting from **1a** (0.3 mmol, 1 eq) and amine **2d** (0.6 mmol, 2 eq). Product **4ad** was obtained as a white powder (90 mg, 99%). **m.p.:** decomposition at 240 °C. **^{1}H NMR** (300 MHz, DMSO-*d*$_6$) δ 11.95 (s, 1H), 8.09 (dd, *J* = 8.0, 1.0 Hz, 1H), 7.98 (dd, *J* = 8.1, 1.0 Hz, 1H), 7.63 – 7.52 (m, 2H), 7.49 (t, *J* = 8.0 Hz, 1H), 7.23 (d, *J* = 8.3 Hz, 1H), 7.11 (td, *J* = 7.5, 1.0 Hz, 1H), 4.14 (q, *J* = 7.0 Hz, 2H), 1.24 (t, *J* = 7.0 Hz, 3H). **^{13}C NMR** (126 MHz, DMSO-*d*$_6$) δ 157.56, 151.24, 138.61, 135.67, 132.67, 132.28, 131.96, 122.24, 120.66, 119.14, 117.99, 116.61, 113.32, 64.37, 14.85. **HRMS (ESI):** calculated for $C_{15}H_{14}N_3O_4$ [M+H]+ 300.0979, found 300.0976.

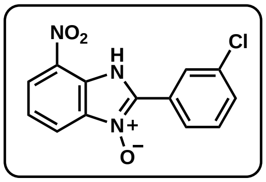

2-(3-Chlorophenyl)-7-nitro-1*H*-benzimidazole 3-oxide (4ae). Synthesized following the procedure for **4aa**, starting from **1a** (0.3 mmol, 1 eq) and amine **2e** (0.6 mmol, 2 eq). Product **4ae** was obtained as a yellow powder (86 mg, 99%). **m.p.:** decomposition at 254 °C. **^{1}H NMR** (300 MHz, DMSO-*d*$_6$) δ 12.78 (s, 1H), 8.34 – 8.21 (m, 2H), 8.11 (d, *J* = 7.9 Hz, 1H), 8.00 (d, *J* = 7.9 Hz, 1H), 7.64 (dd, *J* = 4.8, 2.3 Hz, 2H), 7.49 (t, *J* = 8.1 Hz, 1H). **^{13}C NMR** (101 MHz, DMSO-*d*$_6$) δ 148.53, 138.20, 136.15, 133.51, 131.13, 130.85, 130.68, 129.62, 127.99, 127.22, 122.47, 119.47, 116.47. **HRMS (ESI):** calculated for $C_{13}H_9ClN_3O_3$ [M+H]+ 290.0327, found 290.0329.

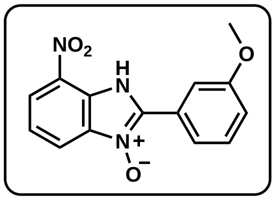

2-(3-Methoxyphenyl)-7-nitro-1*H*-benzimidazole 3-oxide (4af). Synthesized following the procedure for **4aa**, starting from **1a** (0.3 mmol, 1 eq) and amine **2f** (0.6 mmol, 2 eq). Product **4af** was obtained as a yellow powder (84 mg, 98%). **m.p.:** decomposition at 243 °C. **^{1}H NMR** (300 MHz, DMSO-*d*$_6$) δ 12.64 (s, 1H), 8.11 (d, *J* = 7.9 Hz, 1H), 8.00 (d, *J* = 8.0 Hz, 1H), 7.94 – 7.86 (m, 1H), 7.84 (t, *J* = 2.0 Hz, 1H), 7.53 (t, *J* = 8.1 Hz, 1H), 7.49 (t, *J* = 8.0 Hz, 1H), 7.18 (dd, *J* = 8.3, 2.6 Hz, 1H), 3.86 (s, 3H). **^{13}C NMR** (75 MHz, DMSO-*d*$_6$) δ 159.29, 150.03, 138.11, 136.25, 131.20, 130.08, 128.87, 122.21, 121.10, 119.29, 116.61, 116.34, 113.97, 55.38. **HRMS (ESI):** calculated for $C_{14}H_{12}N_3O_4$ [M+H]+ 286.0822, found 274.0626.

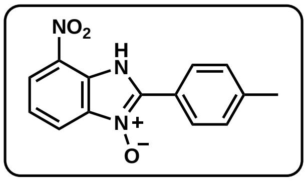

7-Nitro-2-(*p*-tolyl)-1*H*-benzimidazole 3-oxide (4ag). Synthesized following the procedure for **4aa**, starting from **1a** (0.3 mmol, 1 eq) and amine **2g** (0.6 mmol, 2 eq). Product **4ag** was obtained as a yellow powder (41 mg, 51%). **m.p.:** decomposition at 253 °C. **^{1}H NMR:** (300 MHz, DMSO-d6) δ 12.71 (s, 1H), 8.22 (d, J = 8.3 Hz, 2H), 8.08 (dd, J = 8.1, 1.0 Hz, 1H), 7.95 (dd, J = 8.1, 0.9 Hz, 1H), 7.45 (t, J = 8.0 Hz, 1H), 7.40 (d, J = 7.9 Hz, 2H), 2.40 (s, 3H). **^{13}C NMR:** (101 MHz, DMSO-d6) δ 150.41, 140.97, 138.00, 136.35, 131.43, 129.44, 128.64, 124.98, 121.88, 119.12, 116.16, 21.15. **HRMS (ESI):** calculated for $C_{14}H_{12}N_3O_3$ [M+H]+ 270.0873, found 270.0866.

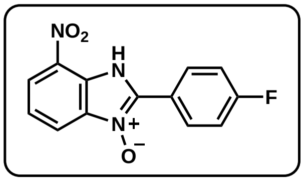

2-(4-Fluorophenyl)-7-nitro-1*H*-benzimidazole 3-oxide (4ah). Synthesized following the procedure for **4aa**, starting from **1a** (0.3 mmol, 1 eq) and amine **2h** (0.6 mmol, 2 eq). Product **4ah** was obtained as a yellow powder (63 mg, 77%). **m.p.:** decomposition at 270 °C. **^{1}H NMR:** (400 MHz, DMSO-d_6) δ 12.66 (s, 1H), 8.40 – 8.36 (m, 2H), 8.10 (dd, *J* = 7.9, 1.1 Hz, 1H), 8.00 (dd, *J* = 8.0, 1.0 Hz, 1H), 7.53 – 7.43 (m, 3H). **^{13}C NMR:** (101 MHz, DMSO-d_6) δ 163.61 (d, *J* = 249.7 Hz), 149.37, 138.12, 136.18, 131.28, 131.25 (d, *J* = 8.7 Hz), 124.30 (d, *J* = 2.9 Hz), 122.23, 119.32, 116.30, 116.09 (d, *J* = 21.9 Hz). **^{19}F NMR** (376 MHz, DMSO-d_6) δ -111.24 (tt, *J* = 8.9, 5.5 Hz). **HRMS (ESI):** calculated for $C_{13}H_9FN_3O_3$ [M+H]+ 274.0622, found 274.0610.

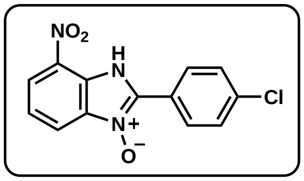

2-(4-Chlorophenyl)-7-nitro-1*H*-benzimidazole 3-oxide (4ai). Synthesized following the procedure for **4aa**, starting from **1a** (0.3 mmol, 1 eq) and amine **2i** (0.6 mmol, 2 eq). Product **4ai** was obtained as a pale yellow powder (75 mg, 86%). **m.p.:** decomposition at 266 °C. **^{1}H NMR:** (300 MHz, DMSO-d_6) δ 12.71 (s, 1H), 8.41 – 8.29 (m, 2H), 8.12 (dd, *J* = 8.0, 1.0 Hz, 1H), 8.02 (dd, *J* = 8.1, 1.0 Hz, 1H), 7.76 – 7.62 (m, 2H), 7.50 (t, *J* = 8.0 Hz, 1H). **^{13}C NMR:** (101 MHz, DMSO-d_6) δ 149.02, 138.14, 136.16, 135.79, 131.22, 130.33, 129.04, 126.53, 122.35, 119.39, 116.34. **HRMS (ESI):** calculated for $C_{13}H_9ClN_3O_3$ [M+H]+ 290.0327, found 290.0314.

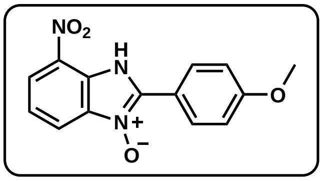

2-(4-Methoxyphenyl)-7-nitro-1*H*-benzimidazole 3-oxide (4aj). Synthesized following the procedure for **4aa**, starting from **1a** (0.3 mmol, 1 eq) and amine **2j** (0.6 mmol, 2 eq). Product **4aj** was obtained as a pale yellow powder (75 mg, 88%). **m.p.:** decomposition at 258 °C. **^{1}H NMR:** (300 MHz, DMSO-d6) δ 12.51 (s, 1H), 8.36 – 8.24 (m, 2H), 8.07 (dd, J = 8.0, 1.0 Hz, 1H), 7.95 (dd, J = 8.0, 1.0 Hz, 1H), 7.44 (t, J = 8.0 Hz, 1H), 7.21 – 7.08 (m, 2H), 3.86 (s, 3H). **^{13}C NMR:** (101 MHz, DMSO-d6) δ 161.36, 150.29, 137.76, 136.26, 131.40, 130.41, 121.64, 119.90, 119.00, 115.88, 114.30, 55.41. **HRMS (ESI):** calculated for $C_{14}H_{12}N_3O_4$ [M+H]+ 286.0822, found 286.0814.

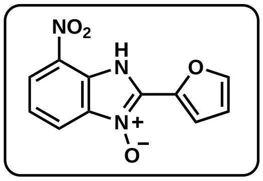

2-(Furan-2-yl)-7-nitro-1*H*-benzimidazole 3-oxide (4ak). Synthesized following the procedure for **4aa**, starting from **1a** (0.3 mmol, 1 eq) and amine **2k** (0.6 mmol, 2 eq). Product **4ak** was obtained as a yellow powder (68 mg, 74%). **m.p.:** decomposition at 188 °C. **^{1}H NMR:** (300 MHz, DMSO-d6) δ 12.75 (s, 1H), 8.14 – 8.04 (m, 2H), 7.95 (dd, J = 8.1, 1.1 Hz, 1H), 7.54 – 7.40 (m, 2H), 6.82 (dd, J = 3.5, 1.7 Hz, 1H). **^{13}C NMR:** (126 MHz, DMSO-d6) δ 146.21, 143.13, 142.03, 138.06, 135.50, 131.82, 122.23, 119.27, 115.92, 115.16, 112.55. **HRMS (ESI):** calculated for $C_{11}H_8N_3O_4$ [M+H]+ 246.0509, found 246.0509.

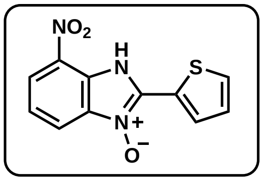

7-Nitro-2-(thiophen-2-yl)-1*H*-benzimidazole 3-oxide (4al). Synthesized following the procedure for **4aa**, starting from **1a** (0.3 mmol, 1 eq) and amine **2l** (0.6 mmol, 2 eq). Product **4al** was obtained as a light orange powder (62 mg, 79%). **m.p.:** decomposition at 208 °C. **^{1}H NMR:** (400 MHz, DMSO-d6) δ 12.78 (s, 1H), 8.17 (d, J = 3.7 Hz, 1H), 8.09 (d, J = 8.0 Hz, 1H), 7.99 – 7.92 (m, 2H), 7.46 (t, J = 8.0 Hz, 1H), 7.33 (t, J = 4.4 Hz, 1H). **^{13}C NMR:** (101 MHz, DMSO-d6) δ 146.45, 137.72, 135.79, 131.72, 131.35, 130.37, 129.10, 128.54, 122.03, 119.37, 115.88. **HRMS (ESI):** calculated for $C_{11}H_8N_3O_3S$ [M+H]+ 262.0281, found 262.0313.

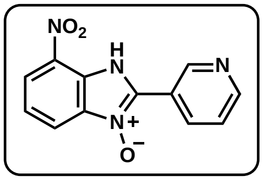

7-Nitro-2-(pyridin-3-yl)-1*H*-benzimidazole 3-oxide (4am). Synthesized following the procedure for **4aa**, starting from **1a** (0.3 mmol, 1 eq) and amine **2m** (0.6 mmol, 2 eq). Product **4am** was obtained as a dark orange powder (47 mg, 61%). **m.p.:** decomposition at 285 °C. **^{1}H NMR:** (300 MHz, DMSO-d6) δ 12.32 (s, 1H), 8.03 (dd, J = 8.1, 1.0 Hz, 1H), 7.90 (dd, J = 8.1, 1.0 Hz, 1H), 7.43 (t, J = 8.0 Hz, 1H), 7.41 – 7.26 (m, 4H), 7.25 (dt, J = 7.1, 3.0 Hz, 1H), 4.34 (s, 2H). **^{13}C NMR:** (101 MHz, DMSO-d6) δ 151.36, 149.02, 148.06, 138.28, 136.04, 135.98, 131.36, 124.07, 123.97, 122.57, 119.46, 116.43. **HRMS (ESI):** calculated for $C_{12}H_9N_4O_3$ [M+H]+ 257.0669, found 257.0688.

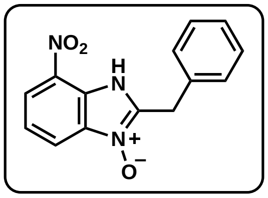

2-Benzyl-7-nitro-1*H*-benzimidazole 3-oxide (4an). Synthesized following the procedure for **4aa**, starting from **1a** (0.3 mmol, 1 eq) and amine **2n** (0.6 mmol, 2 eq). Product **4an** was obtained as a yellow powder (34 mg, 42%). **m.p.:** decomposition at 147 °C. **^{1}H NMR:** (400 MHz, DMSO-d6) δ 12.79 (s, 1H), 9.43 (d, J = 2.1 Hz, 1H), 8.78 (dd, J = 4.8, 1.7 Hz, 1H), 8.62 (dt, J = 8.1, 2.0 Hz, 1H), 8.13 (d, J = 7.8 Hz, 1H), 8.05 (d, J = 8.0 Hz, 1H), 7.66 (dd, J = 8.1, 4.8 Hz, 1H), 7.53 (t, J = 8.0 Hz, 1H). **^{13}C NMR:** (126 MHz, DMSO-d6) δ 153.64, 137.99, 136.25, 134.93, 131.29, 128.94, 128.72, 126.90, 121.76, 118.73, 115.96, 31.74. **HRMS (ESI):** calculated for $C_{14}H_{12}N_3O_3$ [M+H]+ 270.0873, found 270.0918.

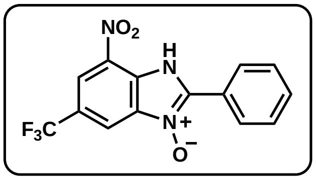

7-Nitro-2-phenyl-5-trifluoromethyl-1*H*-benzimidazole 3-oxide (4ba). Synthesized following the procedure for **4aa**, starting from **1b** (0.3 mmol, 1 eq) and amine **2a** (0.6 mmol, 2 eq). Product **4ba** was obtained as a white powder (85 mg, 88%). **m.p.:** decomposition at 271 °C. **^{1}H NMR:** (300 MHz, DMSO-d6) δ 13.00 (s, 1H), 8.42 – 8.31 (m, 4H), 7.66 (p, J = 3.4 Hz, 3H). **^{13}C NMR:** 13C NMR (126 MHz, DMSO-d6) δ 152.84, 137.98, 136.36, 133.45, 131.66, 129.02, 128.98, 127.06, 123.65 (q, J = 272.8 Hz), 122.28 (q, J = 33.6 Hz), 115.77 (d, J = 4.2 Hz), 113.13 (d, J = 3.9 Hz). **^{19}F NMR:** (377 MHz, DMSO-d6) δ -61.70. **HRMS (ESI):** calculated for $C_{14}H_9F_3N_3O_3$ [M+H]+ 324.0591, found 324.0607.

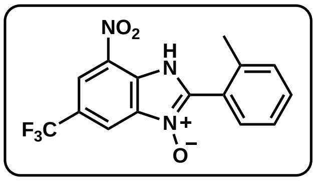

7-Nitro-2-(*o*-tolyl)-5-trifluoromethyl-1*H*-benzimidazole 3-oxide (4bb). Synthesized following the procedure for **4aa**, starting from **1b** (0.3 mmol, 1 eq) and amine **2b** (0.6 mmol, 2 eq). Product **4bb** was obtained as a pale yellow powder (100 mg, 98%). **m.p.:** decomposition at 293 °C. **^{1}H NMR:** (500 MHz, DMSO-d6) δ 12.69 (s, 1H), 8.39 (d, J = 1.6 Hz, 1H), 8.36 (d, J = 1.5 Hz, 1H), 7.67 (dd, J = 7.7, 1.3 Hz, 1H), 7.53 (td, J = 7.5, 1.4 Hz, 1H), 7.52 – 7.38 (m, 2H), 2.39 (s, 3H). **^{13}C NMR:** 13C NMR (126 MHz, DMSO-d6) δ 154.61, 138.19, 138.13, 135.09, 133.32, 130.84, 130.71, 130.63, 126.82, 125.75, 123.66 (q, J = 273.5 Hz), 122.36 (q, J = 33.6 Hz), 115.57 – 115.39 (m), 113.30 – 113.11 (m), 19.78. **^{19}F NMR:** (377 MHz, DMSO-d6) δ -61.70. **HRMS (ESI):** calculated for $C_{15}H_{11}F_3N_3O_3$ [M+H]+ 338.0747, found 338.0763.

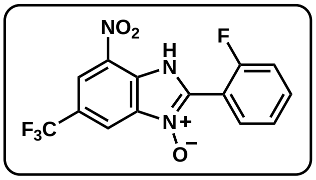

2-(2-Fluorophenyl)-7-nitro-5-trifluoromethyl-1*H*-benzimidazole 3-oxide (4bc). Synthesized following the procedure for **4aa**, starting from **1b** (0.3 mmol, 1 eq) and amine **2c** (0.6 mmol, 2 eq). Product **4bc** was obtained as a dark yellow powder (95 mg, 93%). **m.p.:** decomposition at 263 °C. **^{1}H NMR:** (400 MHz, DMSO-d6) δ 12.83 (s, 1H), 8.41 (s, 1H), 8.38 (s, 1H), 7.89 (td, J = 7.5, 1.8 Hz, 1H), 7.74 (tdd, J = 7.5, 5.3, 1.8 Hz, 1H), 7.56 – 7.43 (m, 2H). **^{13}C NMR:** (126 MHz, DMSO-d6) δ 160.33 (d, J = 252.8 Hz), 150.71, 138.78, 135.74, 134.23 (d, J = 8.5 Hz), 133.96, 132.66, 125.33 (d, J = 3.5 Hz), 124.03 (q, J = 272.4 Hz), 123.22 (q, J = 33.7 Hz), 116.89 (d, J = 20.9 Hz), 116.23 (d, J = 4.0 Hz), 115.84 (d, J = 13.7 Hz), 113.92 (d, J = 4.2 Hz). **^{19}F NMR:** (376 MHz, DMSO-d6) δ -61.83, -113.38 (ddd, J = 10.6, 7.2, 5.4 Hz). **HRMS (ESI):** calculated for $C_{14}H_8F_4N_3O_3$ [M+H]+ 342.0496, found 342.0511.

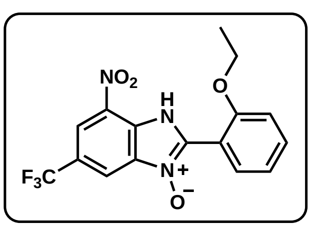

2-(2-Ethoxyphenyl)-7-nitro-5-trifluoromethyl-1*H*-benzimidazole 3-oxide (4bd). Synthesized following the procedure for **4aa**, starting from **1b** (0.3 mmol, 1 eq) and amine **2d** (0.6 mmol, 2 eq). Product **4bd** was obtained as a yellow powder (95 mg, 86%). **m.p.:** decomposition at 242 °C. **^{1}H NMR:** (400 MHz, DMSO-d6) δ 12.36 (s, 1H), 8.34 (s, 2H), 7.66 – 7.54 (m, 2H), 7.25 (d, J = 8.4 Hz, 1H), 7.13 (t, J = 7.5 Hz, 1H), 4.16 (q, J = 7.0 Hz, 2H), 1.25 (t, J = 6.9 Hz, 3H). **^{13}C NMR:** (101 MHz, DMSO-d6) δ 157.11, 153.56, 138.07, 135.19, 133.52, 132.72, 131.80, 123.67 (q, J = 272.1 Hz), 122.16 (q, J = 33.6 Hz), 120.28, 116.77, 115.27 (d, J = 4.0 Hz), 113.02 (d, J = 3.9 Hz), 112.95, 63.97, 14.34. **^{19}F NMR:**

(377 MHz, DMSO-d6) δ -61.72. **HRMS (ESI):** calculated for $C_{16}H_{13}F_3N_3O_4$ [M+H]+ 368.0853, found 368.0865.

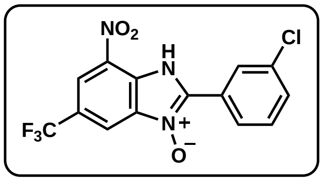

2-(3-Chlorophenyl)-7-nitro-5-trifluoromethyl-1*H*-benzimidazole 3-oxide (4be). Synthesized following the procedure for **4aa**, starting from **1b** (0.3 mmol, 1 eq) and amine **2e** (0.6 mmol, 2 eq). Product **4be** was obtained as a white powder (97 mg, 90%). **m.p.:** decomposition at 274 °C. **^{1}H NMR:** (400 MHz, DMSO-d6) δ 13.14 (s, 1H), 8.39 – 8.27 (m, 4H), 7.68 (dt, J = 15.5, 8.0 Hz, 2H). **^{13}C NMR:** (126 MHz, DMSO-d6) δ 151.14, 138.07, 136.27, 133.62, 133.21, 131.39, 131.03, 128.96, 128.21, 127.52, 123.59 (q, J = 272.5 Hz), 122.63 (q, J = 33.6 Hz), 115.96 (d, J = 2.5 Hz), 113.40 (d, J = 3.3 Hz). **^{19}F NMR:** (376 MHz, DMSO-d6) δ -61.79. **HRMS (ESI):** calculated for $C_{14}H_8ClF_3N_3O_3$ [M+H]+ 358.0201, found 358.0207.

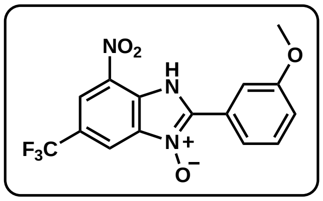

2-(3-Methoxyphenyl)-7-nitro-5-trifluoromethyl-1*H*-benzimidazole 3-oxide (4bf). Synthesized following the procedure for **4aa**, starting from **1b** (0.3 mmol, 1 eq) and amine **2f** (0.6 mmol, 2 eq). Product **4bf** was obtained as a pale yellow powder (96 mg, 91%). **m.p.:** decomposition at 235 ° C. **^{1}H NMR:** (300 MHz, DMSO-d6) δ 13.02 (s, 1H), 8.37 (d, J = 1.6 Hz, 1H), 8.34 (d, J = 1.6 Hz, 1H), 7.94 (dt, J = 7.7, 1.2 Hz, 1H), 7.87 (dd, J = 2.7, 1.5 Hz, 1H), 7.56 (t, J = 8.0 Hz, 1H), 7.23 (ddd, J = 8.5, 2.7, 0.9 Hz, 1H), 3.87 (s, 3H). **^{13}C NMR:** (126 MHz, DMSO-d6) δ 159.33, 152.61, 137.92, 136.39, 133.34, 130.17, 128.20, 123.67 (q, J = 272.1 Hz), 122.33 (q, J = 33.7 Hz), 121.34, 117.24, 115.76 (d, J = 4.1 Hz), 114.16, 113.10 (d, J = 4.1 Hz), 55.40. **^{19}F NMR:** (376 MHz, DMSO-d6) δ -61.80. **HRMS (ESI):** calculated for $C_{15}H_{11}F_3N_3O_4$ [M+H]+ 354.0696, found 354.0712.

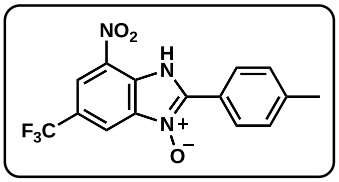

7-Nitro-2-(*p*-tolyl)-5-trifluoromethyl-1*H*-benzimidazole 3-oxide (4bg). Synthesized following the procedure for **4aa**, starting from **1b** (0.3 mmol, 1 eq) and amine **2g** (0.6 mmol, 2 eq). Product **4bg** was obtained as a yellow powder (100 mg, 99%). **m.p.:** decomposition at 313 °C. **^{1}H NMR:** (400 MHz, DMSO-d6) δ 8.10 (d, J = 8.2 Hz, 2H), 8.06 (d, J = 1.6 Hz, 1H), 7.74 (d, J = 1.5 Hz, 1H), 7.02 (d, J = 8.1 Hz, 2H), 2.26 (s, 3H). **^{13}C NMR:** (126 MHz, DMSO-d6) δ 152.34, 140.27, 137.30, 136.95, 133.11, 128.41, 128.19 – 127.87 (m), 124.62, 123.89 (q, J = 272.0 Hz), 120.04 (q, J = 33.1 Hz), 114.34 (d, J =

3.2 Hz), 112.27 (d, J = 3.8 Hz), 20.99. **^{19}F NMR:** (376 MHz, DMSO-d6) δ -61.67. **HRMS (ESI):** calculated for $C_{15}H_{11}F_3N_3O_3$ [M+H]+ 338.0747, found 338.0768.

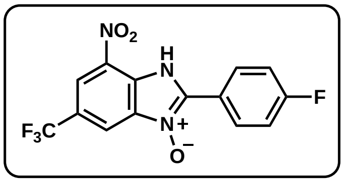

2-(4-Fluorophenyl)-7-nitro-5-trifluoromethyl-1*H*-benzimidazole 3-oxide (4bh). Synthesized following the procedure for **4aa**, starting from **1b** (0.3 mmol, 1 eq) and amine **2h** (0.6 mmol, 2 eq). Product **4bh** was obtained as a pale yellow powder (98 mg, 96%). **m.p.:** decomposition at 239 °C. **^{1}H NMR:** (400 MHz, DMSO-d6) δ 13.04 (s, 1H), 8.43 (ddd, J = 8.7, 5.4, 2.6 Hz, 2H), 8.38 (d, J = 1.6 Hz, 1H), 8.35 (d, J = 1.5 Hz, 1H), 7.56 – 7.45 (m, 2H). **^{13}C NMR:** 13C NMR (126 MHz, DMSO-d6) δ 164.02 (d, J = 250.6 Hz), 151.95, 137.91, 136.35, 133.43, 131.64 (d, J = 9.1 Hz), 123.70 (q, J = 272.4 Hz), 123.66 (d, J = 3.1 Hz), 122.37 (q, J = 33.6 Hz), 116.26 (d, J = 22.1 Hz), 115.85 (d, J = 4.0 Hz), 113.14 (d, J = 3.9 Hz). **^{19}F NMR:** (376 MHz, DMSO-d6) δ -61.79, -110.07 (tt, J = 8.8, 5.5 Hz). **HRMS (ESI):** calculated for $C_{14}H_8F_4N_3O_3$ [M+H]+ 342.0496, found 342.0515.

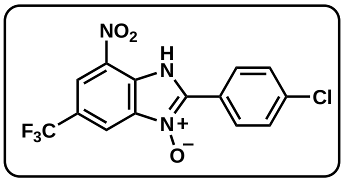

2-(4-Chlorophenyl)-7-nitro-5-trifluoromethyl-1*H*-benzimidazole 3-oxide (4bi). Synthesized following the procedure for **4aa**, starting from **1b** (0.3 mmol, 1 eq) and amine **2i** (0.6 mmol, 2 eq). Product **4bi** was obtained as a pale yellow powder (95 mg, 89%). **m.p.:** decomposition at 263 °C. **^{1}H NMR:** (300 MHz, DMSO-d6) δ 13.13 (s, 1H), 8.45 – 8.32 (m, 4H), 7.79 – 7.67 (m, 2H). **^{13}C NMR:** (126 MHz, DMSO-d6) δ 151.63, 137.97, 136.51, 136.34, 133.33, 130.62, 129.57 – 129.10 (m), 125.89, 123.63 (q, J = 272.3 Hz), 122.44 (q, J = 33.8 Hz), 115.89 (d, J = 3.9 Hz), 113.25 (d, J = 4.3 Hz). **^{19}F NMR:** (376 MHz, DMSO-d6) δ -61.77. **HRMS (ESI):** calculated for $C_{14}H_8ClF_3N_3O_3$ [M+H]+ 358.0201, found 358.0221.

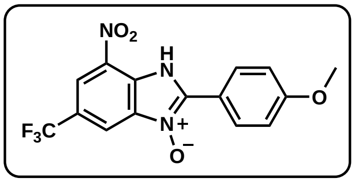

2-(4-Methoxyphenyl)-7-nitro-5-trifluoromethyl-1*H*-benzimidazole 3-oxide (4bj). Synthesized following the procedure for **4aa**, starting from **1b** (0.3 mmol, 1 eq) and amine **2j** (0.6 mmol, 2 eq). Product **4bj** was obtained as a yellow powder (90 mg, 85%). **m.p.:** decomposition at 268 °C. **^{1}H NMR:** (300 MHz, DMSO-d6) δ 13.06 (s, 1H), 8.41 – 8.29 (m, 2H), 8.30 (d, J = 1.6 Hz, 1H), 8.27 (d, J = 1.7 Hz, 1H), 7.24 – 7.12 (m, 2H), 3.88 (s, 3H). **^{13}C NMR:** (126 MHz, DMSO-d6) δ 161.81, 152.87, 137.41, 136.64, 133.68, 130.69, 123.76 (q, J = 272.1 Hz), 121.51 (q, J = 33.7 Hz), 119.37, 115.43 (d, J = 3.3

Hz), 114.35, 112.48 (d, J = 4.4 Hz), 55.48. **^{19}F NMR:** (376 MHz, DMSO-d6) δ -61.66. **HRMS (ESI):** calculated for $C_{15}H_{11}F_3N_3O_4$ [M+H]+ 354.0696, found 354.0715.

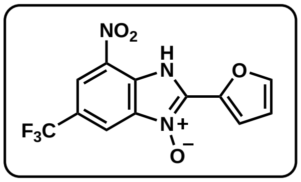

2-(Furan-2-yl)-7-nitro-5-trifluoromethyl-1*H*-benzimidazole 3-oxide (4bk). Synthesized following the procedure for **4aa**, starting from **1b** (0.3 mmol, 1 eq) and amine **2k** (0.6 mmol, 2 eq). Product **4bk** was obtained as a yellow powder (90 mg, 96%). **m.p.:** decomposition at 255 °C. **^{1}H NMR:** (400 MHz, DMSO-d6) δ 8.30 (s, 1H), 8.25 (s, 1H), 8.15 – 8.10 (m, 1H), 7.61 (d, J = 3.7 Hz, 1H), 6.84 (dd, J = 3.7, 2.0 Hz, 1H). **^{13}C NMR:** (126 MHz, DMSO-d6) δ 146.92, 145.52, 141.51, 137.75, 135.76, 134.01, 123.63 (q, J = 272.3 Hz), 122.08 (q, J = 33.7 Hz), 116.52, 115.67 (q, J = 3.6 Hz), 112.75, 112.54 (q, J = 4.0 Hz). **^{19}F NMR:** (376 MHz, DMSO-d6) δ -61.76. **HRMS (ESI):** calculated for $C_{12}H_7F_3N_3O_4$ [M+H]+ 314.0383, found 314.0412.

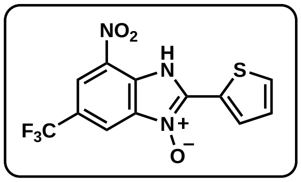

7-Nitro-2-(thiophen-2-yl)-5-trifluoromethyl-1*H*-benzimidazole 3-oxide (4bl). Synthesized following the procedure for **4aa**, starting from **1b** (0.3 mmol, 1 eq) and amine **2l** (0.6 mmol, 2 eq). Product **4bl** was obtained as a yellow powder (97 mg, 98%). **m.p.:** decomposition at 156 °C. **^{1}H NMR:** (300 MHz, DMSO-d6) δ 13.18 (s, 1H), 8.31 (d, J = 1.7 Hz, 1H), 8.30 (d, J = 1.6 Hz, 1H), 8.25 (dd, J = 3.8, 1.2 Hz, 1H), 8.03 (dd, J = 5.0, 1.2 Hz, 1H), 7.36 (dd, J = 5.0, 3.8 Hz, 1H). **^{13}C NMR:** (101 MHz, DMSO-d6) δ 149.07, 137.45, 135.97, 134.03, 132.62, 131.49, 128.74, 128.37, 123.65 (q, J = 272.3 Hz), 121.96 (q, J = 33.4 Hz), 115.83 (q, J = 3.5 Hz), 112.49 (d, J = 3.9 Hz). **^{19}F NMR:** (376 MHz, DMSO-d6) δ -61.70. **HRMS (ESI):** calculated for $C_{12}H_7F_3N_3O_3S$ [M+H]+ 330.0155, found 330.0177.

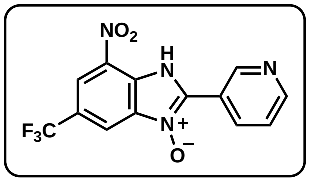

7-Nitro-2-(pyridin-3-yl)-5-trifluoromethyl-1*H*-benzimidazole 3-oxide (4bm). Synthesized following the procedure for **4aa**, starting from **1b** (0.3 mmol, 1 eq) and amine **2m** (0.6 mmol, 2 eq). Product **4bm** was obtained as a light orange powder (85 mg, 87%). **m.p.:** decomposition at 276 °C. **^{1}H NMR:** (400 MHz, DMSO-d6) δ 13.22 (s, 1H), 9.45 (d, J = 2.2 Hz, 1H), 8.80 (d, J = 4.6 Hz, 1H), 8.65 (dt, J = 8.0, 2.0 Hz, 1H), 8.37 (d, J = 1.6 Hz, 1H), 8.33 (d, J = 1.5 Hz, 1H), 7.67 (dd, J = 8.0, 4.8 Hz, 1H). **^{13}C NMR:** (126 MHz, DMSO-d6) δ 151.90, 150.73, 149.21, 138.13, 136.38, 136.14, 133.44, 124.08, 123.60 (q, J = 272.1

Hz), 123.54, 122.66 (q, J = 33.6 Hz), 115.94 (d, J = 3.9 Hz), 113.41 (d, J = 4.1 Hz). **19F NMR:** (376 MHz, DMSO-d6) δ -61.81. **HRMS (ESI):** calculated for $C_{13}H_8F_3N_4O_3$ [M+H]+ 325.0543, found 325.0566.

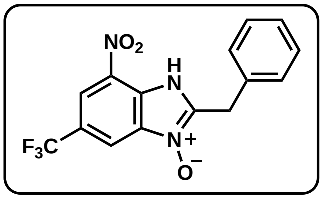

2-Benzyl-7-nitro-5-trifluoromethyl-1*H*-benzimidazole 3-oxide (4bn). Synthesized following the procedure for **4aa**, starting from **1b** (0.3 mmol, 1 eq) and amine **2n** (0.6 mmol, 2 eq). Product **4bn** was obtained as a pale orange powder (53 mg, 52%). **m.p.:** decomposition at 164 °C. **1H NMR:** (300 MHz, DMSO-d6) δ 8.24 (d, J = 1.6 Hz, 1H), 8.13 (s, 1H), 7.40 – 7.23 (m, 5H), 4.37 (s, 2H). **13C NMR:** (126 MHz, DMSO-d6) δ 156.59, 137.72, 135.74, 134.97, 133.31, 128.88, 128.58, 126.83, 123.49 (q, J = 272.3 Hz), 121.64 (q, J = 33.9 Hz), 114.98, 112.49, 31.68. **19F NMR:** (376 MHz, DMSO-d6) δ -61.71. **HRMS (ESI):** calculated for $C_{15}H_{11}F_3N_3O_3$ [M+H]+ 338.0747, found 338.0776.

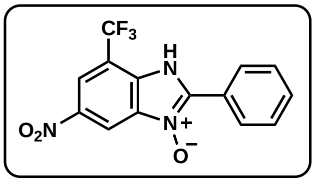

5-Nitro-2-phenyl-7-trifluoromethyl-1*H*-benzimidazole 3-oxide (4ca). Synthesized following the procedure for **4aa**, starting from **1c** (0.3 mmol, 1 eq) and amine **2a** (0.6 mmol, 2 eq). Product **4ca** was obtained as a yellow powder (85 mg, 88%). **m.p.:** decomposition at 239 °C. **1H NMR:** (300 MHz, DMSO-d6) δ 8.52 (d, J = 2.0 Hz, 1H), 8.34 (dd, J = 6.7, 3.1 Hz, 2H), 8.30 (d, J = 2.0 Hz, 1H), 7.61 (dd, J = 5.0, 2.0 Hz, 3H). **13C NMR:** (126 MHz, DMSO-d6) δ 152.81, 141.19, 138.44, 134.08, 131.35, 128.79, 127.30, 122.93 (q, J = 272.9 Hz), 118.42 (q, J = 33.3 Hz), 114.77 (q, J = 5.2 Hz), 109.78. **19F NMR:** (376 MHz, DMSO-d6) δ -62.28. **HRMS (ESI):** calculated for $C_{14}H_9F_3N_3O_3$ [M+H]+ 324.0591, found 324.0617.

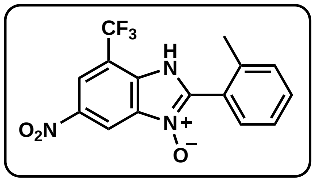

5-Nitro-2-(*o*-tolyl)-7-trifluoromethyl-1*H*-benzimidazole 3-oxide (4cb). Synthesized following the procedure for **4aa**, starting from **1c** (0.3 mmol, 1 eq) and amine **2b** (0.6 mmol, 2 eq). Product **4cb** was obtained as a white powder (91 mg, 90%). **m.p.:** decomposition at 278 °C. **1H NMR:** (300 MHz, DMSO-d6) δ 13.04 (s, 1H), 8.59 (d, J = 2.1 Hz, 1H), 8.34 (d, J = 2.1 Hz, 1H), 7.66 (dd, J = 7.7, 1.4 Hz, 1H), 7.50 (td, J = 7.5, 1.5 Hz, 1H), 7.44 – 7.32 (m, 2H), 2.37 (s, 3H). **13C NMR:** (101 MHz, DMSO-d6) δ 154.58, 141.66, 138.34, 138.14, 132.80, 130.72, 130.53, 130.42, 126.79, 125.66, 122.91 (q, J = 273.1 Hz), 118.78 (q, J = 33.3 Hz), 114.74 (q, J = 5.2 Hz), 110.10, 19.74. **19F NMR:** (376 MHz, DMSO-d6) δ -62.25. **HRMS (ESI):** calculated for $C_{15}H_{11}F_3N_3O_3$ [M+H]+ 338.0747, found 338.0786.

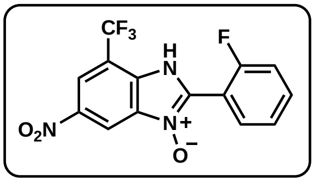

2-(2-Fluorophenyl)-5-nitro-7-trifluoromethyl-1*H*-benzimidazole 3-oxide (4cc). Synthesized following the procedure for **4aa**, starting from **1c** (0.3 mmol, 1 eq) and amine **2c** (0.6 mmol, 2 eq). Product **4cc** was obtained as a pale yellow powder (85 mg, 83%). **m.p.:** decomposition at 258 °C. **^{1}H NMR:** (300 MHz, DMSO-d6) δ 8.71 (d, J = 2.2 Hz, 1H), 8.40 (d, J = 2.1 Hz, 1H), 7.88 (td, J = 7.4, 1.8 Hz, 1H), 7.74 (dddd, J = 8.8, 7.2, 5.3, 1.8 Hz, 1H), 7.58 – 7.41 (m, 2H). **^{13}C NMR:** (126 MHz, DMSO-d6) δ 159.89 (d, J = 253.0 Hz), 150.38, 142.12, 138.61, 133.80 (d, J = 8.5 Hz), 133.07, 132.46 – 131.89 (m), 124.88 (d, J = 3.4 Hz), 122.84 (q, J = 273.1 Hz), 119.28 (q, J = 33.4 Hz), 116.48 (d, J = 21.0 Hz), 115.44 (d, J = 13.8 Hz), 115.15 (d, J = 5.2 Hz), 110.43. **^{19}F NMR:** (376 MHz, DMSO-d6) δ -62.27, -113.27 (dt, J = 11.3, 6.2 Hz). **HRMS (ESI):** calculated for $C_{14}H_8F_4N_3O_3$ [M+H]+ 342.0496, found 342.0521.

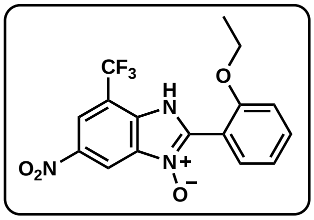

2-(2-Ethoxyphenyl)-5-nitro-7-trifluoromethyl-1*H*-benzimidazole 3-oxide (4cd). Synthesized following the procedure for **4aa**, starting from **1c** (0.3 mmol, 1 eq) and amine **2d** (0.6 mmol, 2 eq). Product **4cd** was obtained as a yellow powder (109 mg, 99%). **m.p.:** decomposition at 216 °C. **^{1}H NMR:** (400 MHz, DMSO-d6) δ 12.45 (s, 1H), 8.65 (d, J = 2.1 Hz, 1H), 8.36 (d, J = 2.0 Hz, 1H), 7.64 – 7.53 (m, 2H), 7.25 (d, J = 8.3 Hz, 1H), 7.13 (t, J = 7.5 Hz, 1H), 4.15 (q, J = 7.0 Hz, 2H), 1.25 (t, J = 7.0 Hz, 3H). **^{13}C NMR:** 13C NMR (101 MHz, DMSO-d6) δ 157.14, 153.57, 141.69, 138.67, 132.84, 132.74, 131.71, 122.90 (q, J = 272.9 Hz), 120.30, 118.82 (q, J = 33.4 Hz), 116.74, 114.70 (q, J = 5.3 Hz), 113.06, 110.03, 64.03, 14.31. **^{19}F NMR:** (376 MHz, DMSO-d6) δ -62.25. **HRMS (ESI):** calculated for $C_{16}H_{13}F_3N_3O_4$ [M+H]+ 368.0853, found 368.0890.

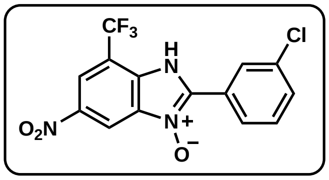

2-(3-Chlorophenyl)-5-nitro-7-trifluoromethyl-1*H*-benzimidazole 3-oxide (4ce). Synthesized following the procedure for **4aa**, starting from **1c** (0.3 mmol, 1 eq) and amine **2e** (0.6 mmol, 2 eq). Product **4ce** was obtained as a yellow powder (84 mg, 78%). **m.p.:** decomposition at 226 °C. **^{1}H NMR:** (400 MHz, DMSO-d6) δ 8.64 (d, J = 2.1 Hz, 1H), 8.35 (d, J = 2.0 Hz, 1H), 8.32 – 8.24 (m, 2H), 7.74 – 7.61 (m, 2H). **^{13}C NMR:** (101 MHz, DMSO-d6) δ 151.21, 141.95, 138.20, 134.00, 133.57, 131.34, 131.02, 128.98, 128.09, 127.48, 122.84 (q, J = 271.9 Hz), 118.90 (q, J = 33.4 Hz), 115.35 (d, J = 5.2 Hz), 110.38. **^{19}F NMR:**

(376 MHz, DMSO-d6) δ -62.18. **HRMS (ESI):** calculated for $C_{14}H_8ClF_3N_3O_3$ [M+H]+ 358.0201, found 358.0219.

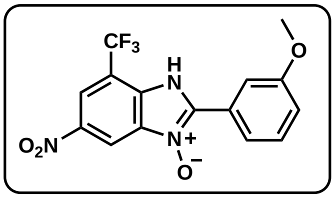

2-(3-Methoxyphenyl)-5-nitro-7-trifluoromethyl-1*H*-benzimidazole 3-oxide (4cf). Synthesized following the procedure for **4aa**, starting from **1c** (0.3 mmol, 1 eq) and amine **2f** (0.6 mmol, 2 eq). Product **4cf** was obtained as a yellow powder (90 mg, 84%). **m.p.:** decomposition at 201 °C. **¹H NMR:** (300 MHz, DMSO-d6) δ 13.11 (s, 1H), 8.66 (d, J = 2.1 Hz, 1H), 8.35 (d, J = 2.2 Hz, 1H), 7.96 – 7.80 (m, 2H), 7.56 (t, J = 8.0 Hz, 1H), 7.28 – 7.19 (m, 1H), 3.87 (s, 3H). **¹³C NMR:** (126 MHz, DMSO-d6) δ 159.59, 153.04, 142.05, 138.66, 134.30, 130.60, 128.43, 123.16 (q, J = 273.1 Hz), 121.61, 119.04 (q, J = 33.4 Hz), 117.48, 115.55 (q, J = 5.2 Hz), 114.46, 110.39, 55.69. **¹⁹F NMR:** (376 MHz, DMSO-d6) δ -62.25. **HRMS (ESI):** calculated for $C_{15}H_{11}F_3N_3O_4$ [M+H]+ 354.0696, found 354.0722.

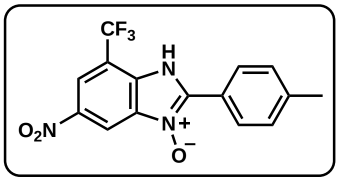

5-Nitro-2-(*p*-tolyl)-7-trifluoromethyl-1*H*-benzimidazole 3-oxide (4cg). Synthesized following the procedure for **4aa**, starting from **1c** (0.3 mmol, 1 eq) and amine **2g** (0.6 mmol, 2 eq). Product **4cg** was obtained as a yellow powder (93 mg, 92%). **m.p.:** decomposition at 271 °C. **¹H NMR:** (300 MHz, DMSO-d6) δ 13.04 (s, 1H), 8.60 (d, J = 2.2 Hz, 1H), 8.32 (d, J = 2.1 Hz, 1H), 8.25 (d, J = 8.4 Hz, 2H), 7.43 (d, J = 8.2 Hz, 2H), 2.42 (s, 3H). **¹³C NMR:** (101 MHz, DMSO-d6) δ 153.06, 141.83, 141.53, 138.61, 134.04, 129.55, 128.85, 124.27, 122.94 (dd, J = 6109.5, 271.2 Hz), 118.49 (q, J = 33.3 Hz), 115.12 (d, J = 5.3 Hz), 109.86, 21.16. **¹⁹F NMR:** (376 MHz, DMSO-d6) δ -62.18. **HRMS (ESI):** calculated for $C_{15}H_{11}F_3N_3O_3$ [M+H]+ 338.0747, found 338.0754.

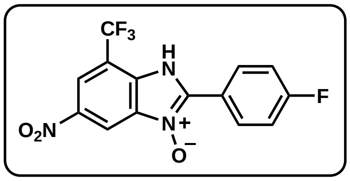

2-(4-Fluorophenyl)-5-nitro-7-trifluoromethyl-1*H*-benzimidazole 3-oxide (4ch). Synthesized following the procedure for **4aa**, starting from **1c** (0.3 mmol, 1 eq) and amine **2h** (0.6 mmol, 2 eq). Product **4ch** was obtained as a pale yellow powder (69 mg, 67%). **m.p.:** decomposition at 223 °C. **¹H NMR:** (300 MHz, DMSO-d6) δ 13.13 (s, 1H), 8.68 (s, 1H), 8.41 (dd, J = 8.8, 5.6 Hz, 2H), 8.36 (d, J = 2.1 Hz, 1H), 7.49 (t, J = 8.8 Hz, 2H). **¹³C NMR:** (126 MHz, DMSO-d6) δ 163.92 (d, J = 250.7 Hz), 151.95, 141.76 (d, J = 4.7 Hz), 138.39, 133.92, 131.54 (d, J = 9.0 Hz), 123.60, 122.86 (q, J = 272.9 Hz), 118.69 (q, J = 33.4 Hz), 116.20 (d, J = 21.8 Hz), 115.24, 110.10 (d, J = 4.6 Hz). **¹⁹F NMR:** (376 MHz, DMSO-d6) δ -62.19,

-110.07 (tt, J = 9.0, 5.5 Hz). **HRMS (ESI):** calculated for $C_{14}H_8F_4N_3O_3$ [M+H]+ 342.0496, found 342.0509.

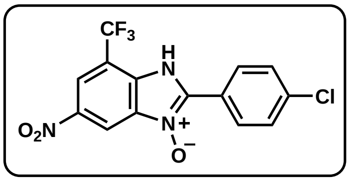

2-(4-Chlorophenyl)-5-nitro-7-trifluoromethyl-1*H*-benzimidazole 3-oxide (4ci). Synthesized following the procedure for **4aa**, starting from **1c** (0.3 mmol, 1 eq) and amine **2h** (0.6 mmol, 2 eq). Product **4ch** was obtained as a pale yellow powder (106 mg, 99%). **m.p.:** decomposition at 256 °C. **^{1}H NMR:** (400 MHz, DMSO-d6) δ 13.19 (s, 1H), 8.68 (d, J = 2.2 Hz, 1H), 8.42 – 8.33 (m, 3H), 7.76 – 7.68 (m, 2H). **^{13}C NMR:** (101 MHz, DMSO-d6) δ 151.75, 141.93, 138.35, 136.50, 134.01, 130.61, 129.21, 125.88, 122.87 (q, J = 273.1 Hz), 118.81 (q, J = 34.1 Hz), 115.38 (d, J = 5.3 Hz), 110.29. **^{19}F NMR:** (376 MHz, DMSO-d6) δ -62.16. **HRMS (ESI):** calculated for $C_{14}H_8ClF_3N_3O_3$ [M+H]+ 358.0201, found 358.0209.

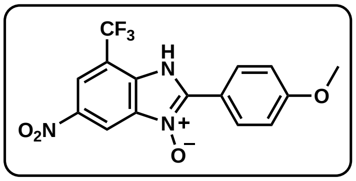

2-(4-Methoxyphenyl)-5-nitro-7-trifluoromethyl-1*H*-benzimidazole 3-oxide (4cj). Synthesized following the procedure for **4aa**, starting from **1c** (0.3 mmol, 1 eq) and amine **2j** (0.6 mmol, 2 eq). Product **4cj** was obtained as a yellow powder (85 mg, 80%). **m.p.:** decomposition at 261 °C. **^{1}H NMR:** (400 MHz, DMSO-d6) δ 13.05 (s, 1H), 8.60 – 8.55 (m, 1H), 8.38 – 8.29 (m, 3H), 7.22 – 7.14 (m, 2H), 3.87 (s, 3H). **^{13}C NMR:** (101 MHz, DMSO-d6) δ 161.86, 152.97, 141.28, 138.74, 134.07, 130.71, 122.94 (q, J = 273.2 Hz), 119.29, 118.12 (q, J = 33.4 Hz), 115.04 (d, J = 5.3 Hz), 114.45, 109.58, 55.48. **^{19}F NMR:** (376 MHz, DMSO-d6) δ -62.14. **HRMS (ESI):** calculated for $C_{15}H_{11}F_3N_3O_4$ [M+H]+ 354.0696, found 354.0703.

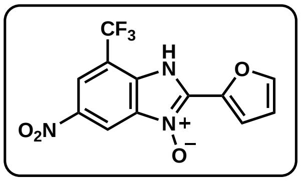

2-(Furan-2-yl)-5-nitro-7-trifluoromethyl-1*H*-benzimidazole 3-oxide (4ck). Synthesized following the procedure for **4aa**, starting from **1c** (0.3 mmol, 1 eq) and amine **2k** (0.6 mmol, 2 eq). Product **4ck** was obtained as a yellow powder (88 mg, 94%). **m.p.:** decomposition at 263 °C. **^{1}H NMR:** (300 MHz, DMSO-d6) δ 13.13 (s, 1H), 8.61 (d, J = 2.1 Hz, 1H), 8.33 (d, J = 2.2 Hz, 1H), 8.13 (d, J = 1.6 Hz, 1H), 7.63 (d, J = 3.5 Hz, 1H), 6.86 (dd, J = 3.6, 1.8 Hz, 1H). **^{13}C NMR:** (101 MHz, DMSO-d6) δ 147.07, 145.57, 141.80, 141.33, 139.04, 133.31, 122.86 (q, J = 273.2 Hz), 118.61 (q, J = 33.1 Hz), 116.79, 115.68 – 114.95 (m),

112.87, 109.71. **¹⁹F NMR:** (376 MHz, DMSO-d6) δ -62.25. **HRMS (ESI):** calculated for $C_{12}H_7F_3N_3O_4$ [M+H]+ 314.0383, found 314.0394.

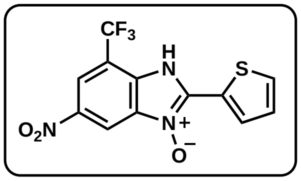

5-Nitro-2-(thiophen-2-yl)-7-trifluoromethyl-1*H*-benzimidazole 3-oxide (4cl). Synthesized following the procedure for **4aa**, starting from **1c** (0.3 mmol, 1 eq) and amine **2l** (0.6 mmol, 2 eq). Product **4cl** was obtained as a pale yellow powder (83 mg, 84%). **m.p.:** decomposition at 237 °C. **^{1}H NMR:** (300 MHz, DMSO-d6) δ 13.30 (s, 2H), 8.60 (d, J = 2.1 Hz, 2H), 8.34 (d, J = 2.1 Hz, 2H), 8.24 (dd, J = 3.8, 1.2 Hz, 2H), 8.03 (dd, J = 5.0, 1.2 Hz, 2H), 7.36 (dd, J = 5.1, 3.8 Hz, 2H). **^{13}C NMR:** (126 MHz, DMSO-d6) δ 149.21, 141.52, 139.11, 133.57, 132.70, 131.60, 128.76, 128.33, 122.90 (q, J = 273.0 Hz), 118.19 (q, J = 33.4 Hz), 115.40 (d, J = 5.4 Hz), 109.54. **^{19}F NMR:** 19F NMR (376 MHz, DMSO-d6) δ -62.17. **HRMS (ESI):** calculated for $C_{12}H_7F_3N_3O_3S$ [M+H]+ 330.0155, found 330.0169.

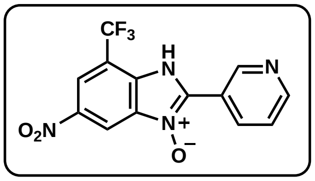

5-Nitro-2-(pyridin-3-yl)-7-trifluoromethyl-1*H*-benzimidazole 3-oxide (4cm). Synthesized following the procedure for **4aa**, starting from **1c** (0.3 mmol, 1 eq) and amine **2m** (0.6 mmol, 2 eq). Product **4cm** was obtained as a yellow powder (82 mg, 84%). **m.p.:** decomposition at 289 °C. **^{1}H NMR:** (300 MHz, DMSO-d6) δ 13.13 (s, 1H), 9.45 (d, J = 2.1 Hz, 1H), 8.81 (dd, J = 4.9, 1.7 Hz, 1H), 8.71 (d, J = 2.2 Hz, 1H), 8.64 (dt, J = 8.1, 2.0 Hz, 1H), 8.37 (d, J = 2.1 Hz, 1H), 7.68 (dd, J = 8.0, 4.8 Hz, 1H). **^{13}C NMR:** (101 MHz, DMSO-d6) δ 151.91, 150.84, 149.14, 142.06, 138.43, 136.33, 133.81, 124.06, 122.81 (q, J = 272.9 Hz), 119.01 (q, J = 33.5 Hz), 115.76 – 115.07 (m), 110.37. **^{19}F NMR:** (376 MHz, DMSO-d6) δ -62.17. **HRMS (ESI):** calculated for $C_{13}H_8F_3N_4O_3$ [M+H]+ 325.0543, found 325.0554.

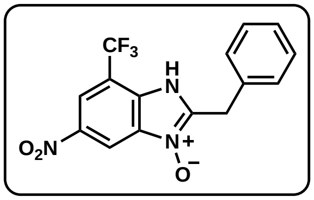

2-Benzyl-5-nitro-7-trifluoromethyl-1*H*-benzimidazole 3-oxide (4cn). Synthesized following the procedure for **4aa**, starting from **1c** (0.3 mmol, 1 eq) and amine **2m** (0.6 mmol, 2 eq). Product **4cm** was obtained as a yellow powder (78 mg, 77%). **m.p.:** decomposition at 174 °C. **^{1}H NMR:** (300 MHz, DMSO-d6) δ 12.83 (s, 1H), 8.58 (d, J = 2.3 Hz, 1H), 8.31 (d, J = 2.1 Hz, 1H), 7.43 – 7.19 (m, 5H), 4.39 (s, 2H). **^{13}C NMR:** (126 MHz, DMSO-d6) δ 156.79, 141.53, 138.53, 135.66, 132.59, 128.87, 128.67, 126.92, 122.88 (q, J = 273.1 Hz), 118.54 (q, J = 33.3 Hz), 114.57 (d, J = 5.3 Hz), 109.72, 31.84. **^{19}F NMR:** (376

MHz, DMSO-d6) δ -62.17. **HRMS (ESI):** calculated for $C_{15}H_{11}F_3N_3O_3$ [M+H]+ 338.0747, found 338.0772.

Scaled-up reaction of *7-nitro-2-phenyl-1H-benzimidazole 3-oxide* (**4aa**)

Procedure was similar than previous reaction but with different amounts of reactants: 2-Chloro-1,3-dinitrobenzene (**1a**, 1.5 mmol, 1 eq), benzylamine (**2a**, 3 mmol, 2 eq) and ethanol (3 mL). For the second step, 2.5 M aqueos potassium carbonate (2ML) was added. Reaction times and temperatures were the same than previous protocol. The product mixture was acidified with 2M hydrochloric acid to a pH of between 2 and 5. Immediately after the first drop of HCl, precipitate started to form. The stir bar was removed and the reaction vessel placed into an ice bath for 1 h to promote further product precipitation. After this time, the solid product was removed by filtration and dried at 80 °C to constant weight. Analytically pure **4aa** was obtained as a yellow powder (366 mg, 95%); characterized by ^{1}H-NMR.

^{1}H-NMR (300 MHz $(CD_3)_2SO$). **7-nitro-2-phenyl-1*H*-benzimidazole 3-oxide (4aa)**

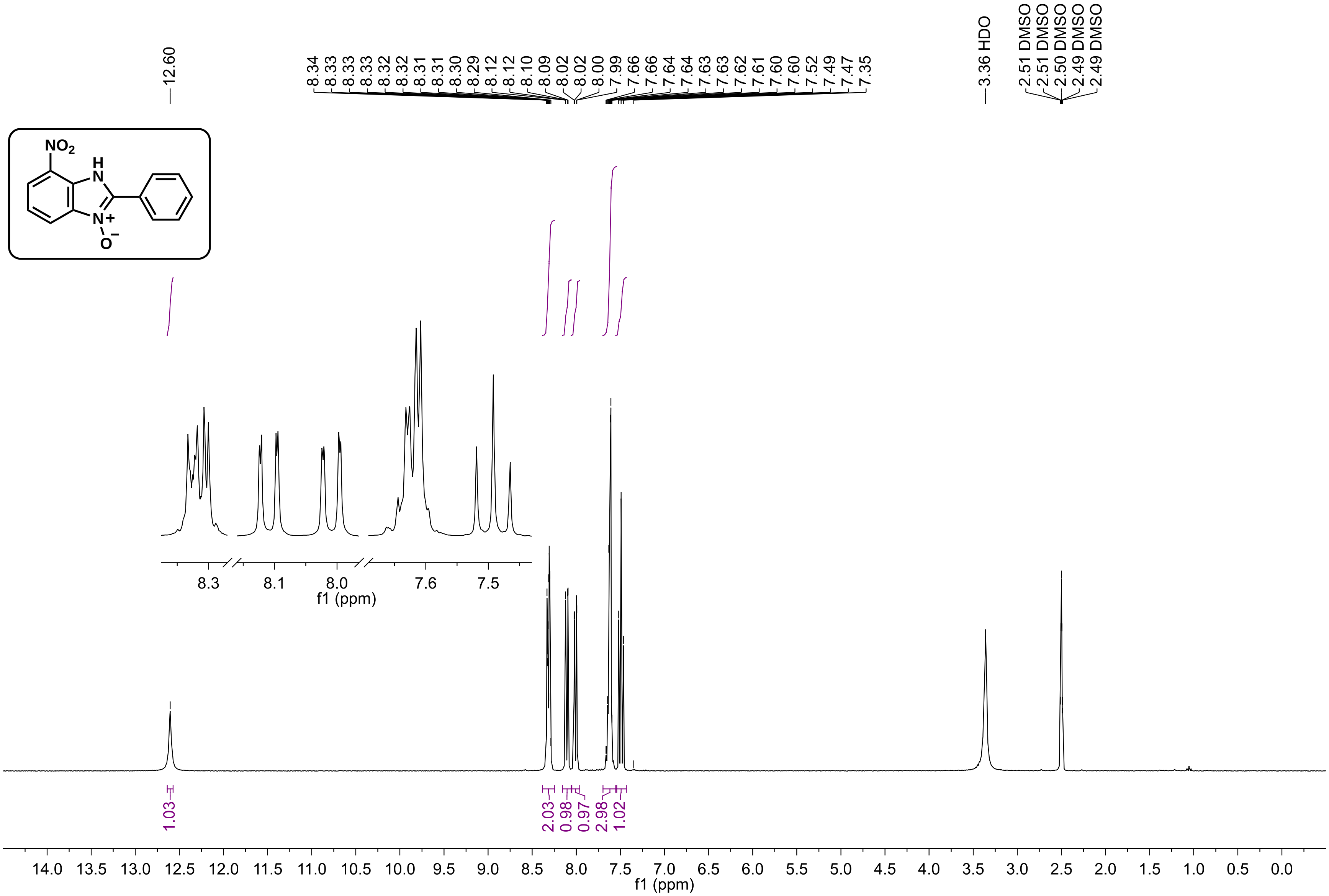

^{1}H-NMR (400 MHz $(CD_3)_2SO$). **7-nitro-2-(*o*-tolyl)-1*H*-benzimidazole 3-oxide (4ab)**

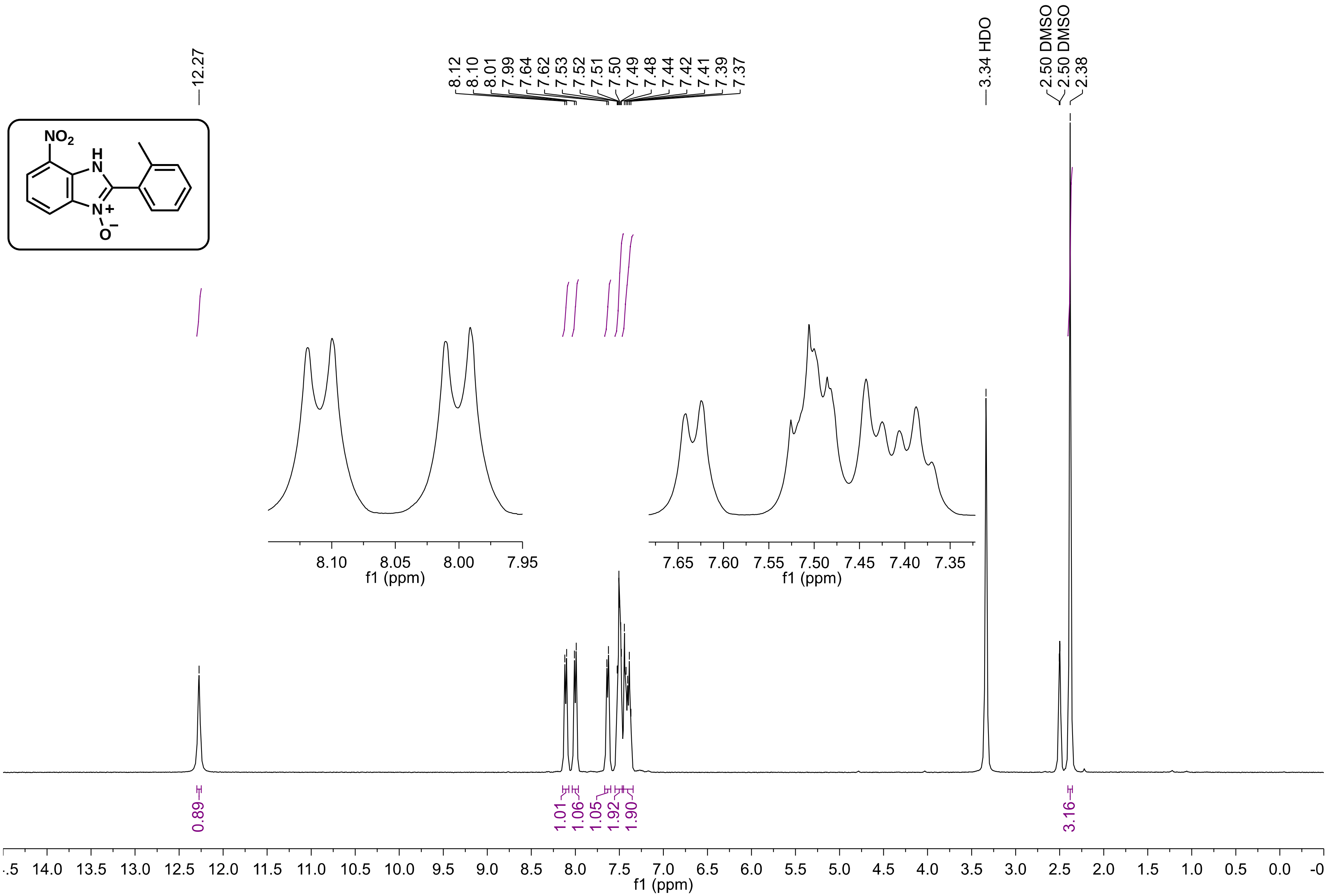

^{1}H-NMR (300 MHz $(CD_3)_2SO$). **2-(2-fluorophenyl)-7-nitro-1*H*-benzimidazole 3-oxide (4ac)**

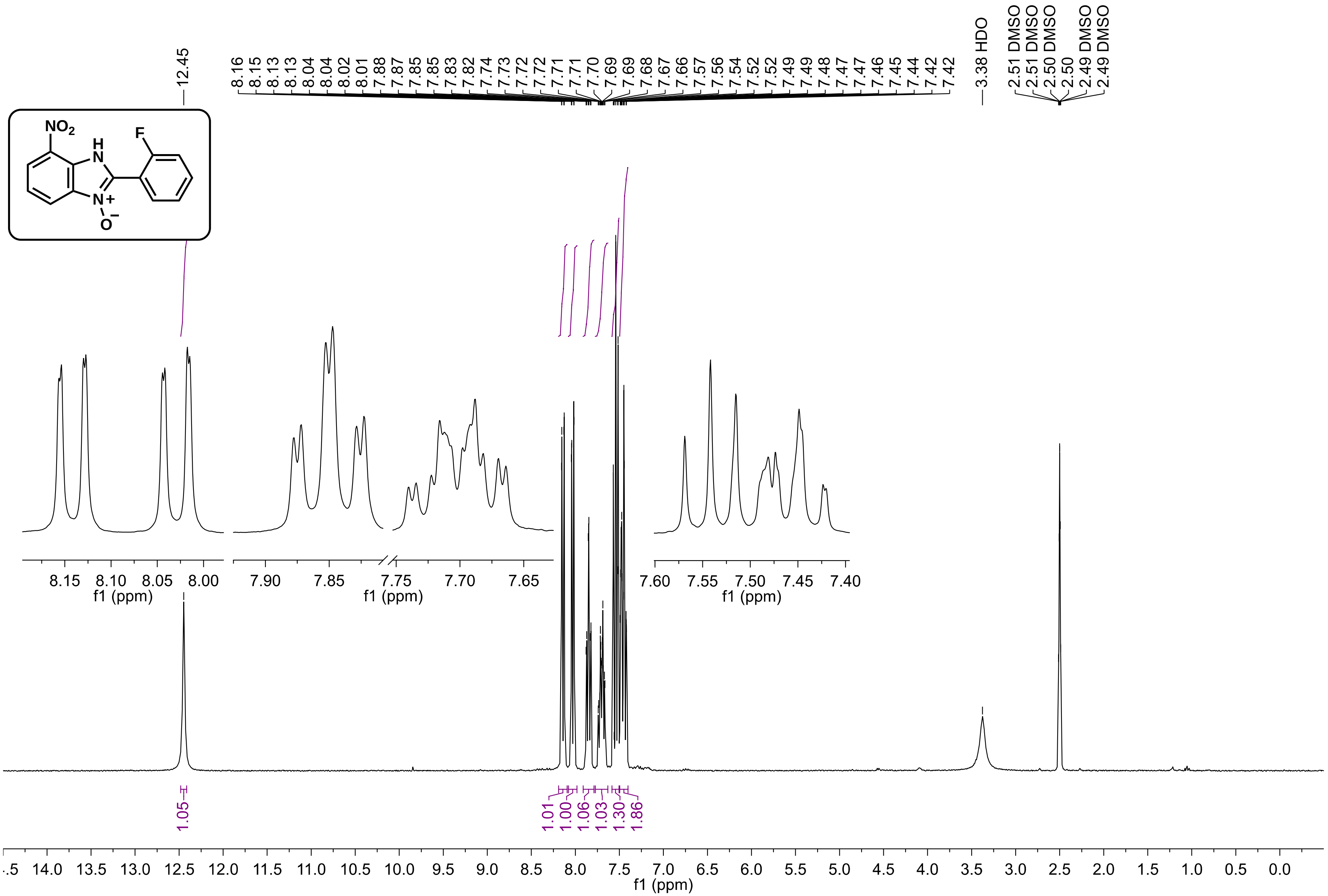

^{1}H-NMR (300 MHz $(CD_3)_2SO$). **2-(2-ethoxyphenyl)-7-nitro-1*H*-benzimidazole 3-oxide (4ad)**

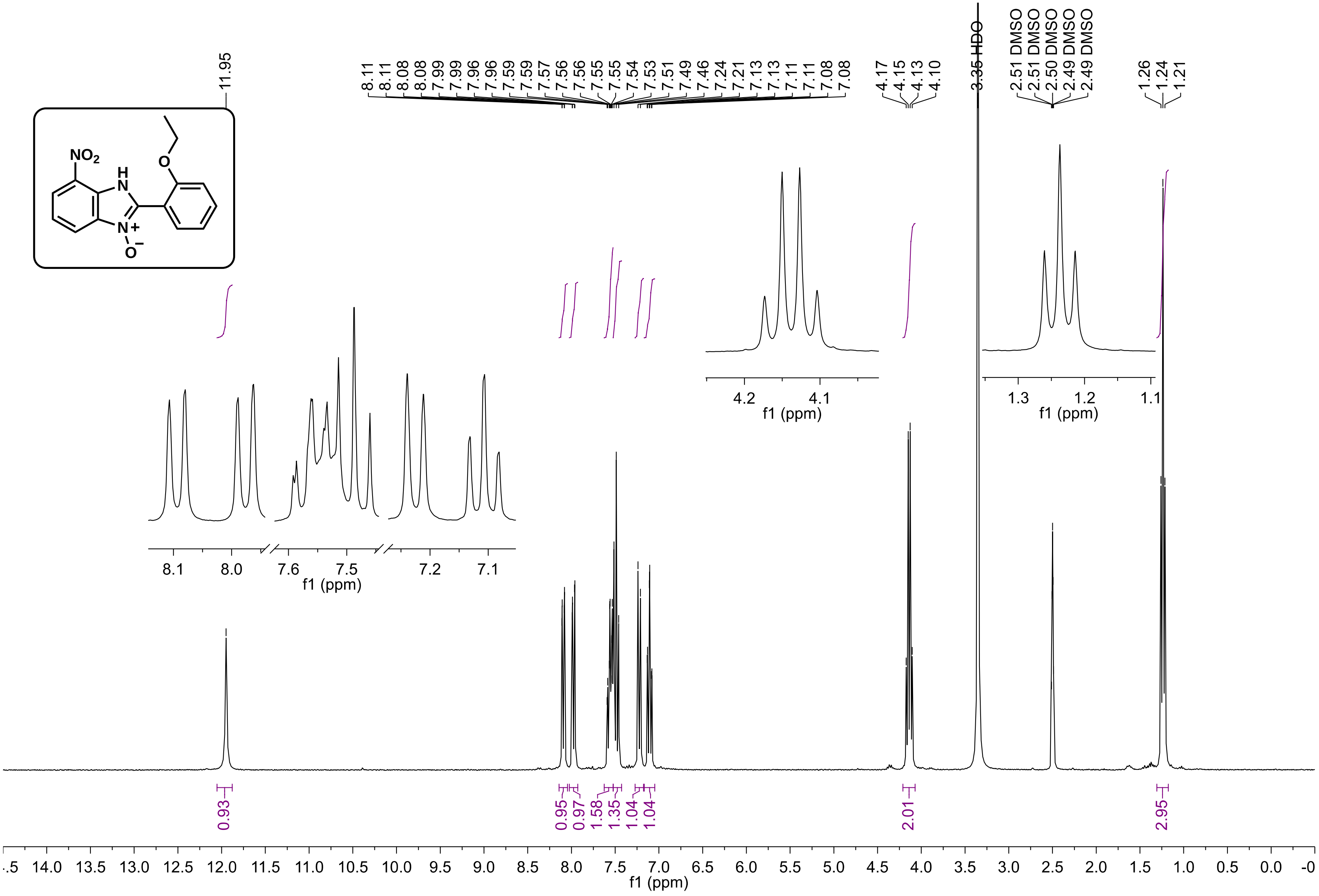

^{1}H-NMR (300 MHz $(CD_3)_2SO$). **2-(3-chlorophenyl)-7-nitro -1*H*-benzimidazole 3-oxide (4ae)**

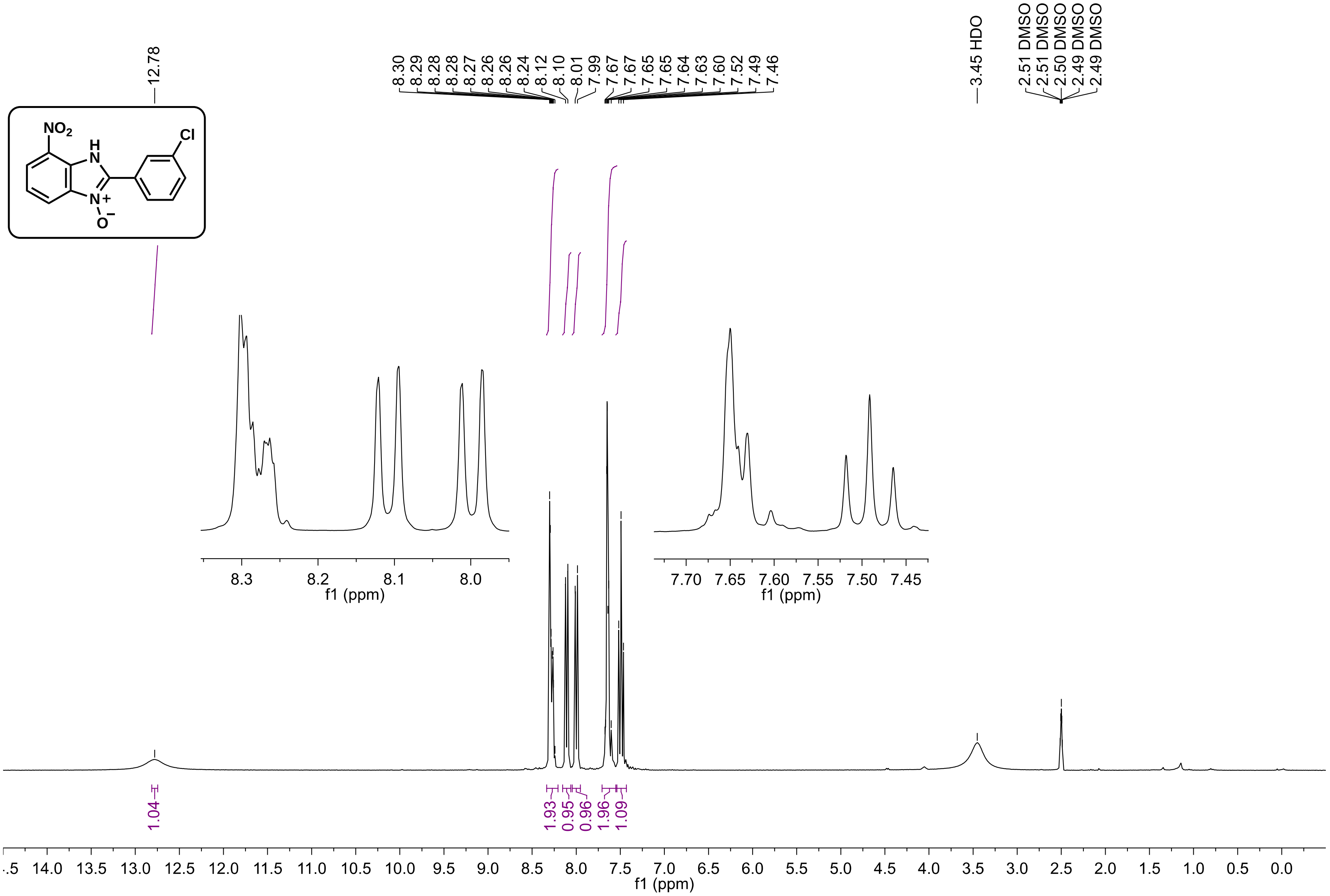

^{1}H-NMR (300 MHz $(CD_3)_2SO$). **2-(3-methoxyphenyl)-7-nitro-1*H*-benzimidazole 3-oxide (4af)**

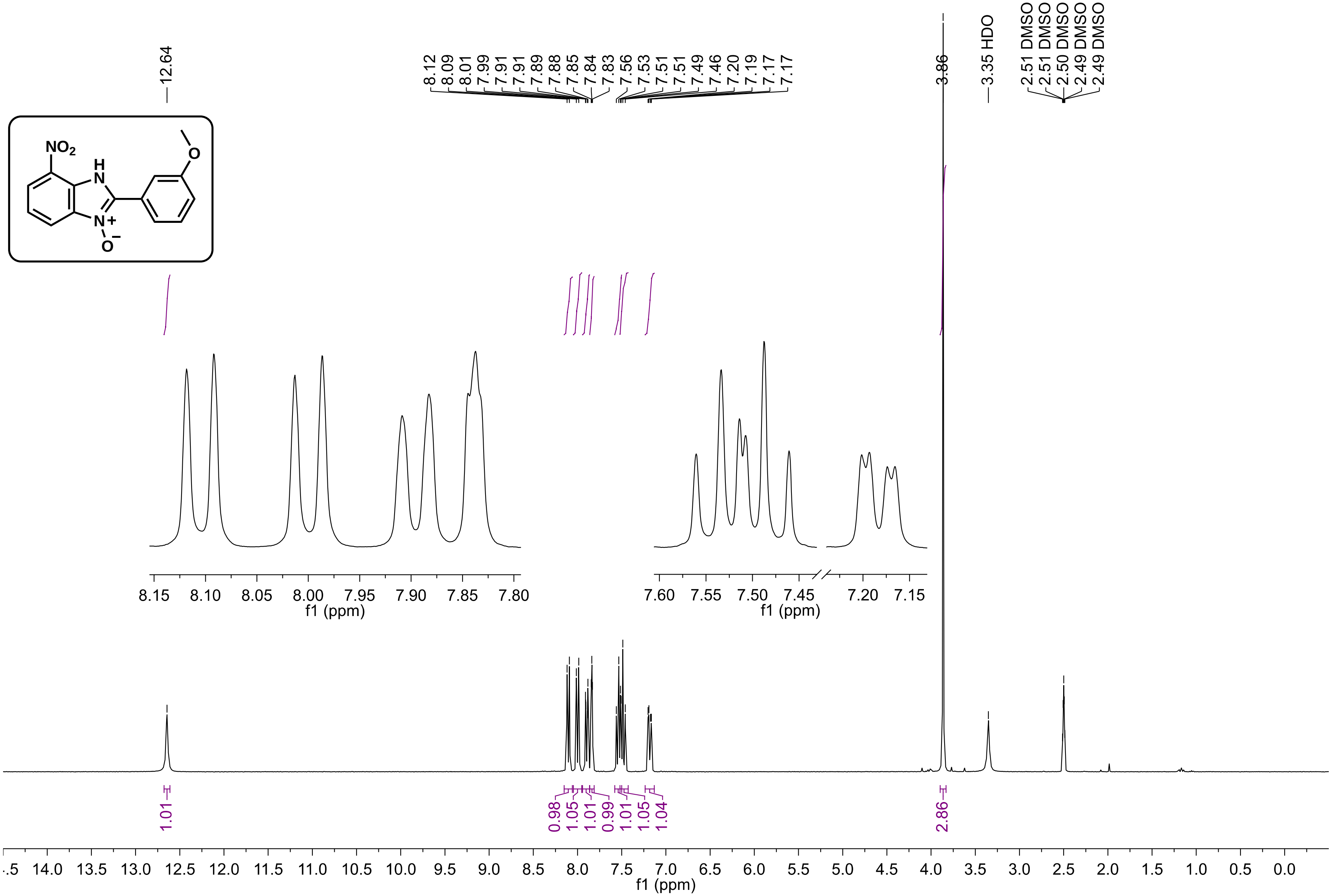

^{1}H-NMR (300 MHz $(CD_3)_2SO$). **7-nitro-2-(*p*-tolyl)-1*H*-benzimidazole 3-oxide (4ag)**

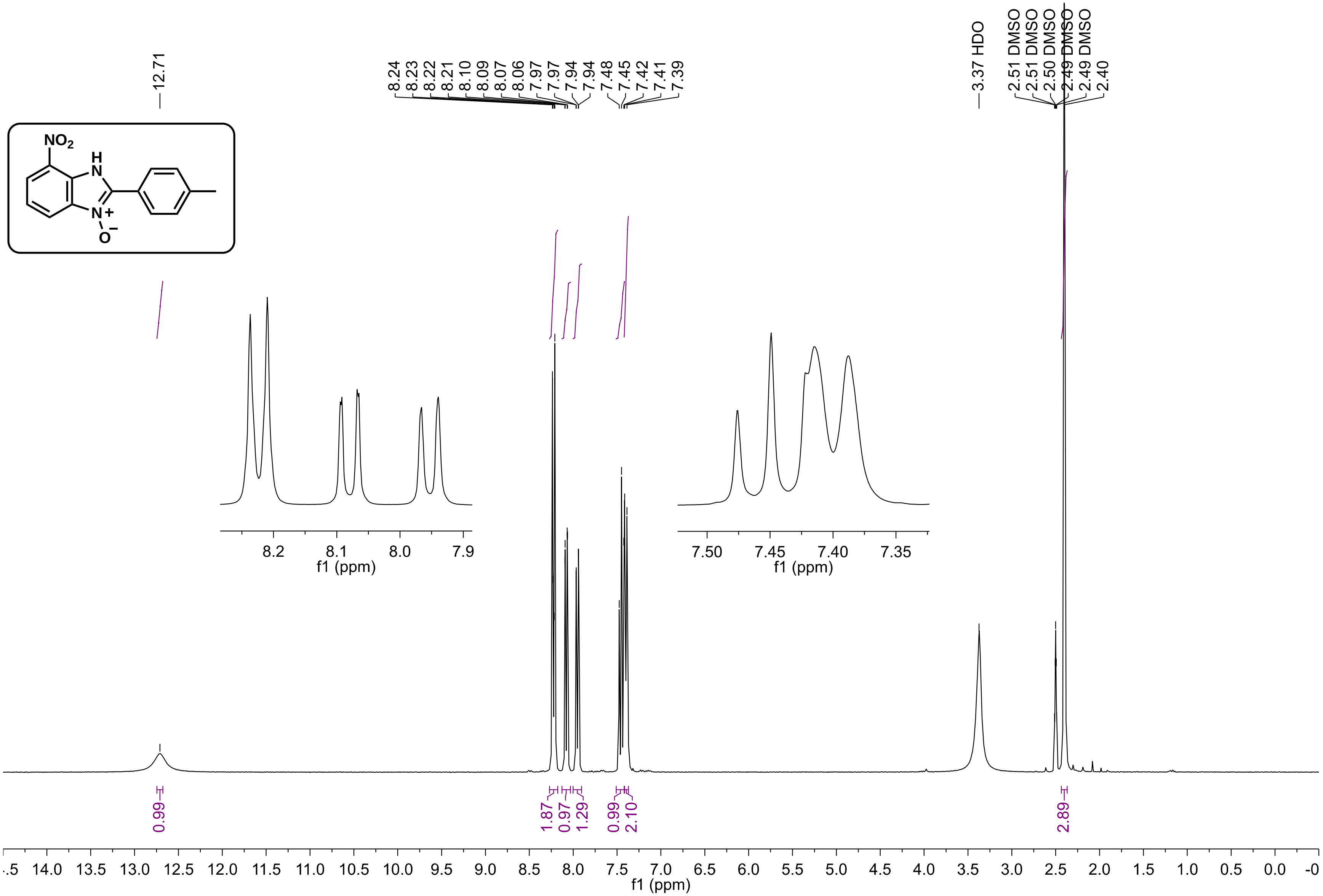

^{1}H-NMR (400 MHz $(CD_3)_2SO$). **2-(4-fluorophenyl)-7-nitro-1*H*-benzimidazole 3-oxide (4ah)**

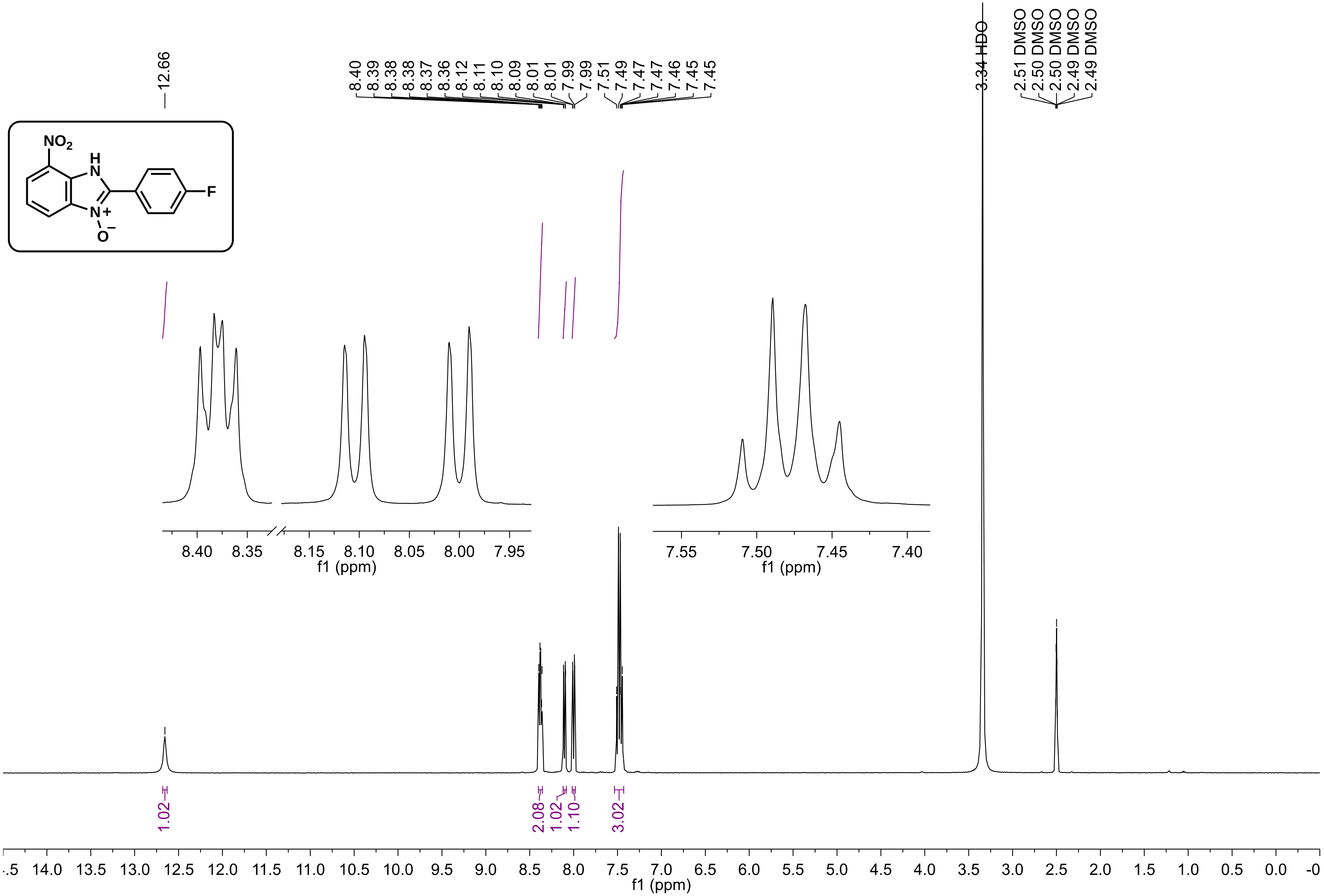

^{1}H-NMR (300 MHz $(CD_3)_2SO$). **2-(4-chlorophenyl)-7-nitro-1*H*-benzimidazole 3-oxide (4ai)**

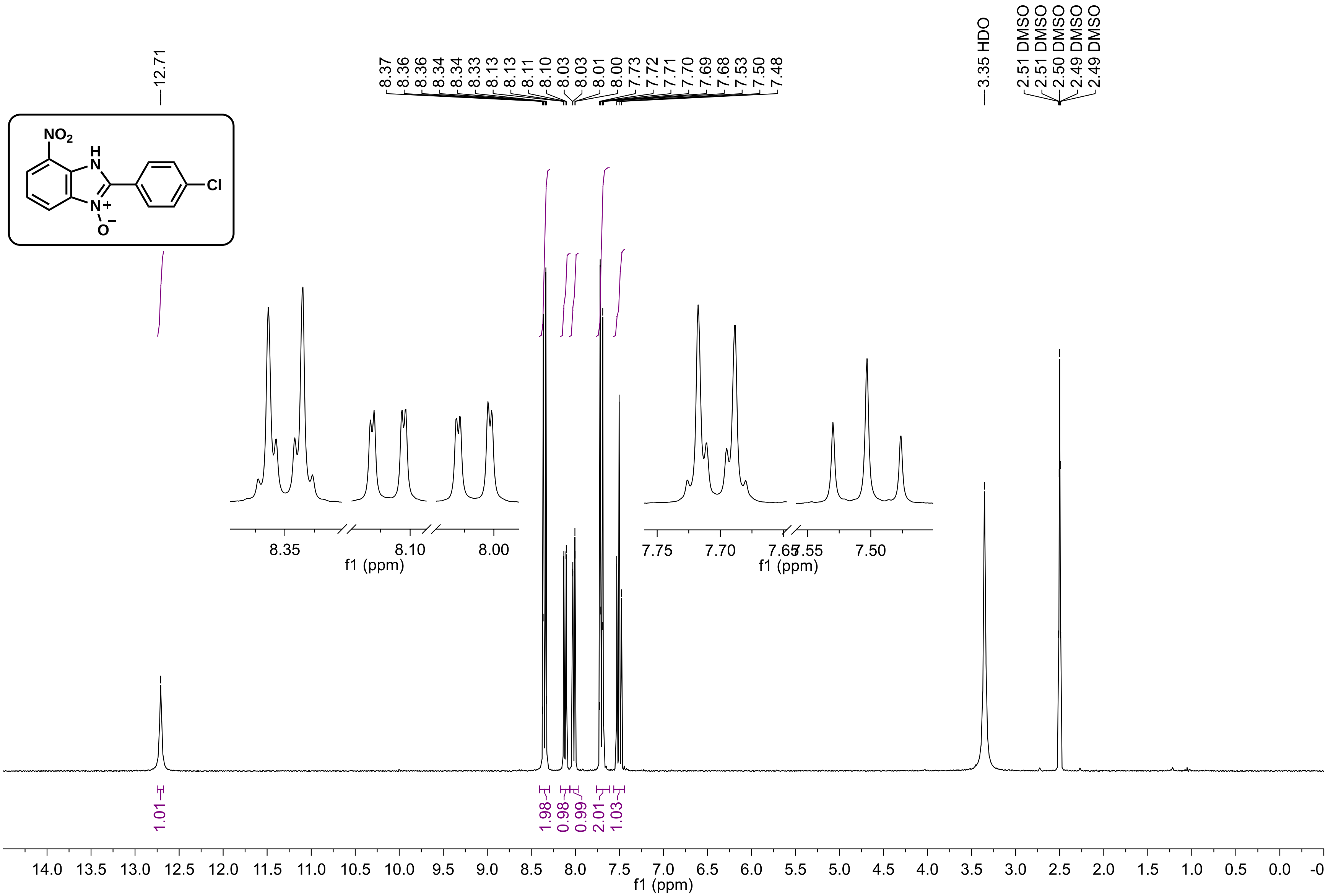

^{1}H-NMR (300 MHz $(CD_3)_2SO$). **2-(4-methoxyphenyl)-7-nitro-1*H*-benzimidazole 3-oxide (4aj)**

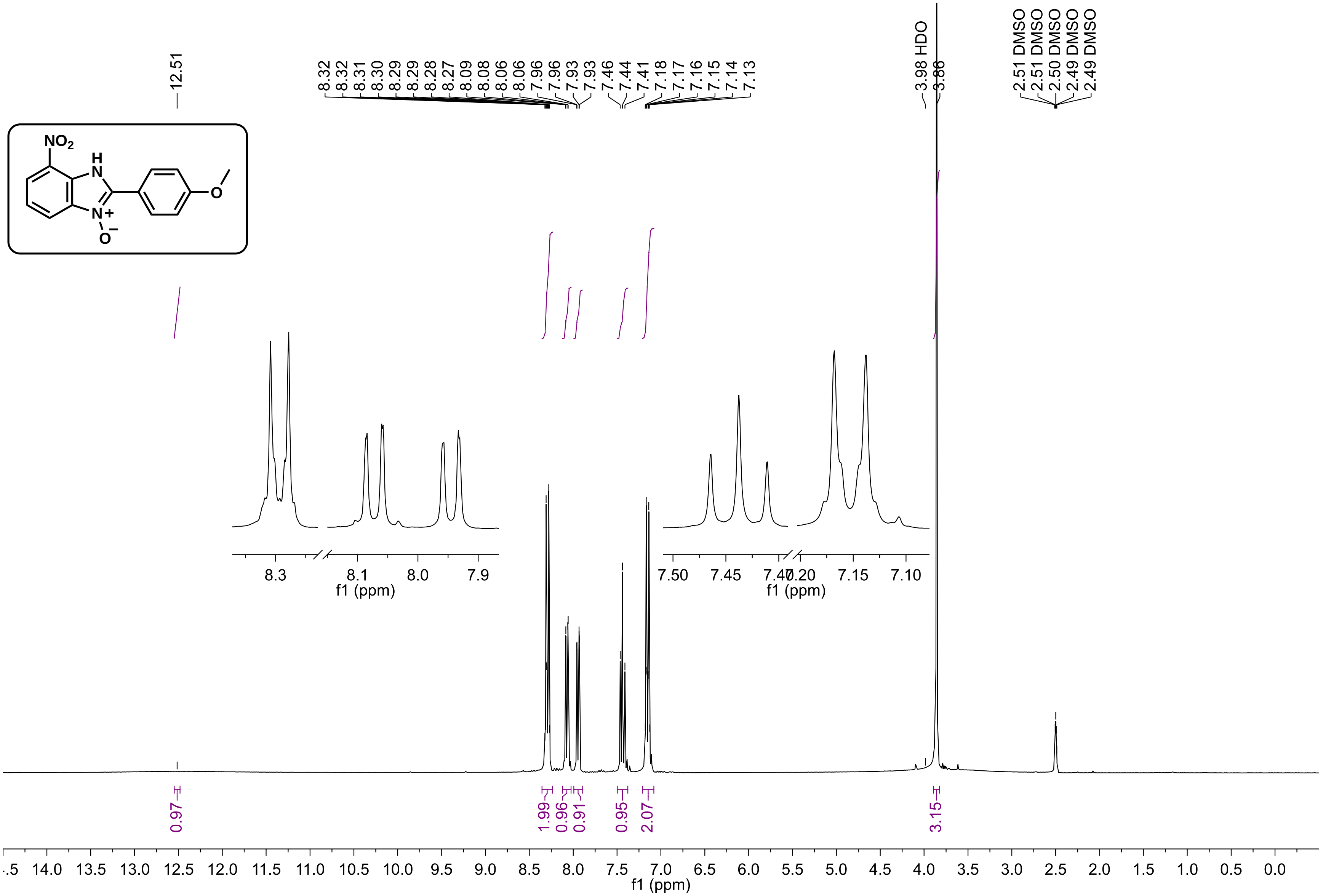

^{1}H-NMR (300 MHz $(CD_3)_2SO$). **2-(furan-2-yl)-7-nitro-1*H*-benzimidazole 3-oxide (4ak)**

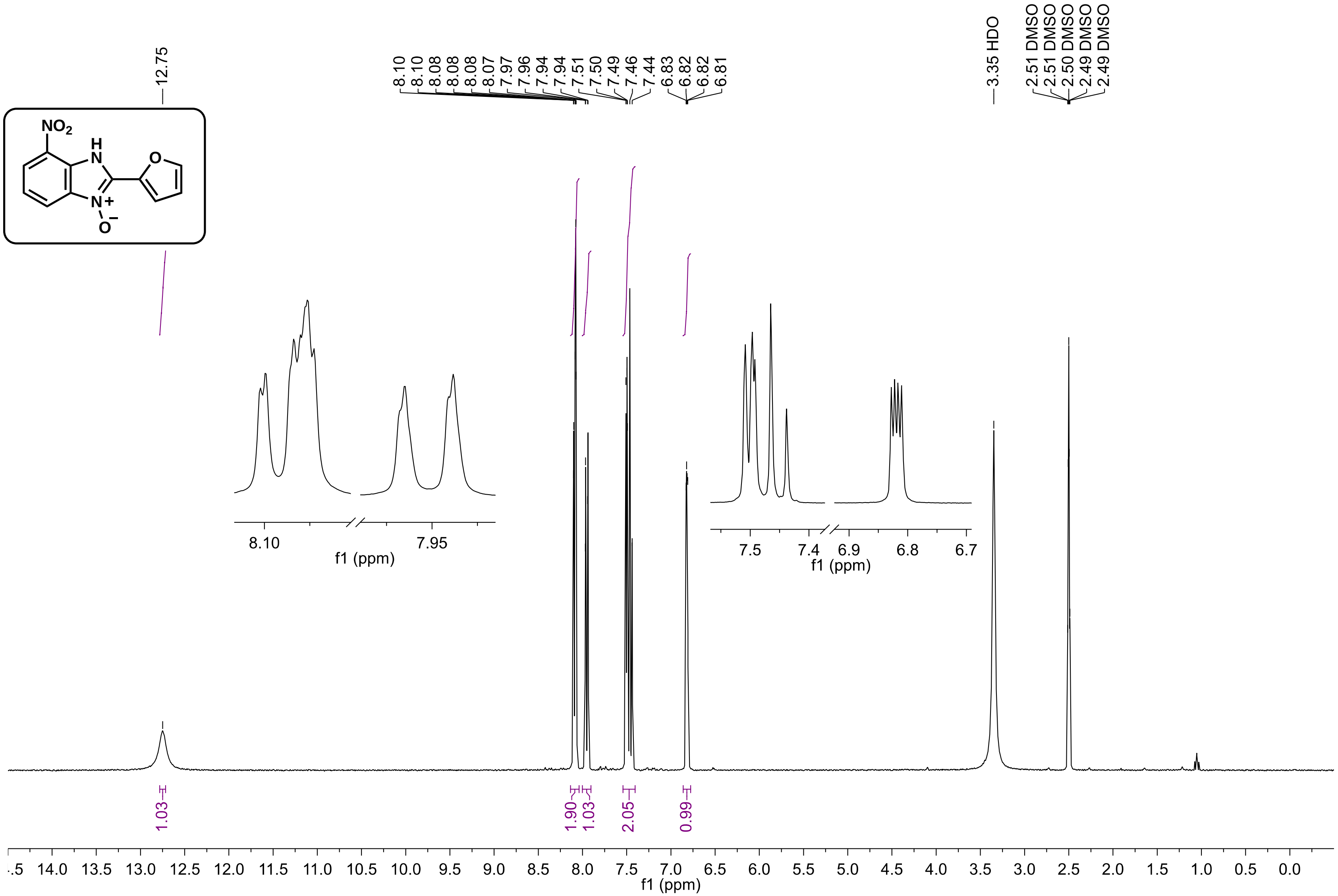

^{1}H-NMR (400 MHz $(CD_3)_2SO$). **7-nitro-2-(thiophen-2-yl)-1H-benzimidazole 3-oxide (4al)**

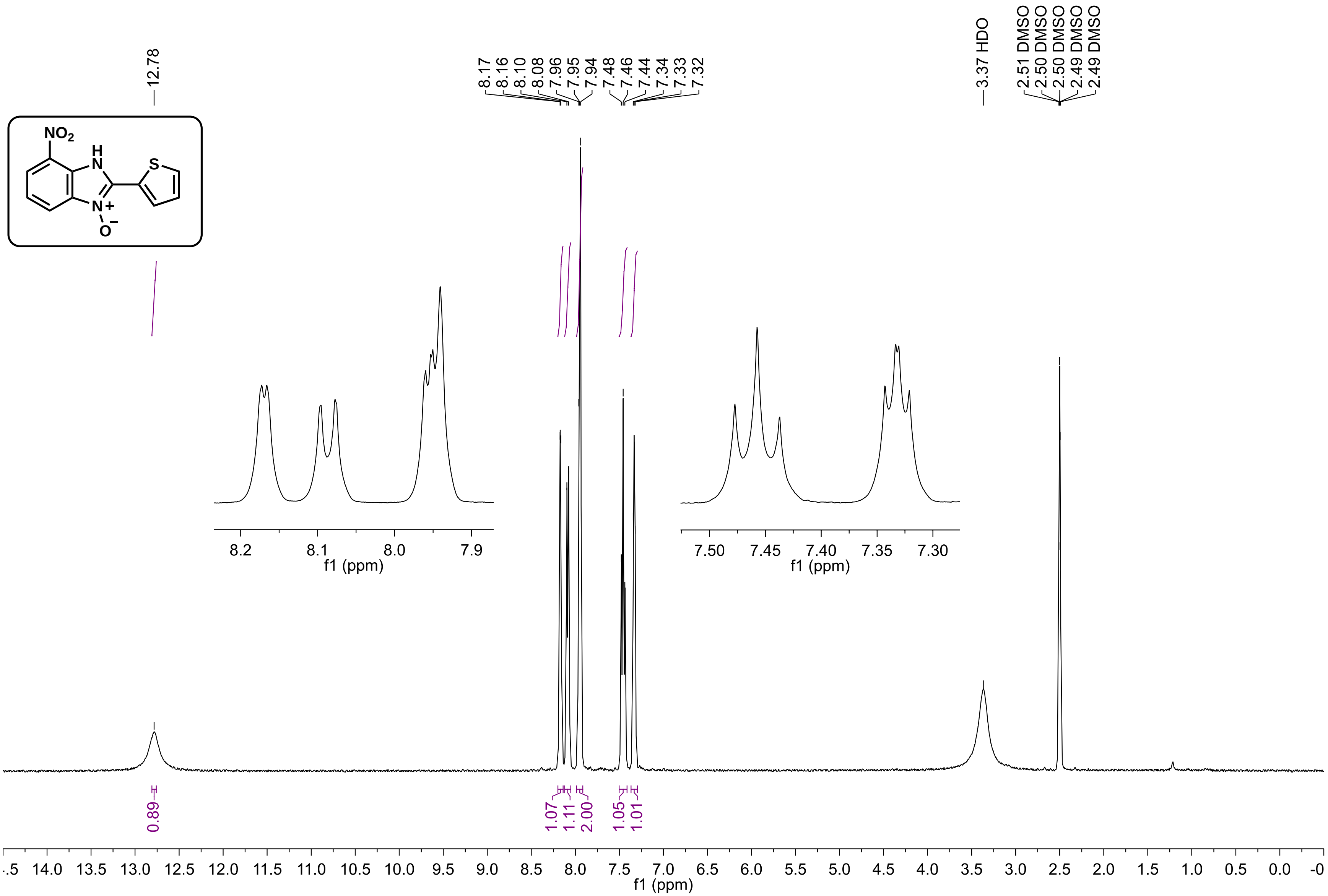

^{1}H-NMR (400 MHz $(CD_3)_2SO$). **7-nitro-2-(pyridin-3-yl)-1H-benzimidazole 3-oxide (4am)**

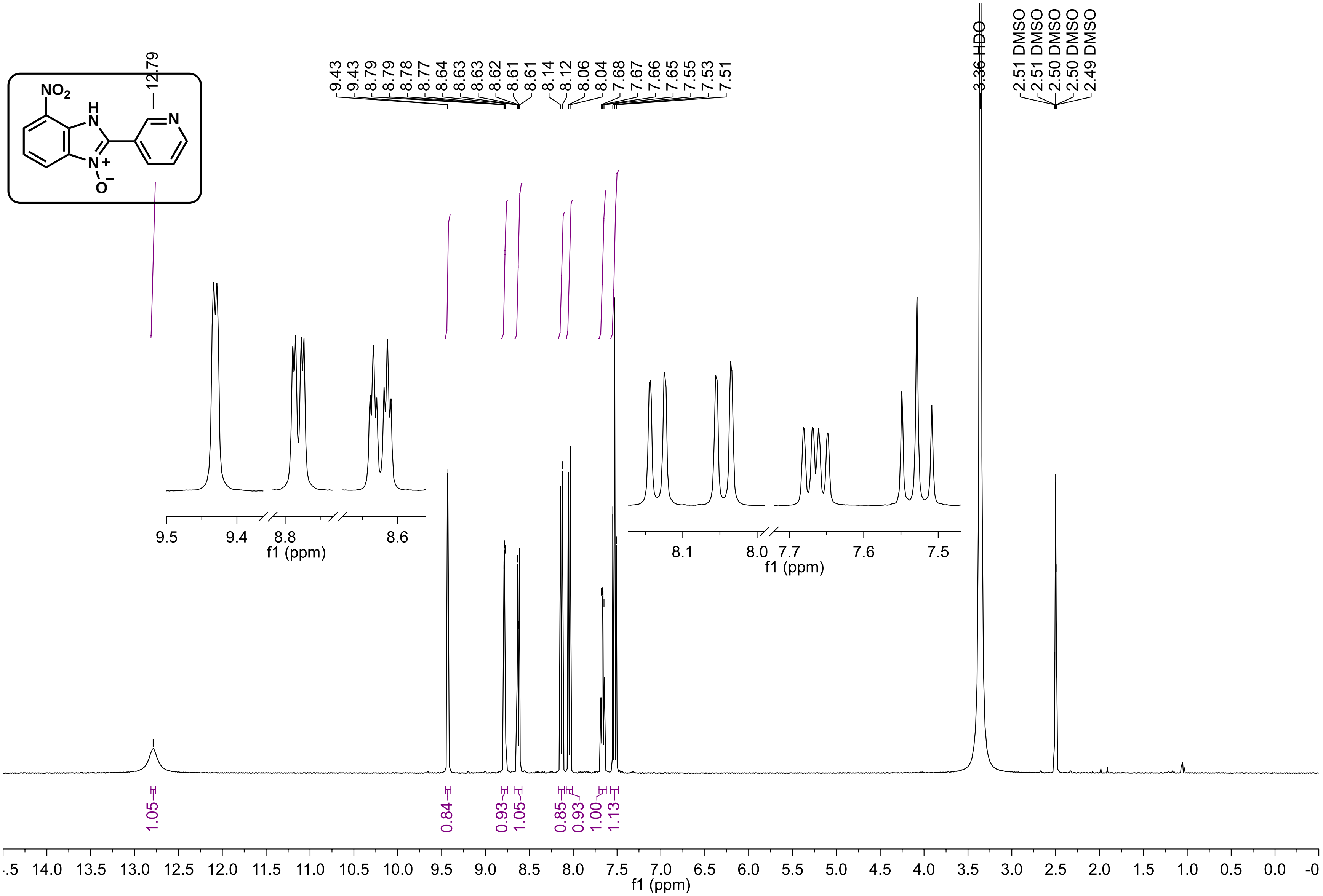

^{1}H-NMR (400 MHz $(CD_3)_2SO$). **2-benzyl-7-nitro-1H-benzimidazole 3-oxide (4an)**

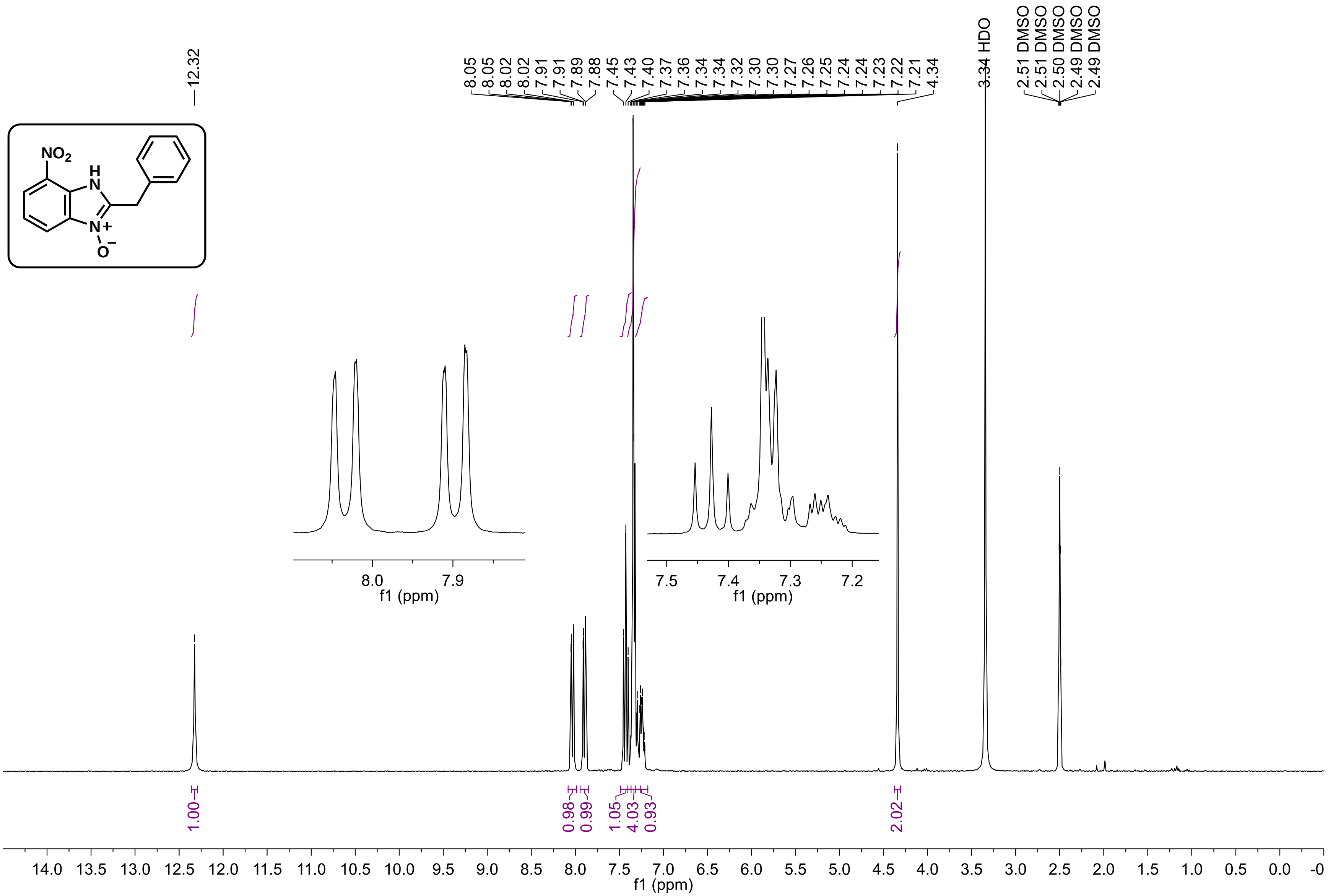

^{1}H-NMR (300 MHz $(CD_3)_2SO$). **7-nitro-2-phenyl-5-trifluoromethyl-1*H*-benzimidazole 3-oxide (4ba)**

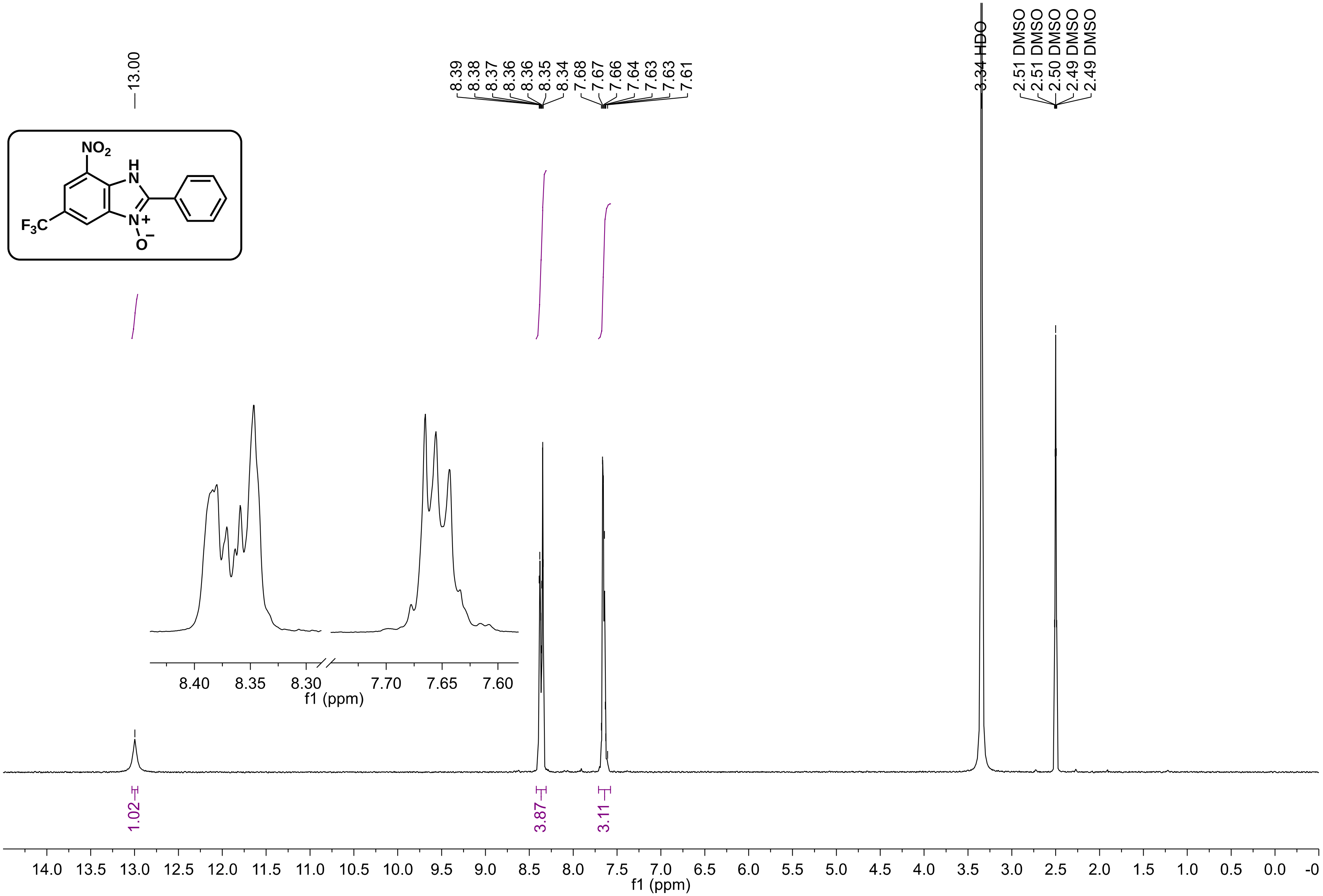

^{1}H-NMR (500 MHz $(CD_3)_2SO$). **7-nitro-2-(*o*-tolyl)-5-trifluoromethyl-1*H*-benzimidazole 3-oxide (4bb)**

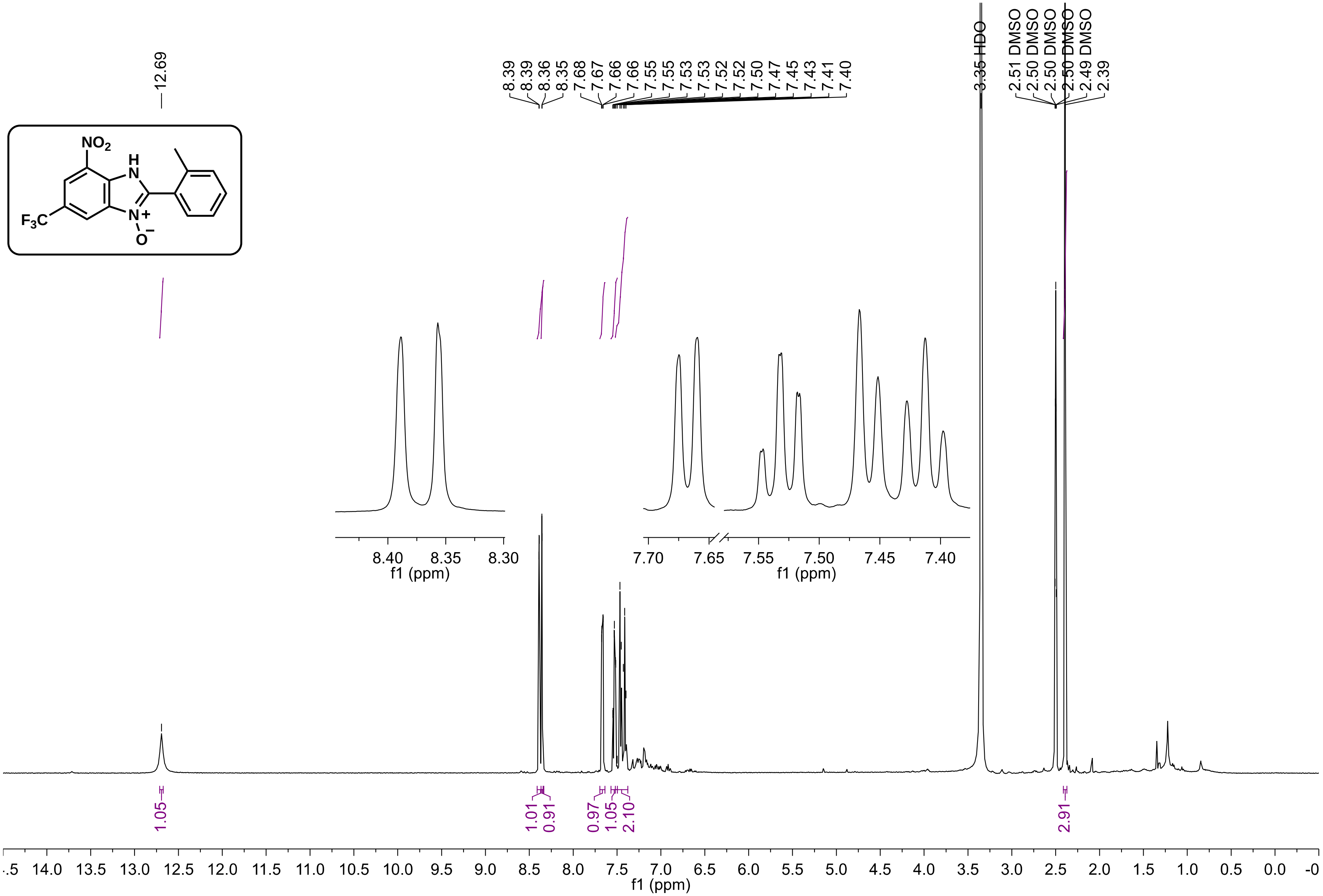

^{1}H-NMR (400 MHz $(CD_3)_2SO$). **2-(2-fluorophenyl)-7-nitro-5-trifluoromethyl-1*H*-benzimidazole 3-oxide (4bc)**

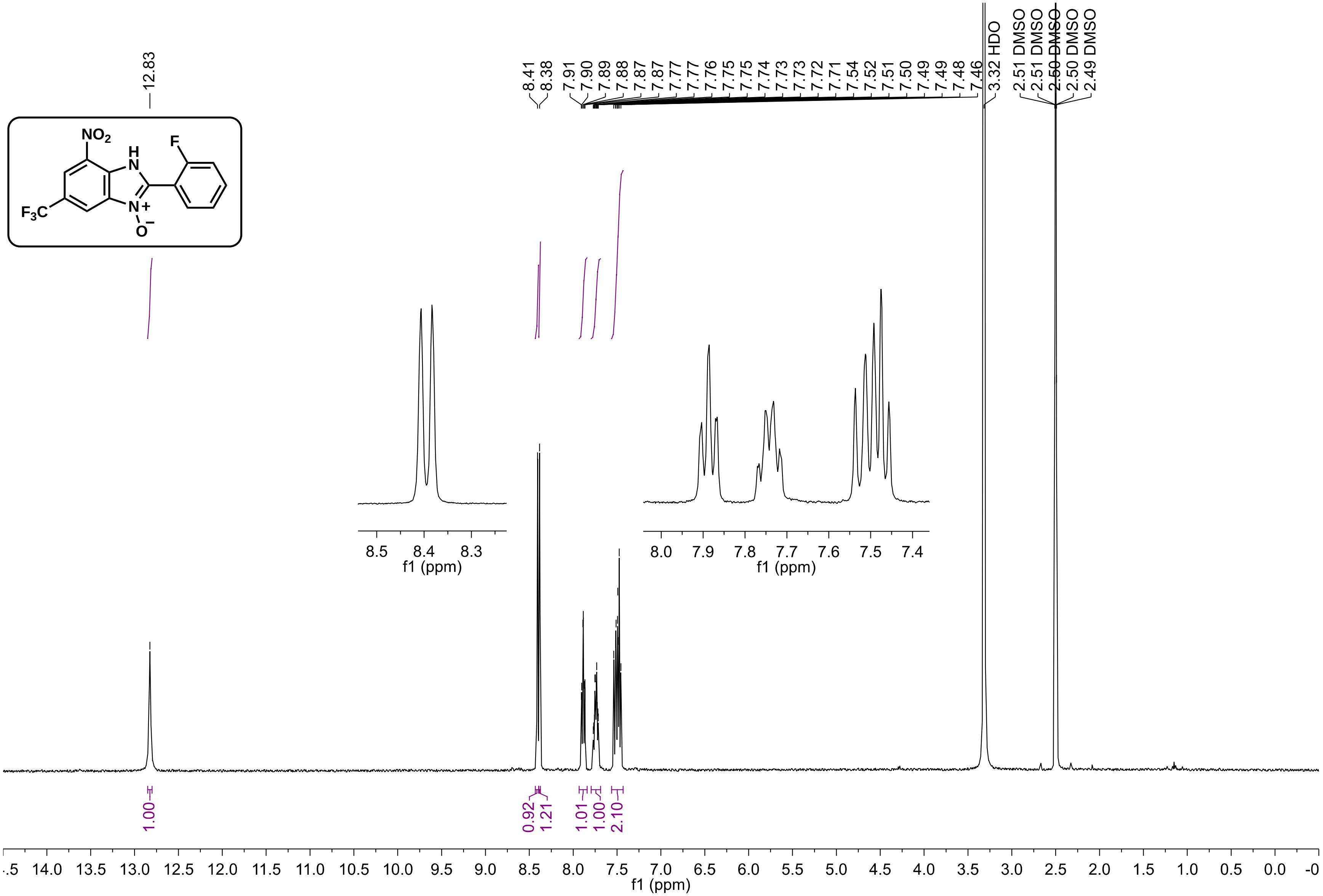

^{1}H-NMR (400 MHz $(CD_3)_2SO$). **2-(2-ethoxyphenyl)-7-nitro-5-trifluoromethyl-1*H*-benzimidazole 3-oxide (4bd)**

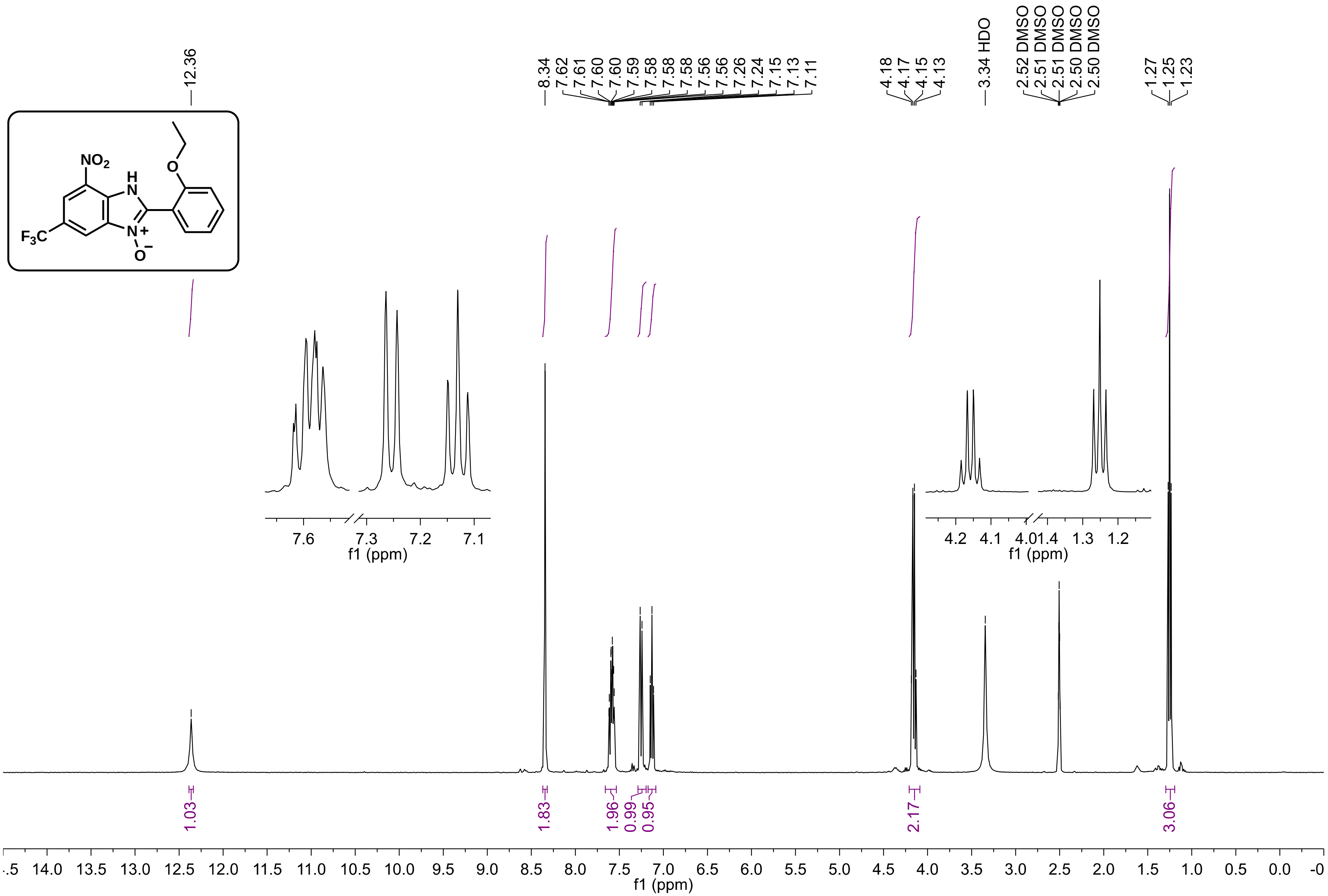

^{1}H-NMR (400 MHz $(CD_3)_2SO$). **2-(3-chlorophenyl)-7-nitro-5-trifluoromethyl-1*H*-benzimidazole 3-oxide (4be)**

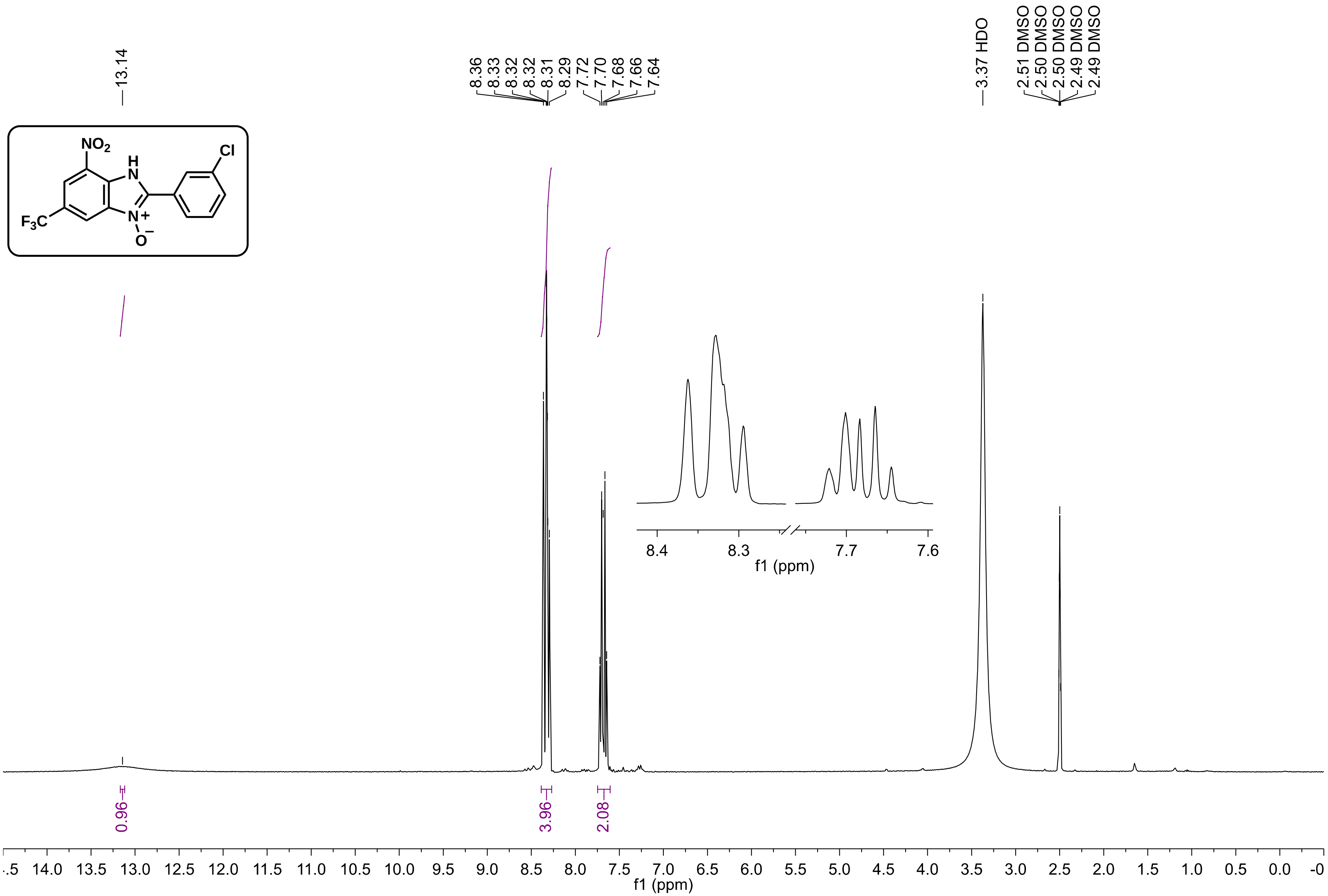

^{1}H-NMR (300 MHz $(CD_3)_2SO$). **2-(3-methoxyphenyl)-7-nitro-5-trifluoromethyl-1*H*-benzimidazole 3-oxide (4bf)**

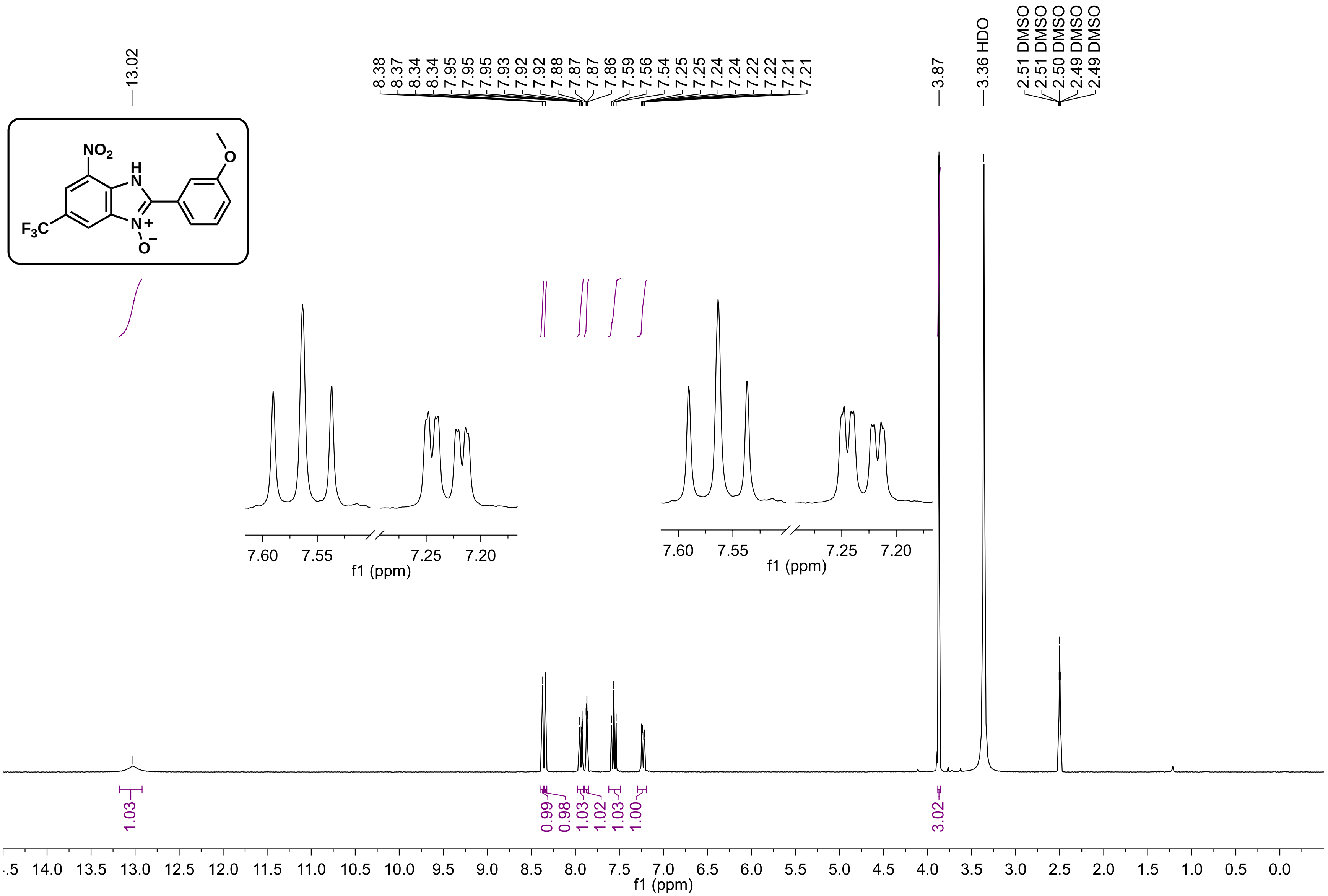

^{1}H-NMR (400 MHz $(CD_3)_2SO$). **7-nitro-2-(*p*-tolyl)-5-trifluoromethyl-1*H*-benzimidazole 3-oxide (4bg)**

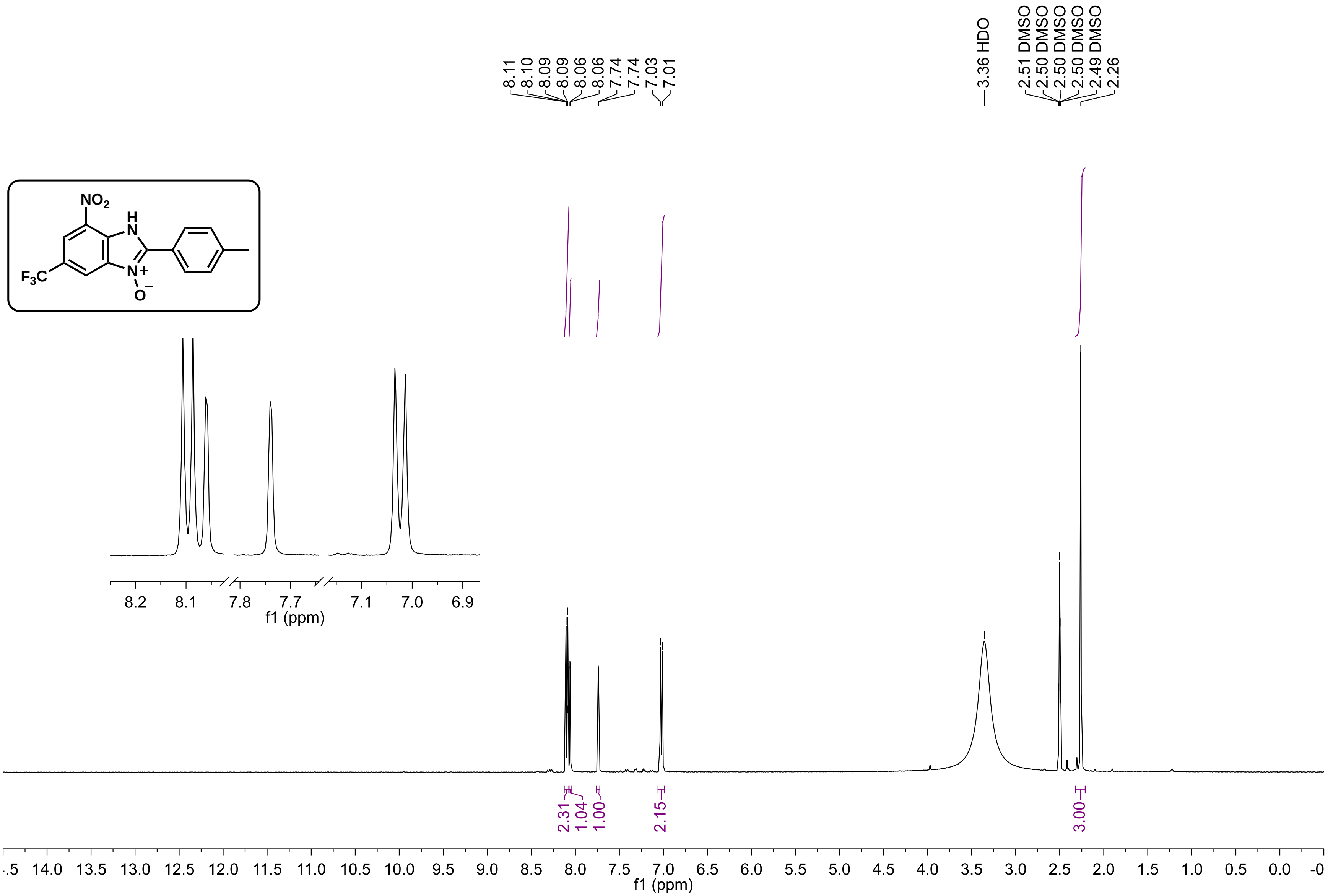

^{1}H-NMR (400 MHz $(CD_3)_2SO$). **2-(4-fluorophenyl)-7-nitro-5-trifluoromethyl-1*H*-benzimidazole 3-oxide (4bh)**

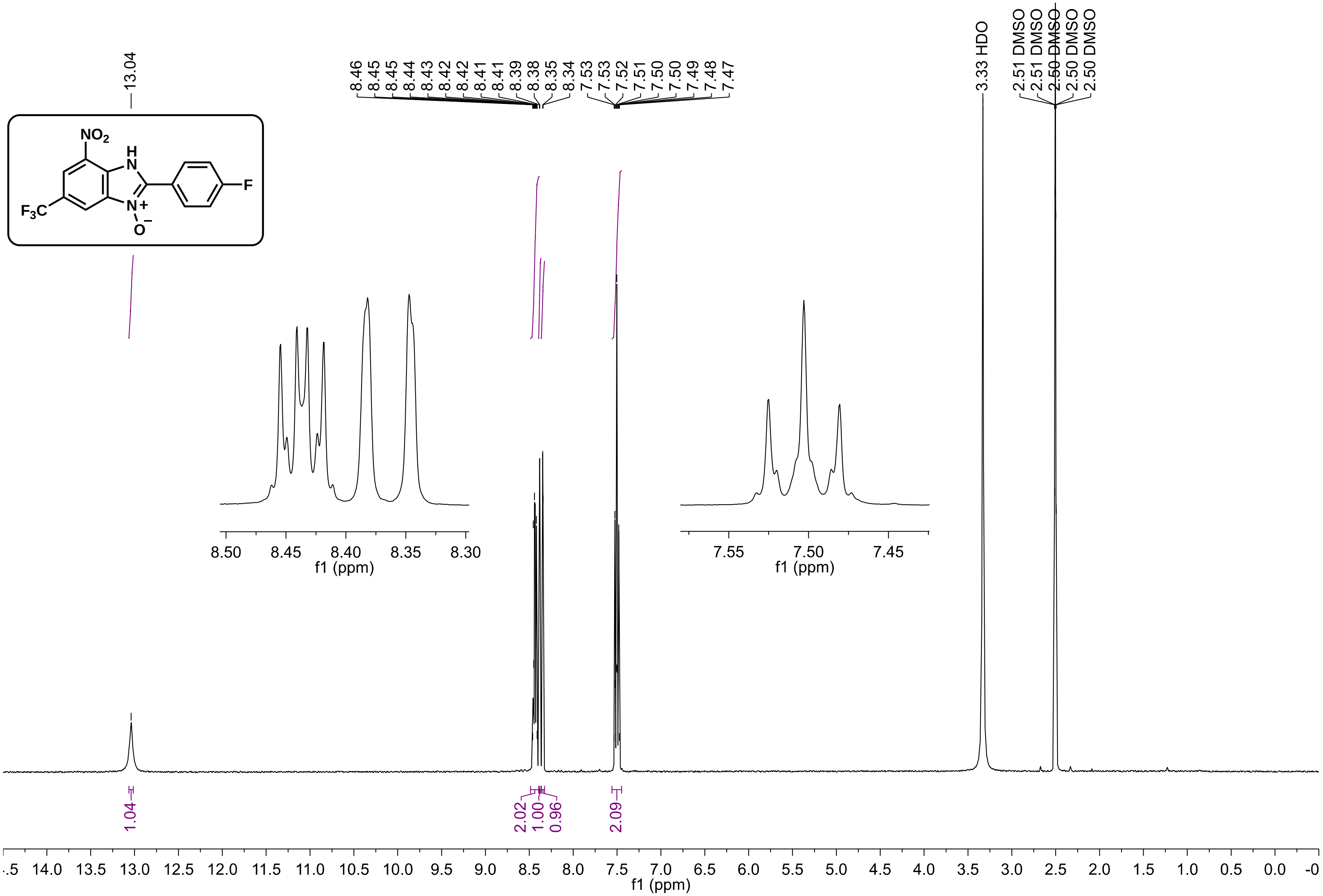

^{1}H-NMR (300 MHz $(CD_3)_2SO$). **2-(4-chlorophenyl)-7-nitro-5-trifluoromethyl-1*H*-benzimidazole 3-oxide (4bi)**

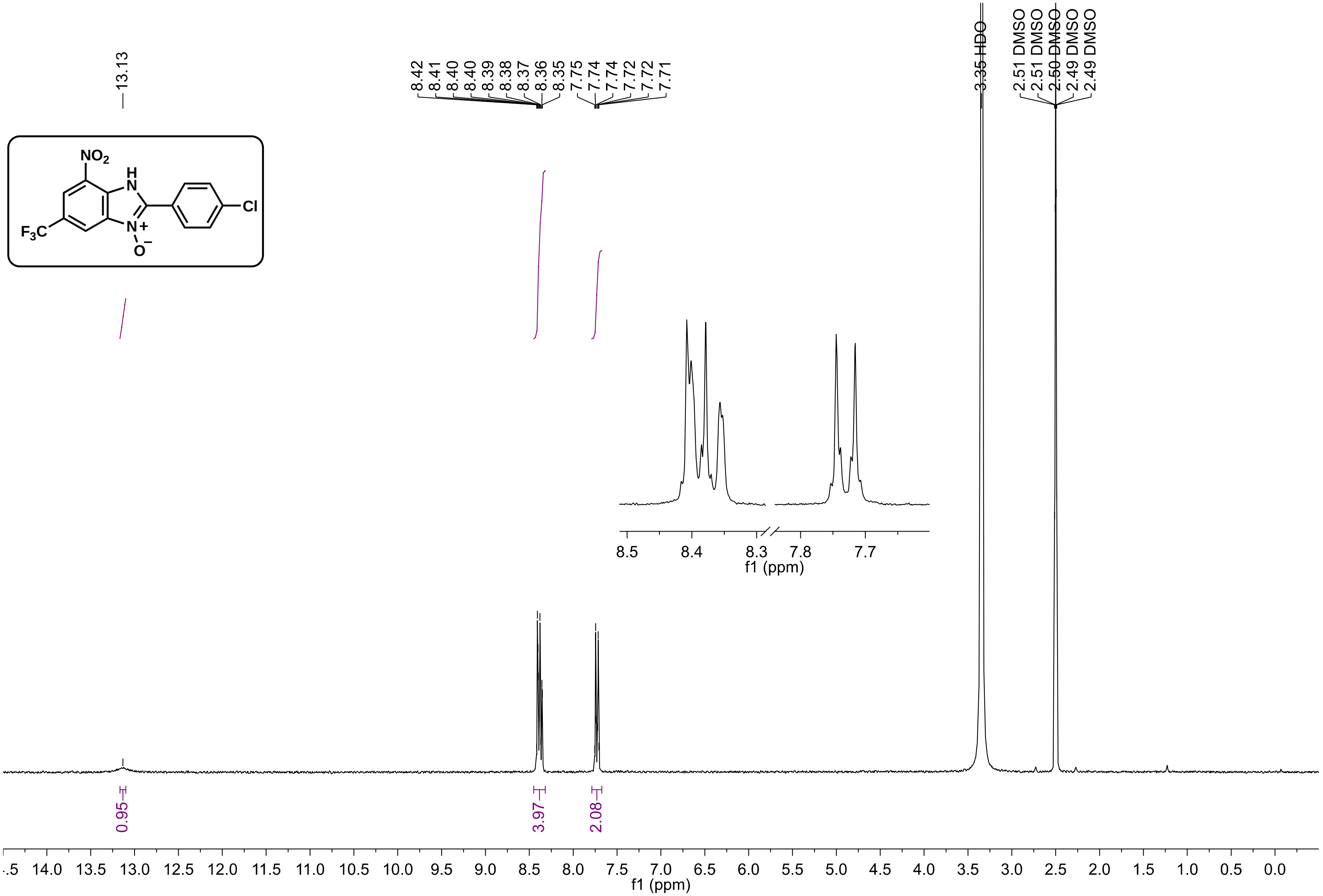

^{1}H-NMR (300 MHz $(CD_3)_2SO$). **2-(4-methoxyphenyl)-7-nitro-5-trifluoromethyl-1*H*-benzimidazole 3-oxide (4bj)**

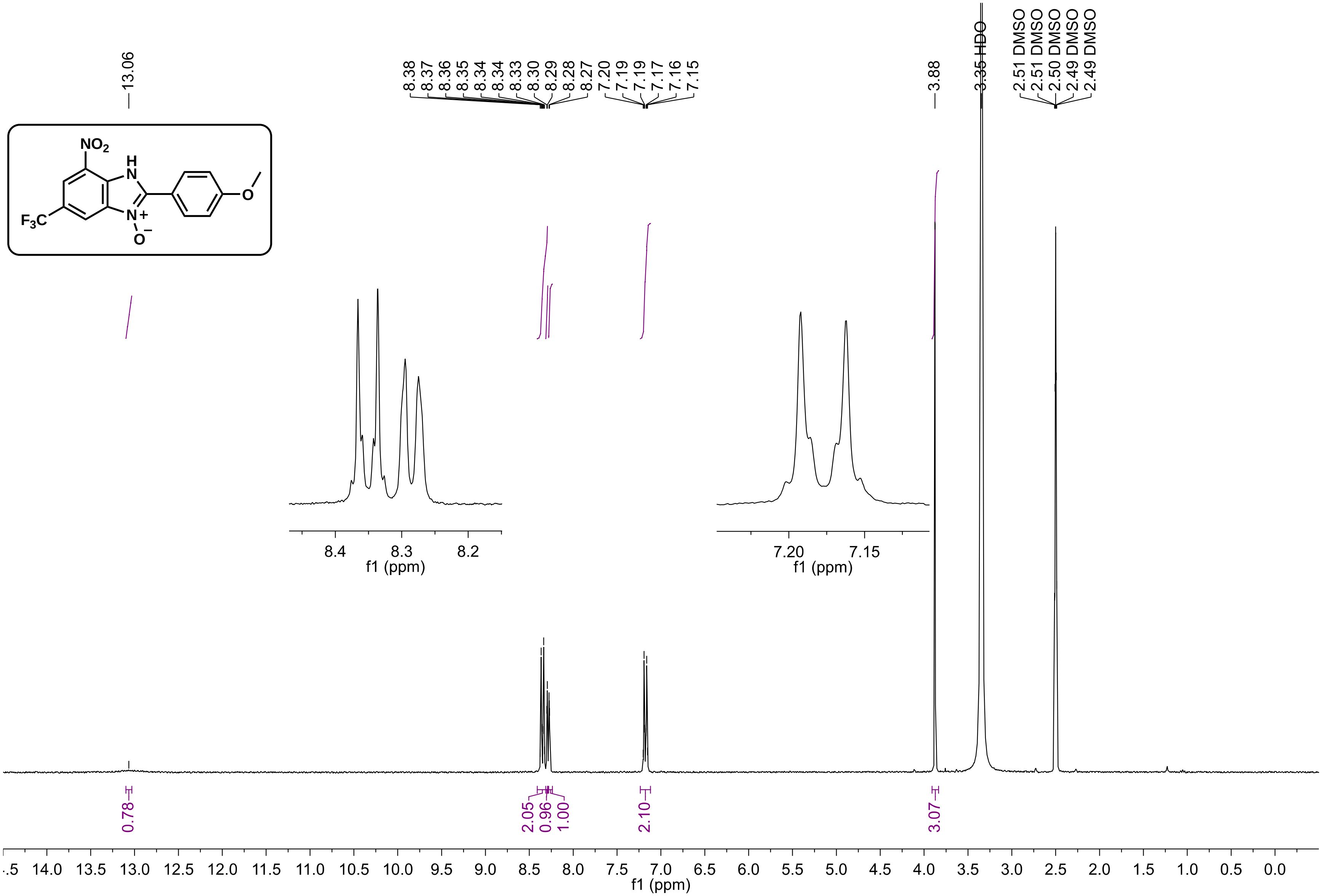

^{1}H-NMR (400 MHz $(CD_3)_2SO$). **2-(furan-2-yl)-7-nitro-5-trifluoromethyl-1*H*-benzimidazole 3-oxide (4bk)**

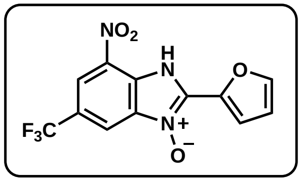

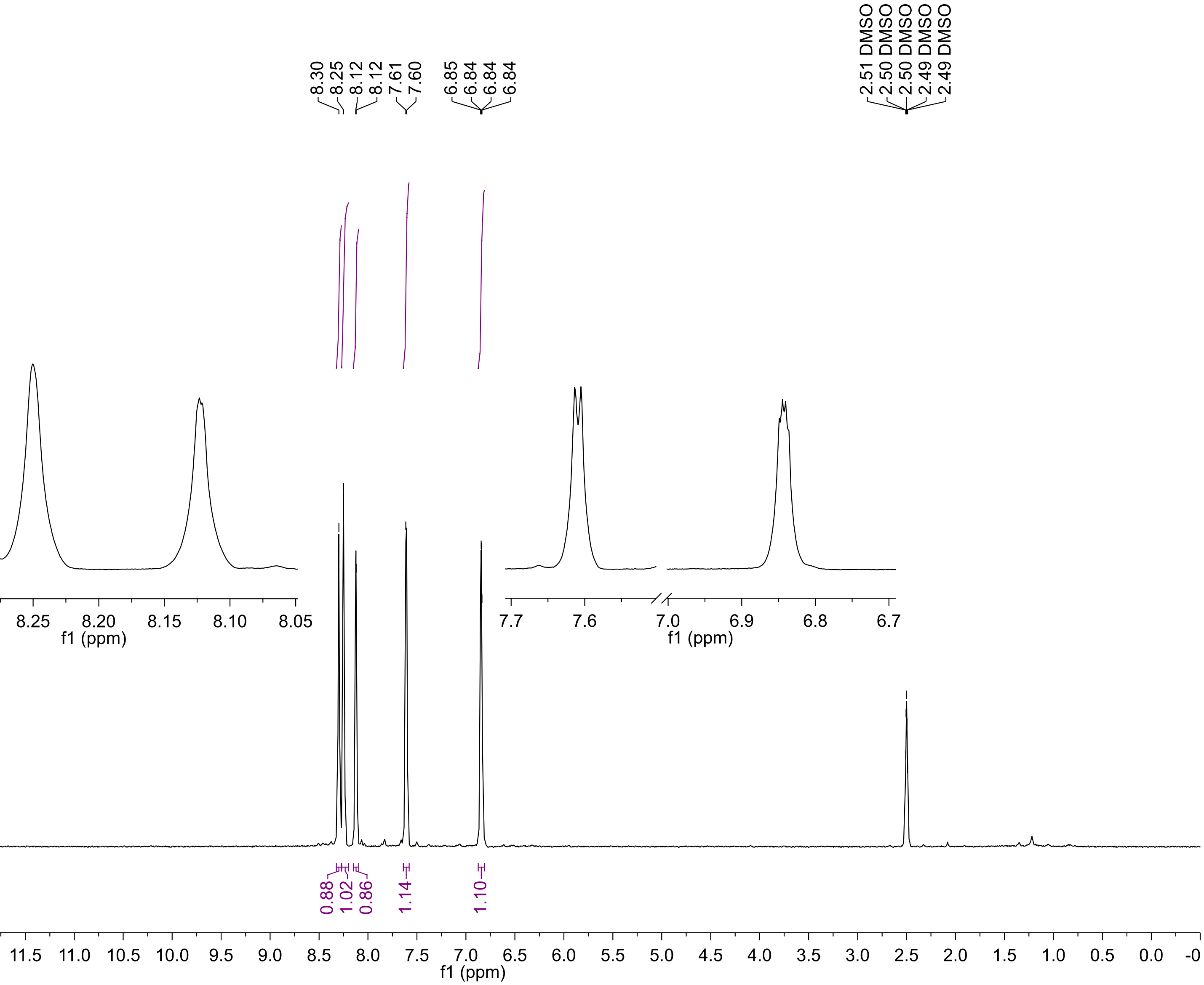

^{1}H-NMR (300 MHz $(CD_3)_2SO$). **7-nitro-2-(thiophen-2-yl)-5-trifluoromethyl-1*H*-benzimidazole 3-oxide (4bl)**

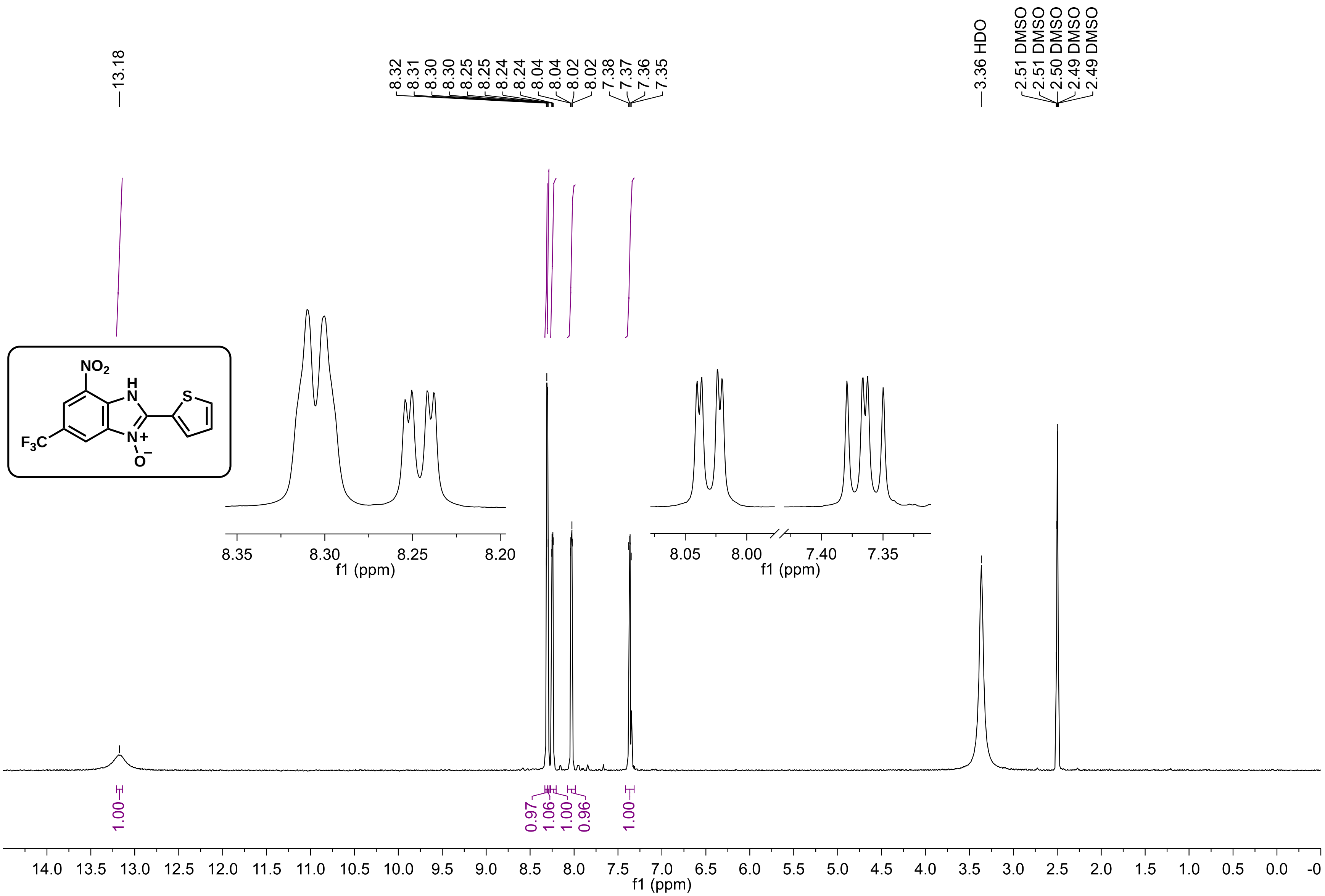

^{1}H-NMR (400 MHz $(CD_3)_2SO$). **7-nitro-2-(pyridin-3-yl)-5-trifluoromethyl-1*H*-benzimidazole 3-oxide (4bm)**

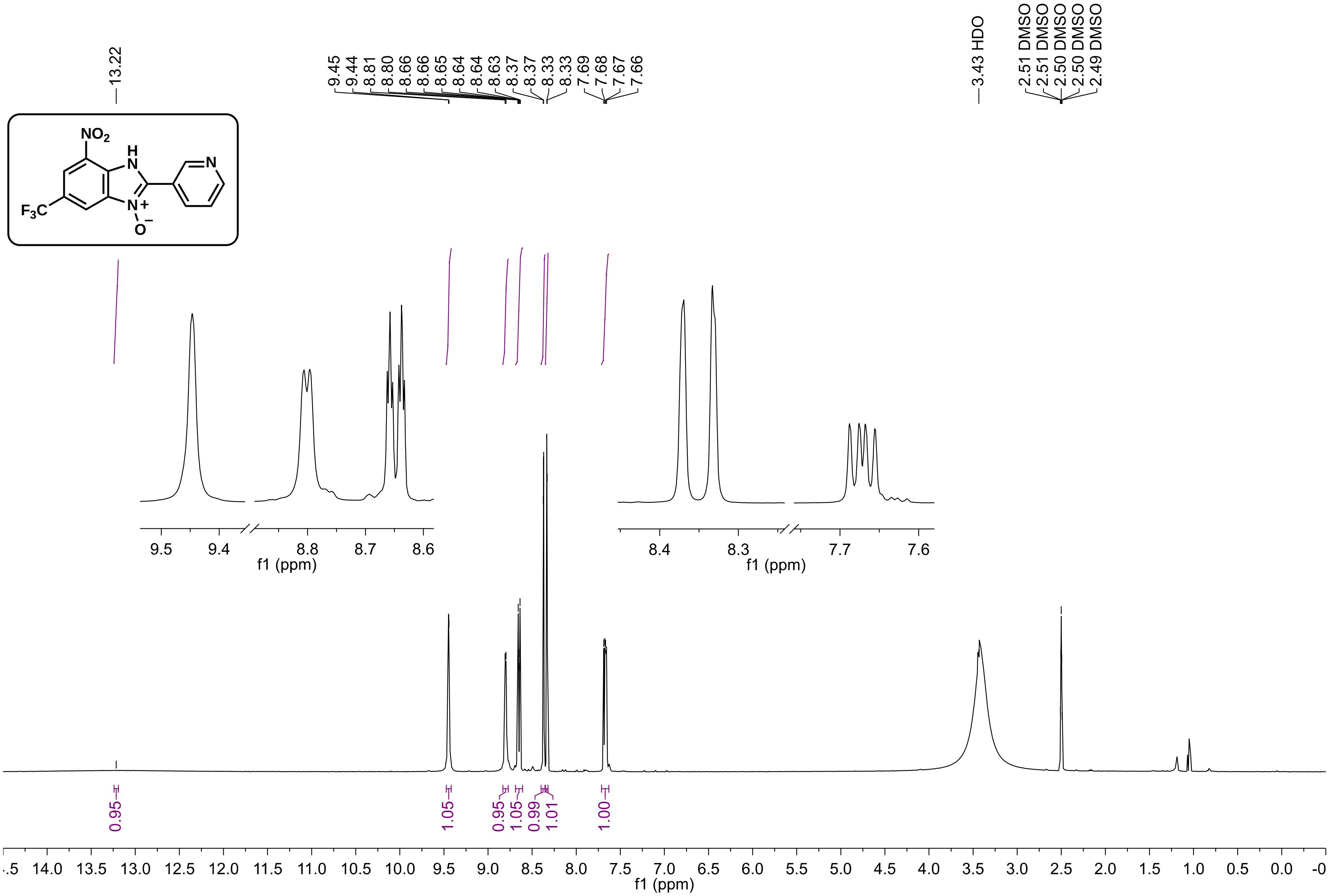

^{1}H-NMR (300 MHz $(CD_3)_2SO$). **2-benzyl-7-nitro-5-trifluoromethyl-1*H*-benzimidazole 3-oxide (4bn)**

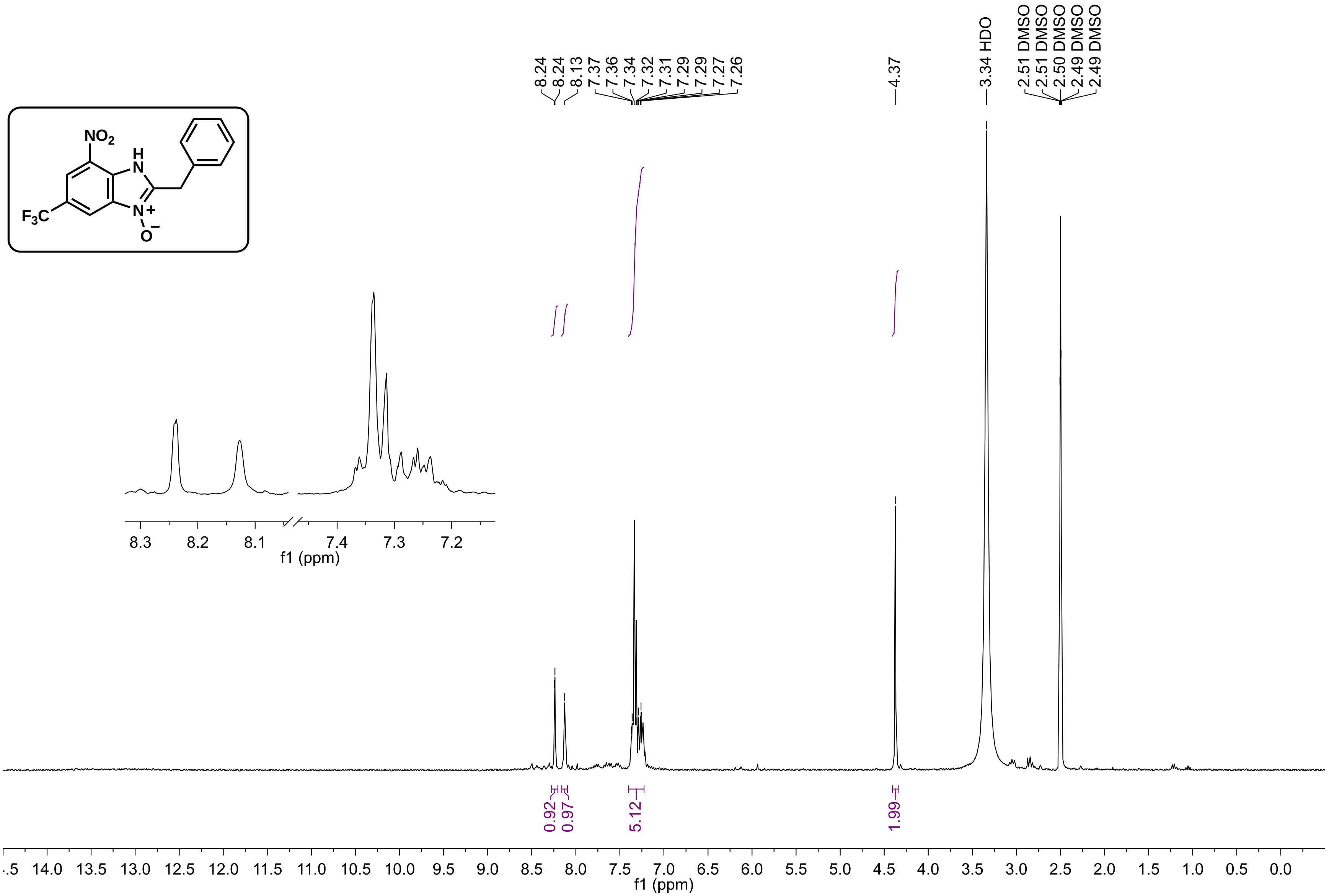

^{1}H-NMR (300 MHz $(CD_3)_2SO$). **5-nitro-2-phenyl-7-trifluoromethyl-1*H*-benzimidazole 3-oxide (4ca)**

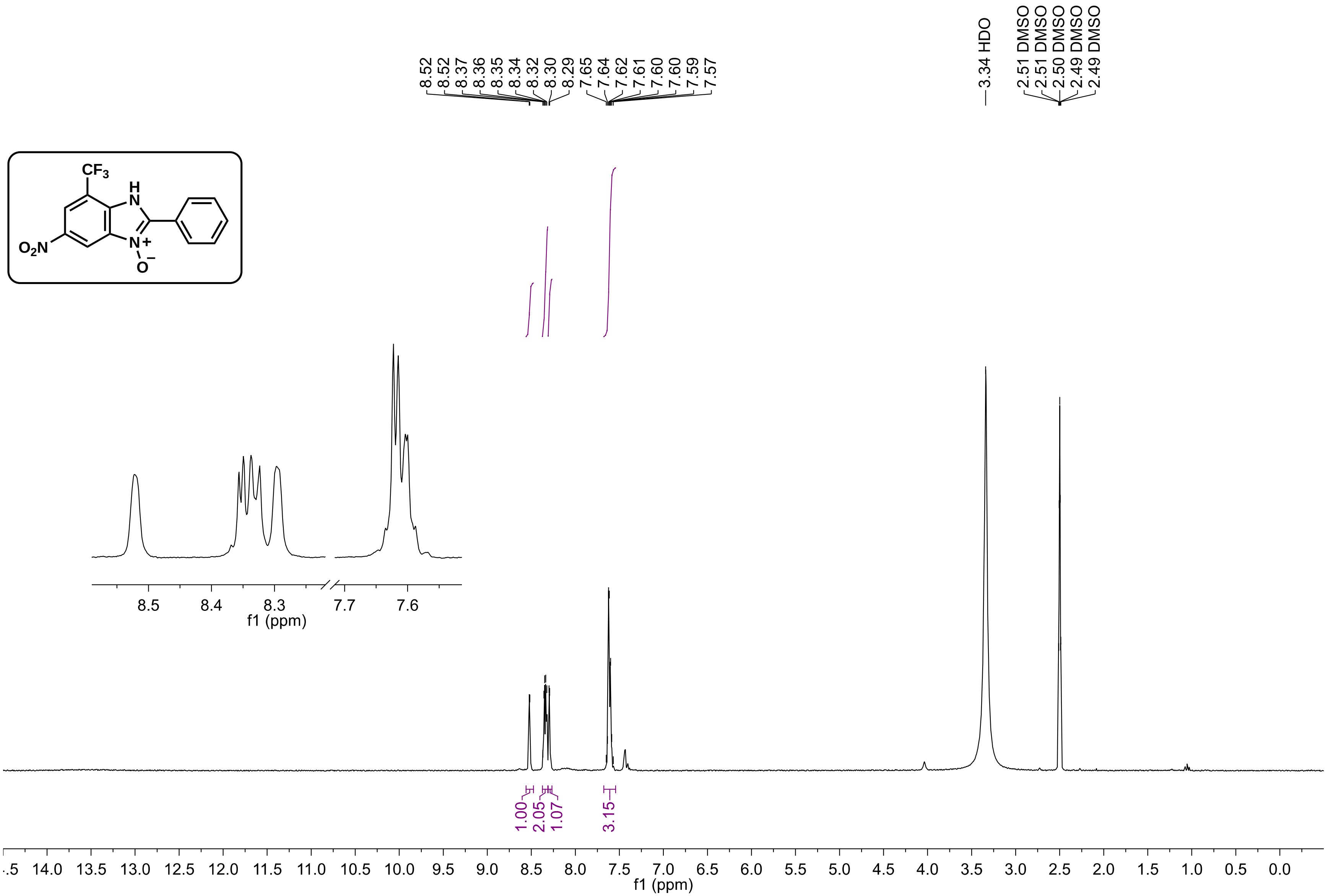

^{1}H-NMR (300 MHz $(CD_3)_2SO$). **5-nitro-2-(*o*-tolyl)-7-trifluoromethyl-1*H*-benzimidazole 3-oxide (4cb)**

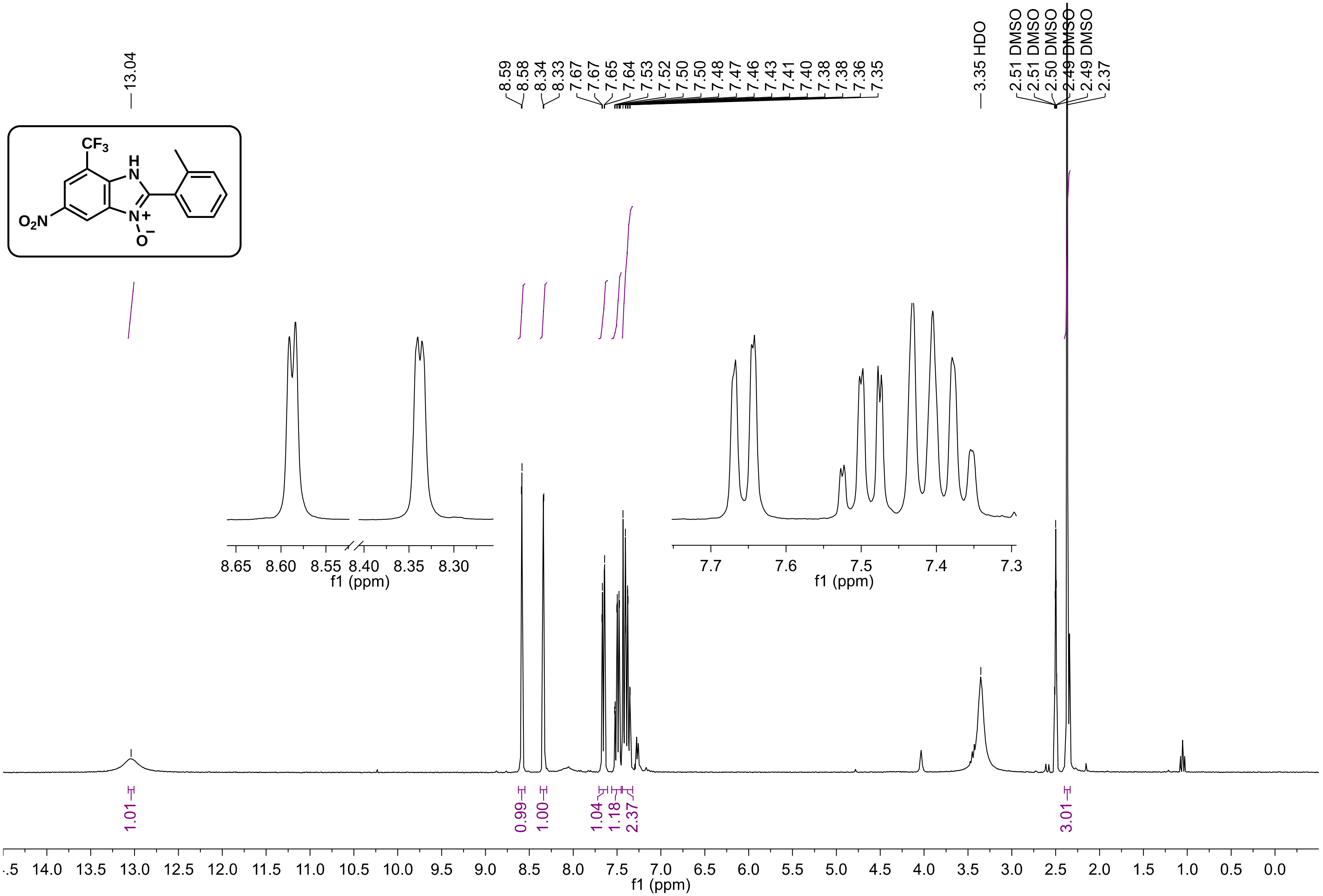

^{1}H-NMR (300 MHz $(CD_3)_2SO$). **2-(2-fluorophenyl)-5-nitro-7-trifluoromethyl-1*H*-benzimidazole 3-oxide (4cc)**

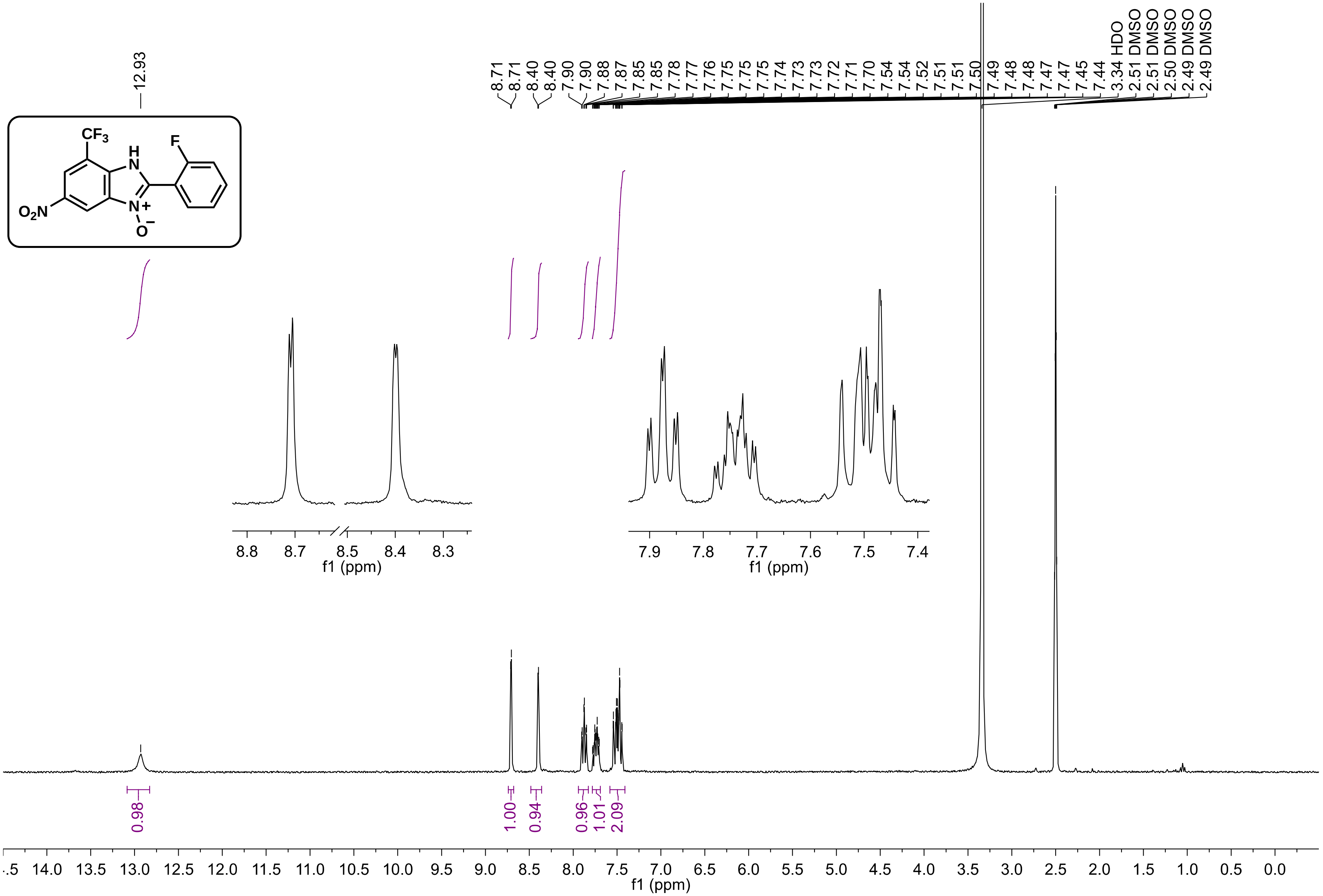

^{1}H-NMR (400 MHz $(CD_3)_2SO$). **2-(2-ethoxyphenyl)-5-nitro-7-trifluoromethyl-1*H*-benzimidazole 3-oxide (4cd)**

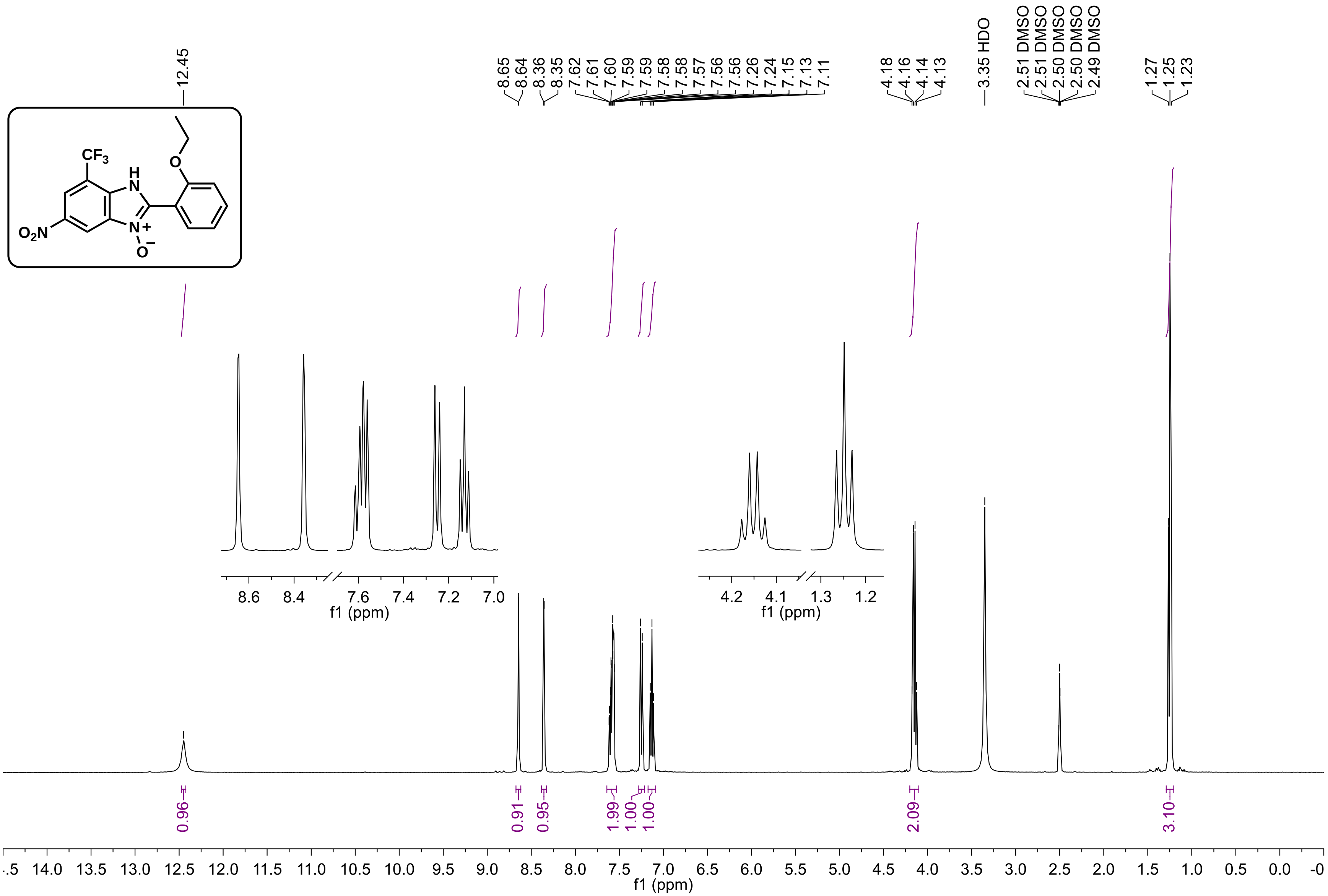

^{1}H-NMR (400 MHz $(CD_3)_2SO$). **2-(3-chlorophenyl)-5-nitro-7-trifluoromethyl-1*H*-benzimidazole 3-oxide (4ce)**

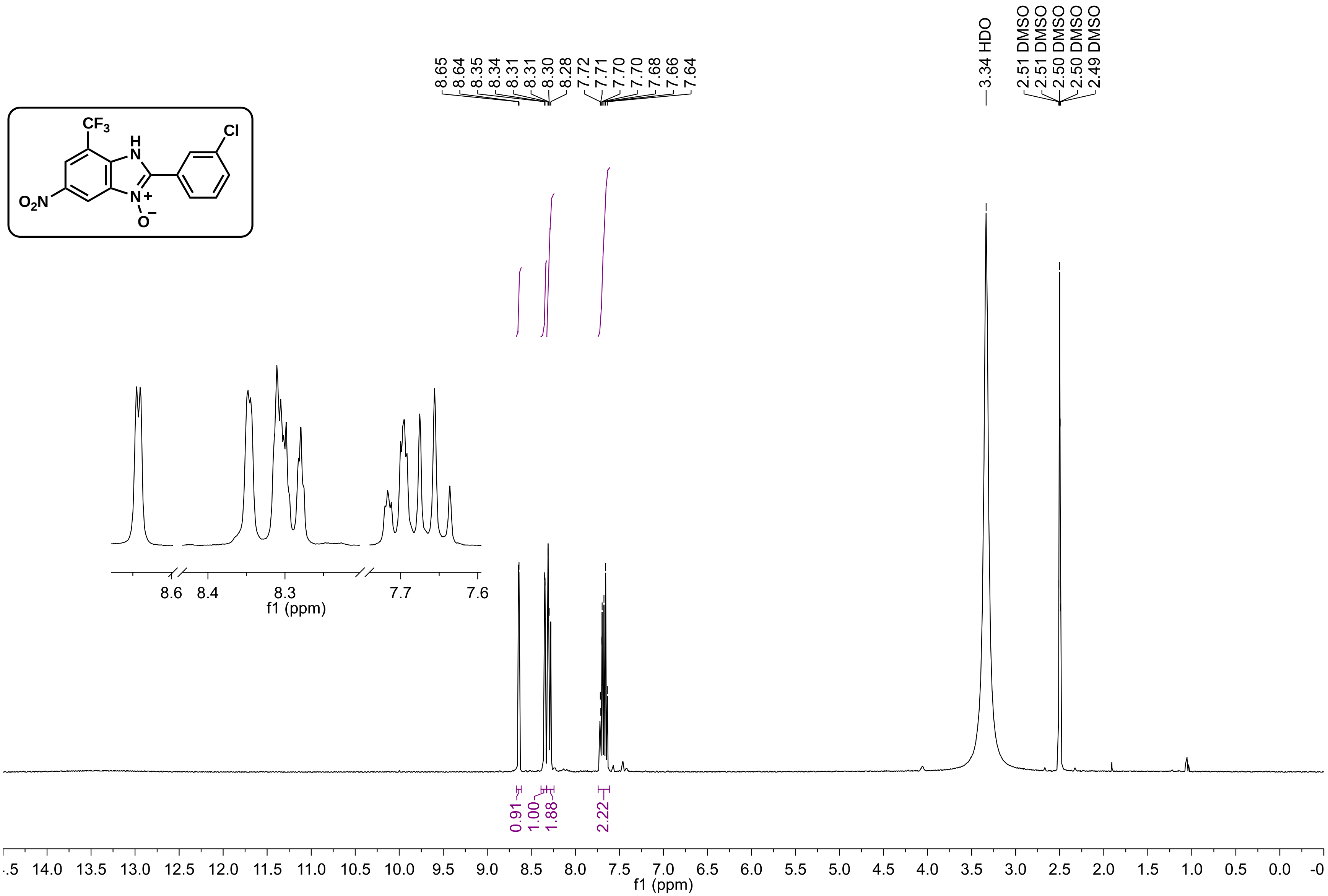

^{1}H-NMR (300 MHz $(CD_3)_2SO$). **2-(3-methoxyphenyl)-5-nitro-7-trifluoromethyl-1*H*-benzimidazole 3-oxide (4cf)**

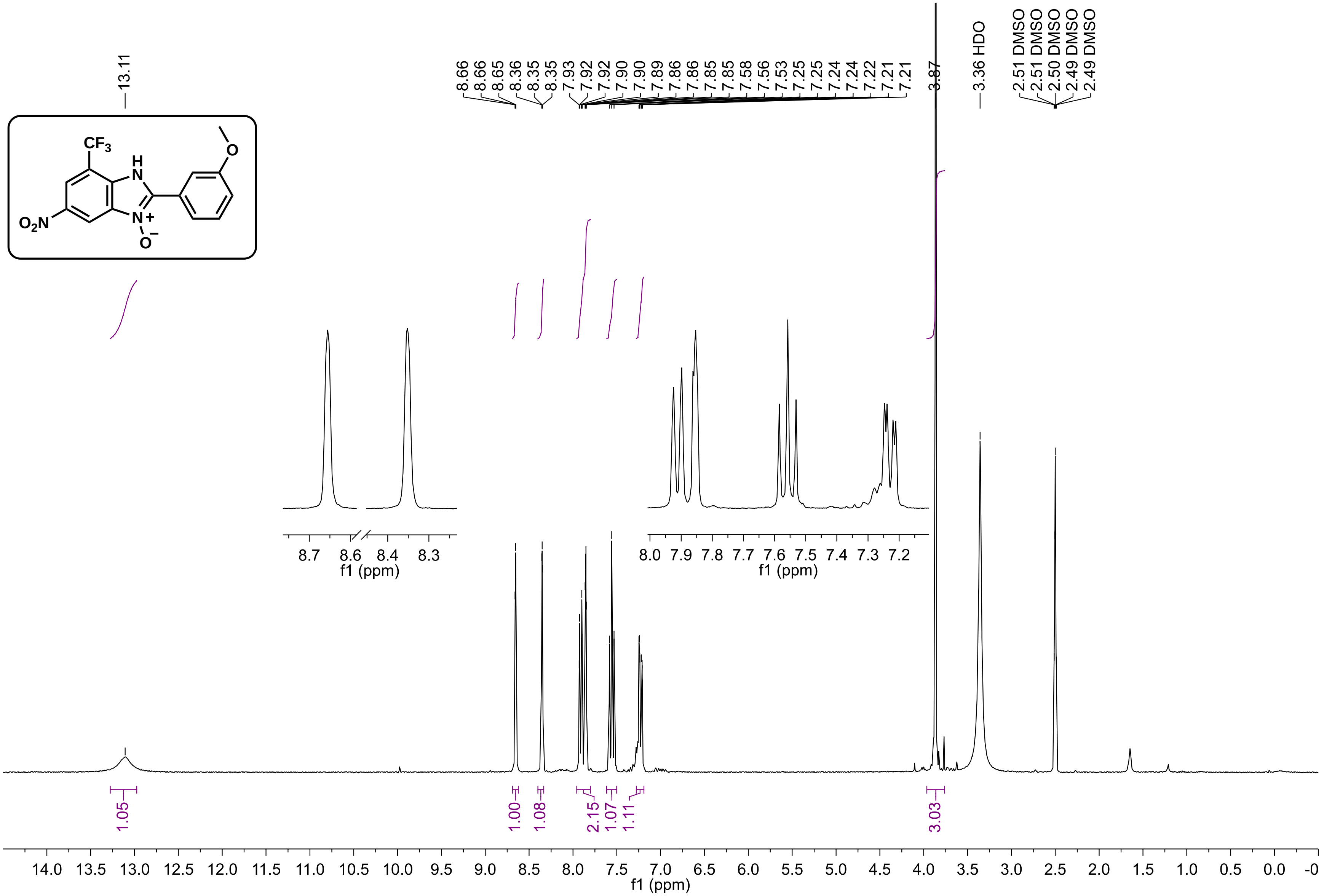

^{1}H-NMR (300 MHz $(CD_3)_2SO$). **5-nitro-2-(*p*-tolyl)-7-trifluoromethyl-1*H*-benzimidazole 3-oxide (4cg)**

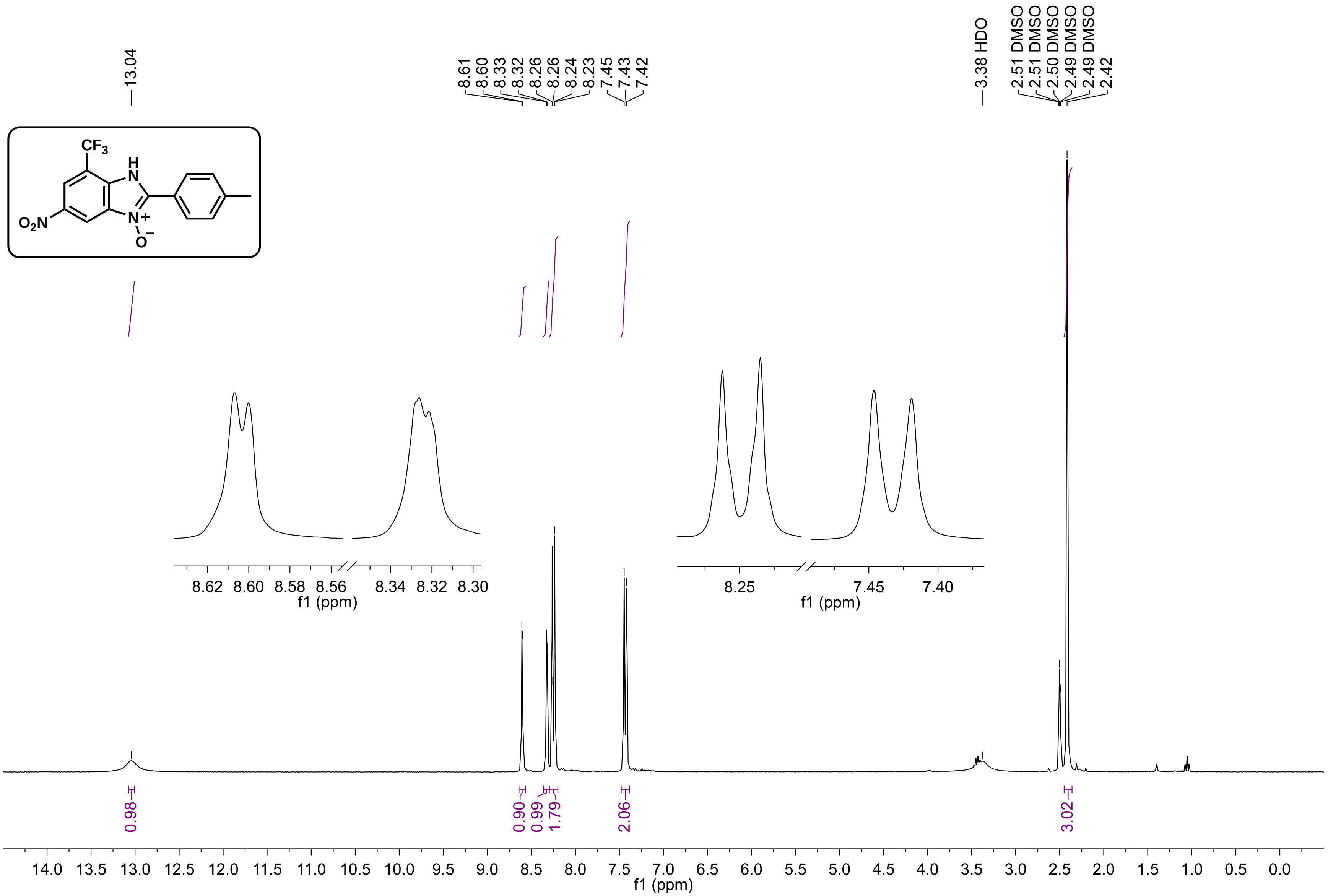

^{1}H-NMR (300 MHz $(CD_3)_2SO$). **2-(4-fluorophenyl)-5-nitro-7-trifluoromethyl-1*H*-benzimidazole 3-oxide (4ch)**

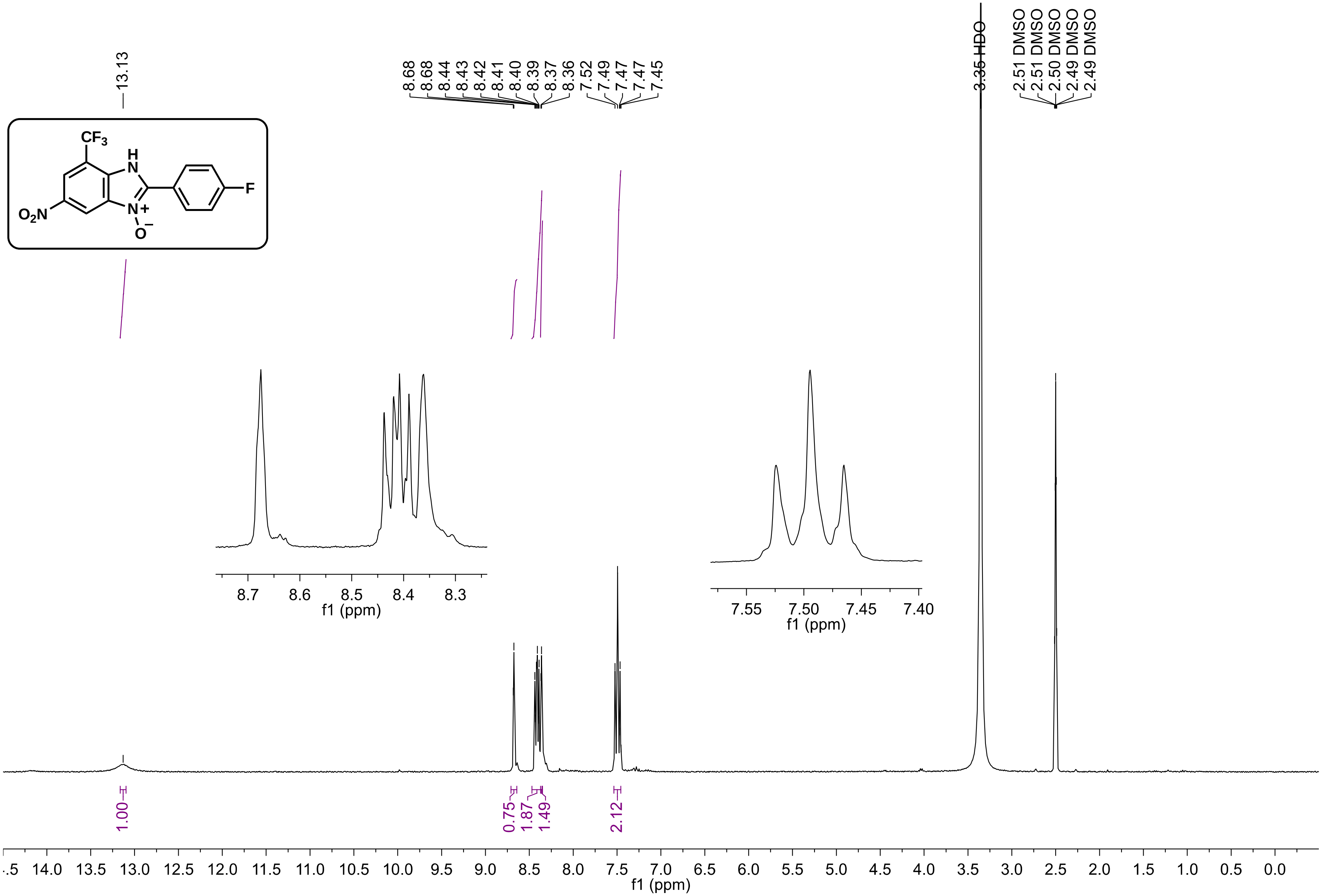

^{1}H-NMR (400 MHz $(CD_3)_2SO$). **2-(4-chlorophenyl)-5-nitro-7-trifluoromethyl-1*H*-benzimidazole 3-oxide (4ci)**

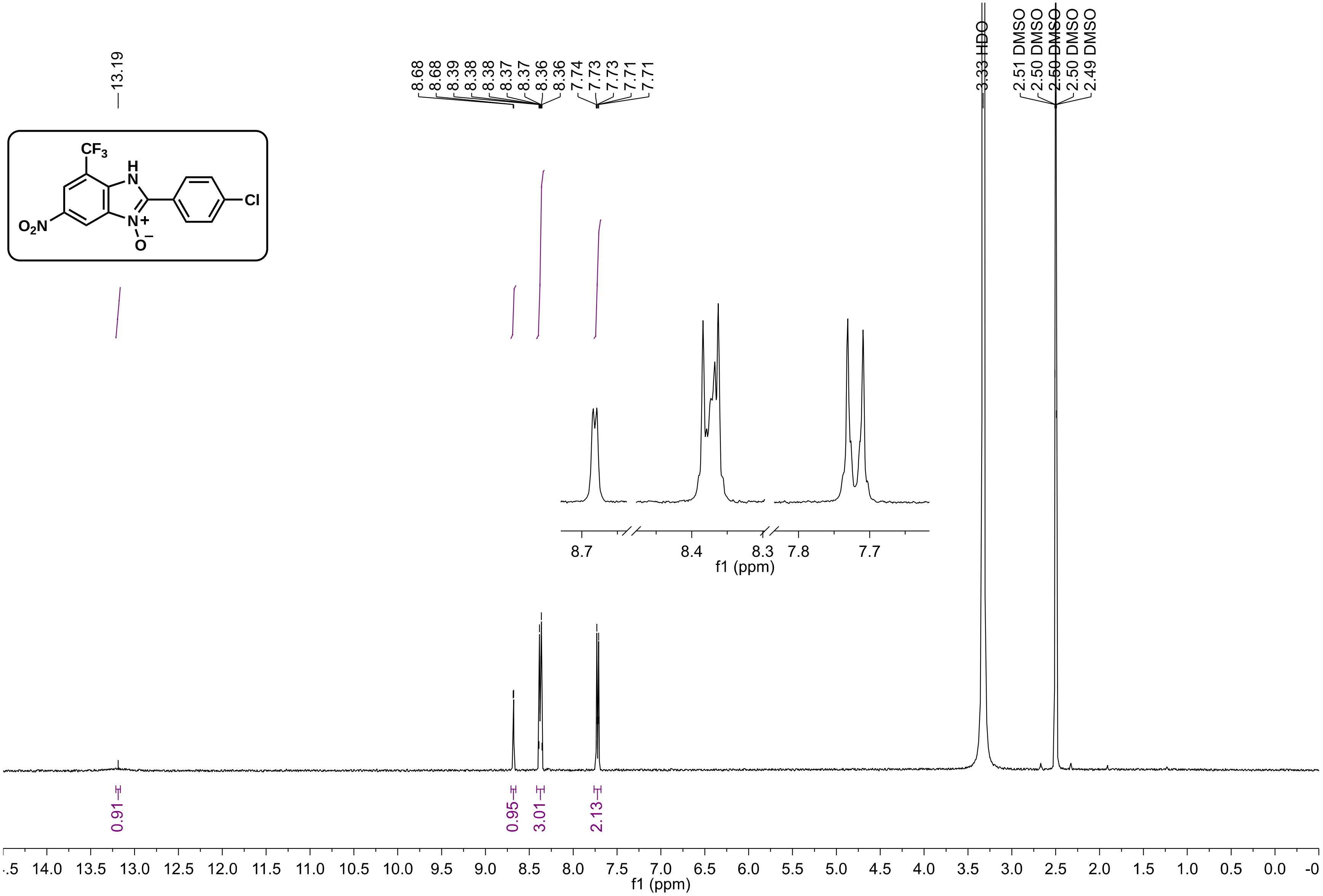

^{1}H-NMR (400 MHz $(CD_3)_2SO$). **2-(4-methoxyphenyl)-5-nitro-7-trifluoromethyl-1*H*-benzimidazole 3-oxide (4cj)**

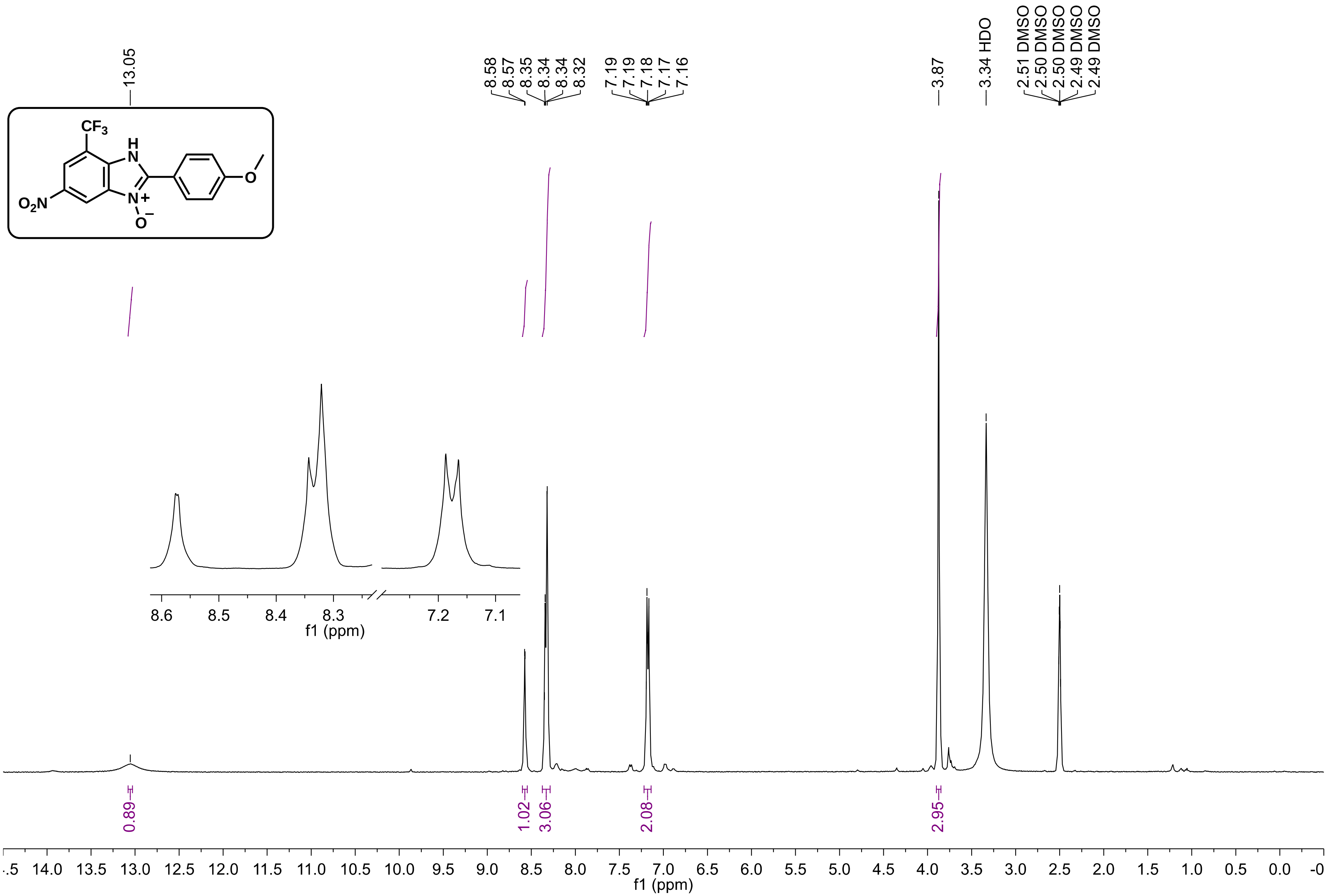

^{1}H-NMR (300 MHz $(CD_3)_2SO$). **2-(furan-2-yl)-5-nitro-7-trifluoromethyl-1*H*-benzimidazole 3-oxide (4ck)**

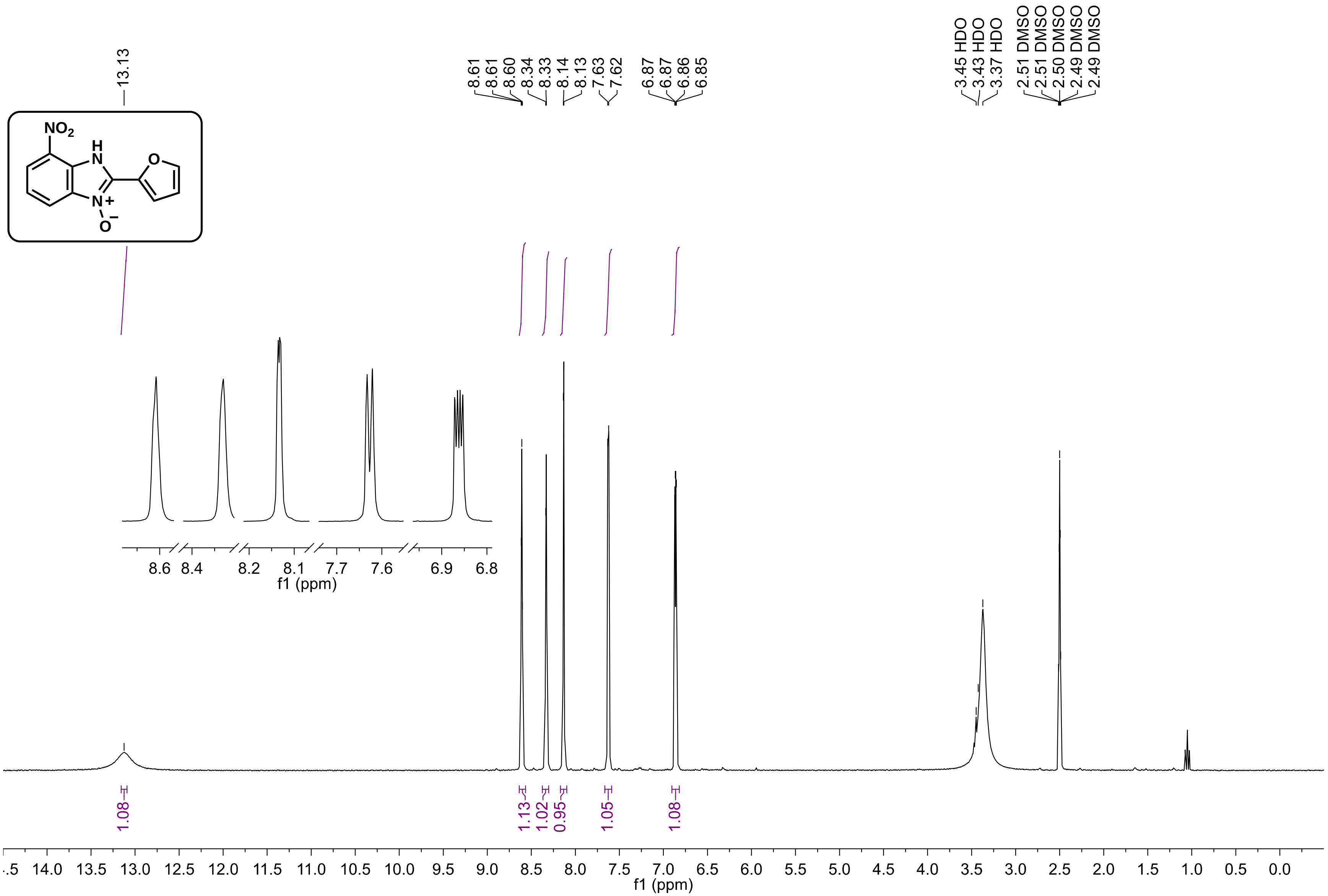

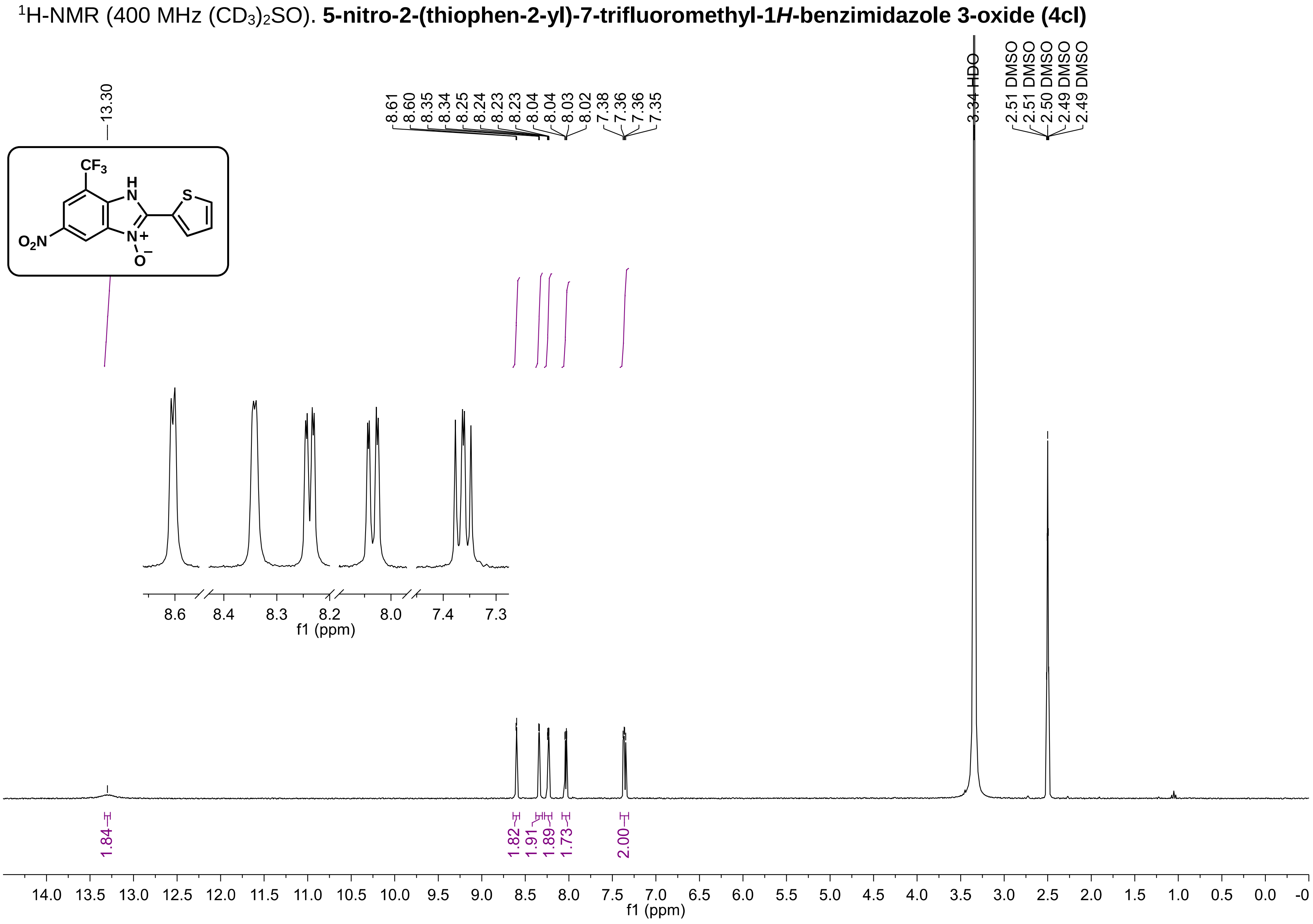
^{1}H-NMR (400 MHz $(CD_3)_2SO$). **5-nitro-2-(thiophen-2-yl)-7-trifluoromethyl-1*H*-benzimidazole 3-oxide (4cl)**
13.30
8.61
8.60
8.35
8.34
8.25
8.24
8.23
8.23
8.04
8.04
8.03
8.02
7.38
7.36
7.36
7.35
3.34 HDO
2.51 DMSO
2.51 DMSO
2.50 DMSO
2.49 DMSO
2.49 DMSO
CF_3
H
N
S
O_2N
N+
O−
1.84
1.82
1.91
1.89
1.73
2.00
f1 (ppm)

^{1}H-NMR (300 MHz $(CD_3)_2SO$). **5-nitro-2-(pyridin-3-yl)-7-trifluoromethyl-1*H*-benzimidazole 3-oxide (4cm)**

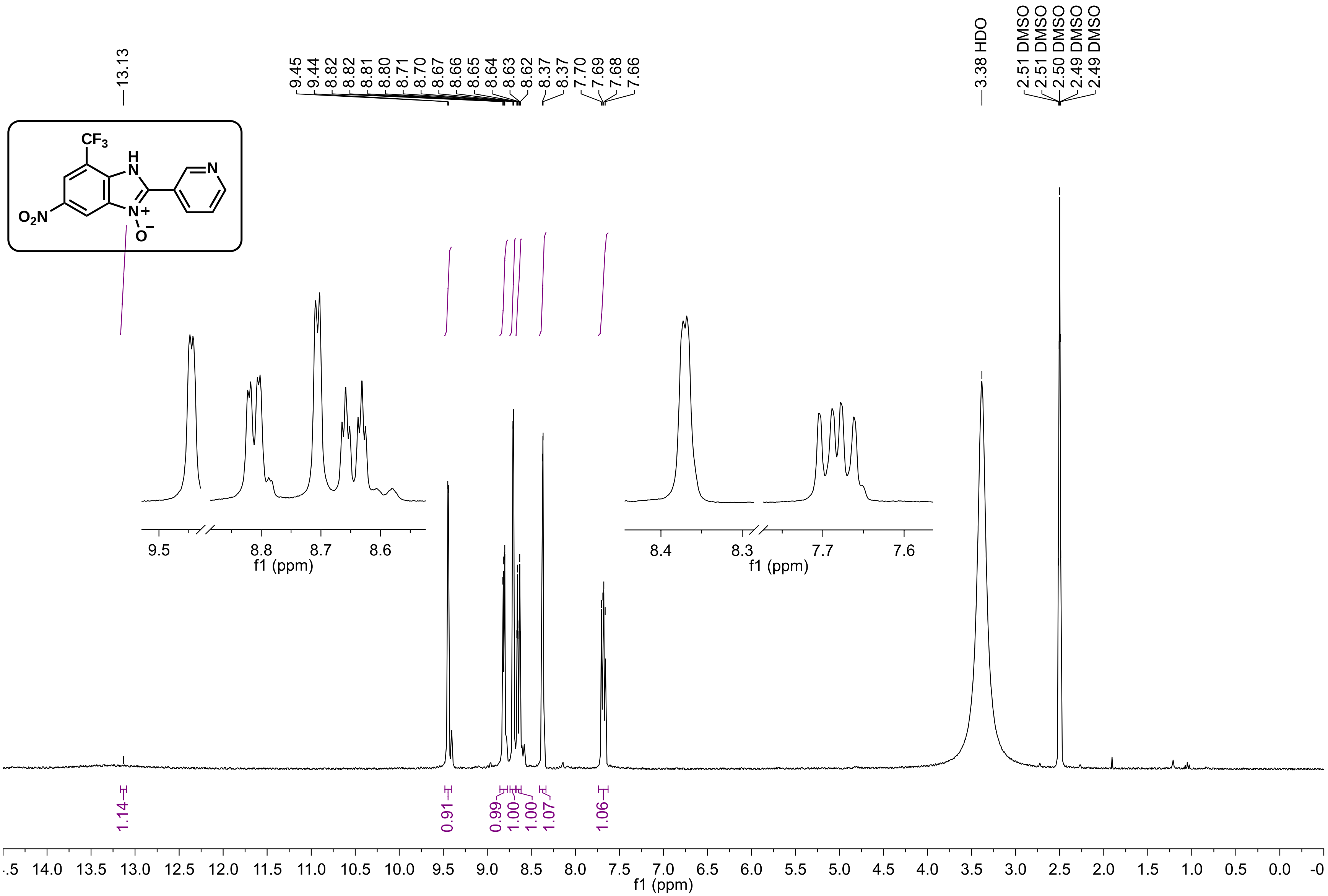

^{1}H-NMR (300 MHz $(CD_3)_2SO$). **2-benzyl-5-nitro-7-trifluoromethyl-1*H*-benzimidazole 3-oxide (4cn)**

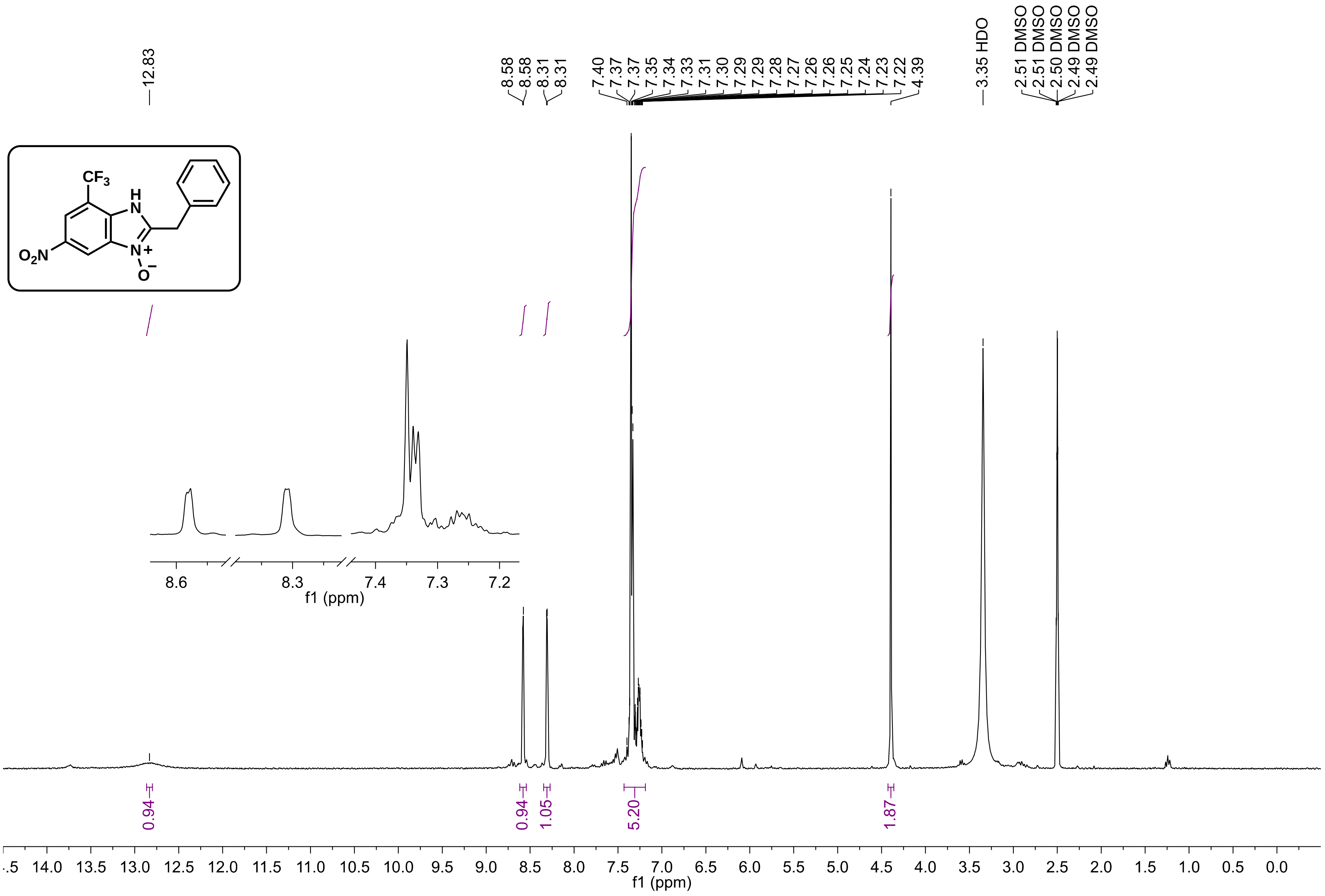

^{13}C-NMR (101 MHz $(CD_3)_2SO$). **7-nitro-2-phenyl-1*H*-benzimidazole 3-oxide (4aa)**

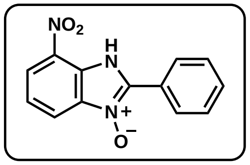

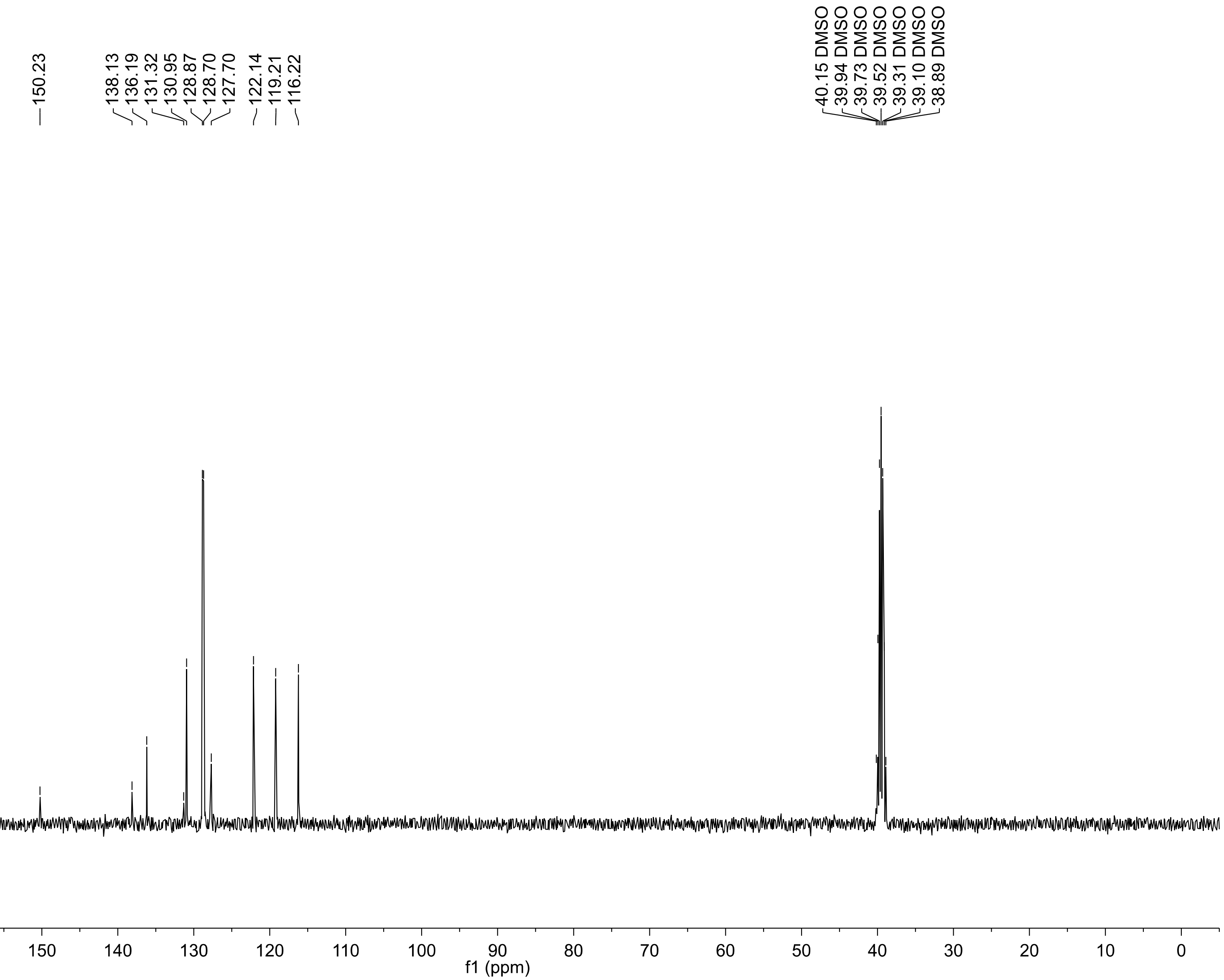

^{13}C-NMR (101 MHz $(CD_3)_2SO$). **7-nitro-2-(*o*-tolyl)-1*H*-benzimidazole 3-oxide (4ab)**

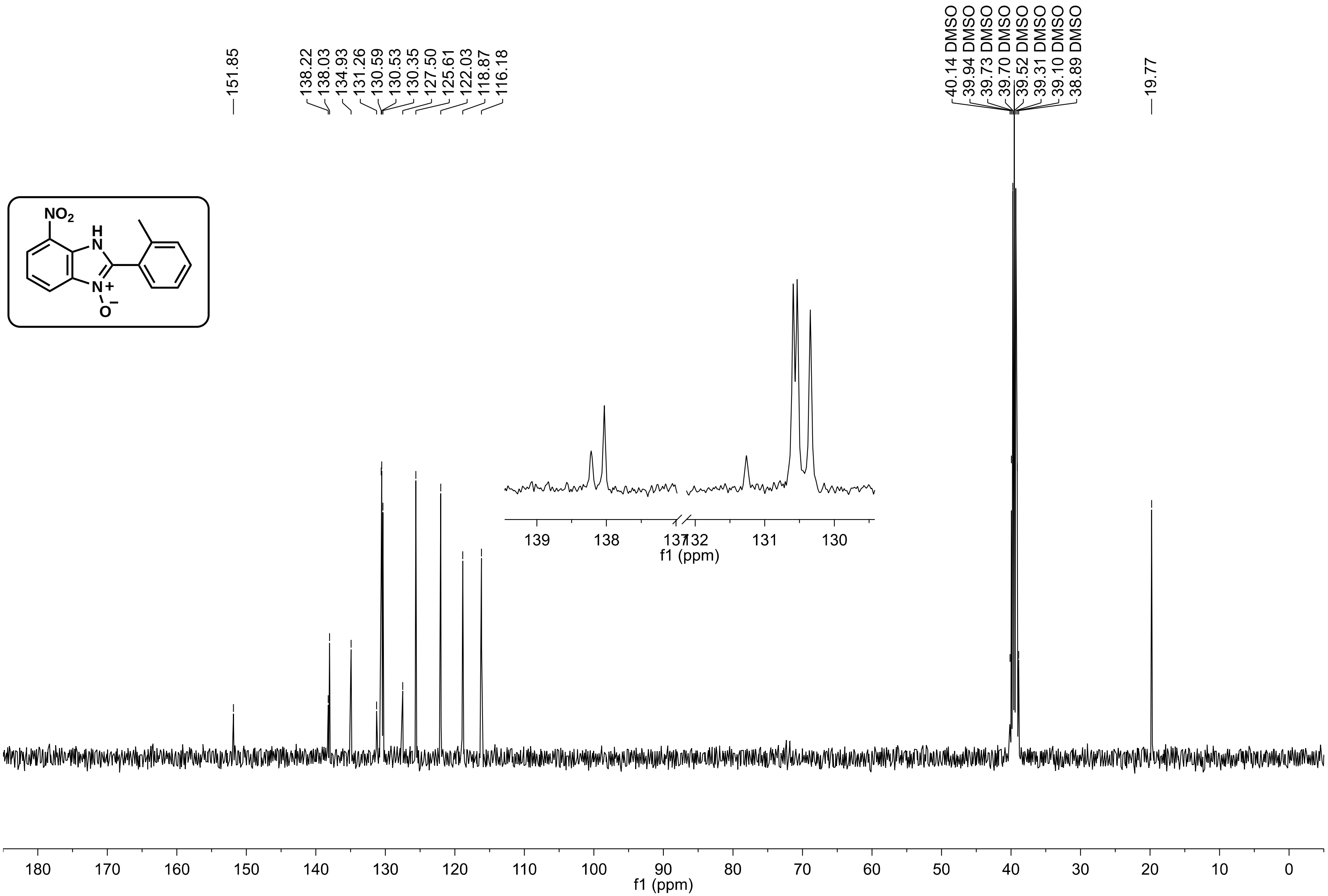

^{13}C-NMR (126 MHz $(CD_3)_2SO$). **2-(2-fluorophenyl)-7-nitro-1*H*-benzimidazole 3-oxide (4ac)**

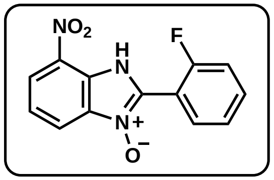

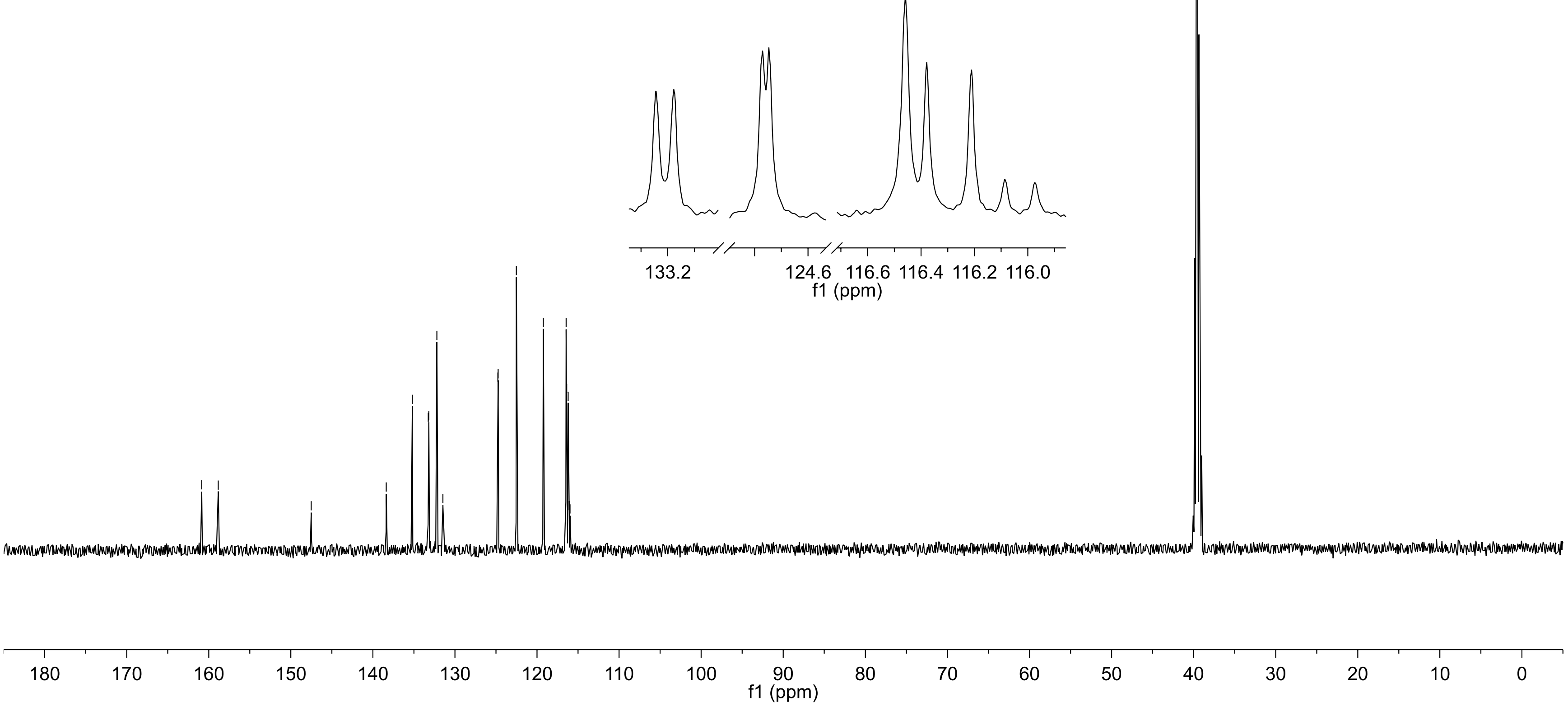

^{13}C-NMR (126 MHz $(CD_3)_2SO$). **2-(2-ethoxyphenyl)-7-nitro-1*H*-benzimidazole 3-oxide (4ad)**

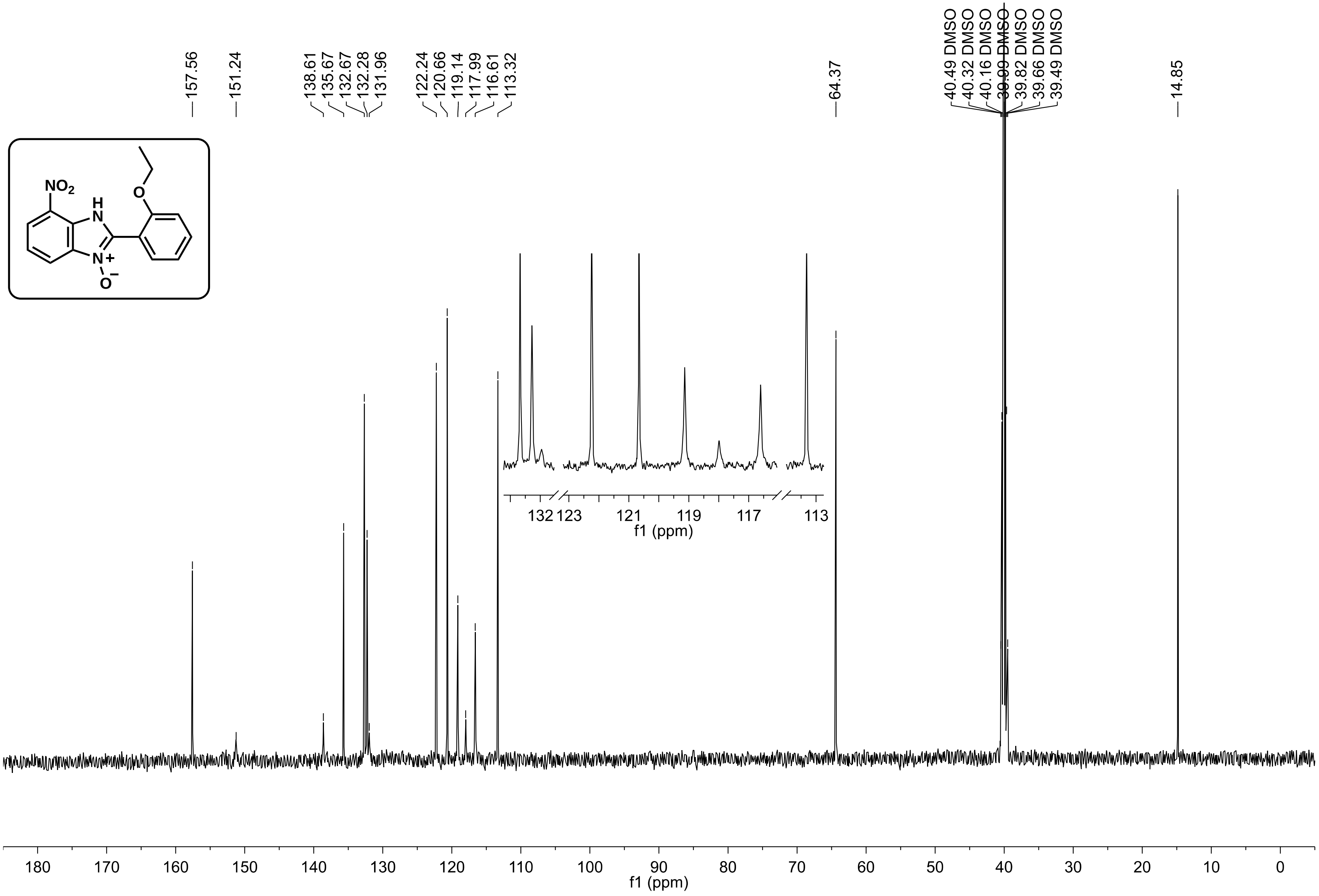

^{13}C-NMR (101 MHz $(CD_3)_2SO$). **2-(3-chlorophenyl)-7-nitro -1*H*-benzimidazole 3-oxide (4ae)**

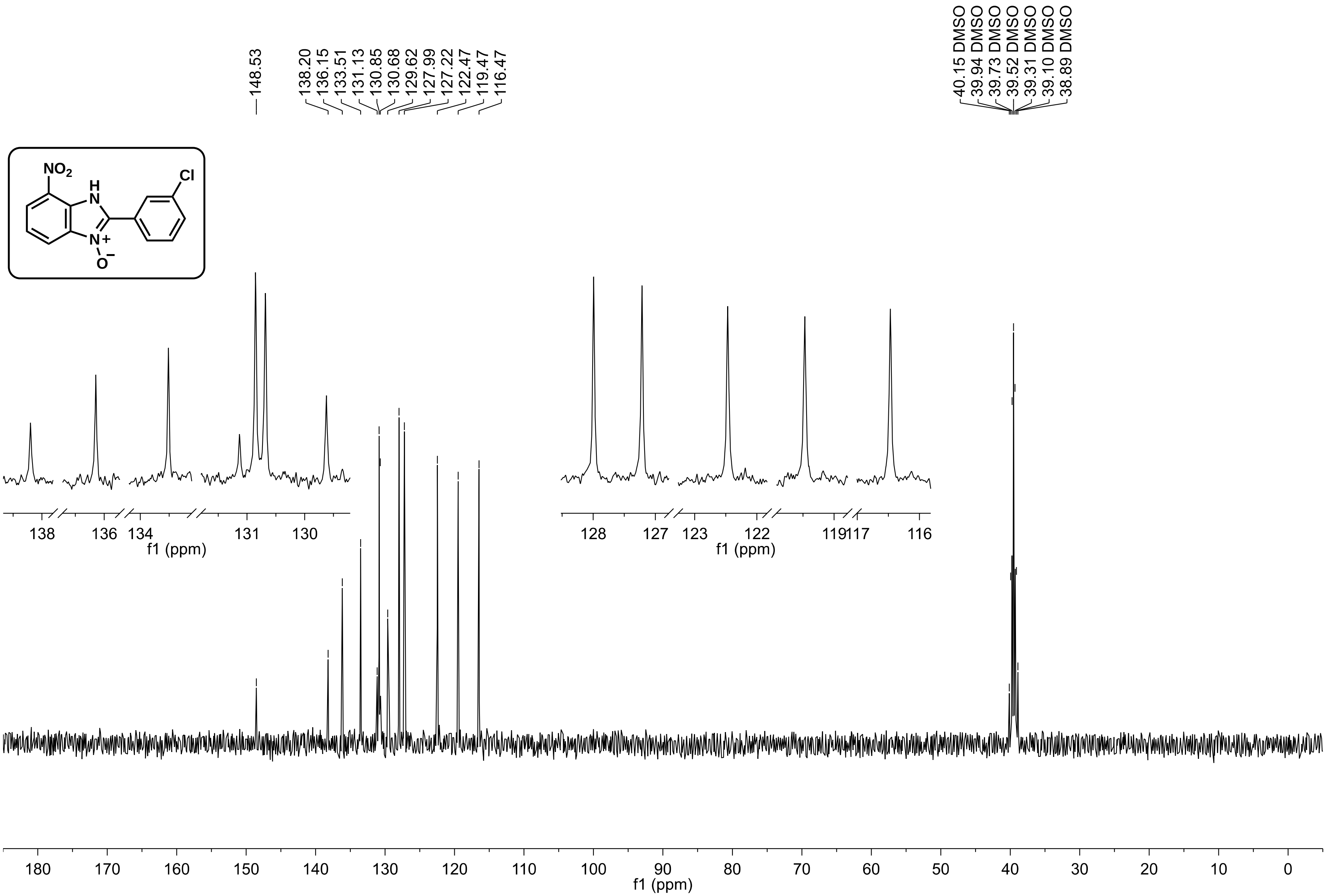

^{13}C-NMR (75 MHz $(CD_3)_2SO$). **2-(3-methoxyphenyl)-7-nitro-1*H*-benzimidazole 3-oxide (4af)**

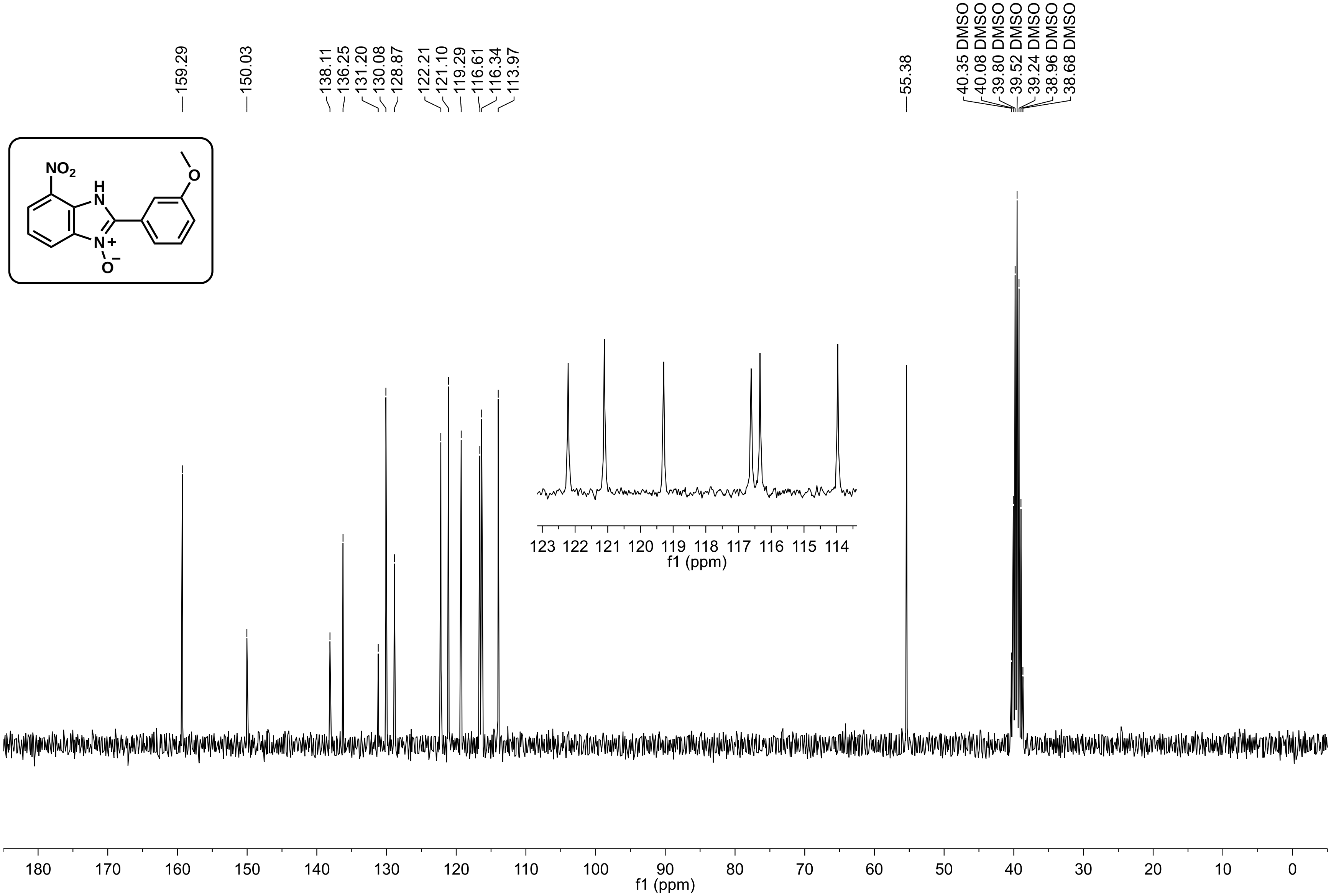

^{13}C-NMR (101 MHz $(CD_3)_2SO$). **7-nitro-2-(*p*-tolyl)-1*H*-benzimidazole 3-oxide (4ag)**

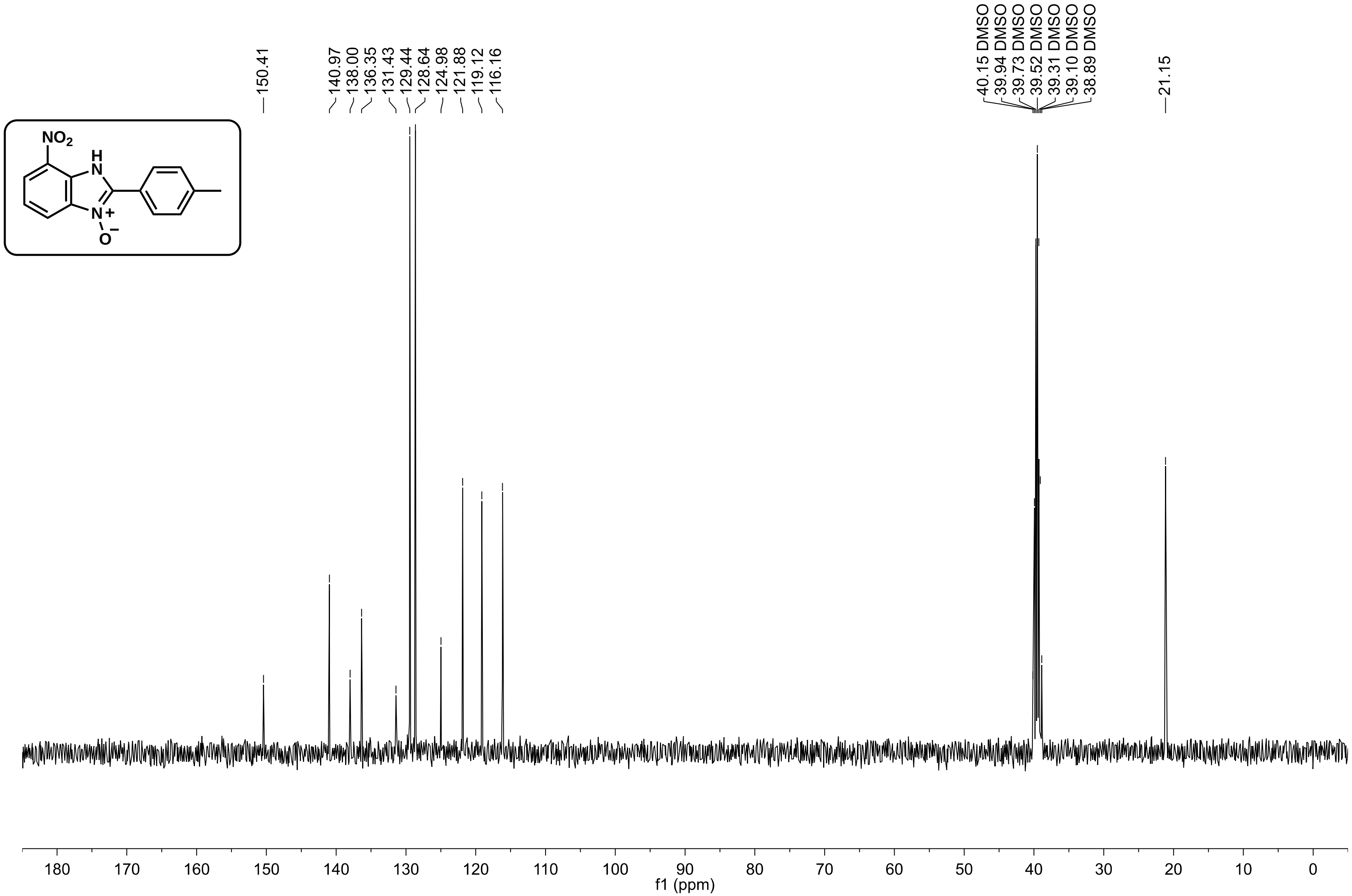

^{13}C-NMR (101 MHz $(CD_3)_2SO$). **2-(4-fluorophenyl)-7-nitro-1*H*-benzimidazole 3-oxide (4ah)**

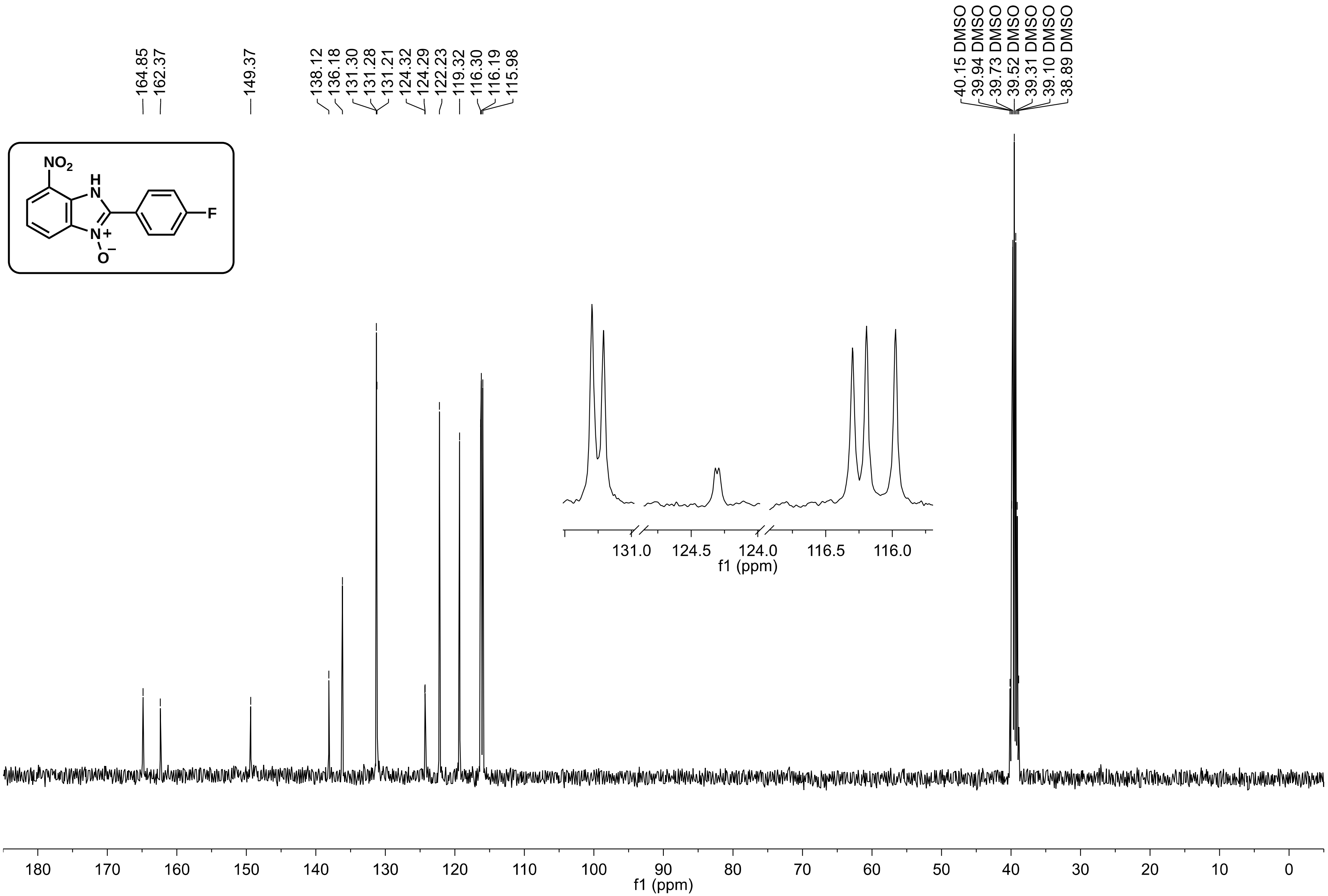

^{13}C-NMR (101 MHz $(CD_3)_2SO$). **2-(4-chlorophenyl)-7-nitro-1*H*-benzimidazole 3-oxide (4ai)**

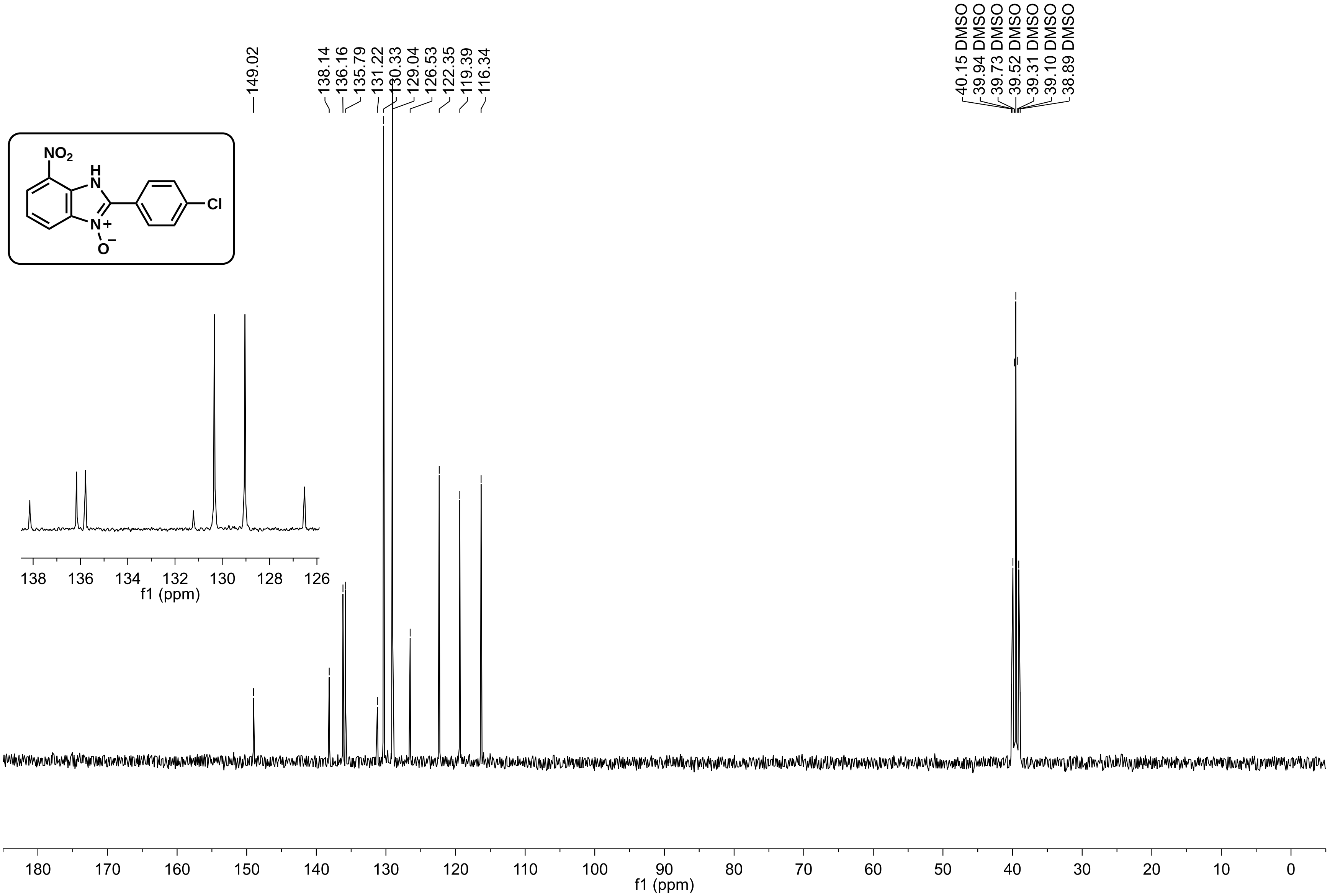

^{13}C-NMR (101 MHz $(CD_3)_2SO$). **2-(4-methoxyphenyl)-7-nitro-1*H*-benzimidazole 3-oxide (4aj)**

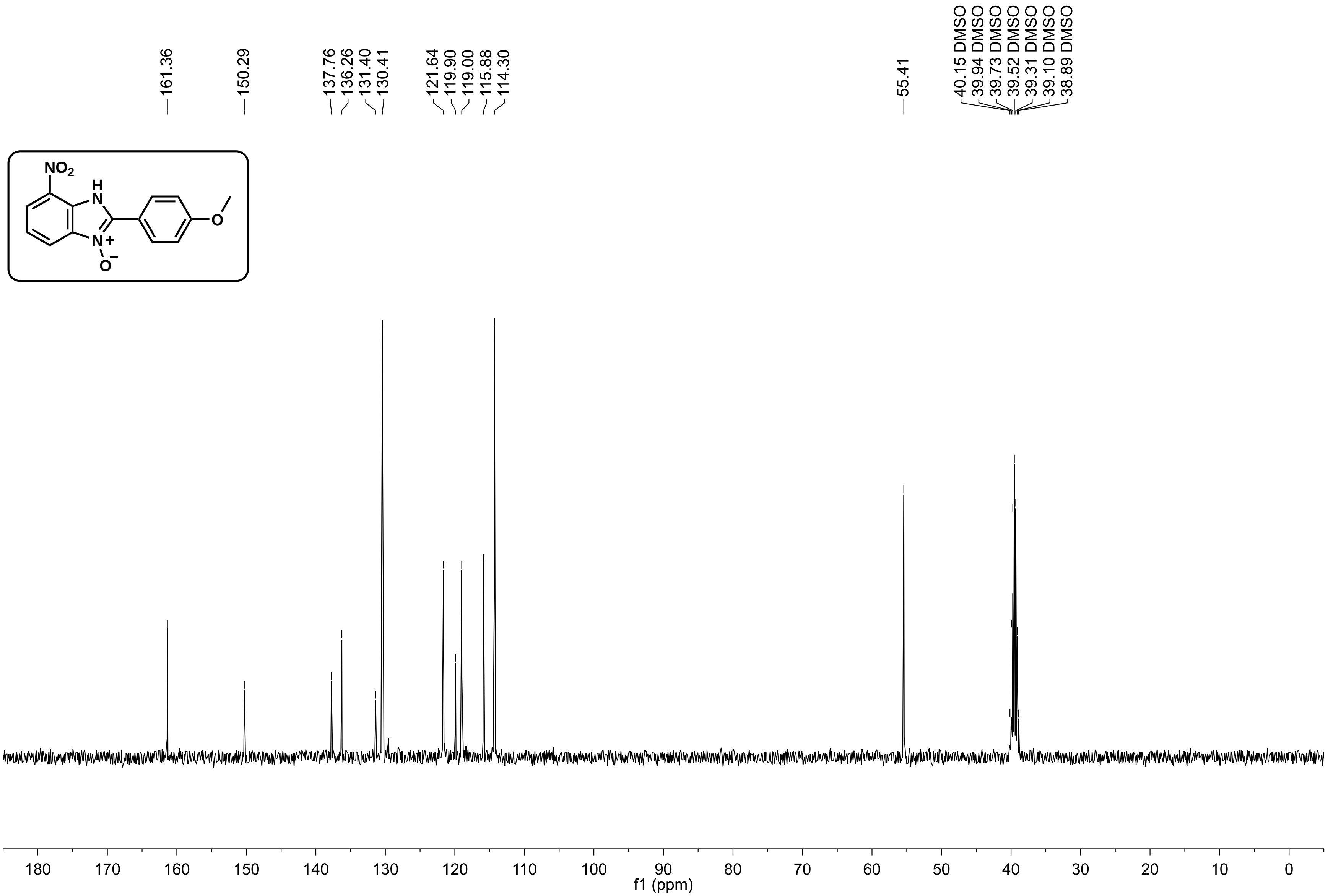

^{13}C-NMR (126 MHz $(CD_3)_2SO$). **2-(furan-2-yl)-7-nitro-1*H*-benzimidazole 3-oxide (4ak)**

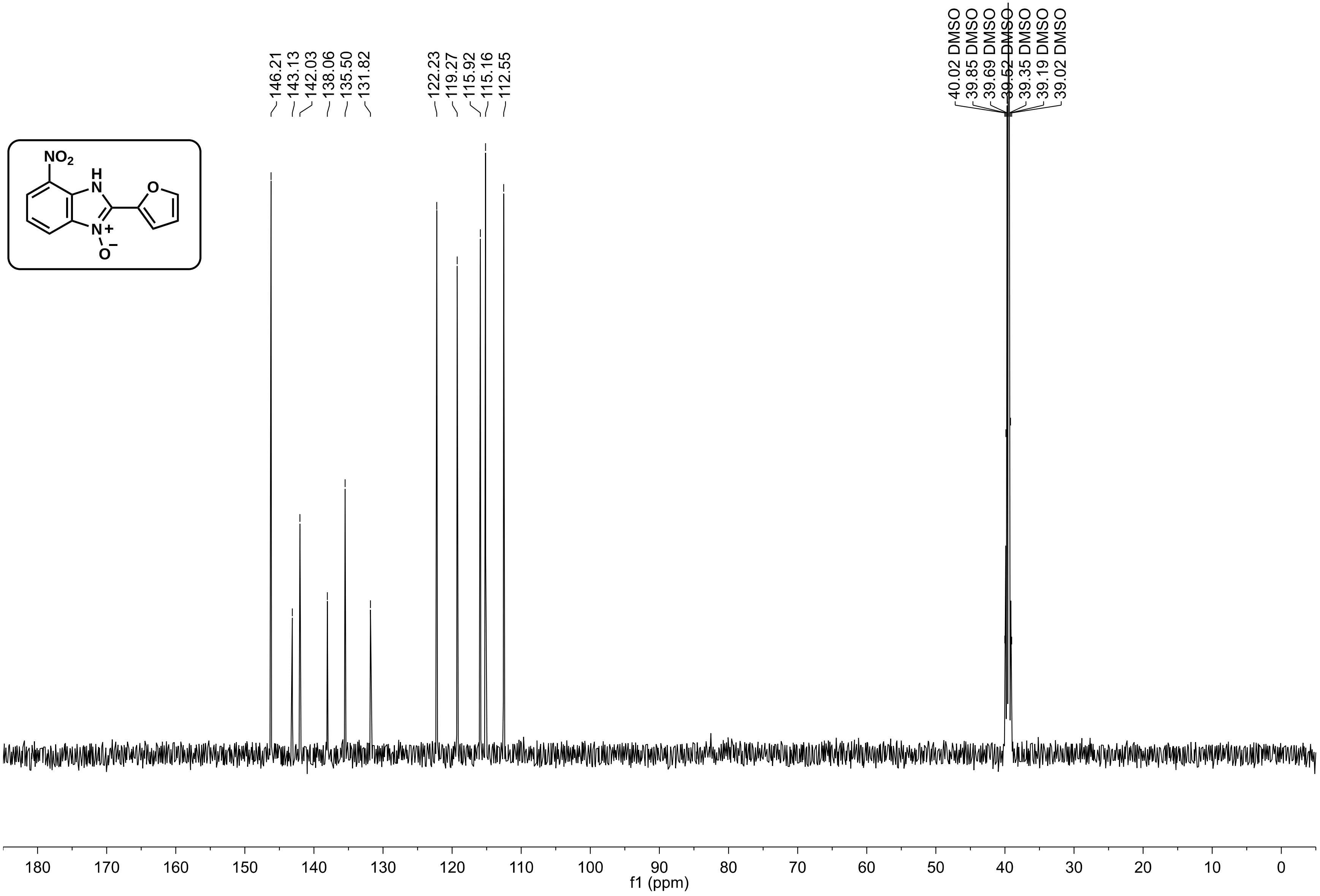

^{13}C-NMR (101 MHz $(CD_3)_2SO$). **7-nitro-2-(thiophen-2-yl)-1H-benzimidazole 3-oxide (4al)**

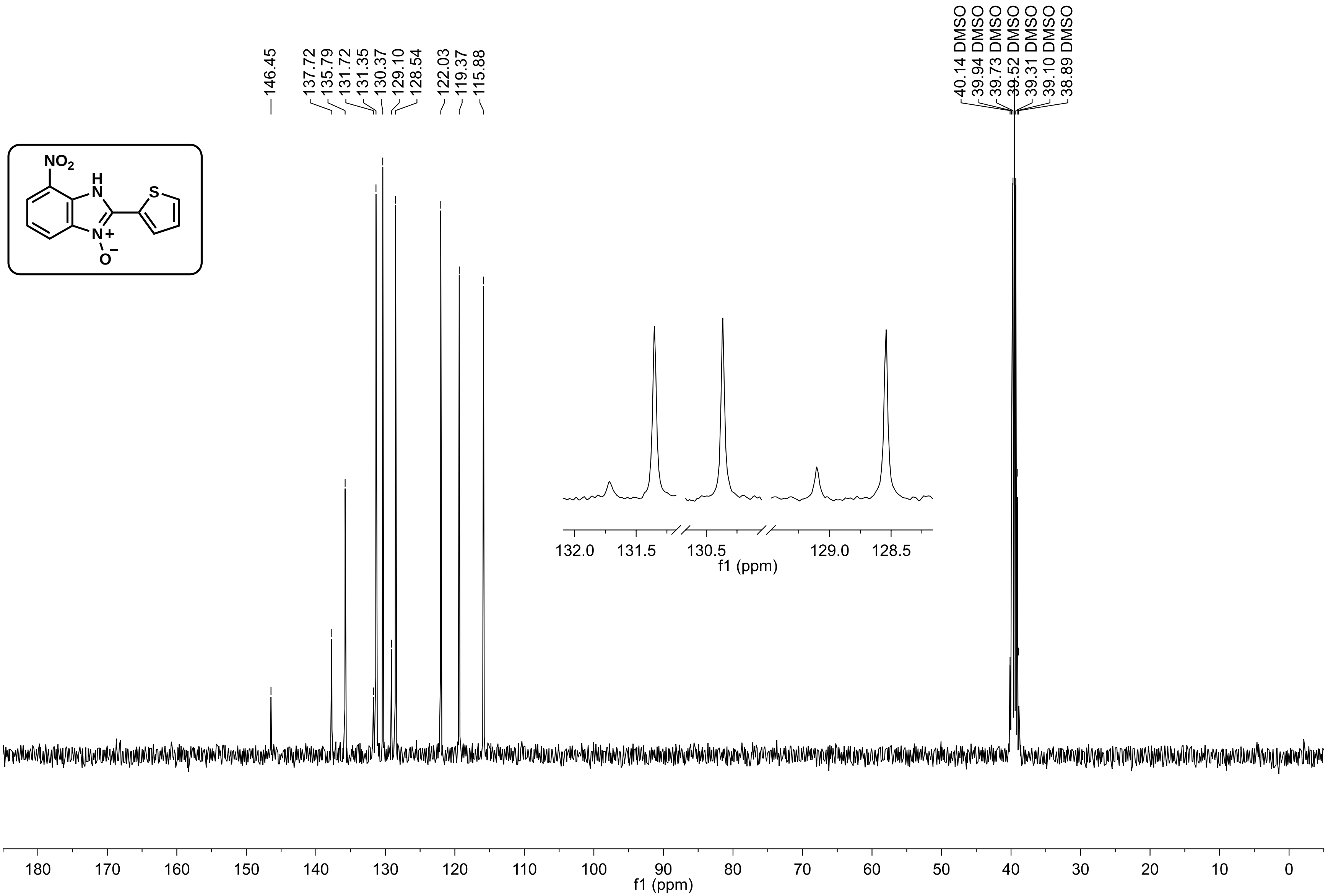

^{13}C-NMR (101 MHz $(CD_3)_2SO$). **7-nitro-2-(pyridin-3-yl)-1H-benzimidazole 3-oxide (4am)**

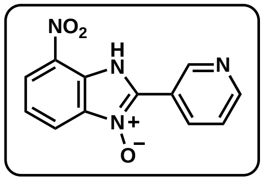

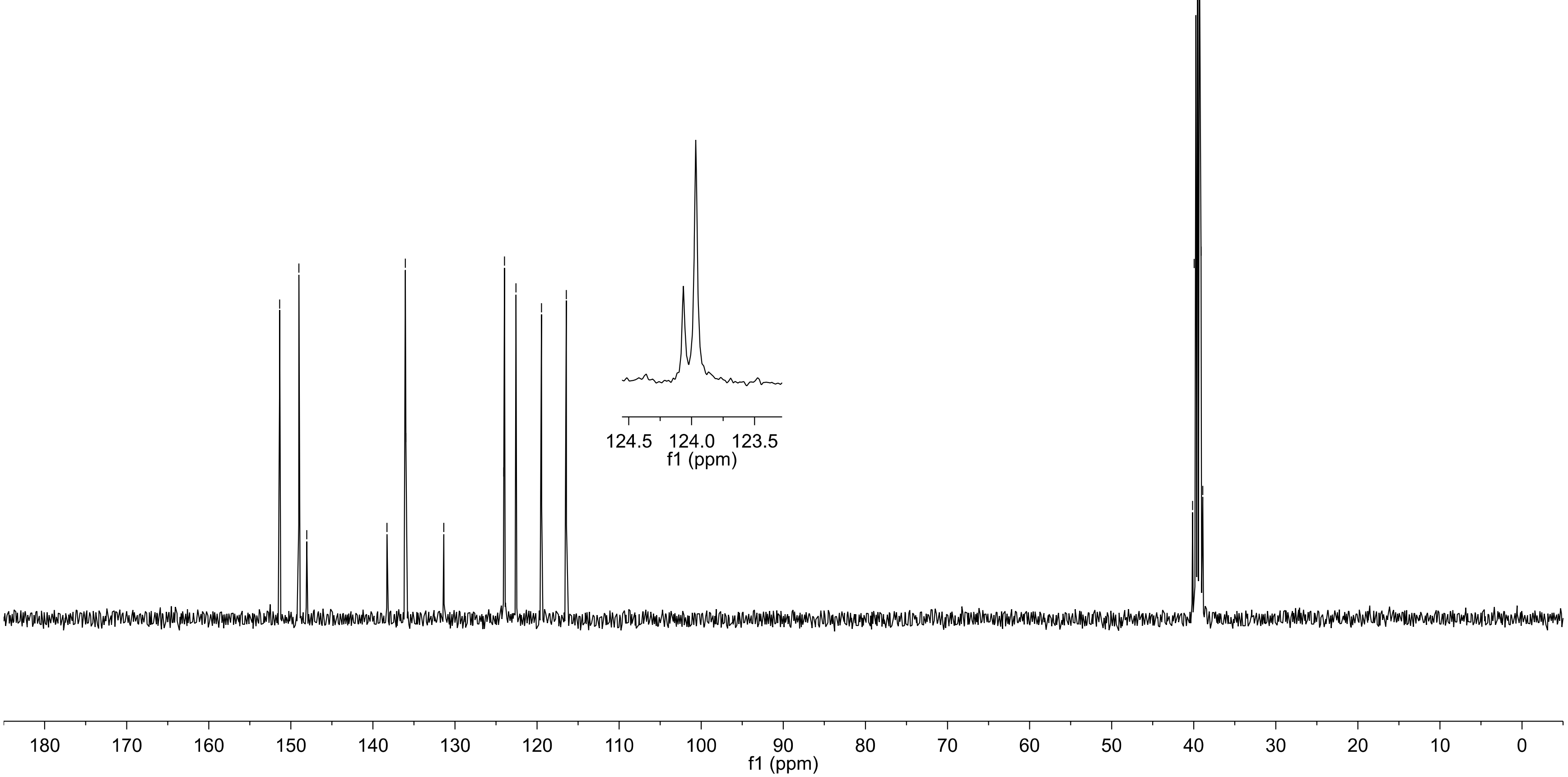

^{13}C-NMR (126 MHz $(CD_3)_2SO$). **2-benzyl-7-nitro-1H-benzimidazole 3-oxide (4an)**

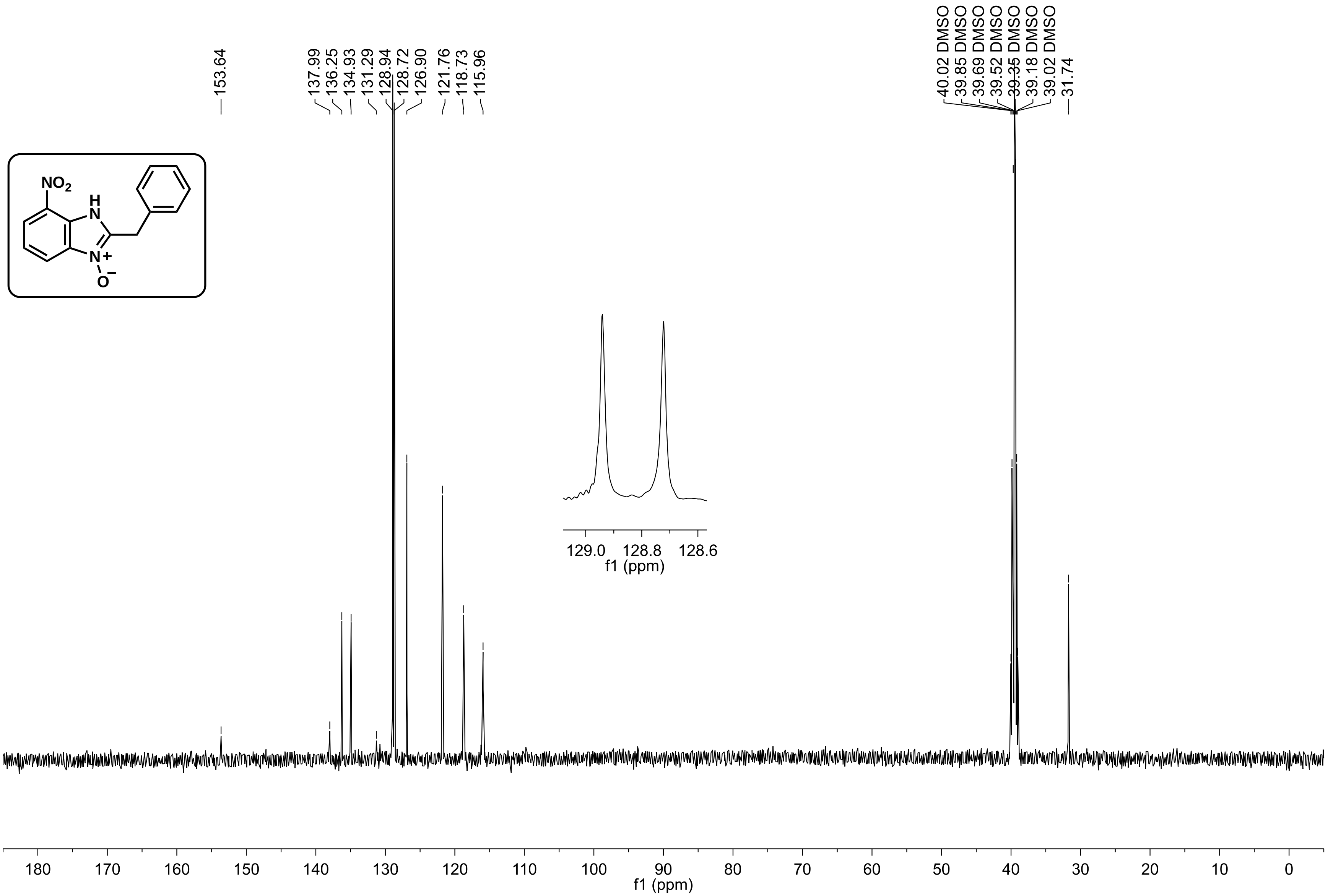

^{13}C-NMR (126 MHz $(CD_3)_2SO$). **7-nitro-2-phenyl-5-trifluoromethyl-1*H*-benzimidazole 3-oxide (4ba)**

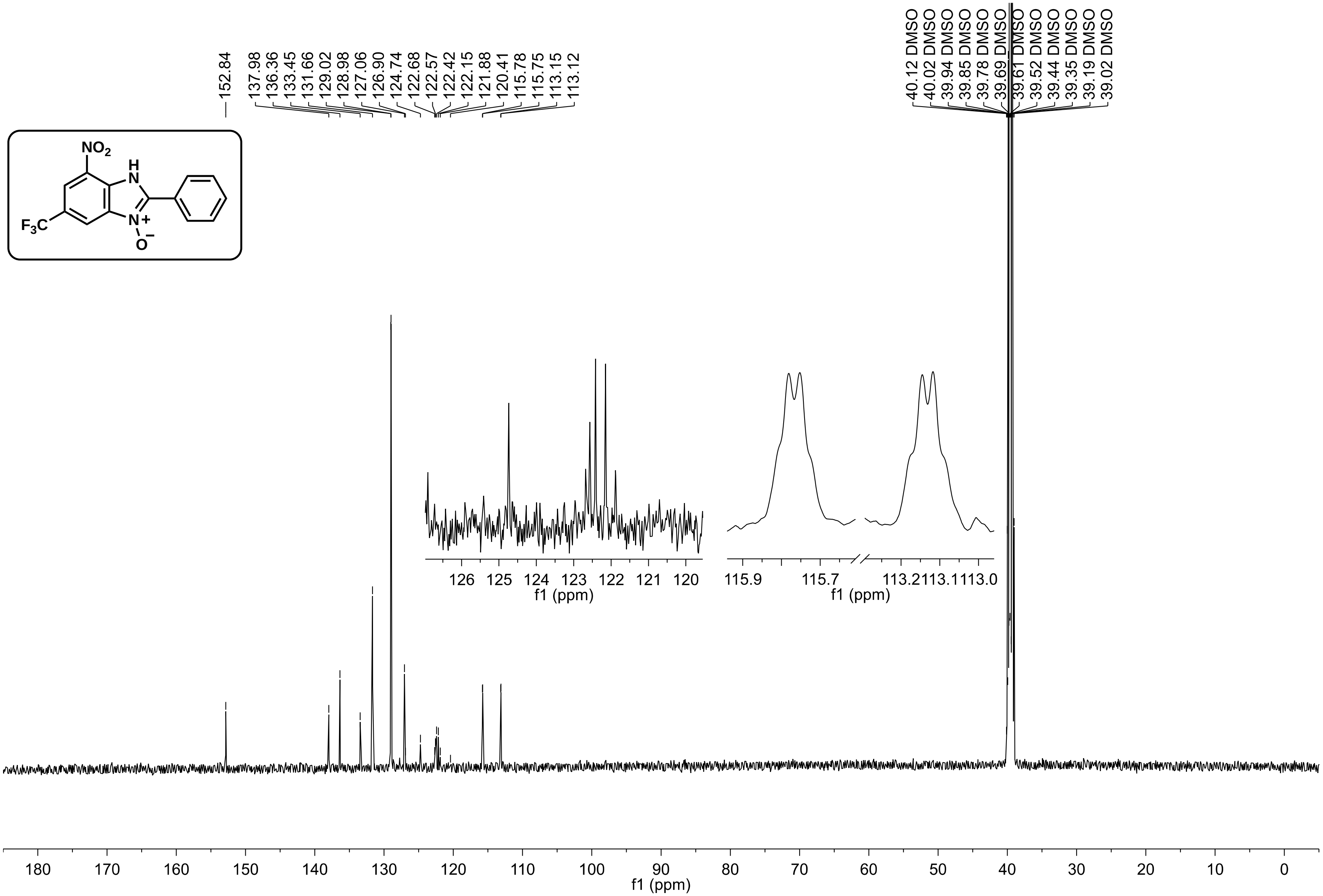

^{13}C-NMR (126 MHz $(CD_3)_2SO$). **7-nitro-2-(*o*-tolyl)-5-trifluoromethyl-1*H*-benzimidazole 3-oxide (4bb)**

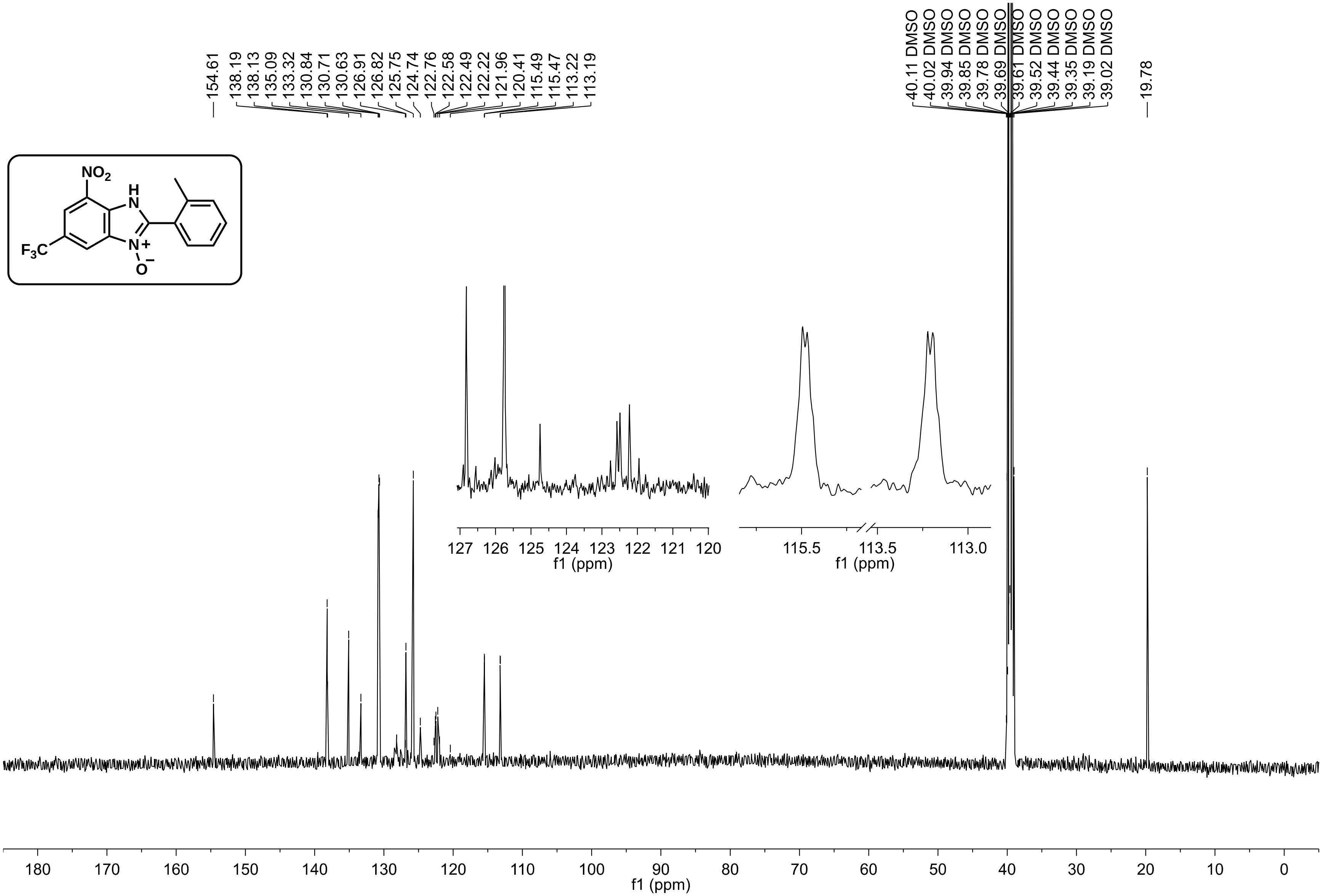

^{13}C-NMR (126 MHz $(CD_3)_2SO$). **2-(2-fluorophenyl)-7-nitro-5-trifluoromethyl-1*H*-benzimidazole 3-oxide (4bc)**

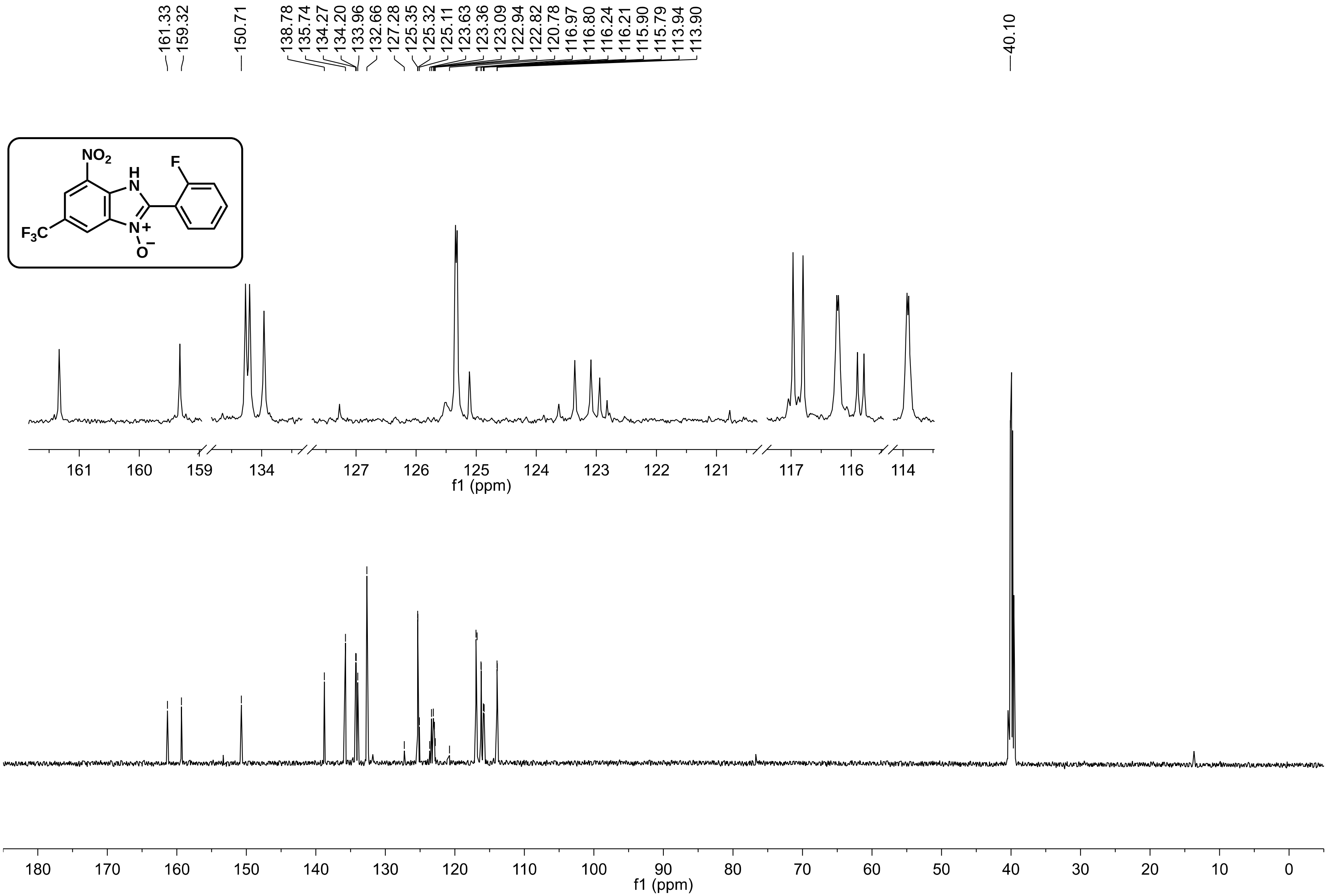

^{13}C-NMR (101 MHz $(CD_3)_2SO$). **2-(2-ethoxyphenyl)-7-nitro-5-trifluoromethyl-1*H*-benzimidazole 3-oxide (4bd)**

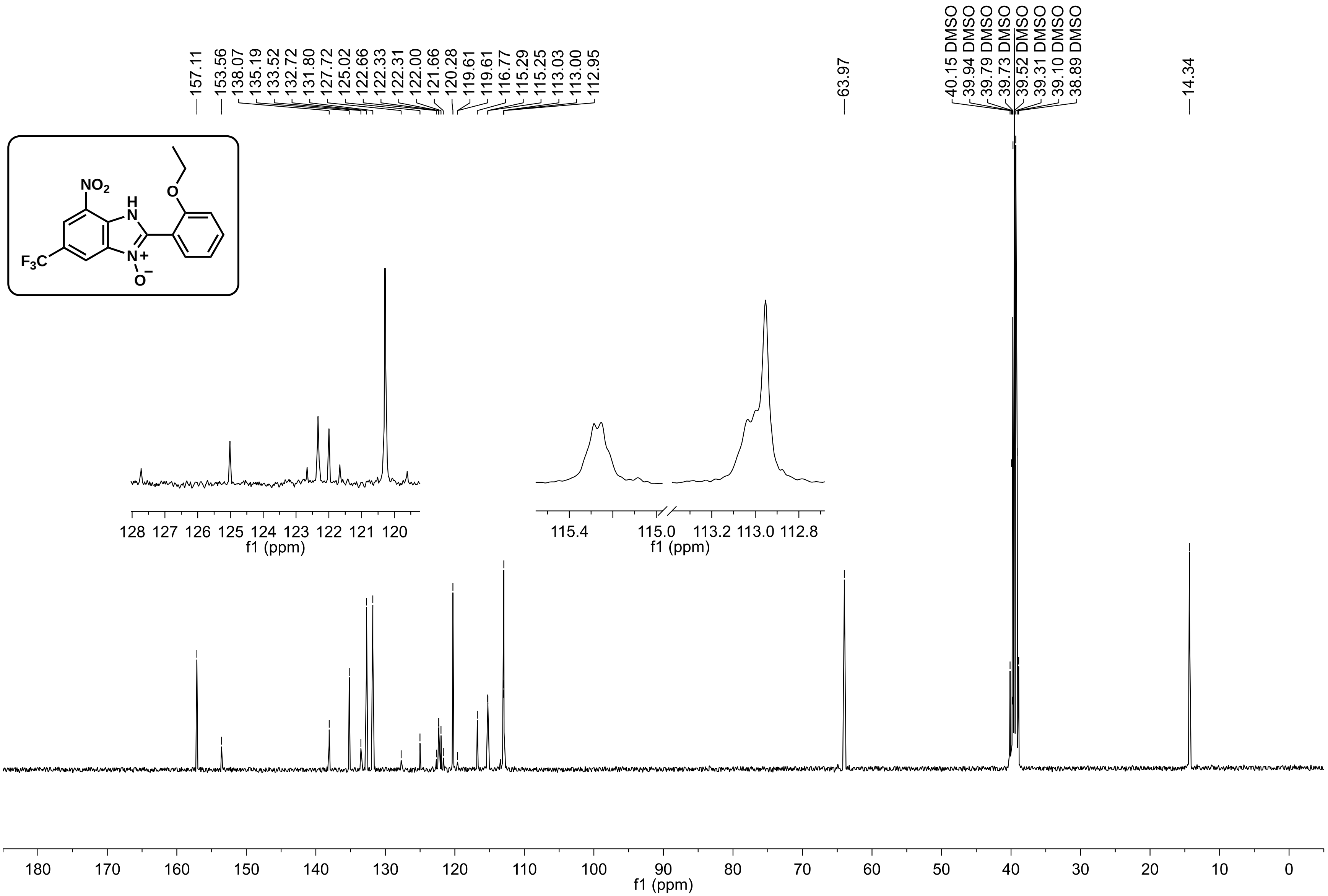

^{13}C-NMR (126 MHz $(CD_3)_2SO$). **2-(3-chlorophenyl)-7-nitro-5-trifluoromethyl-1*H*-benzimidazole 3-oxide (4be)**

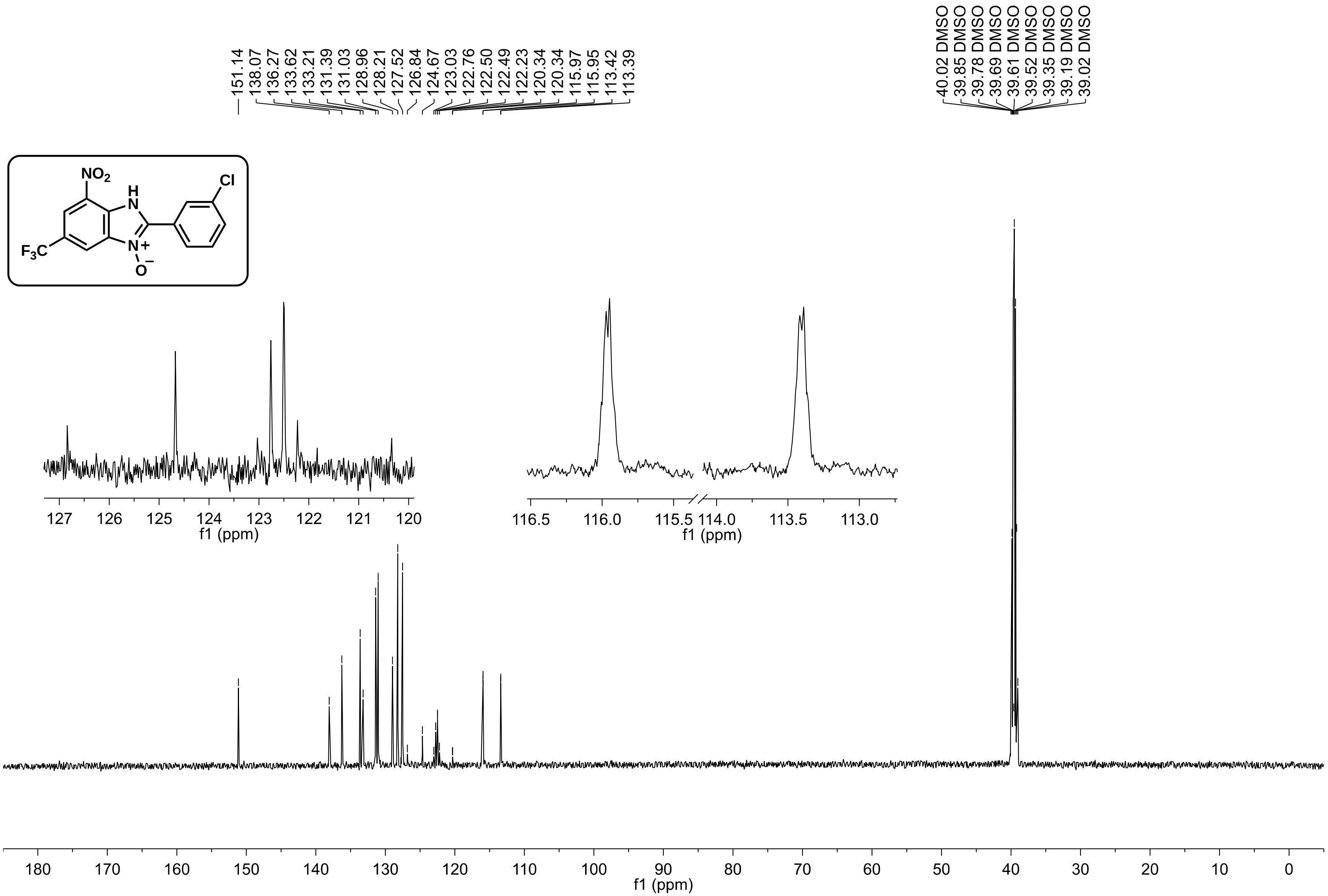

^{13}C-NMR (126 MHz $(CD_3)_2SO$). **2-(3-methoxyphenyl)-7-nitro-5-trifluoromethyl-1*H*-benzimidazole 3-oxide (4bf)**

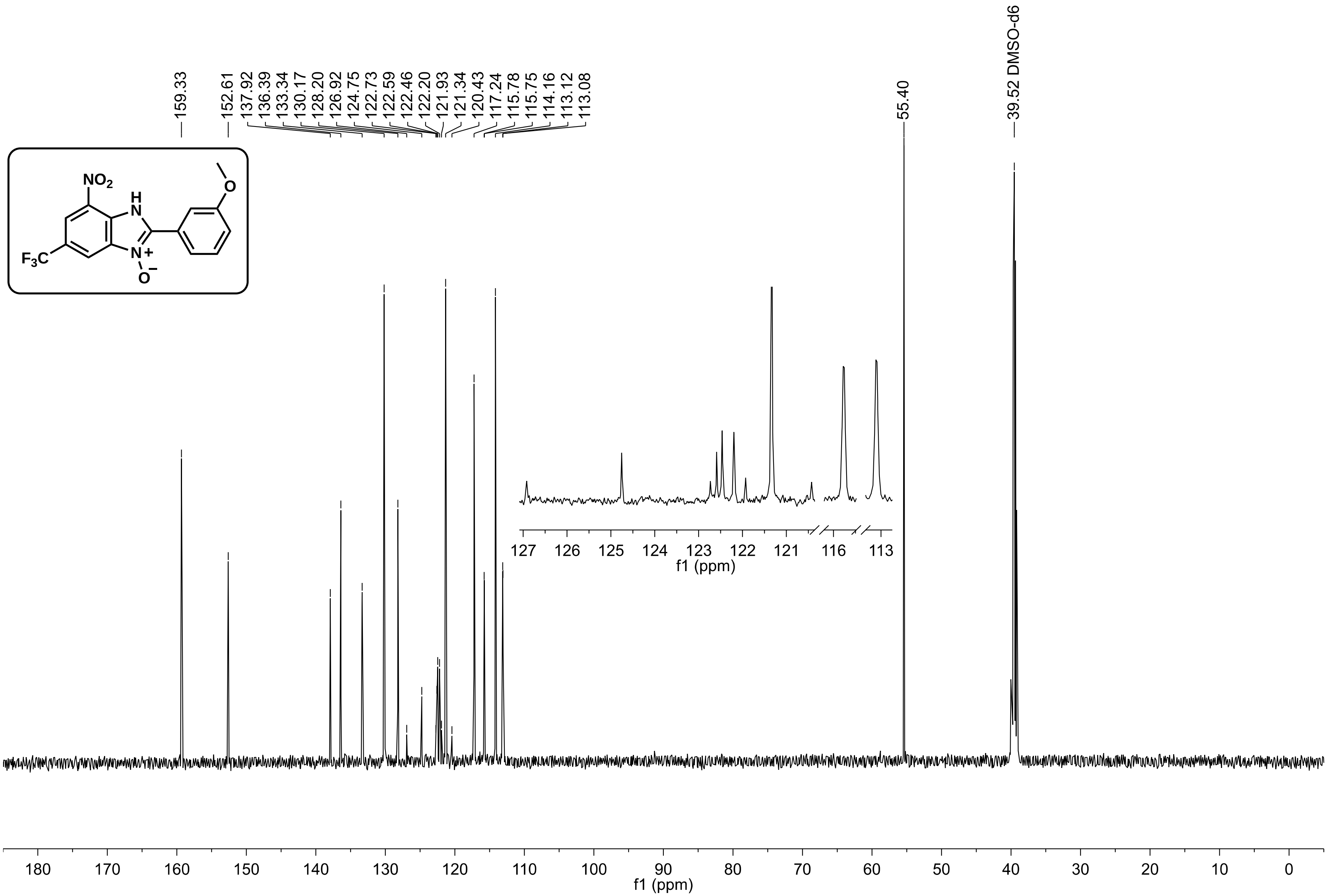

^{13}C-NMR (126 MHz $(CD_3)_2SO$). **7-nitro-2-(*p*-tolyl)-5-trifluoromethyl-1*H*-benzimidazole 3-oxide (4bg)**

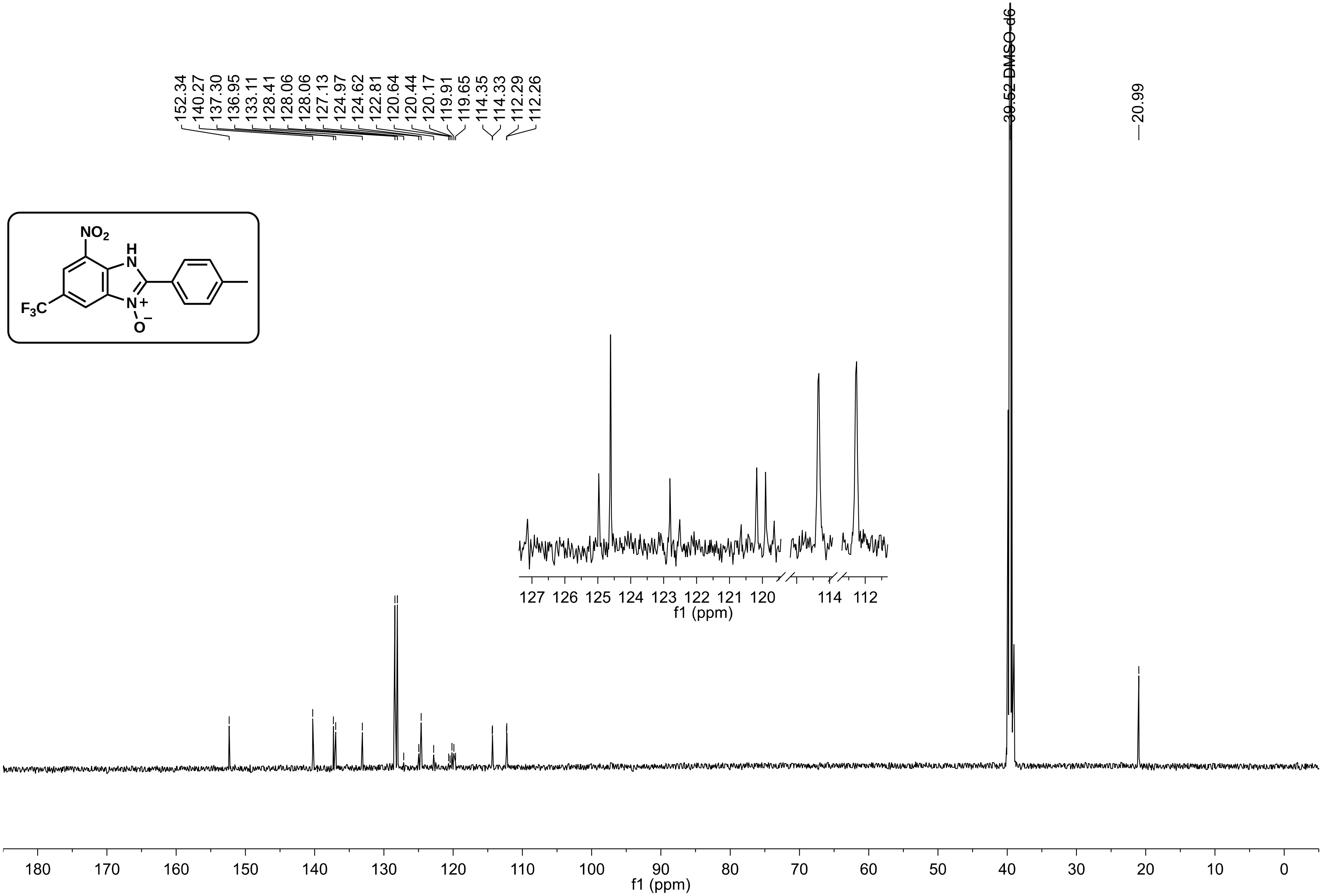

^{13}C-NMR (126 MHz $(CD_3)_2SO$). **2-(4-fluorophenyl)-7-nitro-5-trifluoromethyl-1*H*-benzimidazole 3-oxide (4bh)**

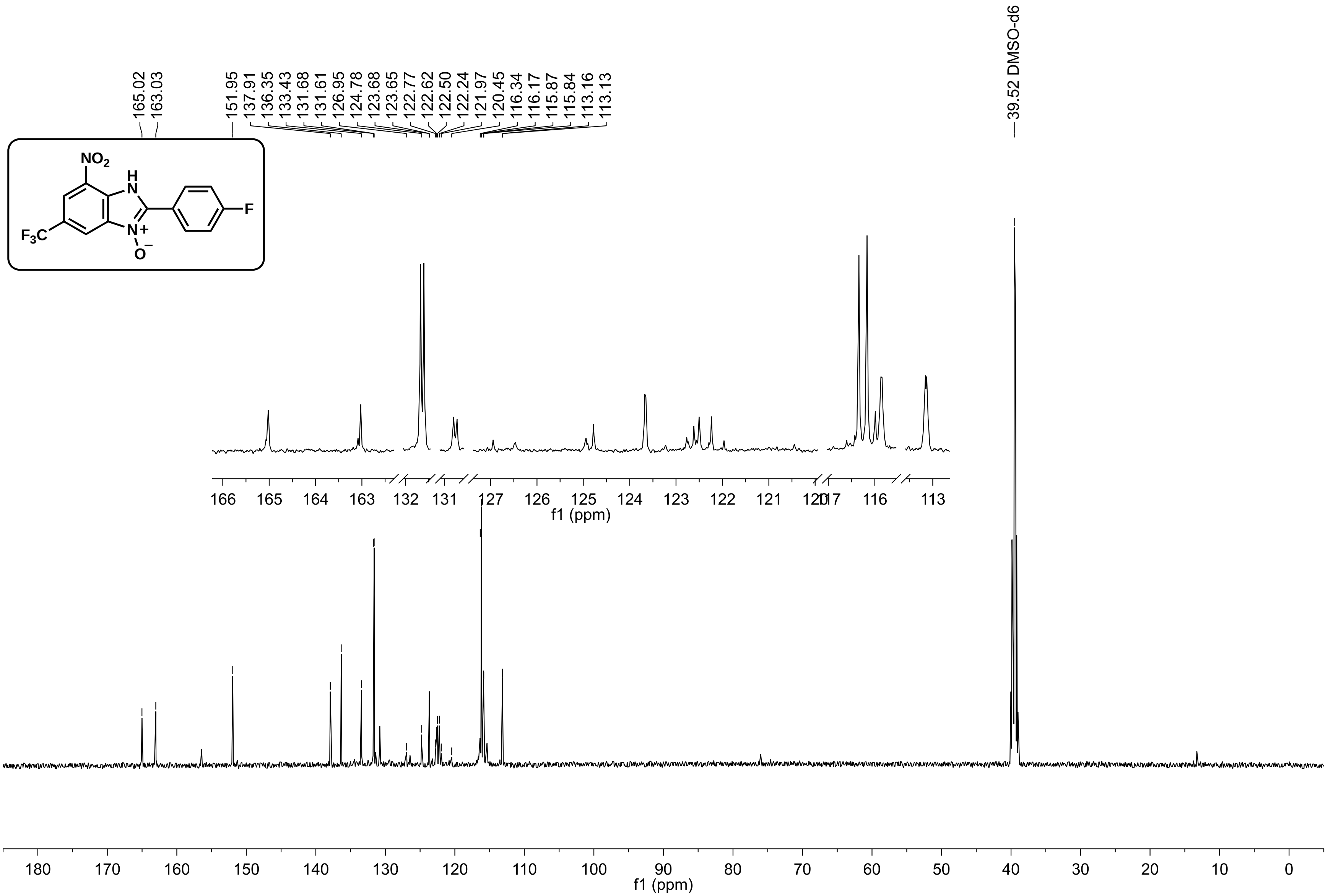

^{13}C-NMR (126 MHz $(CD_3)_2SO$). **2-(4-chlorophenyl)-7-nitro-5-trifluoromethyl-1*H*-benzimidazole 3-oxide (4bi)**

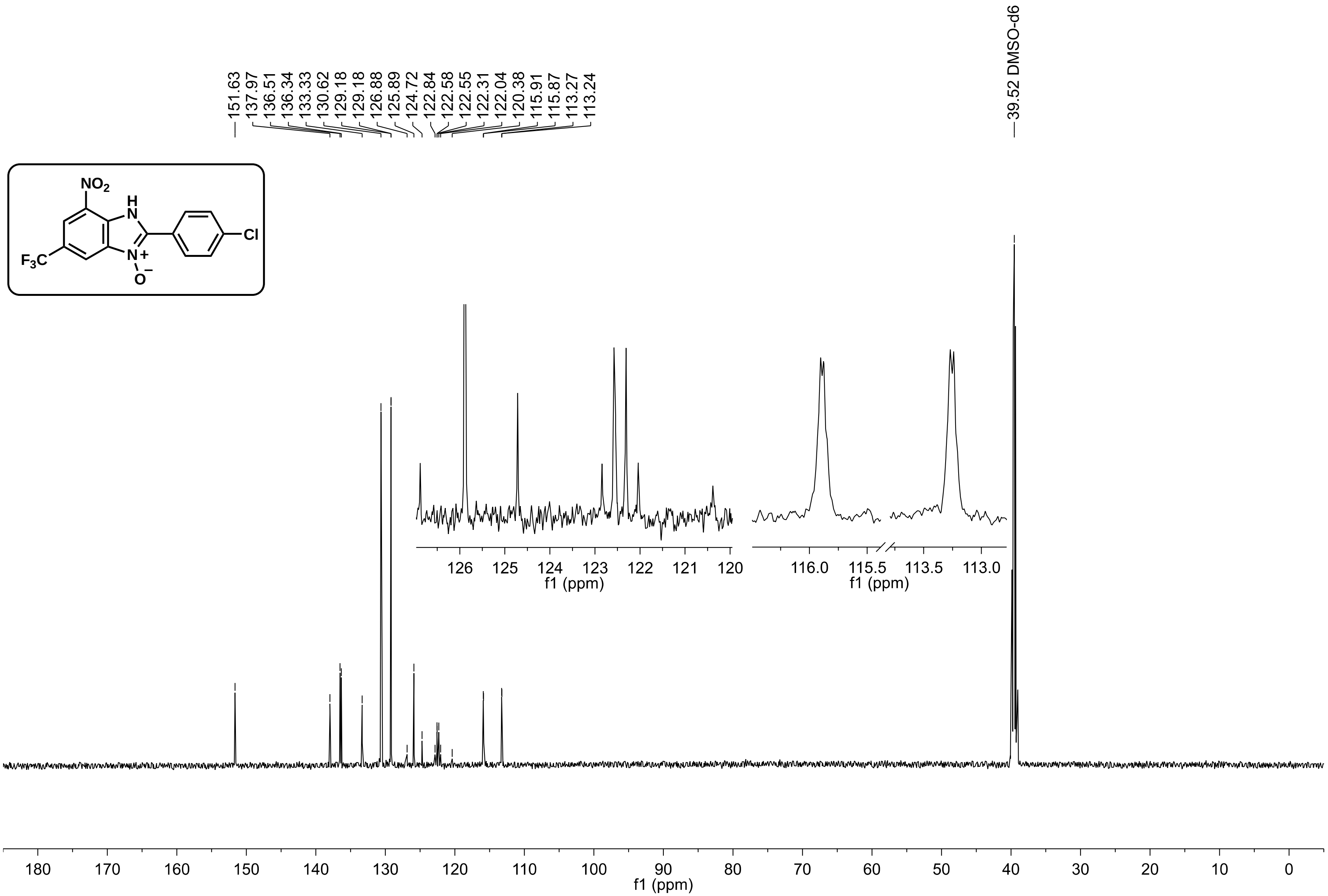

^{13}C-NMR (126 MHz $(CD_3)_2SO$). **2-(4-methoxyphenyl)-7-nitro-5-trifluoromethyl-1*H*-benzimidazole 3-oxide (4bj)**

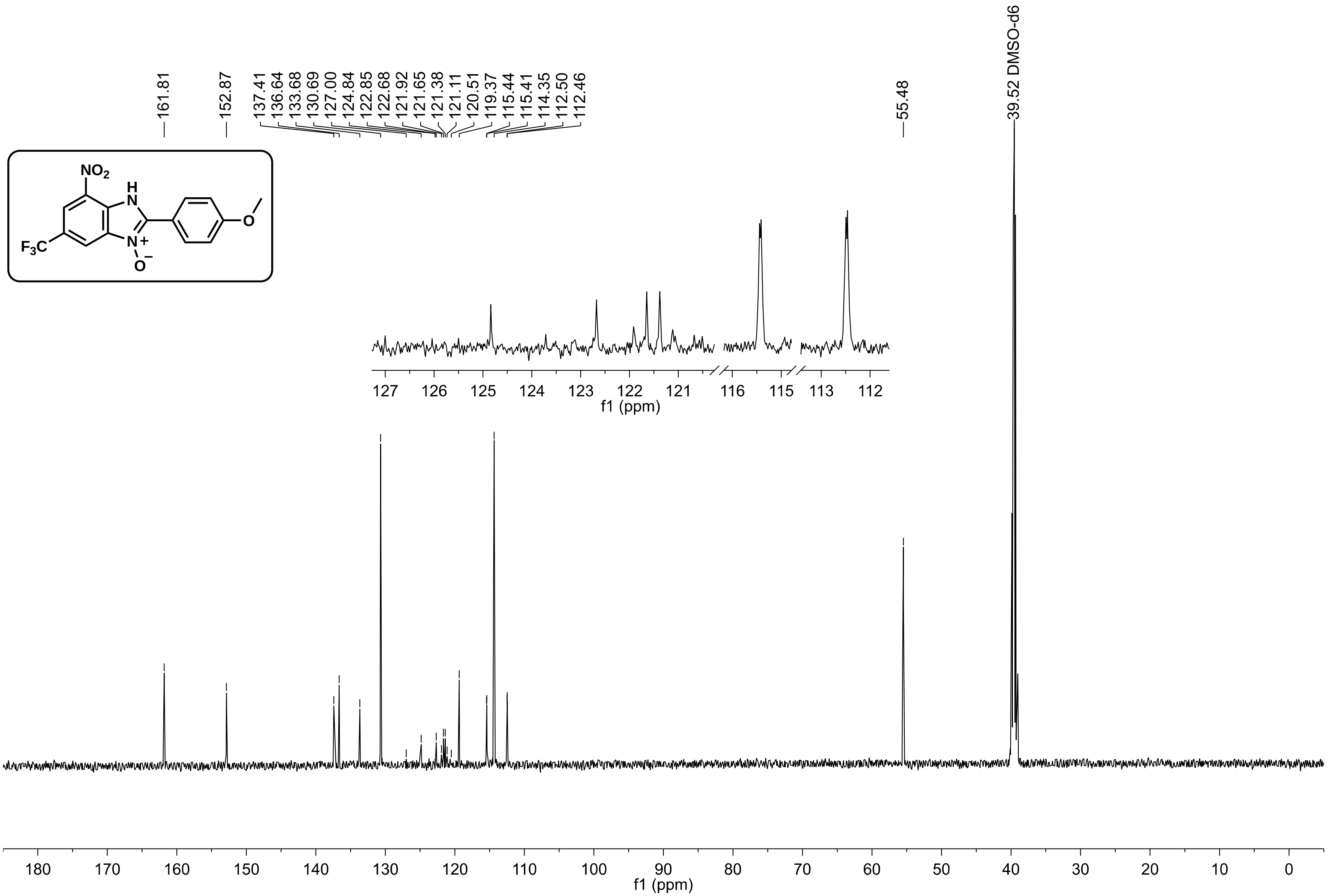

^{13}C-NMR (126 MHz $(CD_3)_2SO$). **2-(furan-2-yl)-7-nitro-5-trifluoromethyl-1*H*-benzimidazole 3-oxide (4bk)**

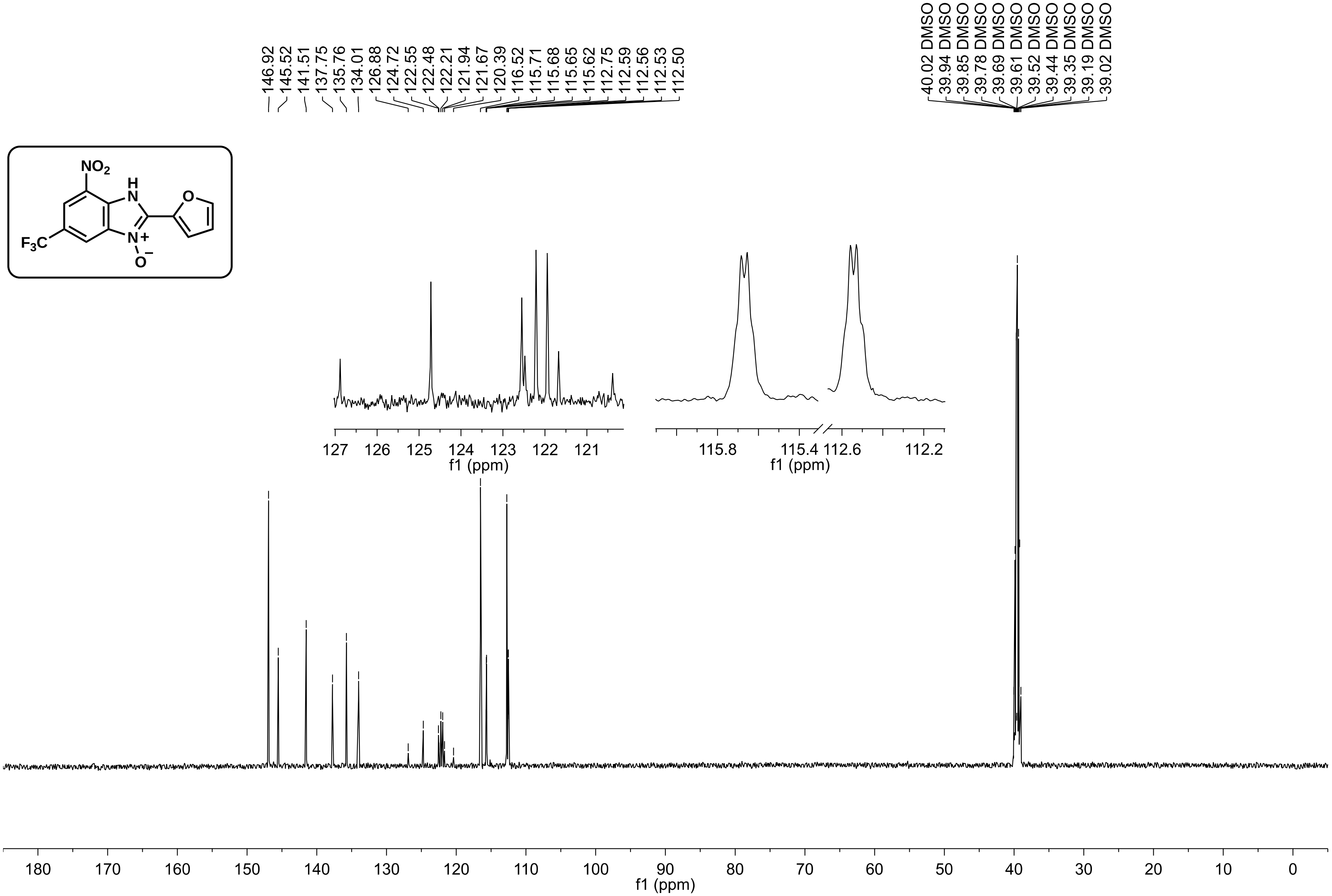

^{13}C-NMR (101 MHz $(CD_3)_2SO$). **7-nitro-2-(thiophen-2-yl)-5-trifluoromethyl-1*H*-benzimidazole 3-oxide (4bl)**

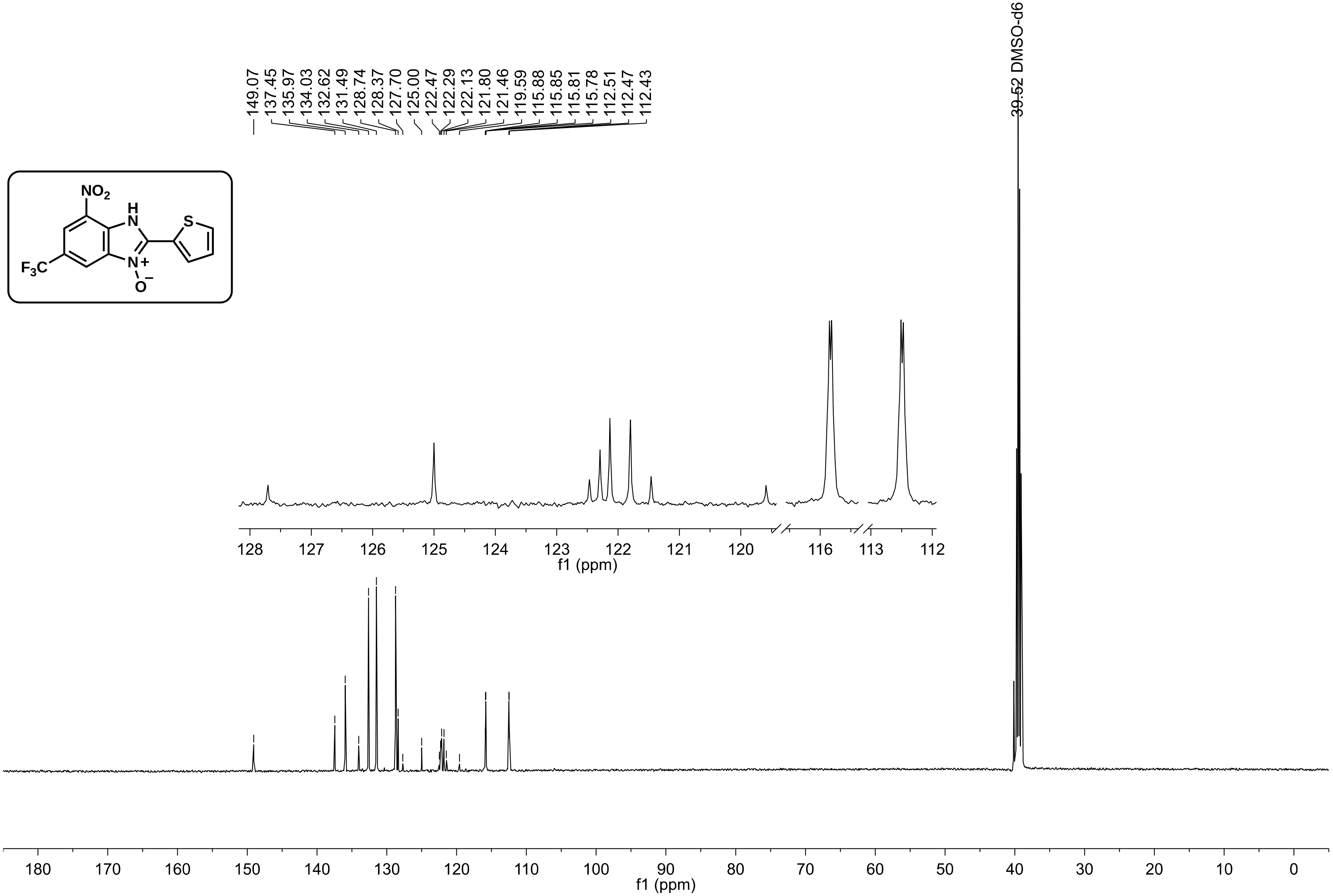

^{13}C-NMR (126 MHz $(CD_3)_2SO$). **7-nitro-2-(pyridin-3-yl)-5-trifluoromethyl-1*H*-benzimidazole 3-oxide (4bm)**

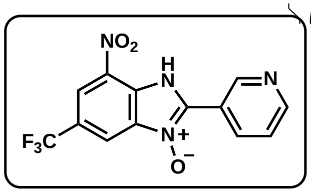

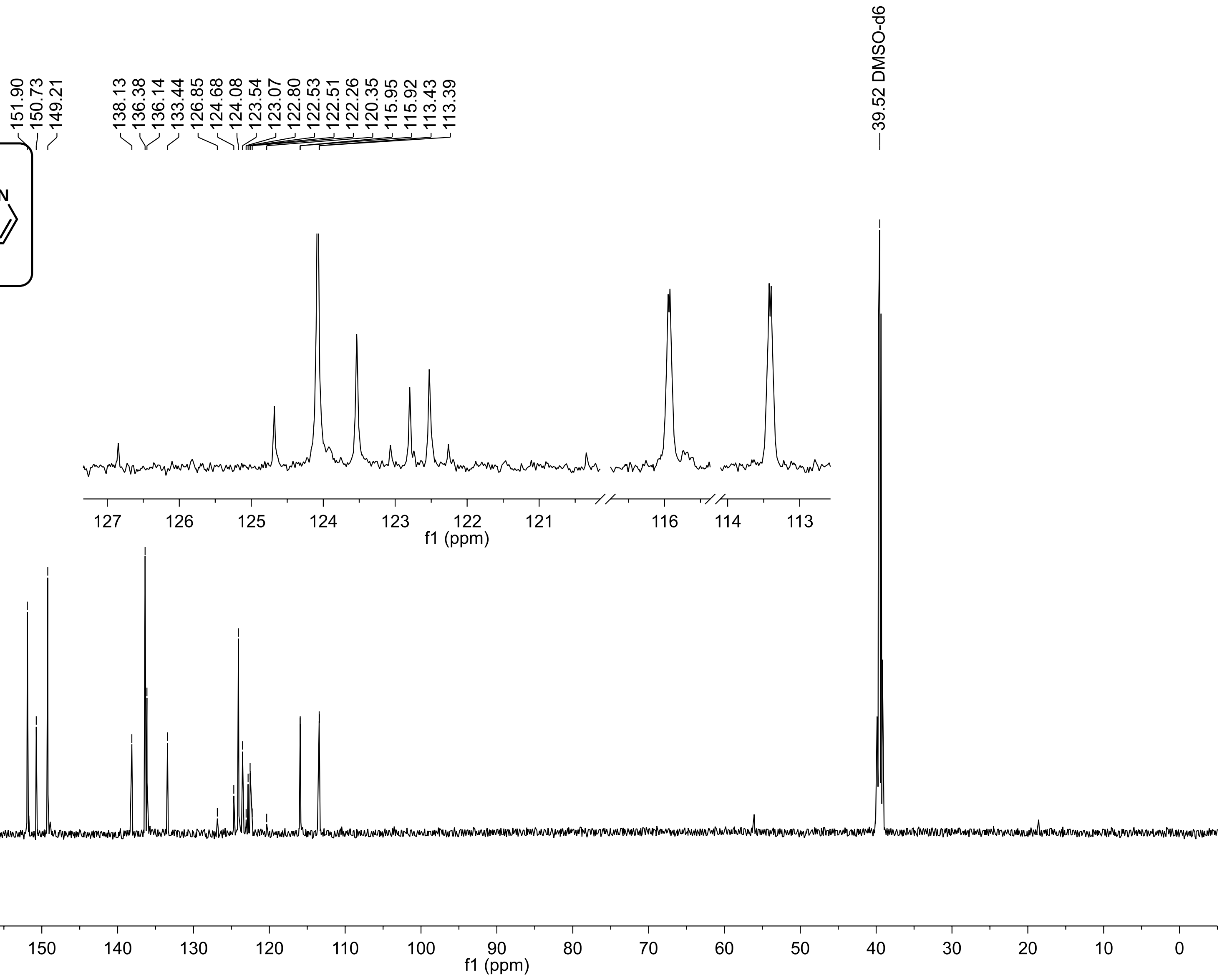

^{13}C-NMR (126 MHz $(CD_3)_2SO$). **2-benzyl-7-nitro-5-trifluoromethyl-1*H*-benzimidazole 3-oxide (4bn)**

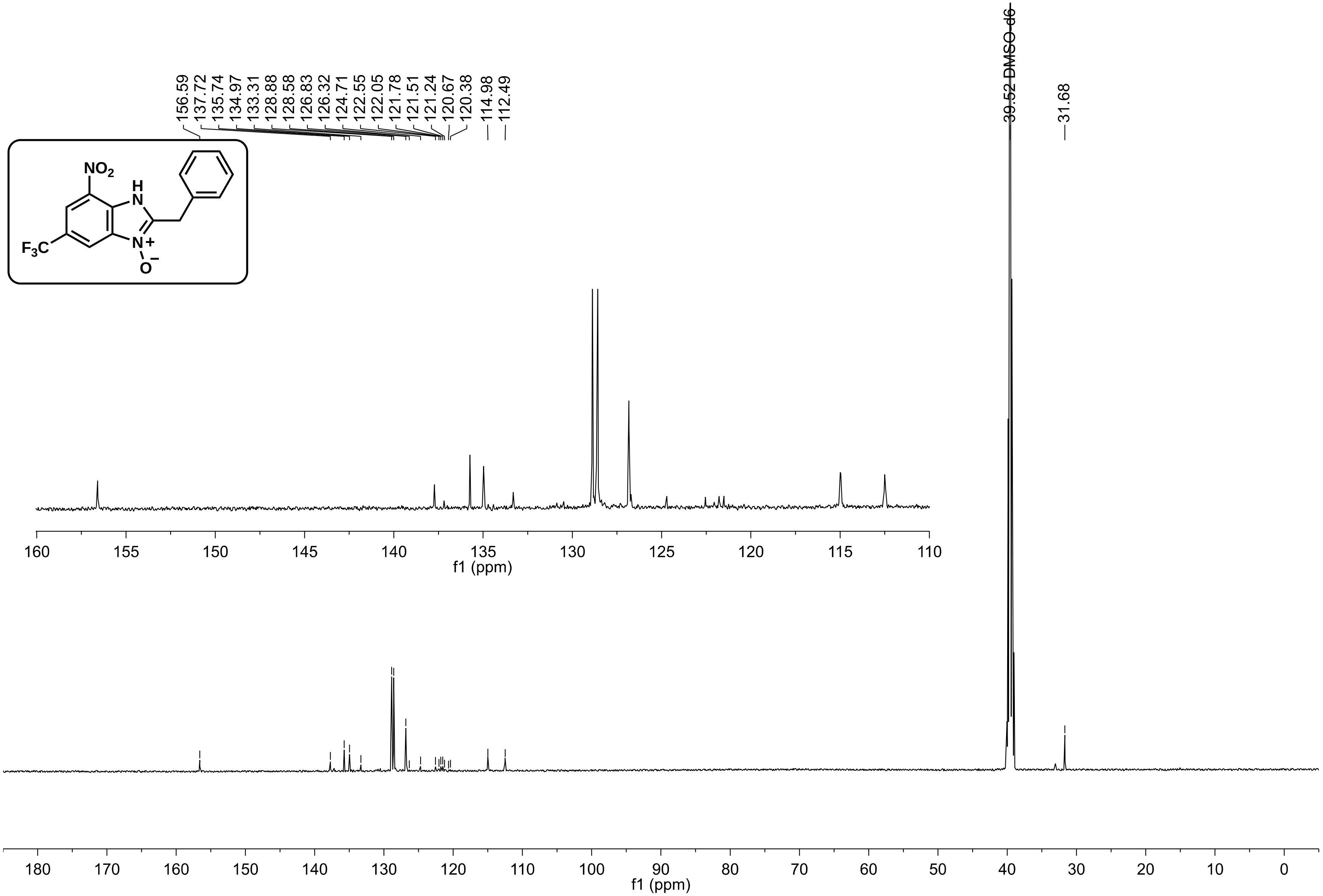

^{13}C-NMR (126 MHz $(CD_3)_2SO$). **5-nitro-2-phenyl-7-trifluoromethyl-1*H*-benzimidazole 3-oxide (4ca)**

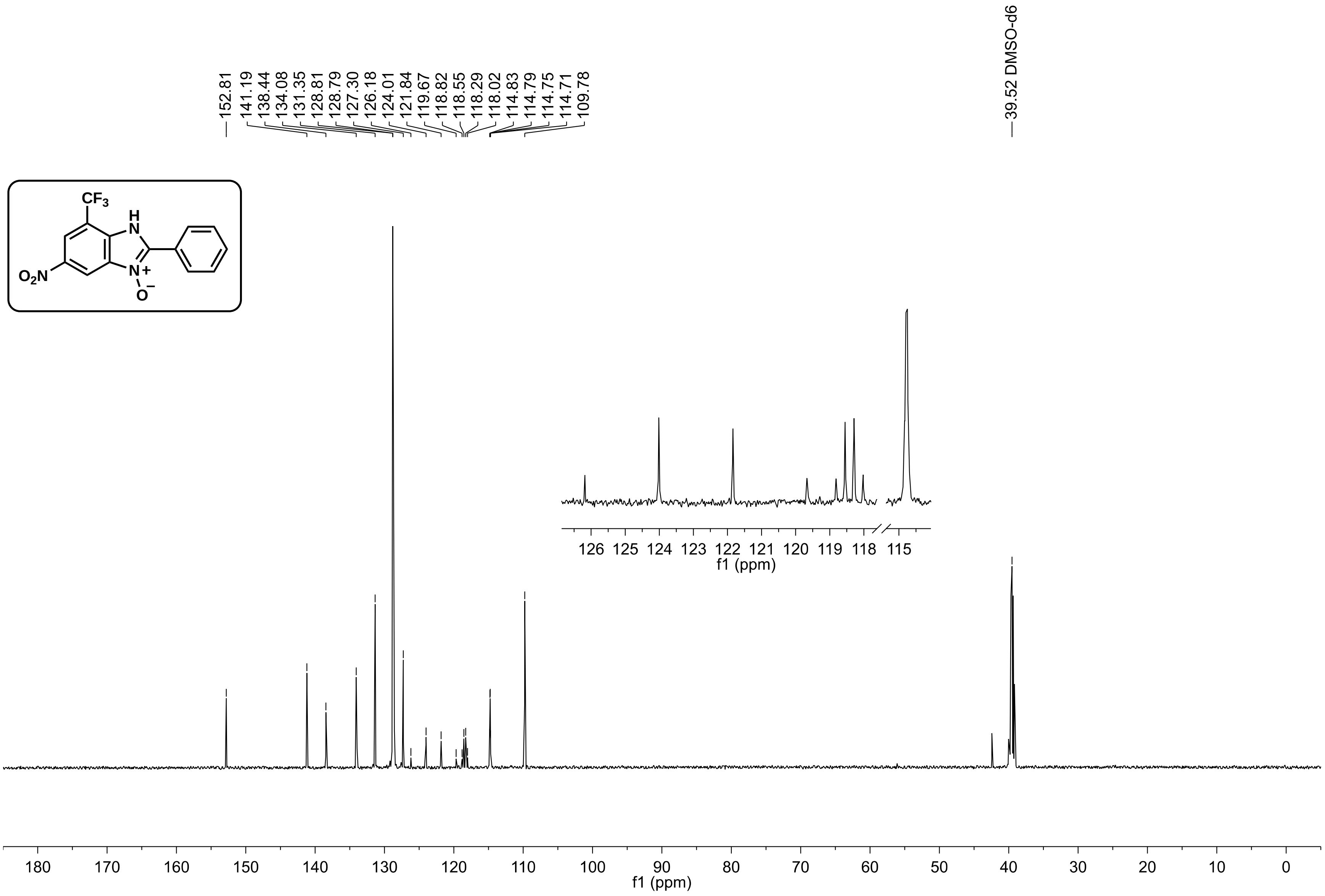

^{13}C-NMR (101 MHz $(CD_3)_2SO$). **5-nitro-2-(*o*-tolyl)-7-trifluoromethyl-1*H*-benzimidazole 3-oxide (4cb)**

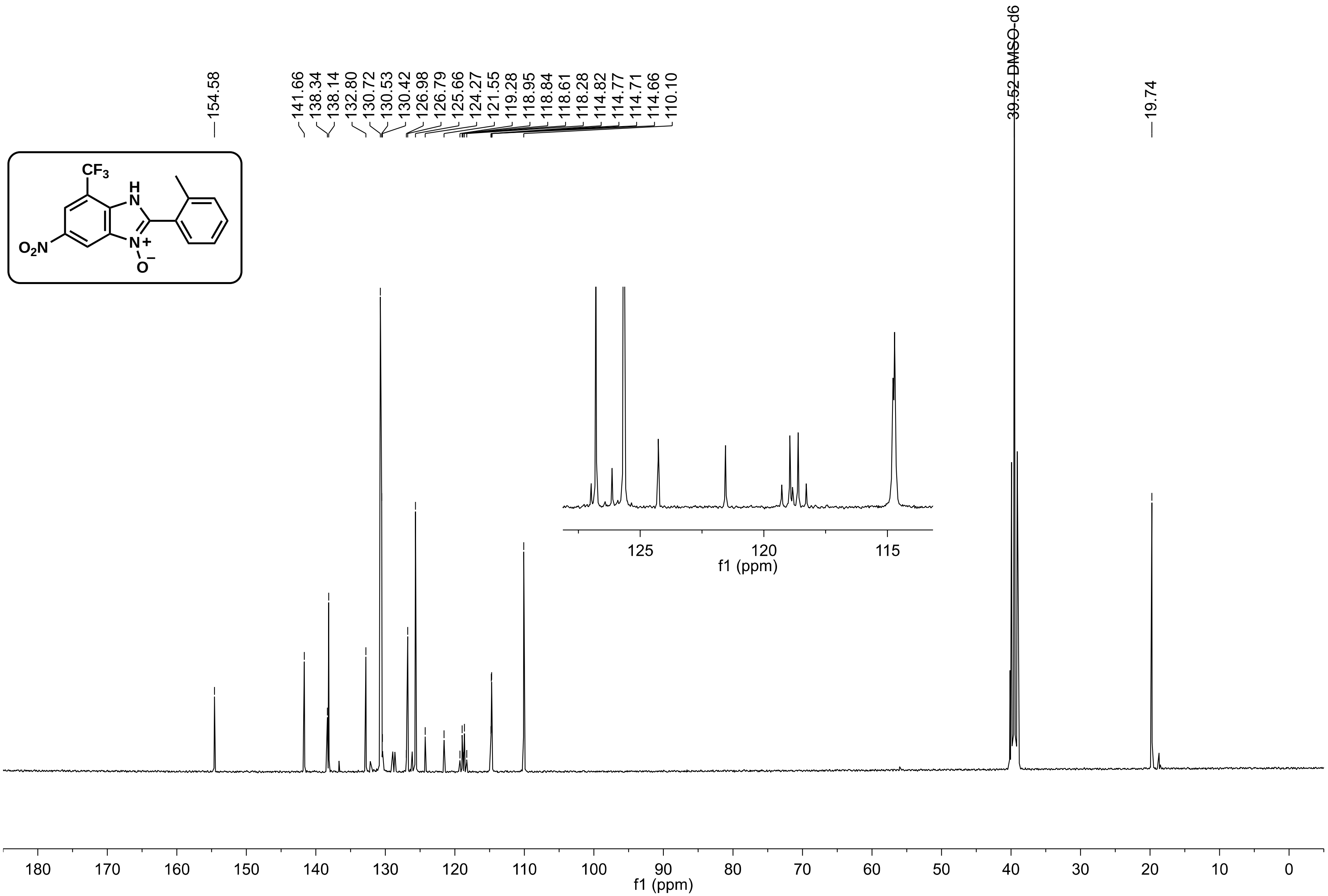

^{13}C-NMR (126 MHz $(CD_3)_2SO$). **2-(2-fluorophenyl)-5-nitro-7-trifluoromethyl-1*H*-benzimidazole 3-oxide (4cc)**

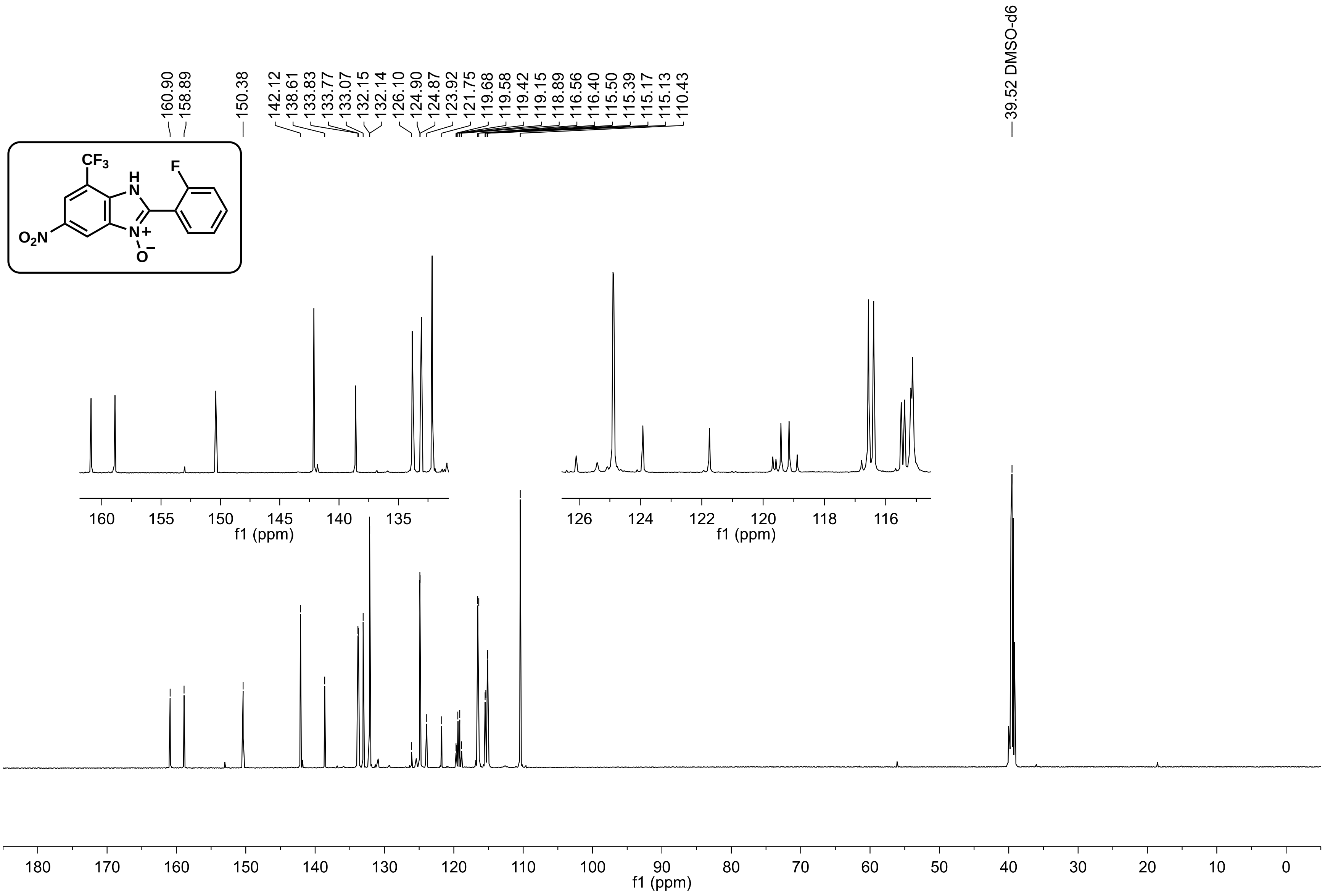

^{13}C-NMR (101 MHz $(CD_3)_2SO$). **2-(2-ethoxyphenyl)-5-nitro-7-trifluoromethyl-1*H*-benzimidazole 3-oxide (4cd)**

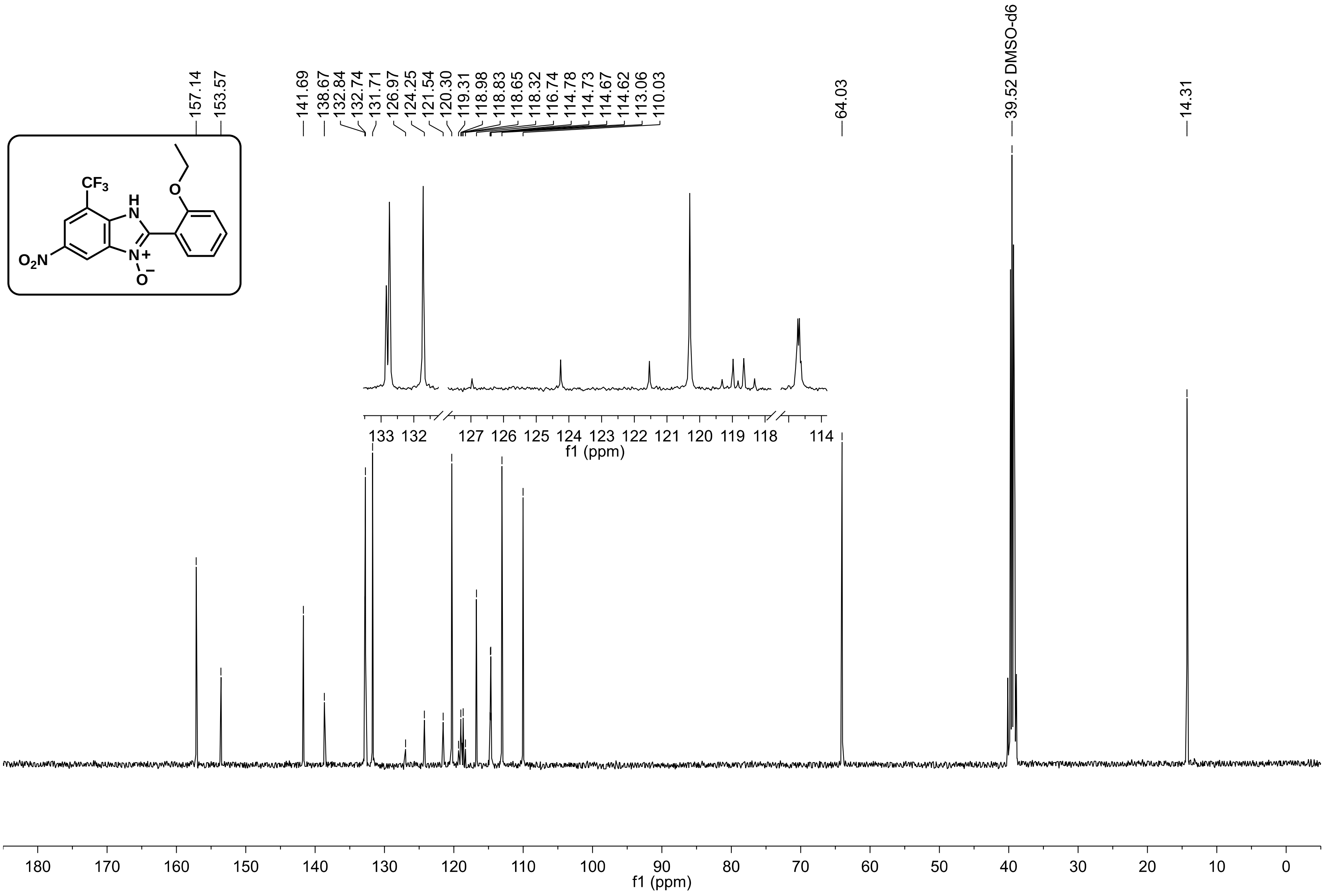

^{13}C-NMR (101 MHz $(CD_3)_2SO$). **2-(3-chlorophenyl)-5-nitro-7-trifluoromethyl-1*H*-benzimidazole 3-oxide (4ce)**

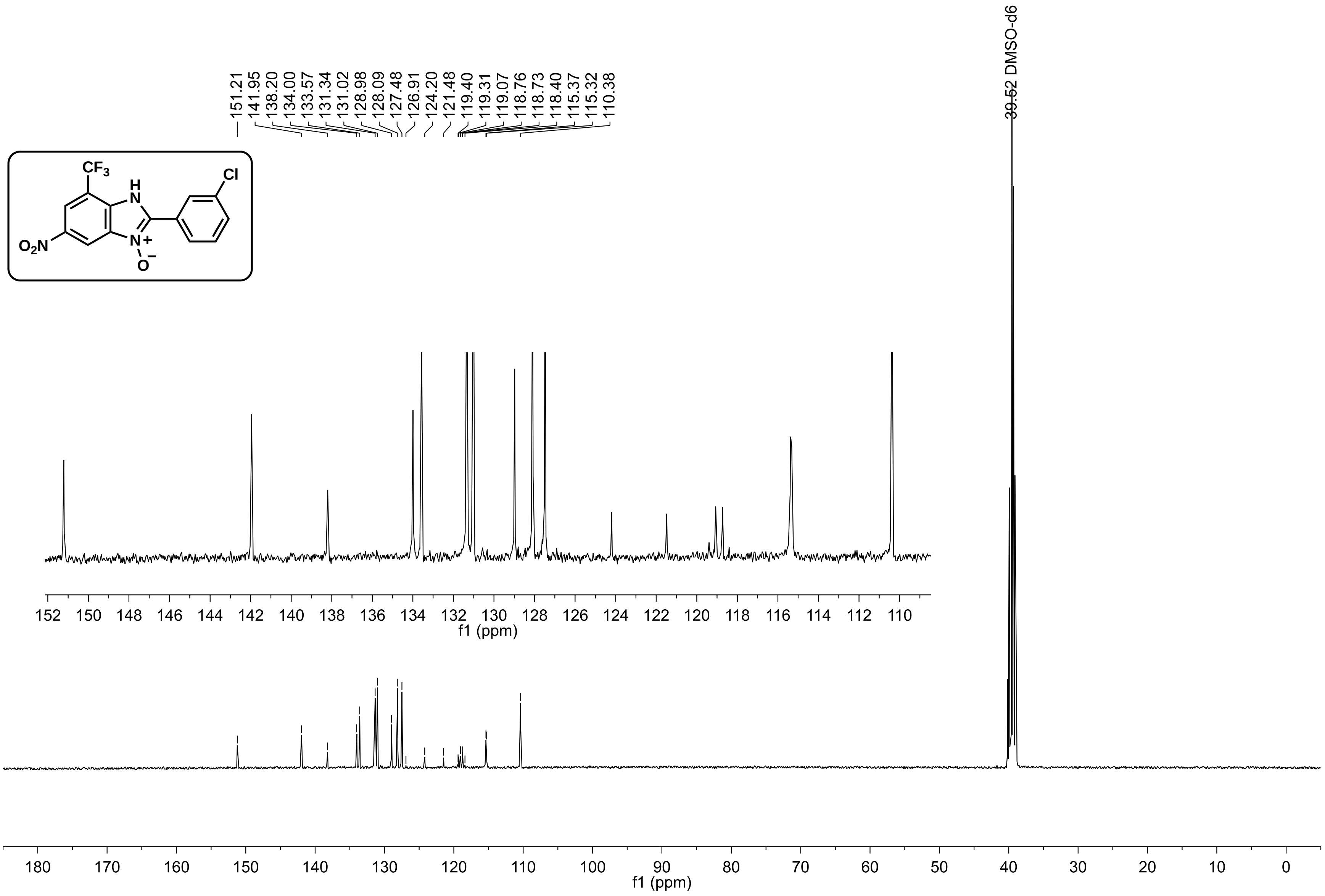

^{13}C-NMR (126 MHz $(CD_3)_2SO$). **2-(3-methoxyphenyl)-5-nitro-7-trifluoromethyl-1*H*-benzimidazole 3-oxide (4cf)**

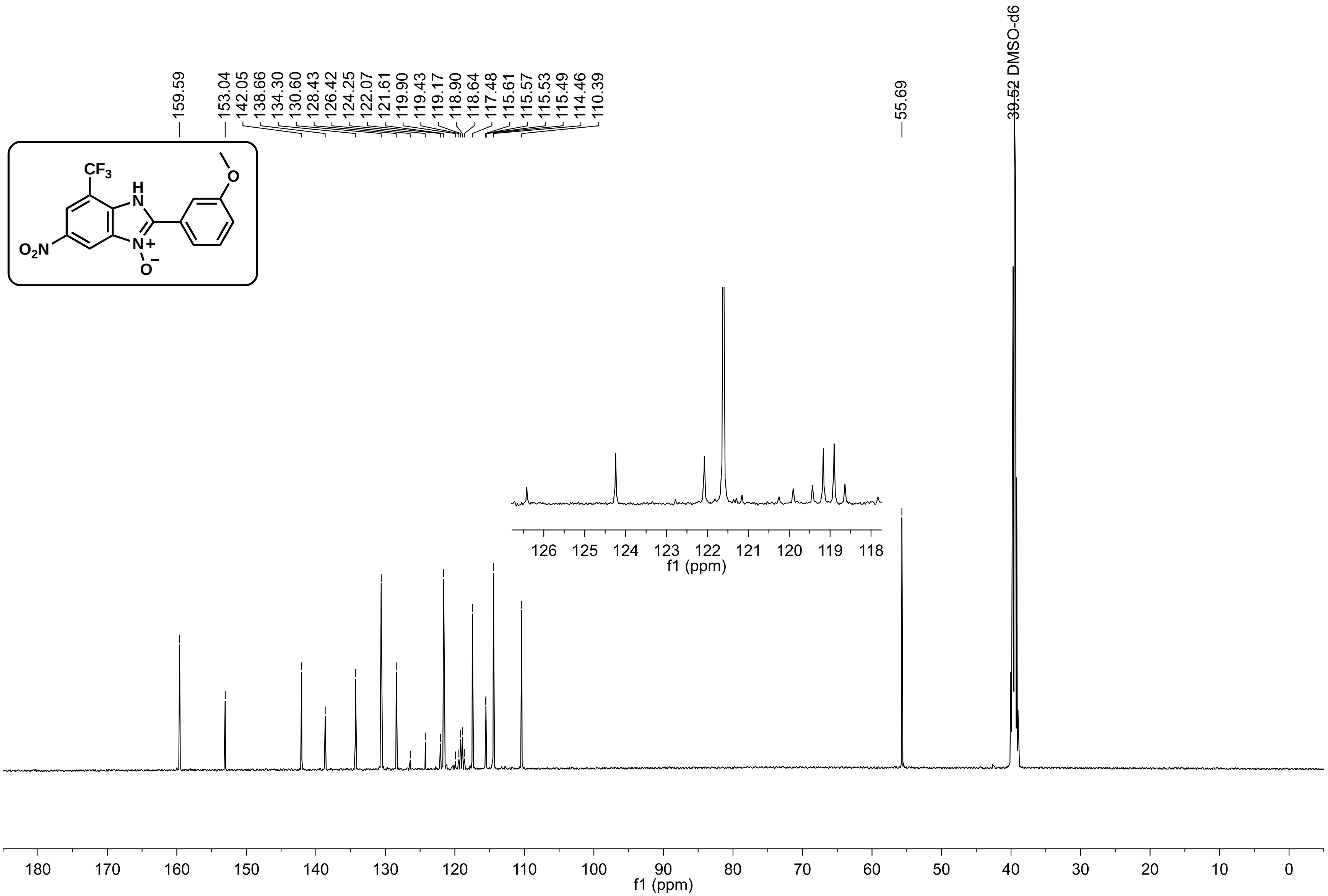

^{13}C-NMR (101 MHz $(CD_3)_2SO$). **5-nitro-2-(*p*-tolyl)-7-trifluoromethyl-1*H*-benzimidazole 3-oxide (4cg)**

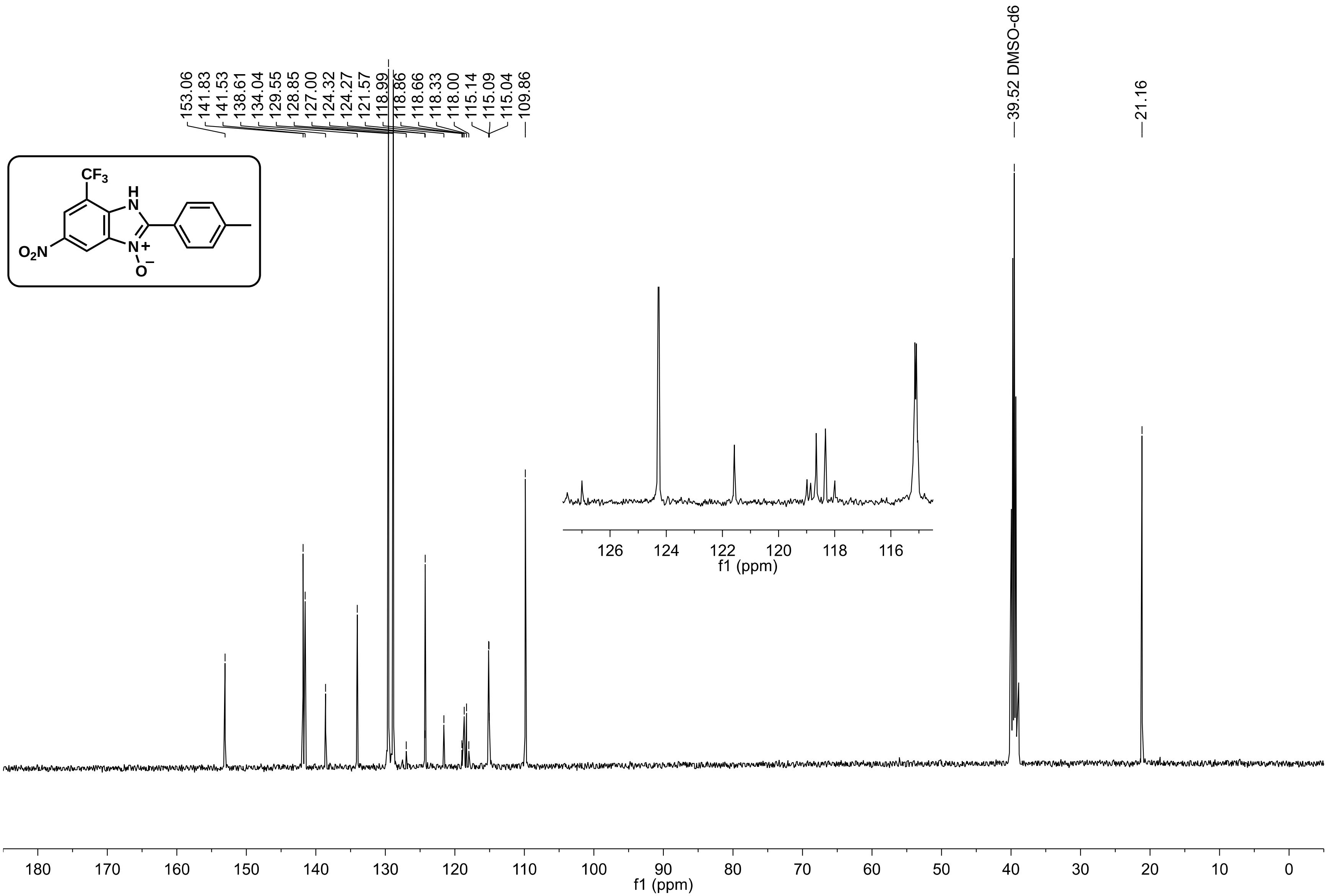

^{13}C-NMR (126 MHz $(CD_3)_2SO$). **2-(4-fluorophenyl)-5-nitro-7-trifluoromethyl-1*H*-benzimidazole 3-oxide (4ch)**

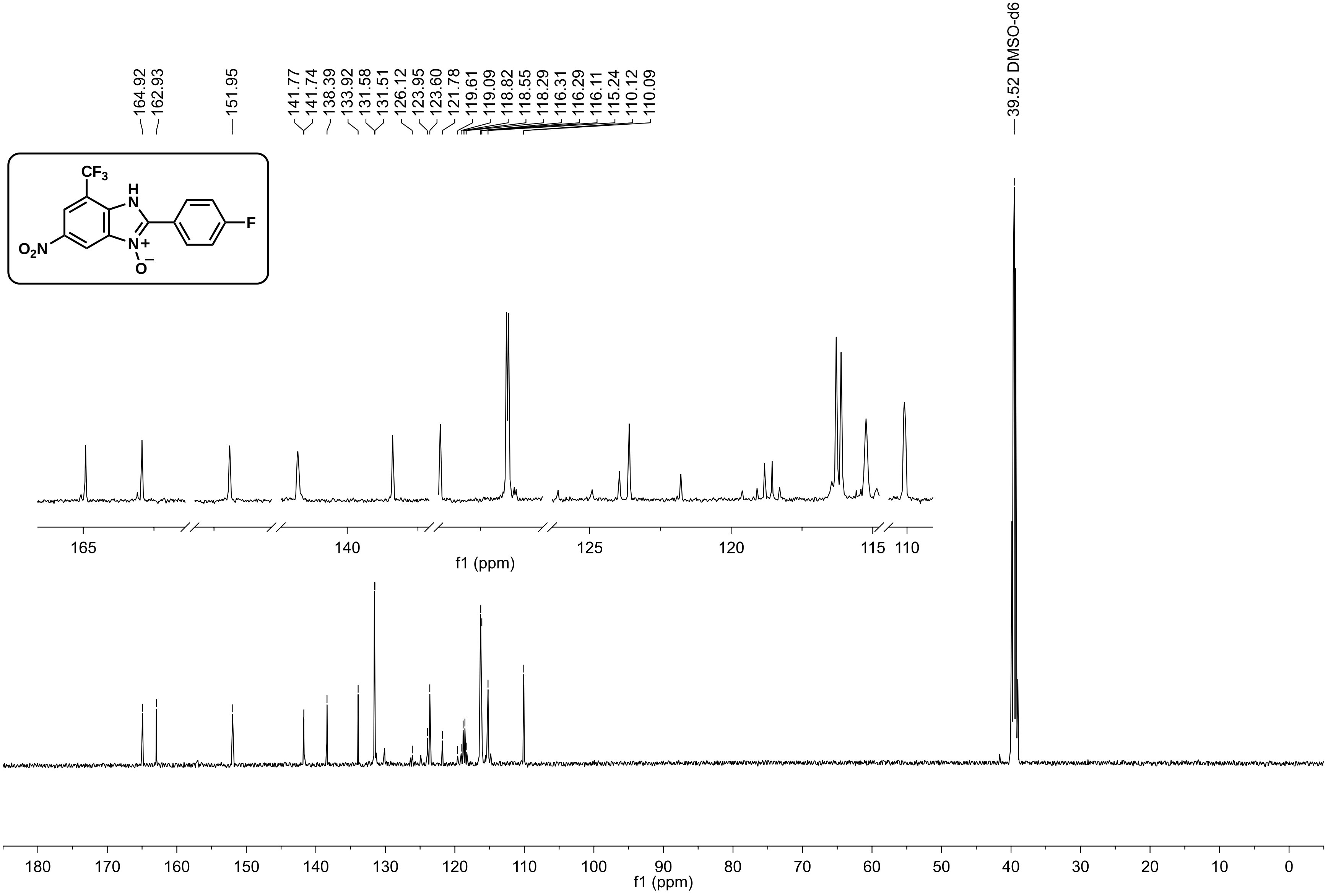

^{13}C-NMR (101 MHz $(CD_3)_2SO$). **2-(4-chlorophenyl)-5-nitro-7-trifluoromethyl-1*H*-benzimidazole 3-oxide (4ci)**

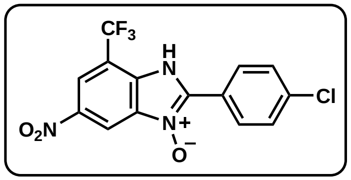

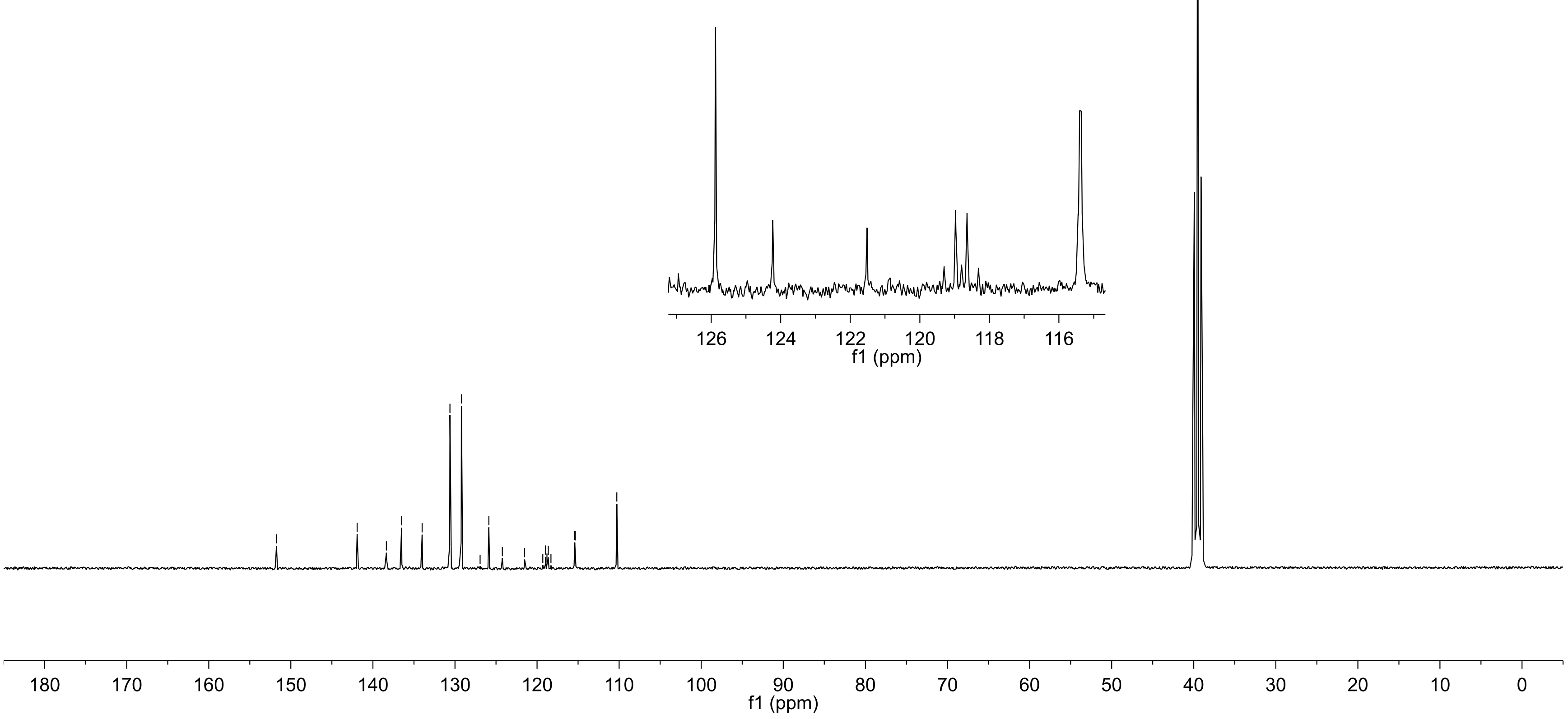

^{13}C-NMR (101 MHz $(CD_3)_2SO$). **2-(4-methoxyphenyl)-5-nitro-7-trifluoromethyl-1*H*-benzimidazole 3-oxide (4cj)**

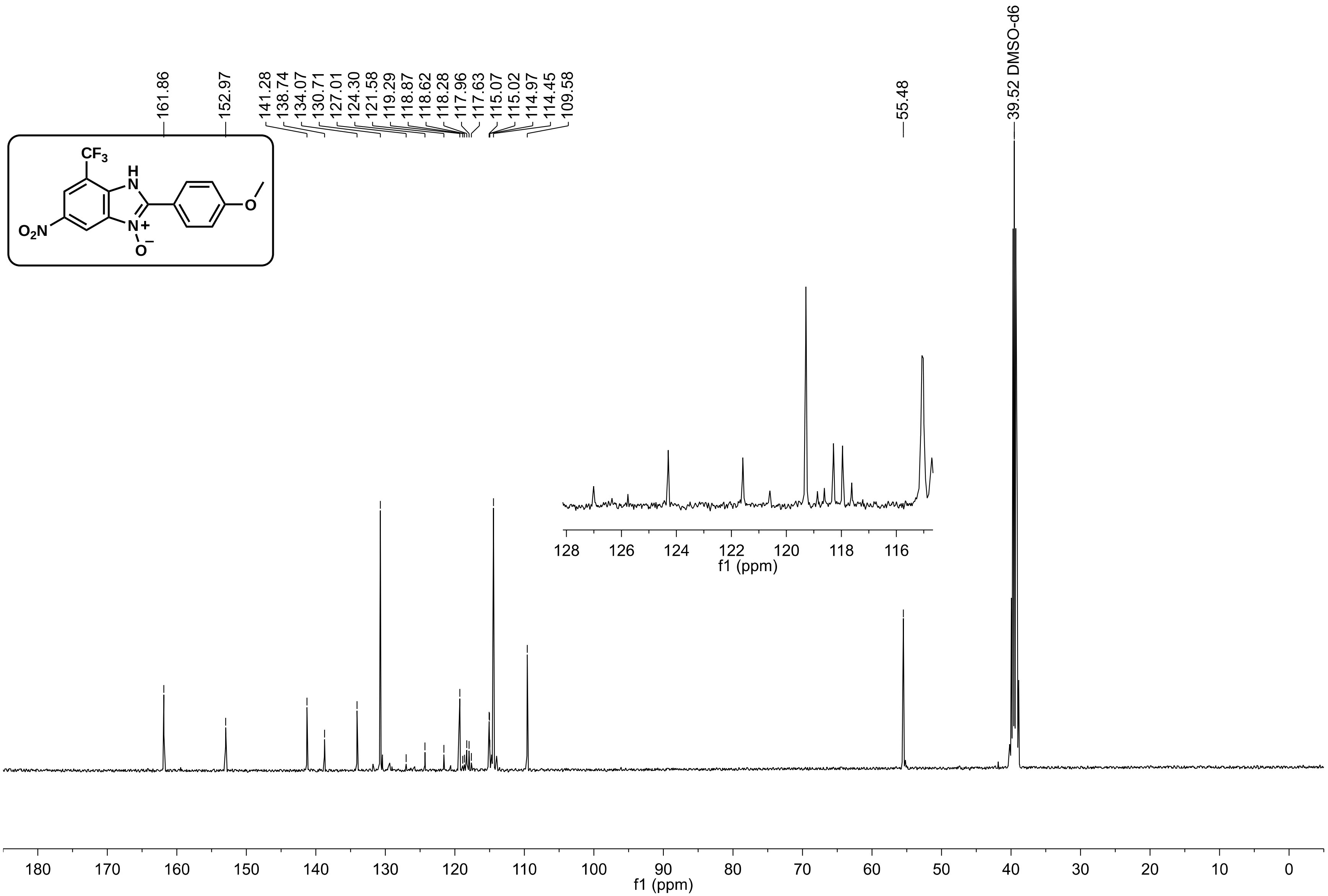

^{13}C-NMR (101 MHz $(CD_3)_2SO$). **2-(furan-2-yl)-5-nitro-7-trifluoromethyl-1*H*-benzimidazole 3-oxide (4ck)**

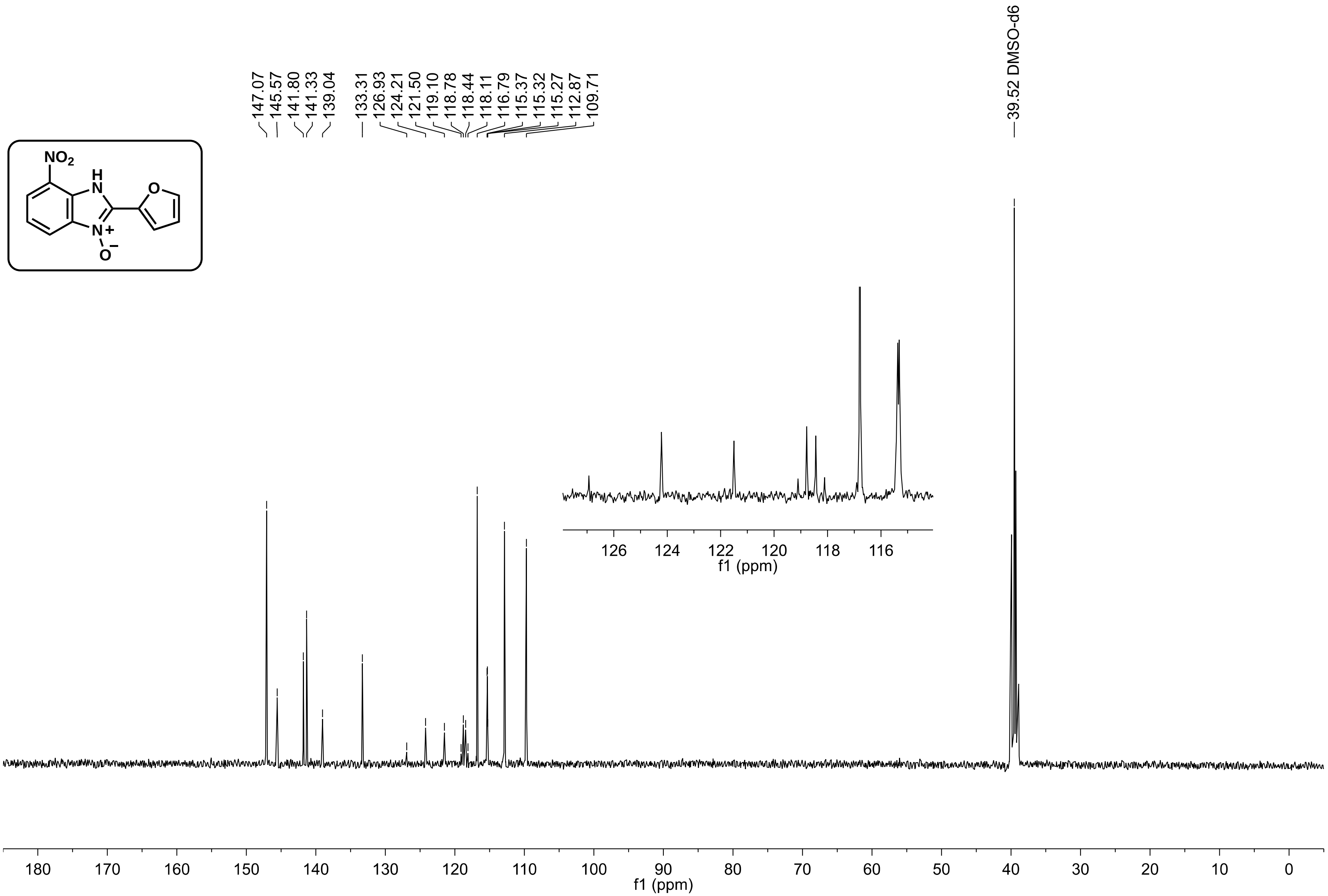

^{13}C-NMR (126 MHz $(CD_3)_2SO$). **5-nitro-2-(thiophen-2-yl)-7-trifluoromethyl-1*H*-benzimidazole 3-oxide (4cl)**

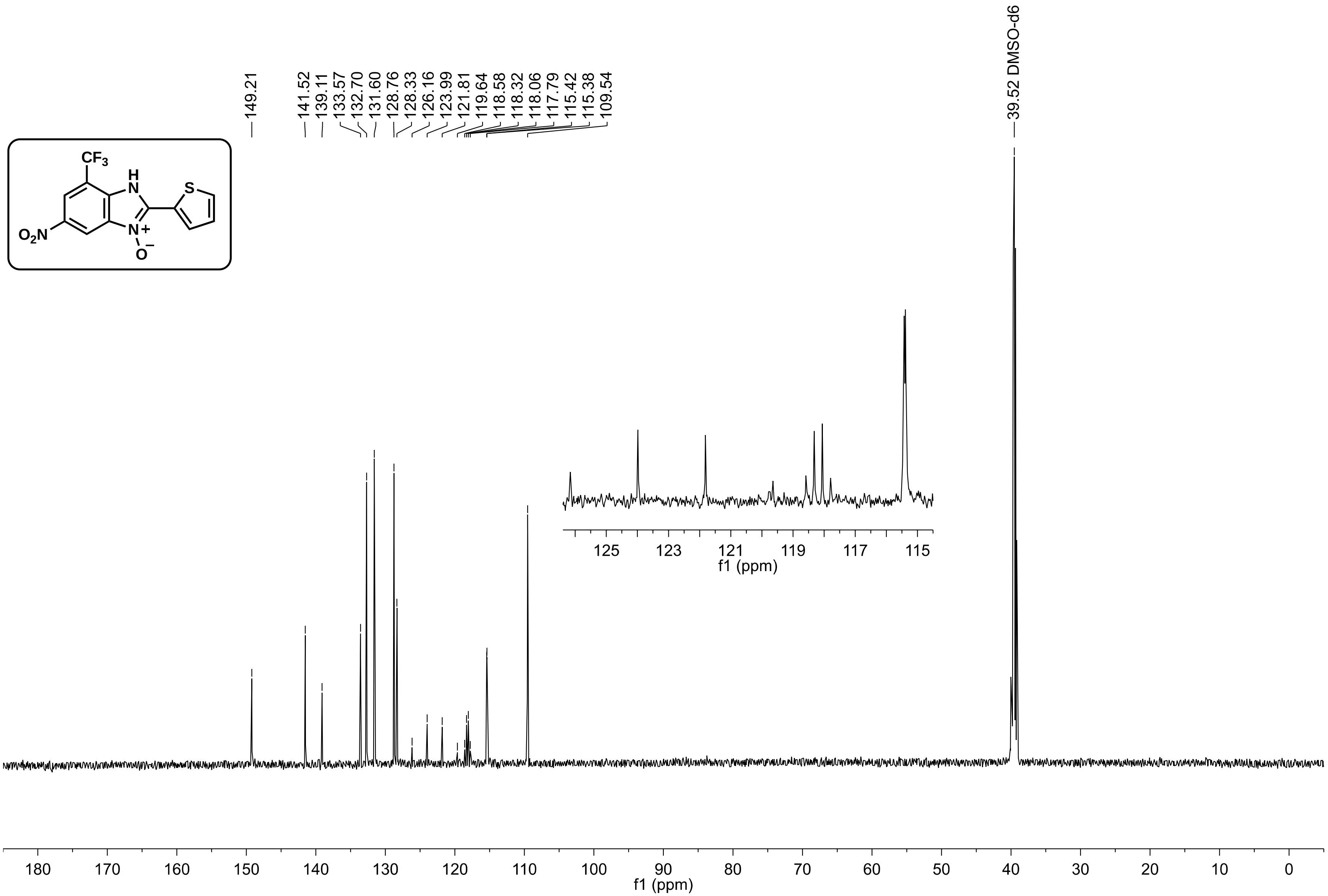

^{13}C-NMR (101 MHz $(CD_3)_2SO$). **5-nitro-2-(pyridin-3-yl)-7-trifluoromethyl-1*H*-benzimidazole 3-oxide (4cm)**

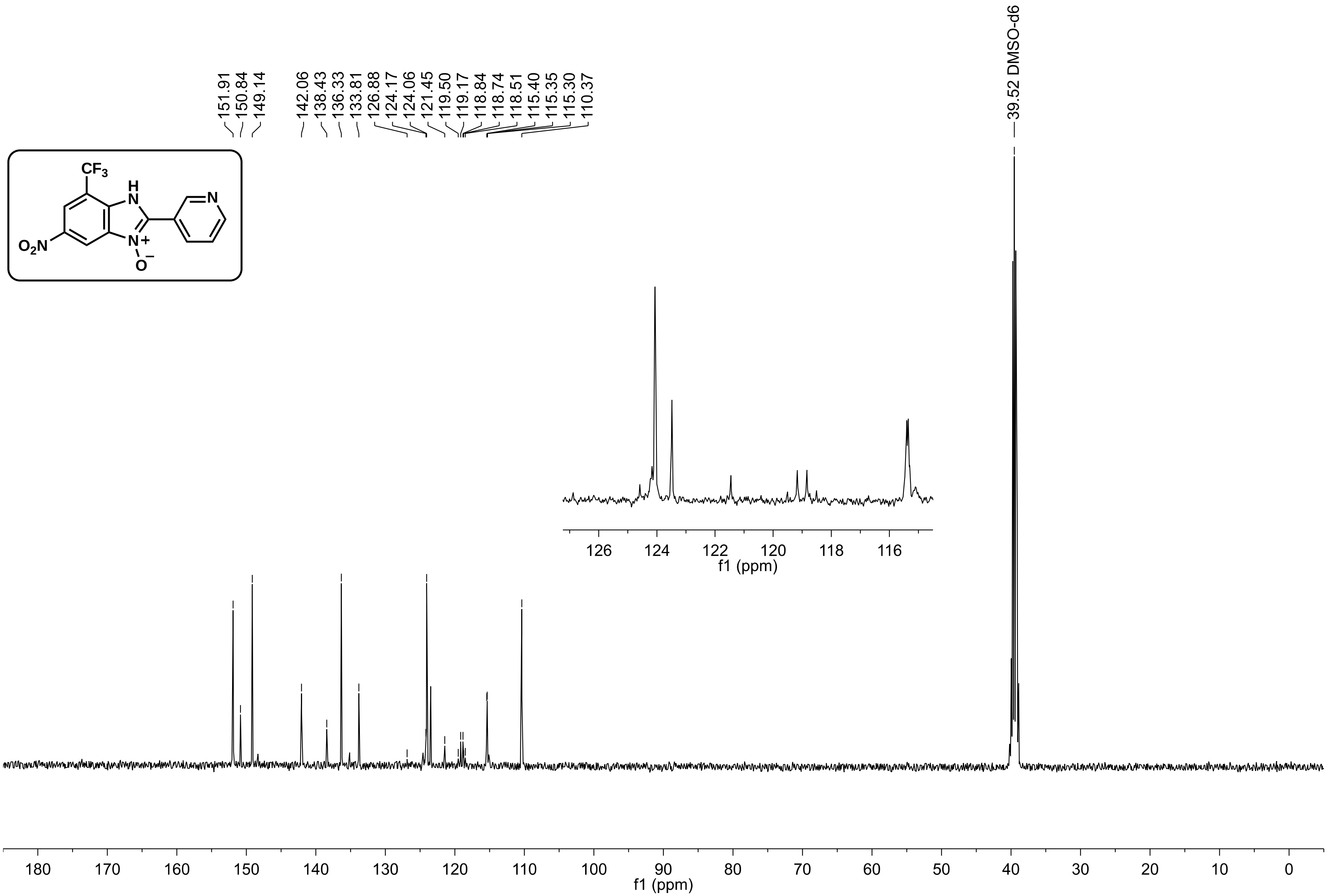

^{13}C-NMR (126 MHz $(CD_3)_2SO$). **2-benzyl-5-nitro-7-trifluoromethyl-1*H*-benzimidazole 3-oxide (4cn)**

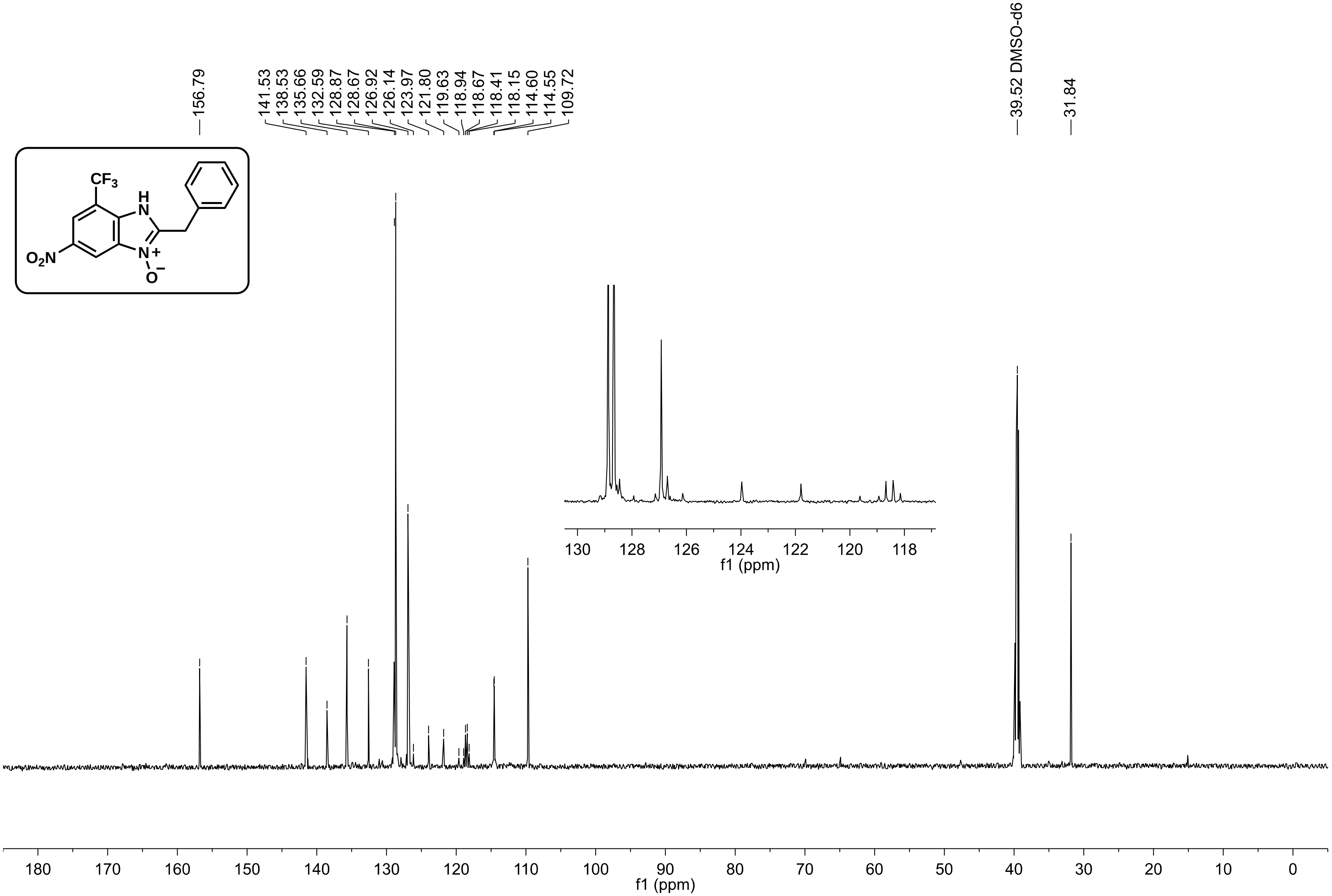

^{19}F NMR (376 MHz, $(CD_3)_2SO$). **2-(2-fluorophenyl)-7-nitro-1*H*-benzimidazole 3-oxide (4ac)**

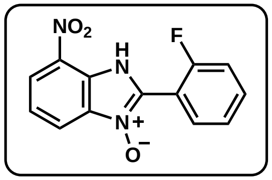

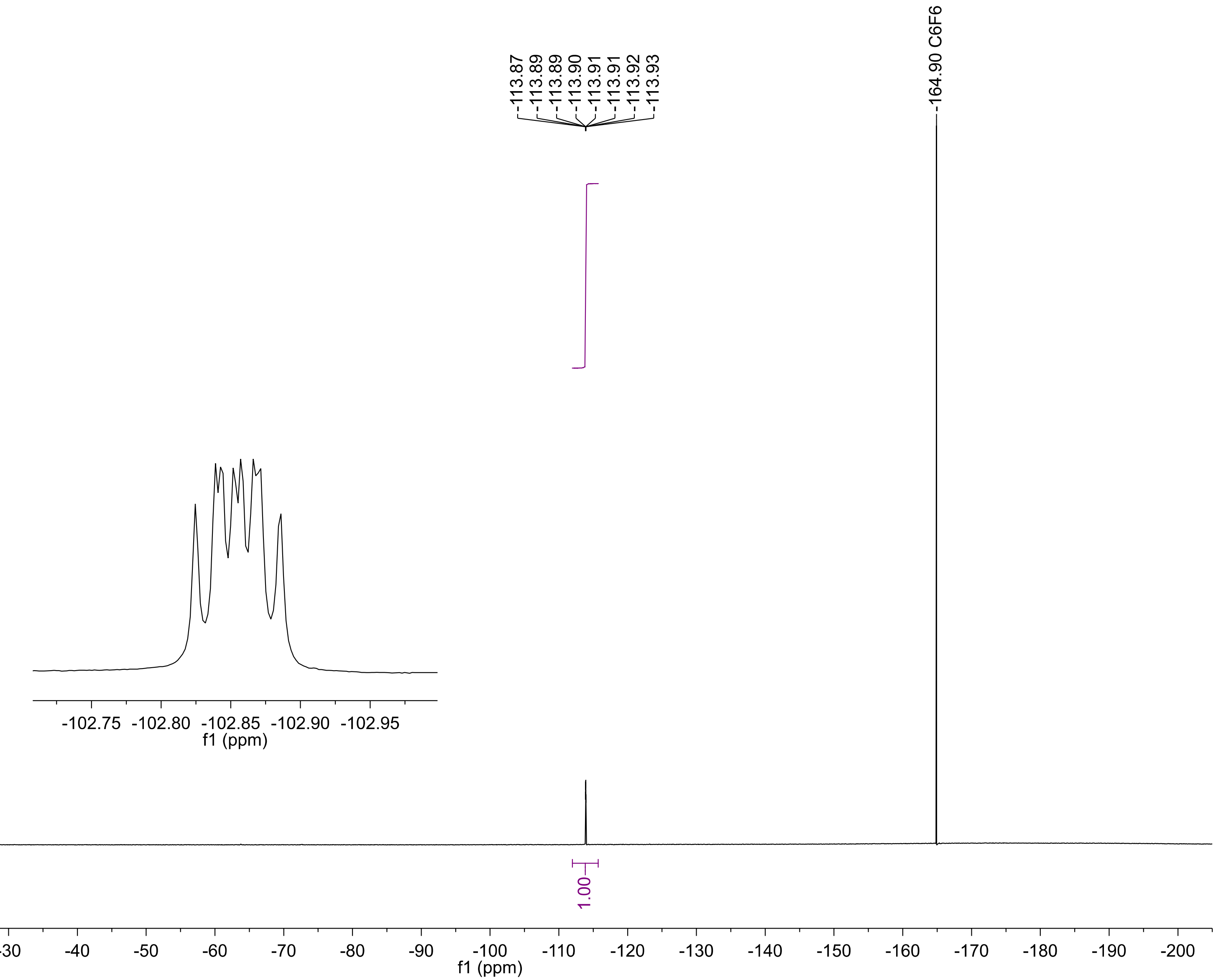

^{19}F-NMR (376 MHz $(CD_3)_2SO$). **2-(4-fluorophenyl)-7-nitro-1*H*-benzimidazole 3-oxide (4ah)**

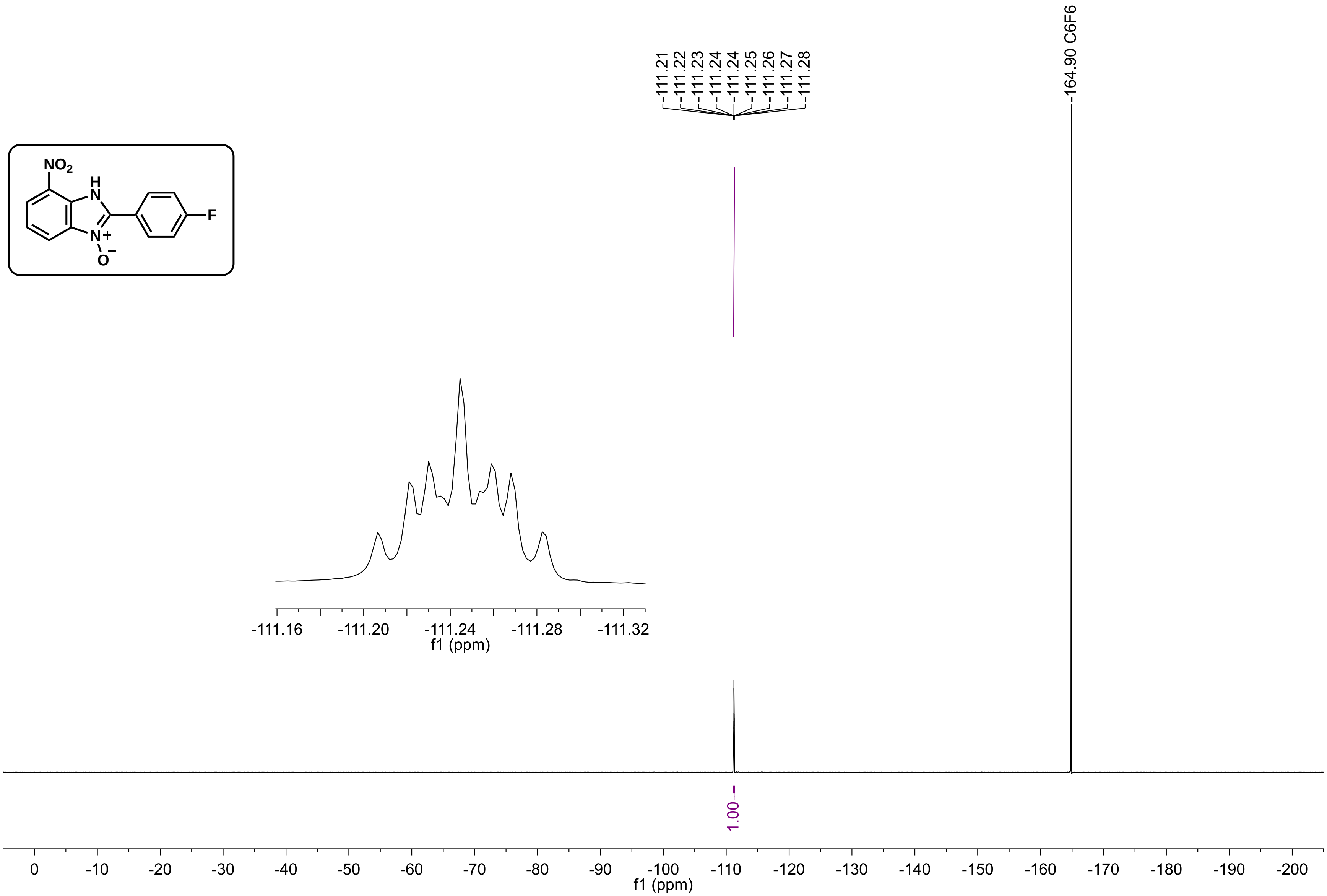

^{19}F-NMR (376 MHz $(CD_3)_2SO$). **7-nitro-2-phenyl-5-trifluoromethyl-1*H*-benzimidazole 3-oxide (4ba)**

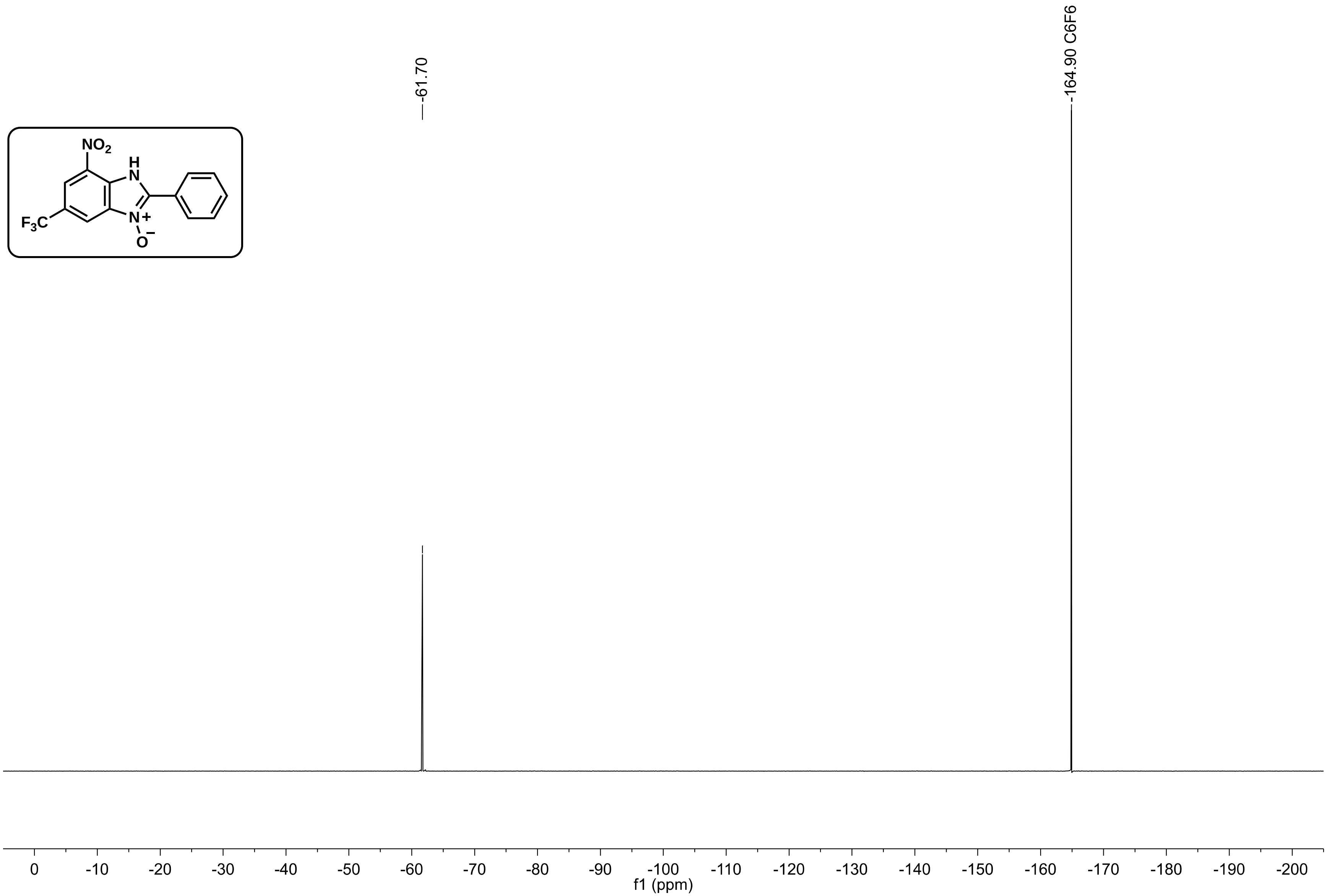

^{19}F-NMR (376 MHz $(CD_3)_2SO$). **7-nitro-2-(*o*-tolyl)-5-trifluoromethyl-1*H*-benzimidazole 3-oxide (4bb)**

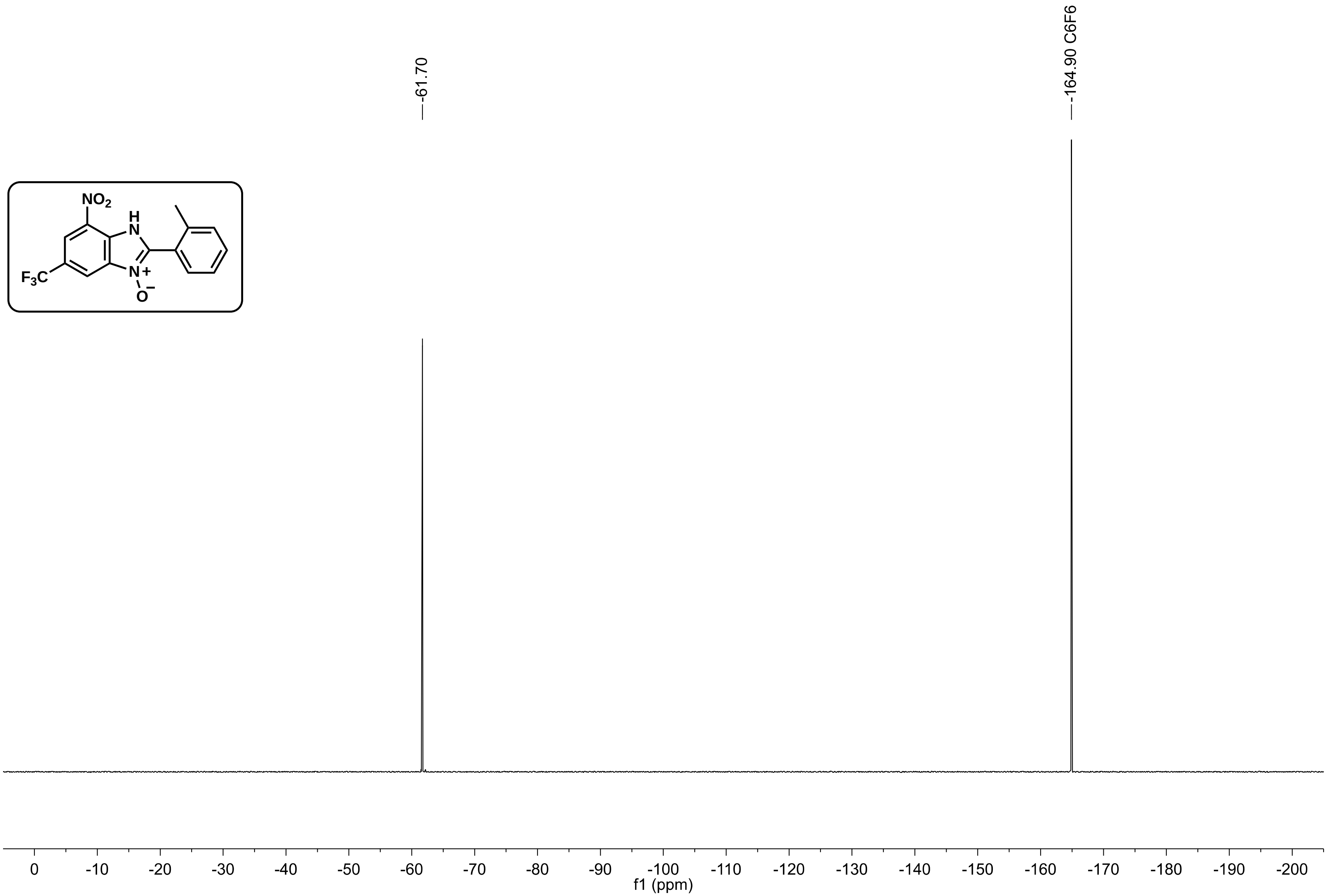

^{19}F-NMR (376 MHz $(CD_3)_2SO$). **2-(2-fluorophenyl)-7-nitro-5-trifluoromethyl-1*H*-benzimidazole 3-oxide (4bc)**

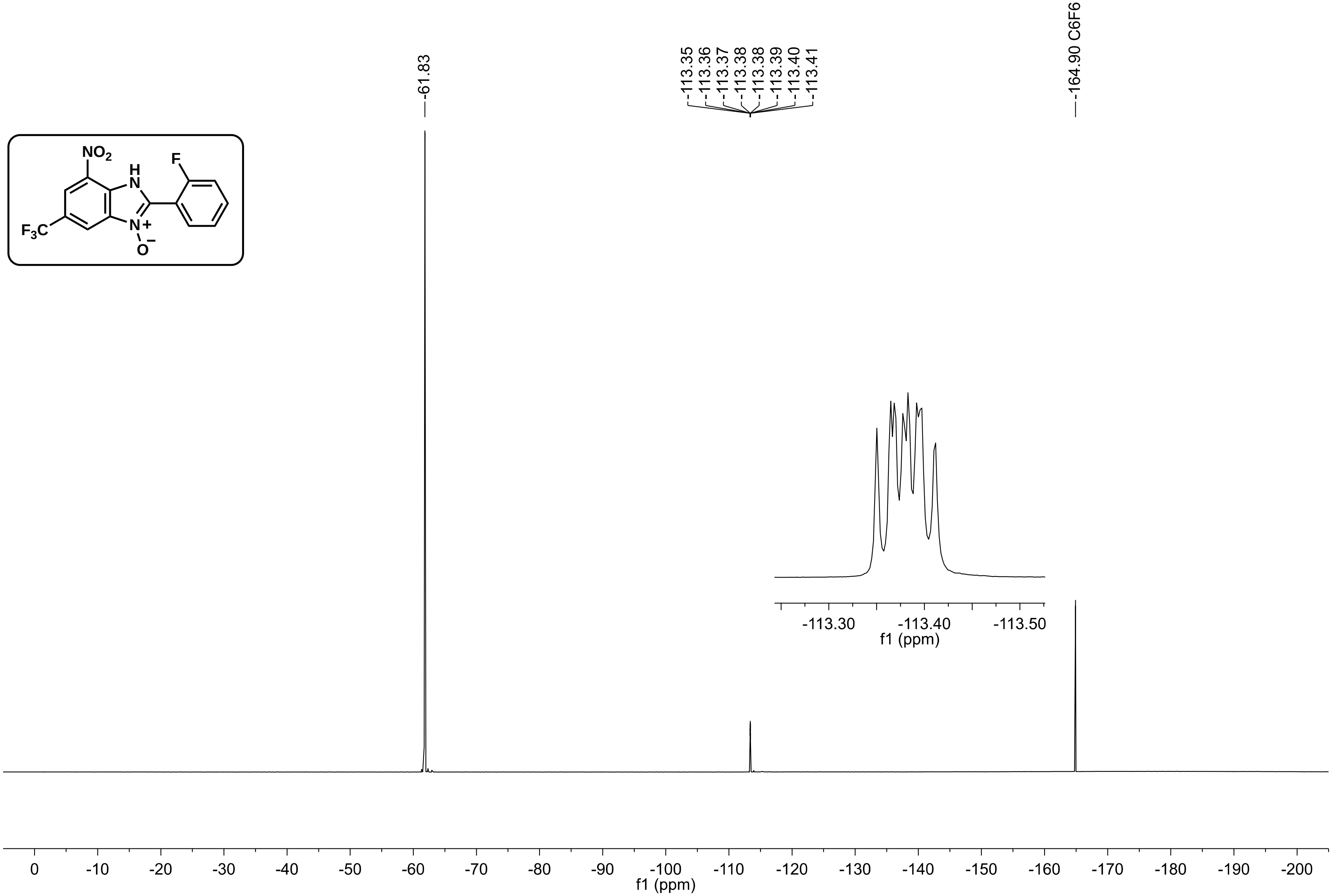

^{19}F-NMR (376 MHz $(CD_3)_2SO$). **2-(2-ethoxyphenyl)-7-nitro-5-trifluoromethyl-1*H*-benzimidazole 3-oxide (4bd)**

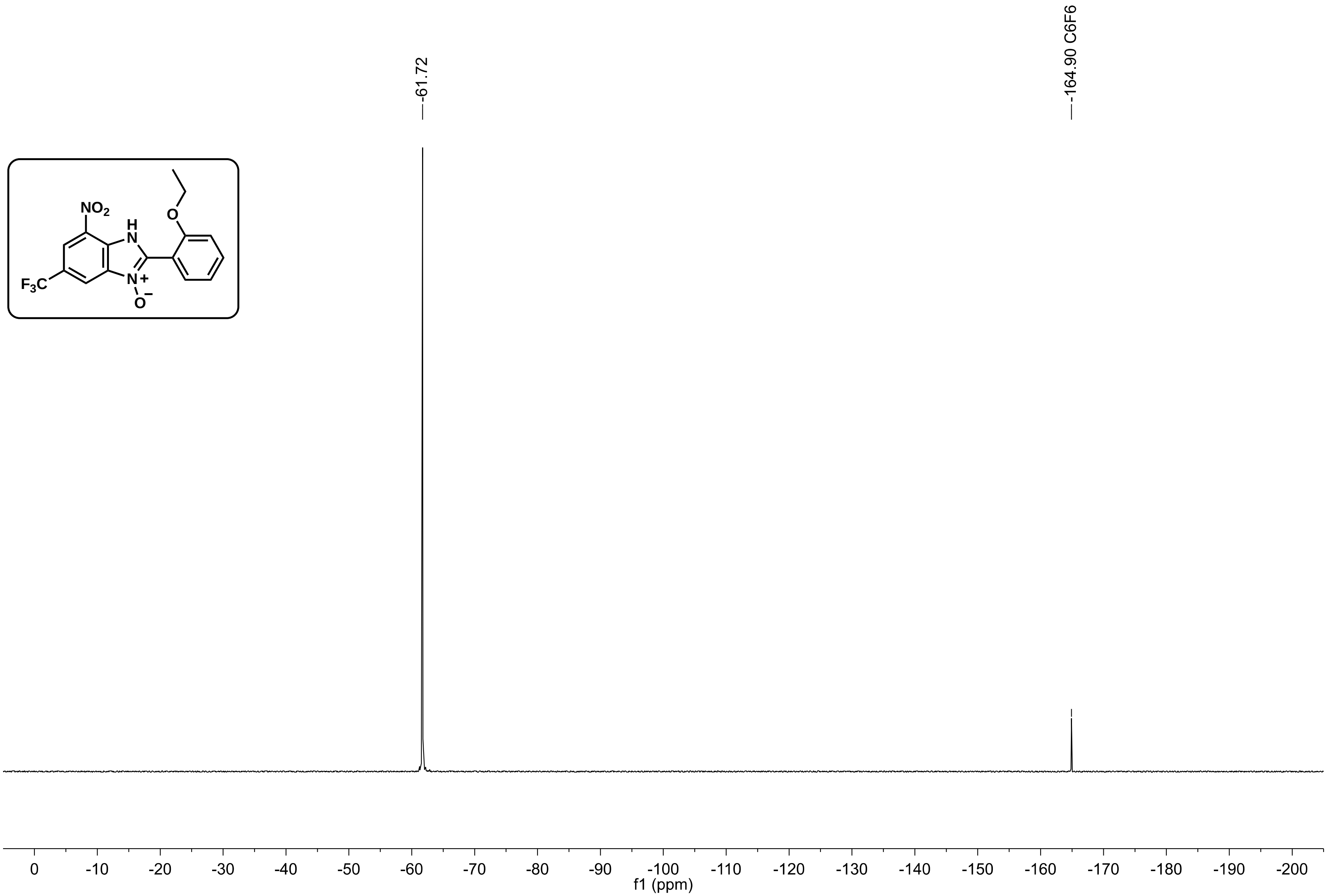

^{19}F-NMR (376 MHz $(CD_3)_2SO$). **2-(3-chlorophenyl)-7-nitro-5-trifluoromethyl-1*H*-benzimidazole 3-oxide (4be)**

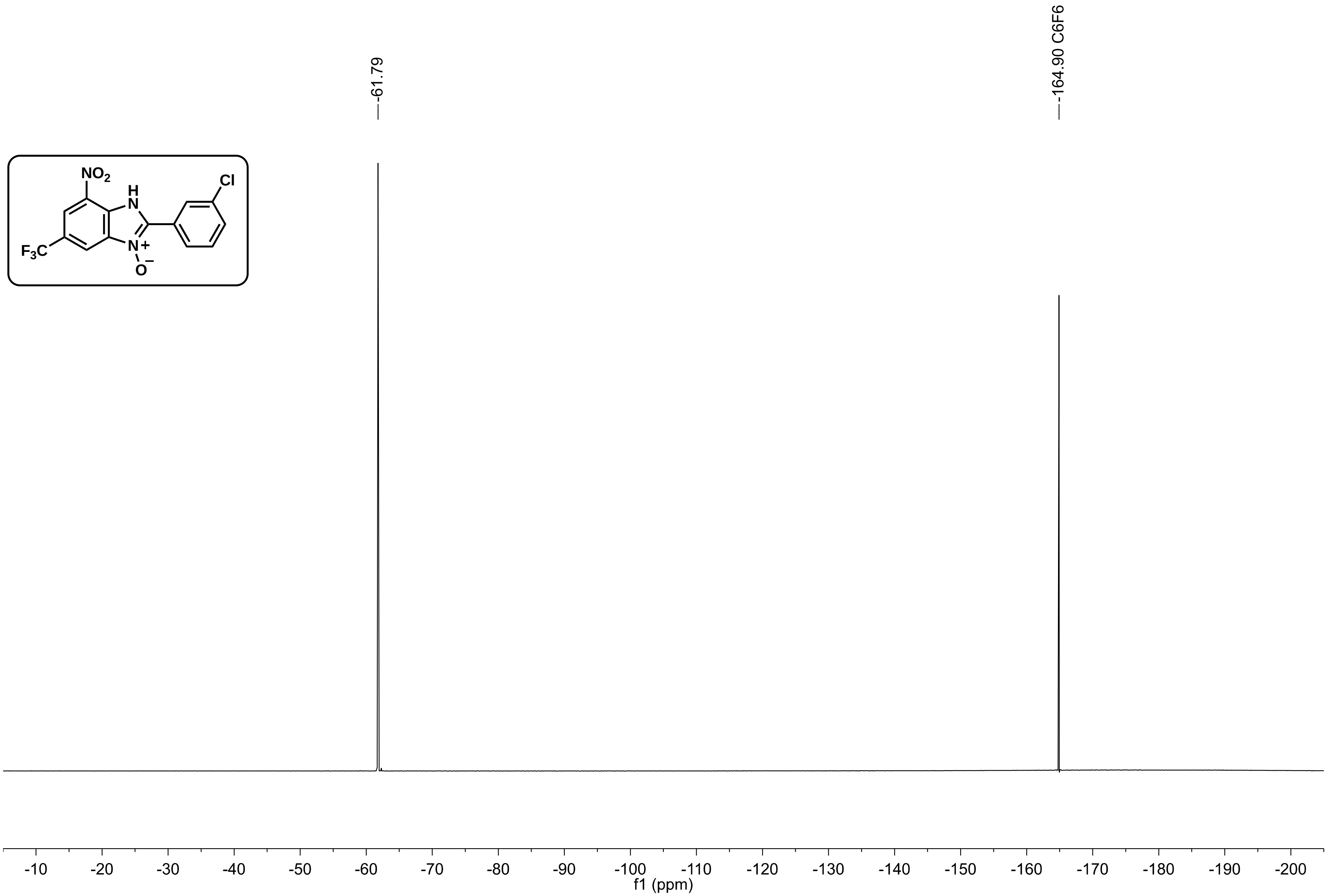

^{19}F-NMR (376 MHz $(CD_3)_2SO$). **2-(3-methoxyphenyl)-7-nitro-5-trifluoromethyl-1*H*-benzimidazole 3-oxide (4bf)**

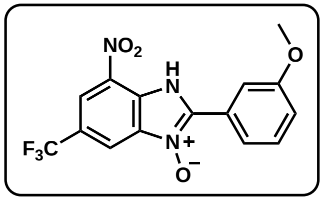

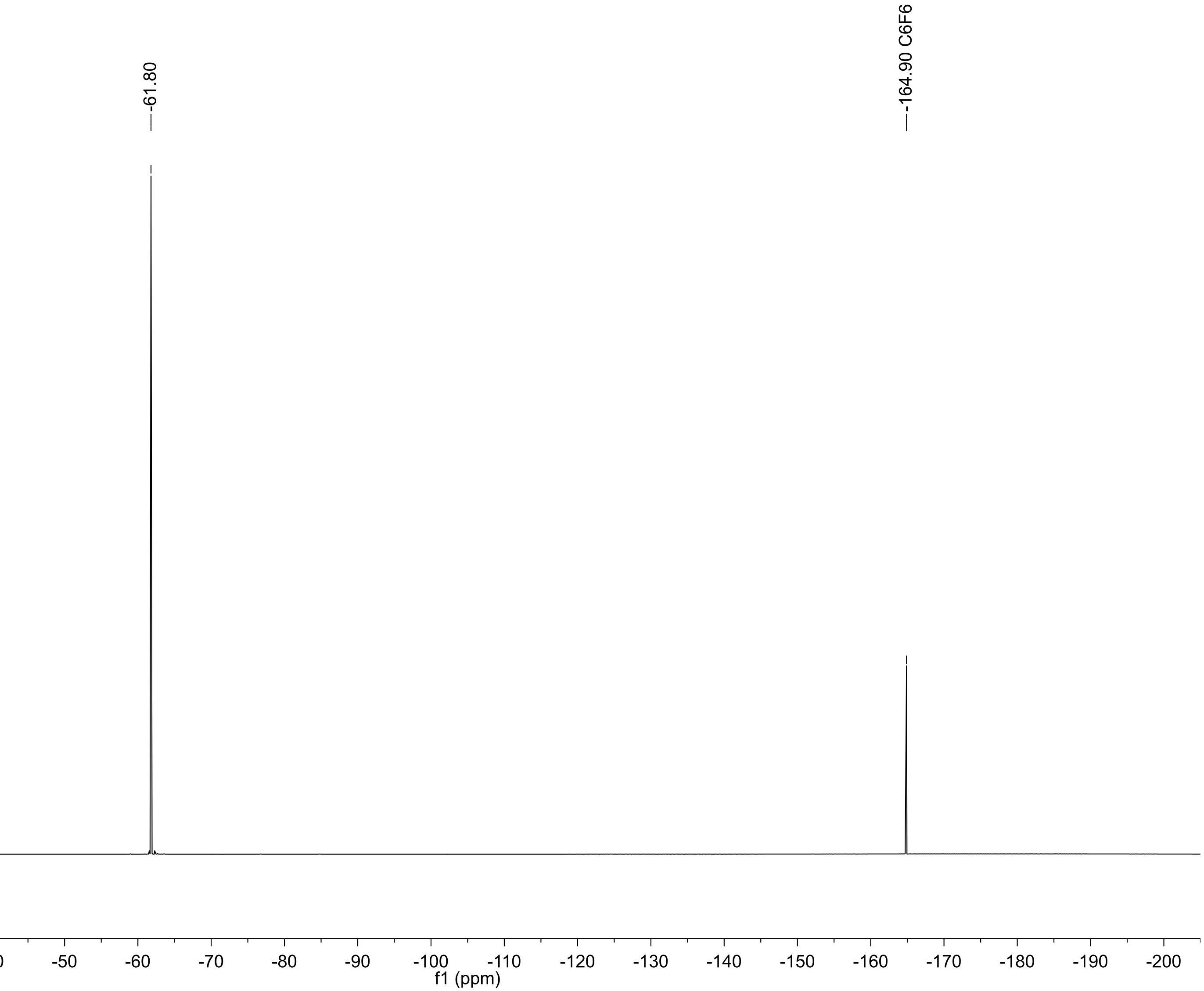

^{19}F-NMR (376 MHz $(CD_3)_2SO$). **7-nitro-2-(*p*-tolyl)-5-trifluoromethyl-1*H*-benzimidazole 3-oxide (4bg)**

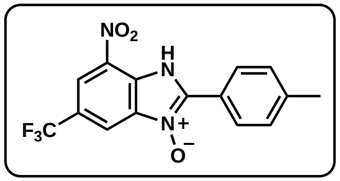

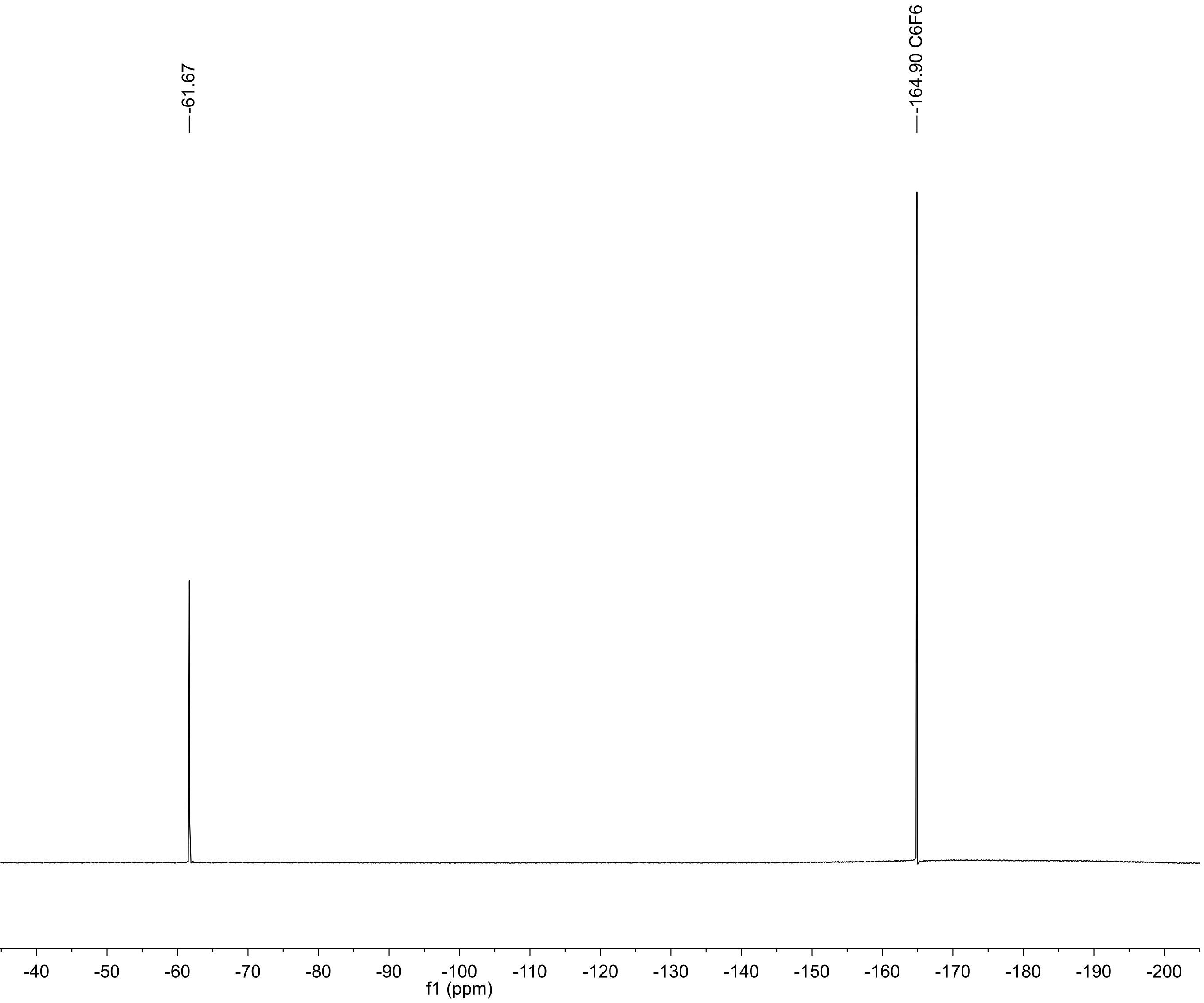

^{19}F-NMR (376 MHz $(CD_3)_2SO$). **2-(4-fluorophenyl)-7-nitro-5-trifluoromethyl-1*H*-benzimidazole 3-oxide (4bh)**

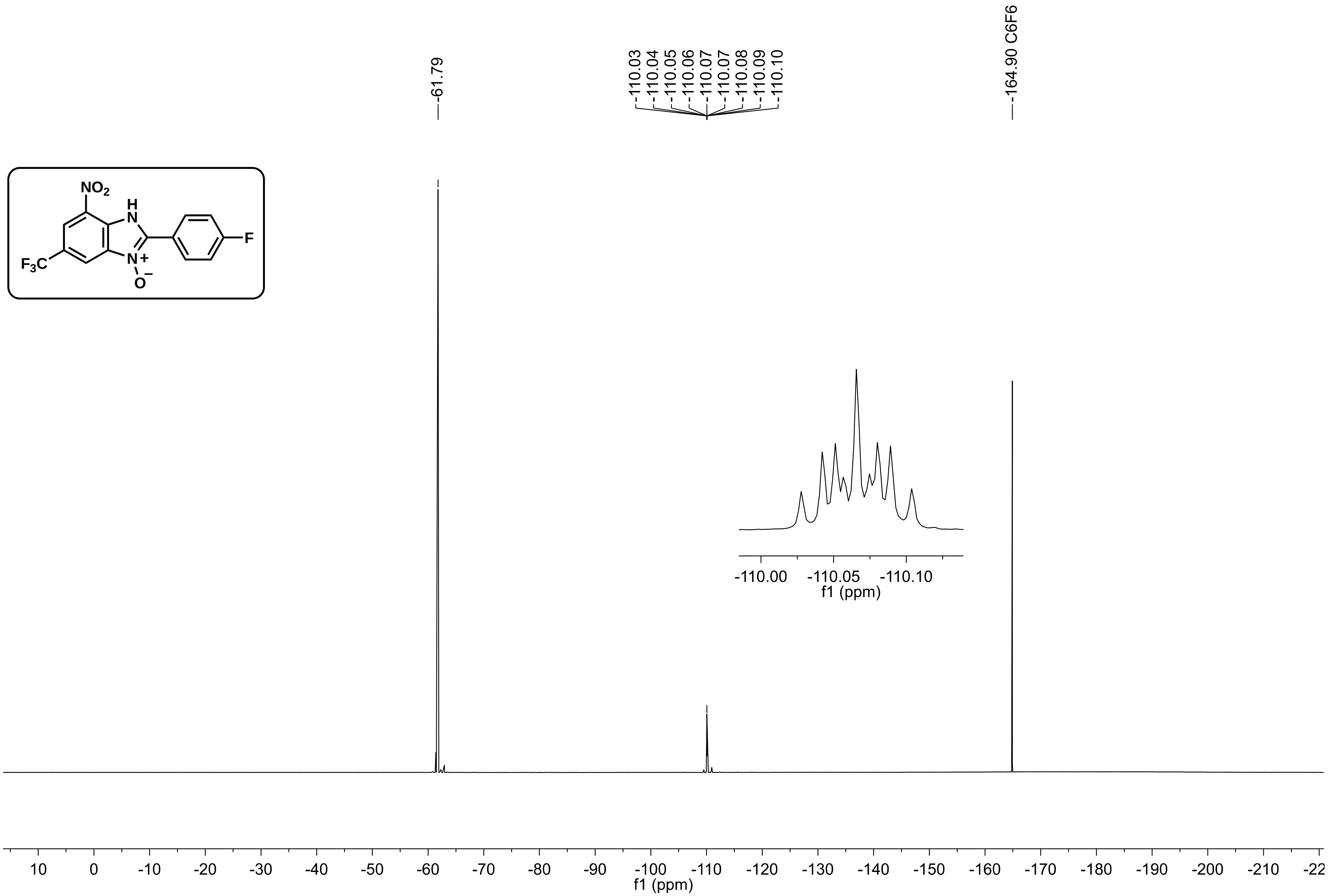

^{19}F-NMR (376 MHz $(CD_3)_2SO$). **2-(4-chlorophenyl)-7-nitro-5-trifluoromethyl-1*H*-benzimidazole 3-oxide (4bi)**

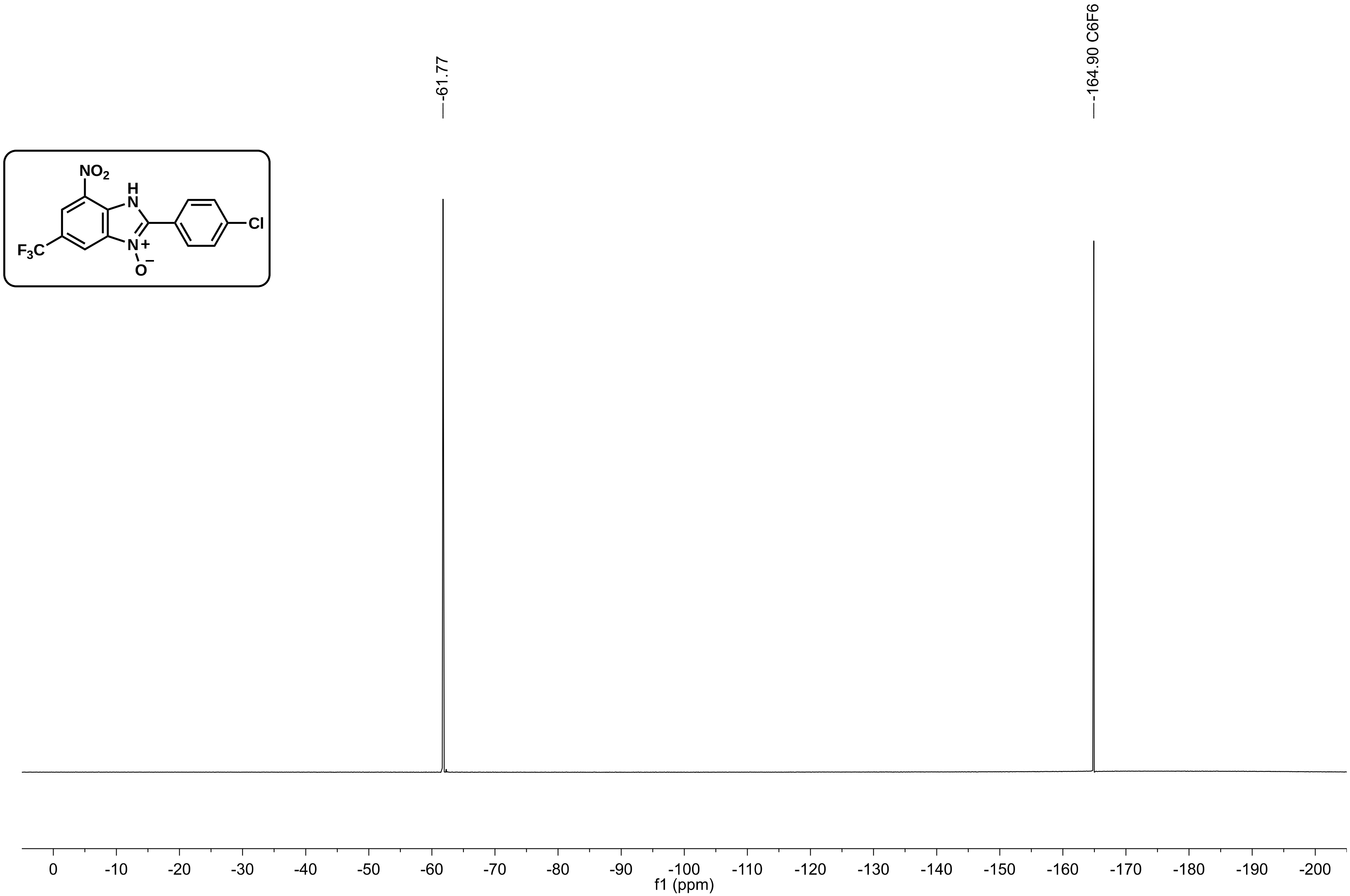

^{19}F-NMR (376 MHz $(CD_3)_2SO$). **2-(4-methoxyphenyl)-7-nitro-5-trifluoromethyl-1*H*-benzimidazole 3-oxide (4bj)**

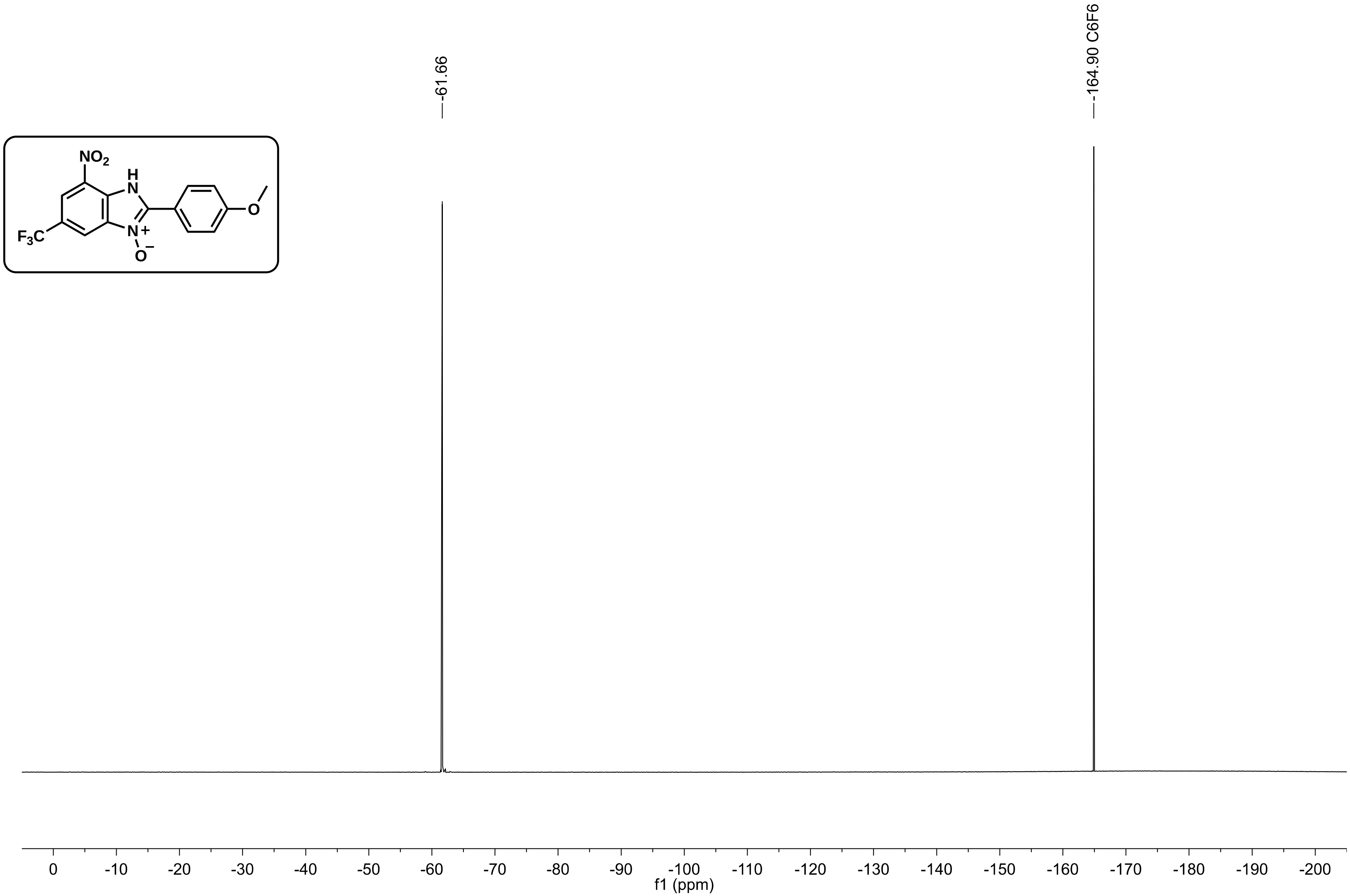

^{19}F-NMR (376 MHz $(CD_3)_2SO$). **2-(furan-2-yl)-7-nitro-5-trifluoromethyl-1*H*-benzimidazole 3-oxide (4bk)**

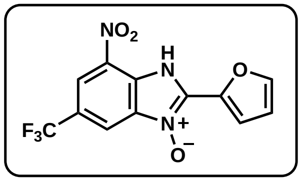

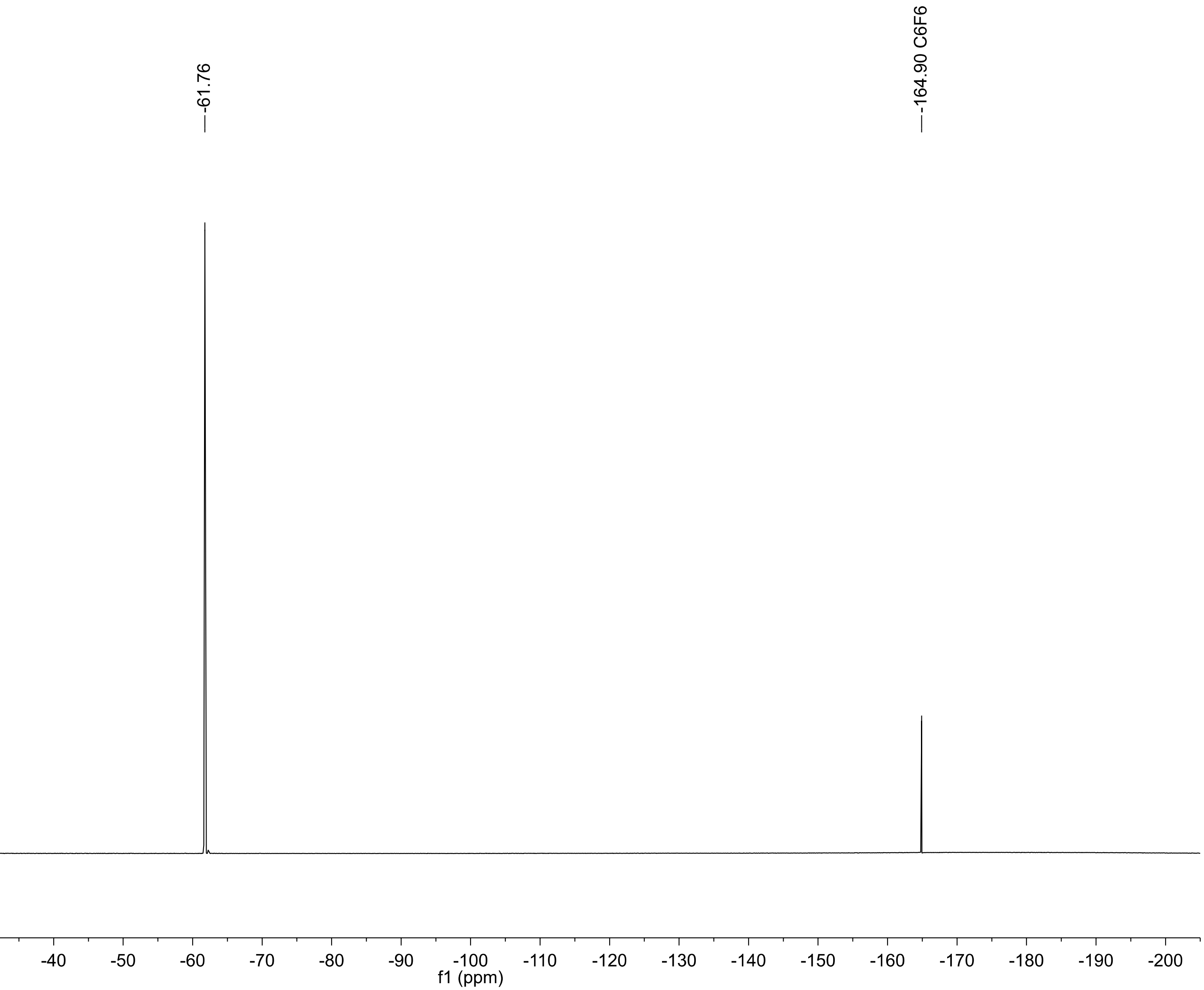

^{19}F-NMR (376 MHz $(CD_3)_2SO$). **7-nitro-2-(thiophen-2-yl)-5-trifluoromethyl-1*H*-benzimidazole 3-oxide (4bl)**

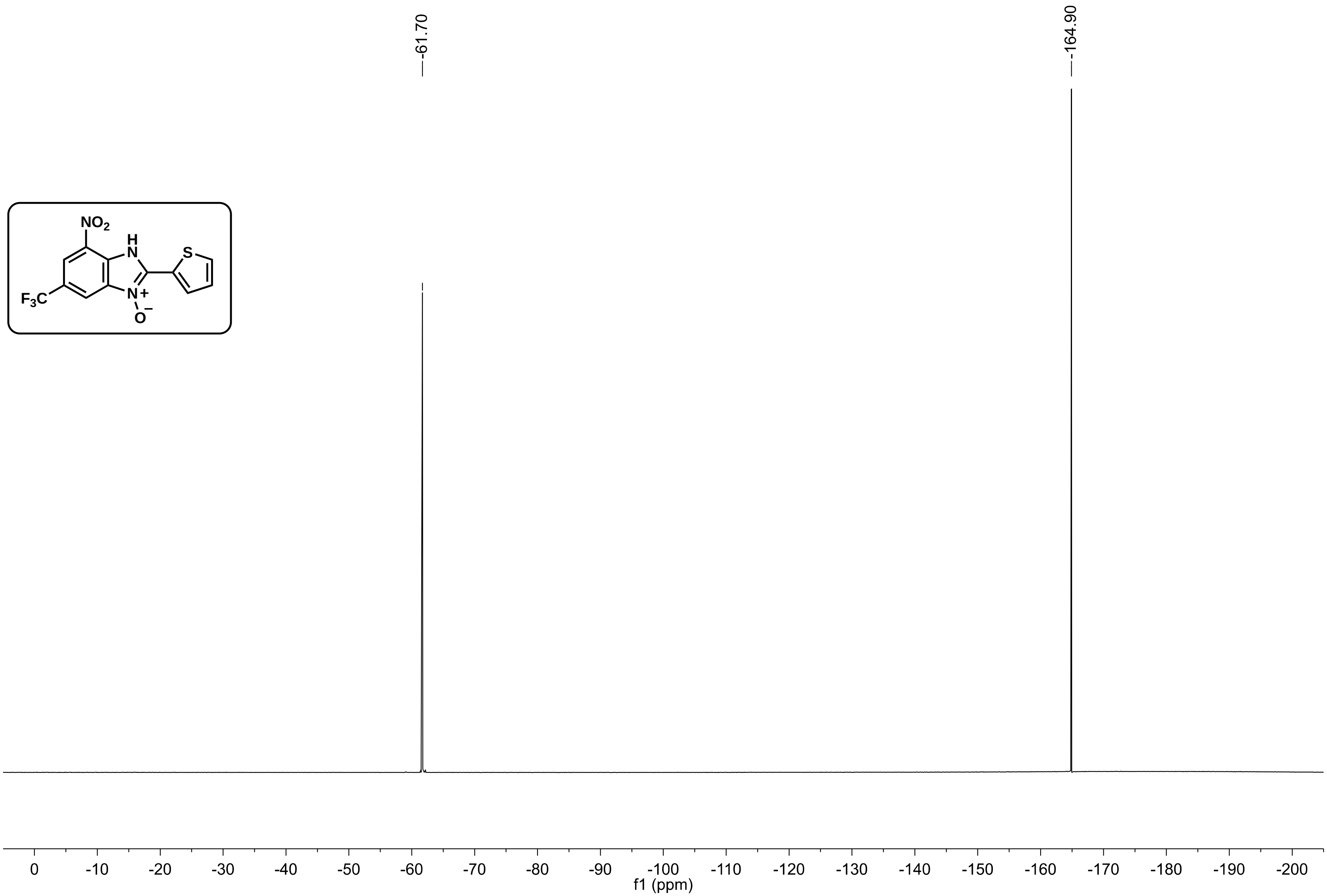

^{19}F-NMR (376 MHz $(CD_3)_2SO$). **7-nitro-2-(pyridin-3-yl)-5-trifluoromethyl-1*H*-benzimidazole 3-oxide (4bm)**

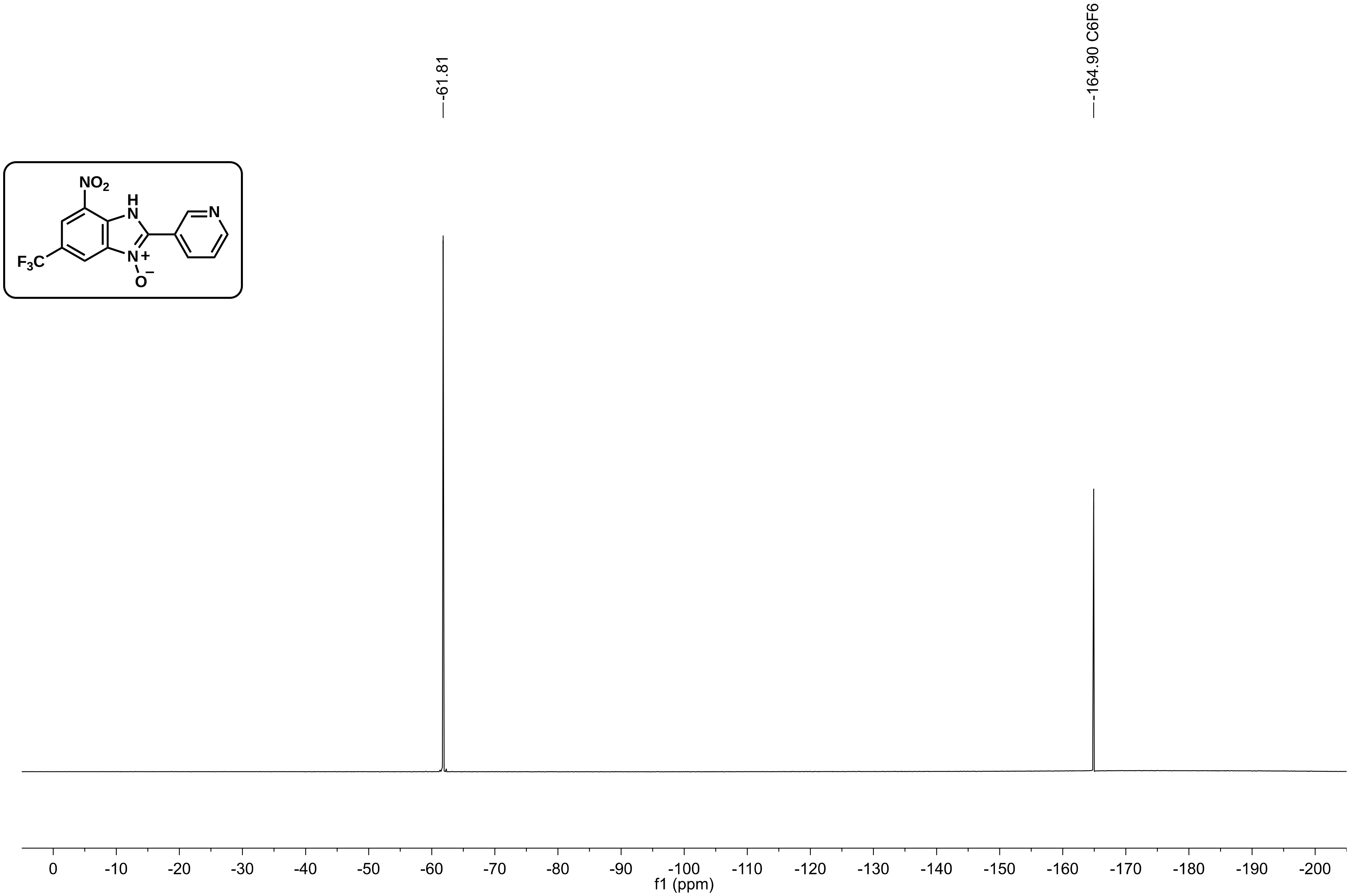

^{19}F-NMR (376 MHz $(CD_3)_2SO$). **2-benzyl-7-nitro-5-trifluoromethyl-1*H*-benzimidazole 3-oxide (4bn)**

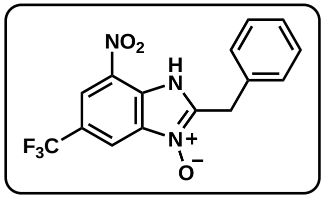

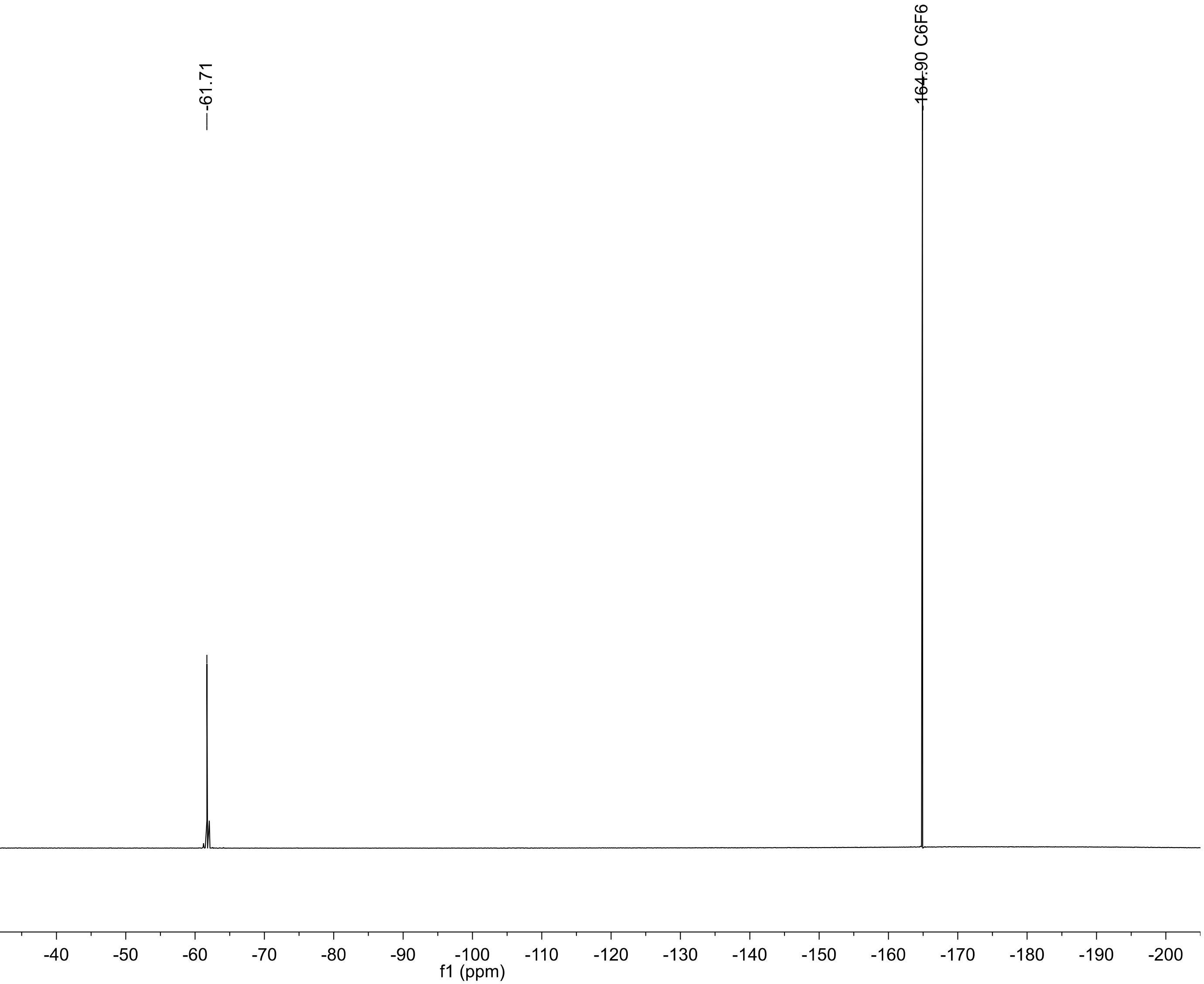

^{19}F-NMR (376 MHz $(CD_3)_2SO$). **5-nitro-2-phenyl-7-trifluoromethyl-1*H*-benzimidazole 3-oxide (4ca)**

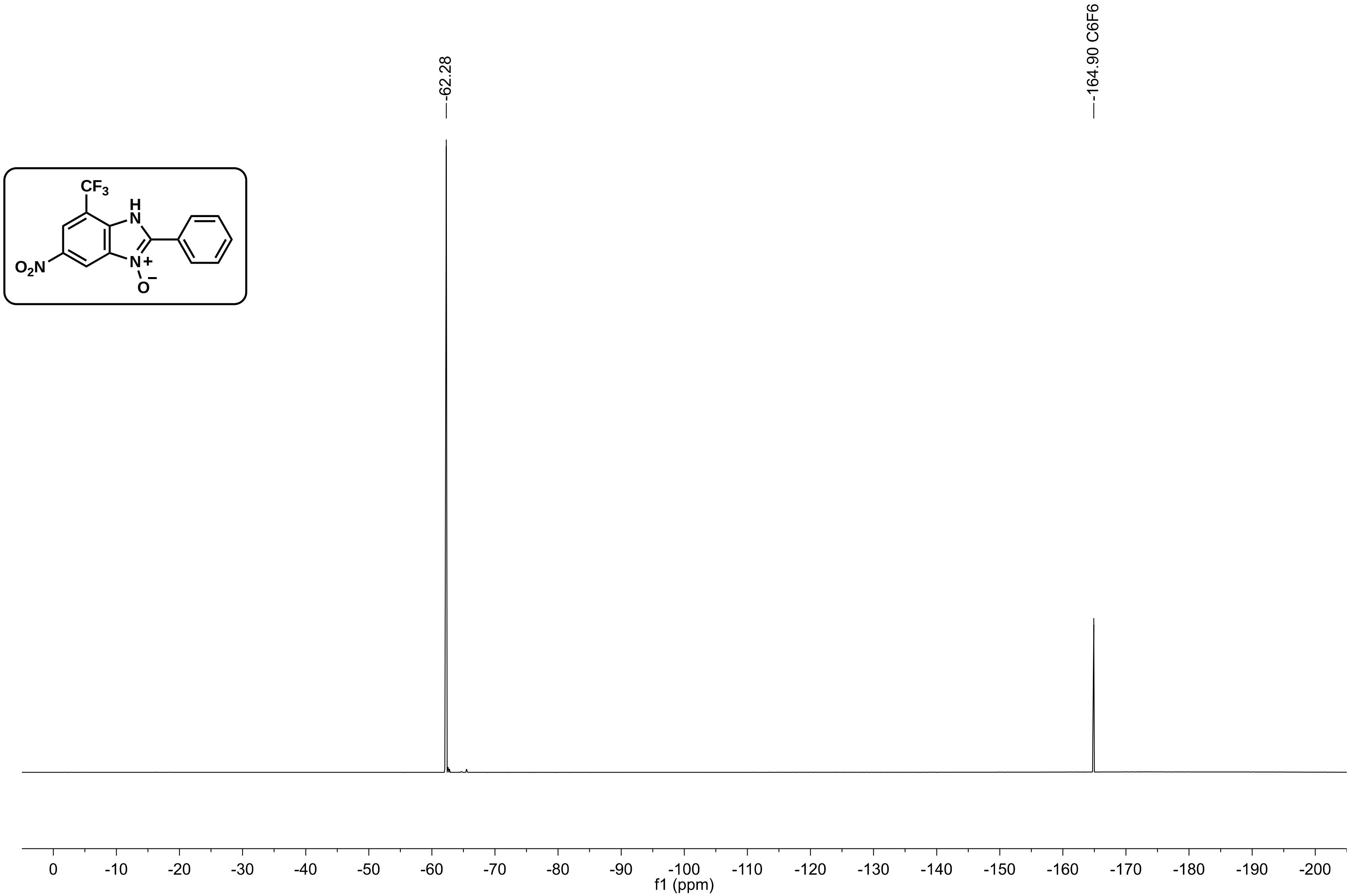

^{19}F-NMR (376 MHz $(CD_3)_2SO$). **5-nitro-2-(*o*-tolyl)-7-trifluoromethyl-1*H*-benzimidazole 3-oxide (4cb)**

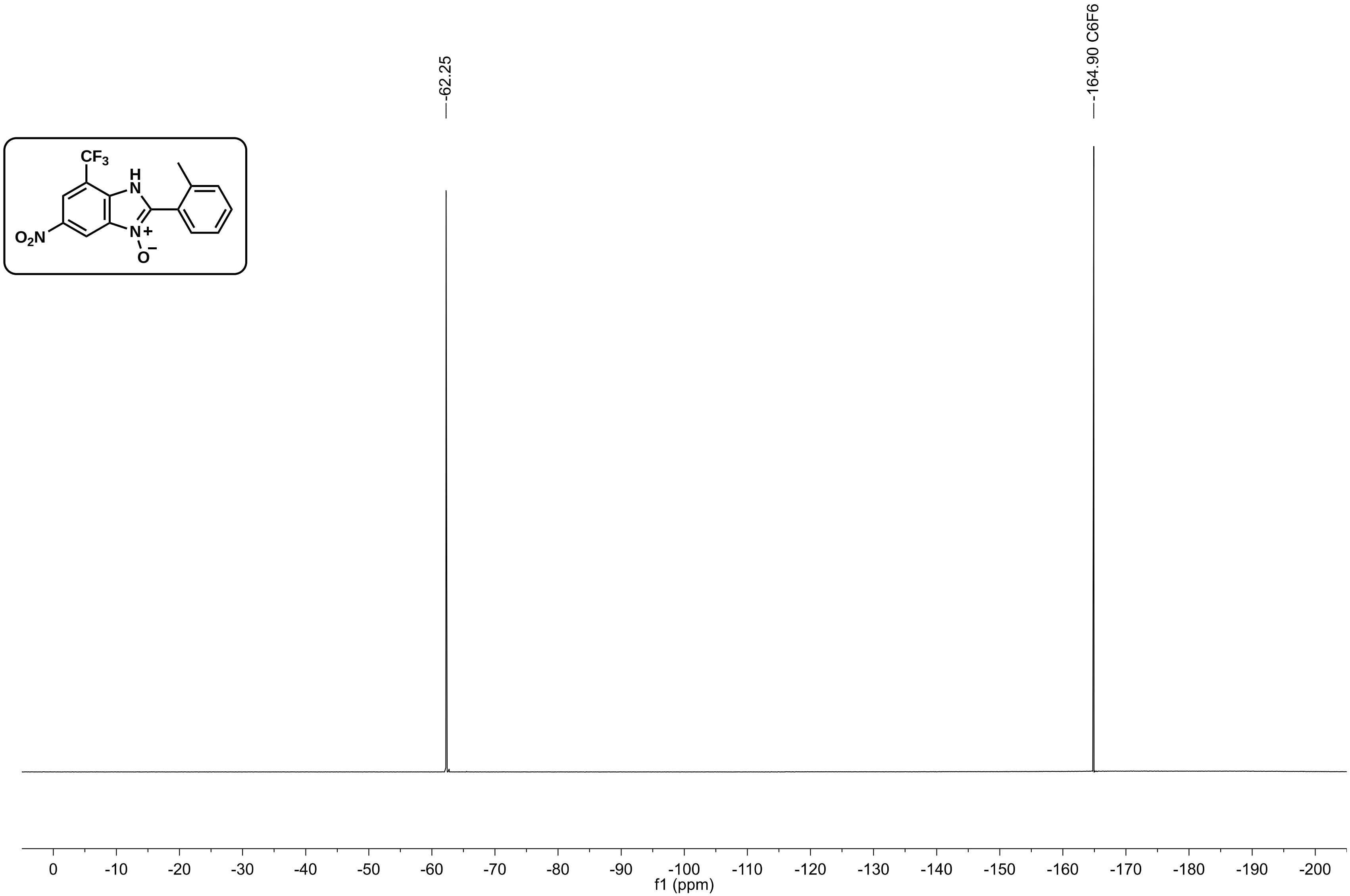

^{19}F-NMR (376 MHz $(CD_3)_2SO$). **2-(2-fluorophenyl)-5-nitro-7-trifluoromethyl-1*H*-benzimidazole 3-oxide (4cc)**

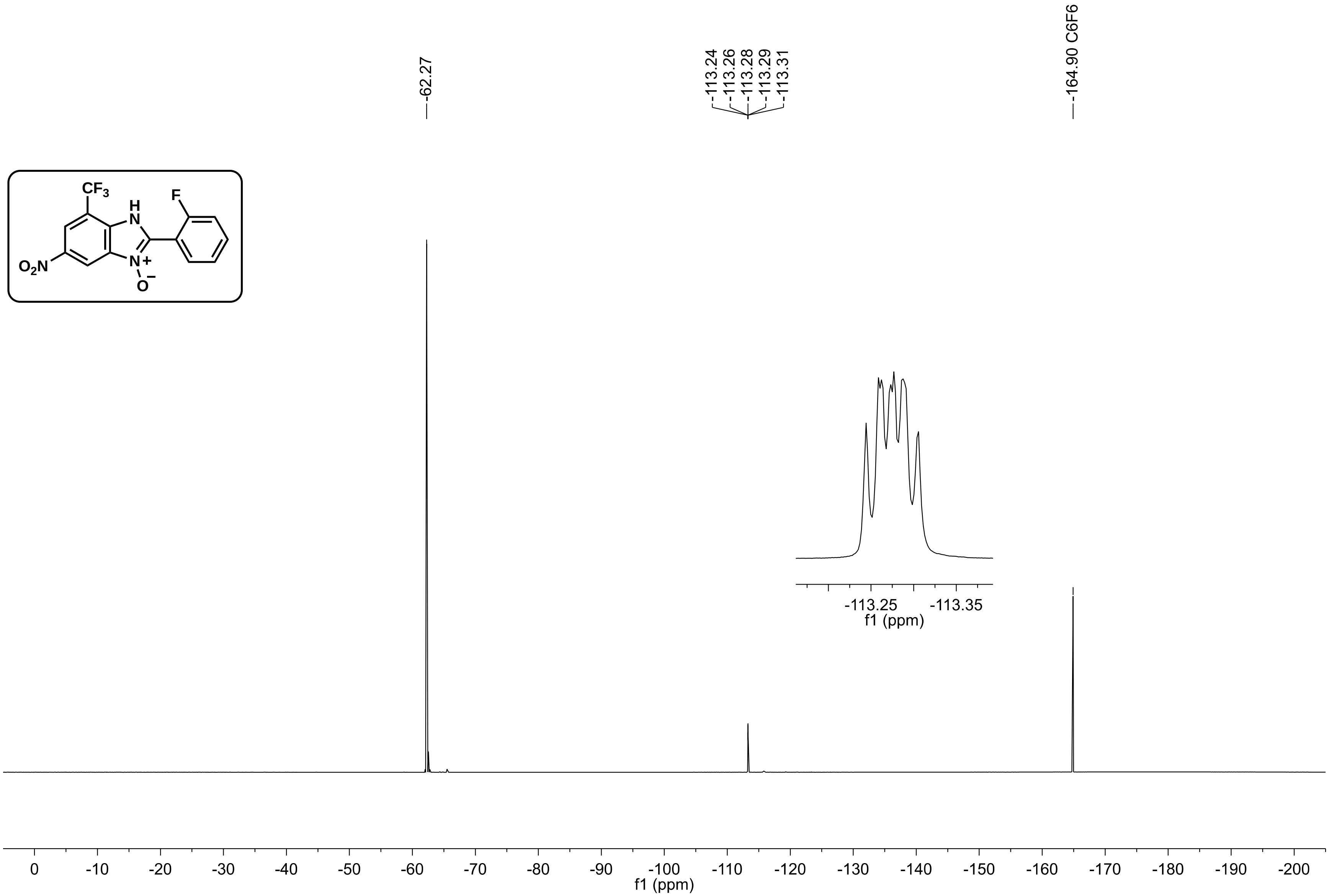

^{19}F-NMR (376 MHz $(CD_3)_2SO$). **2-(2-ethoxyphenyl)-5-nitro-7-trifluoromethyl-1*H*-benzimidazole 3-oxide (4cd)**

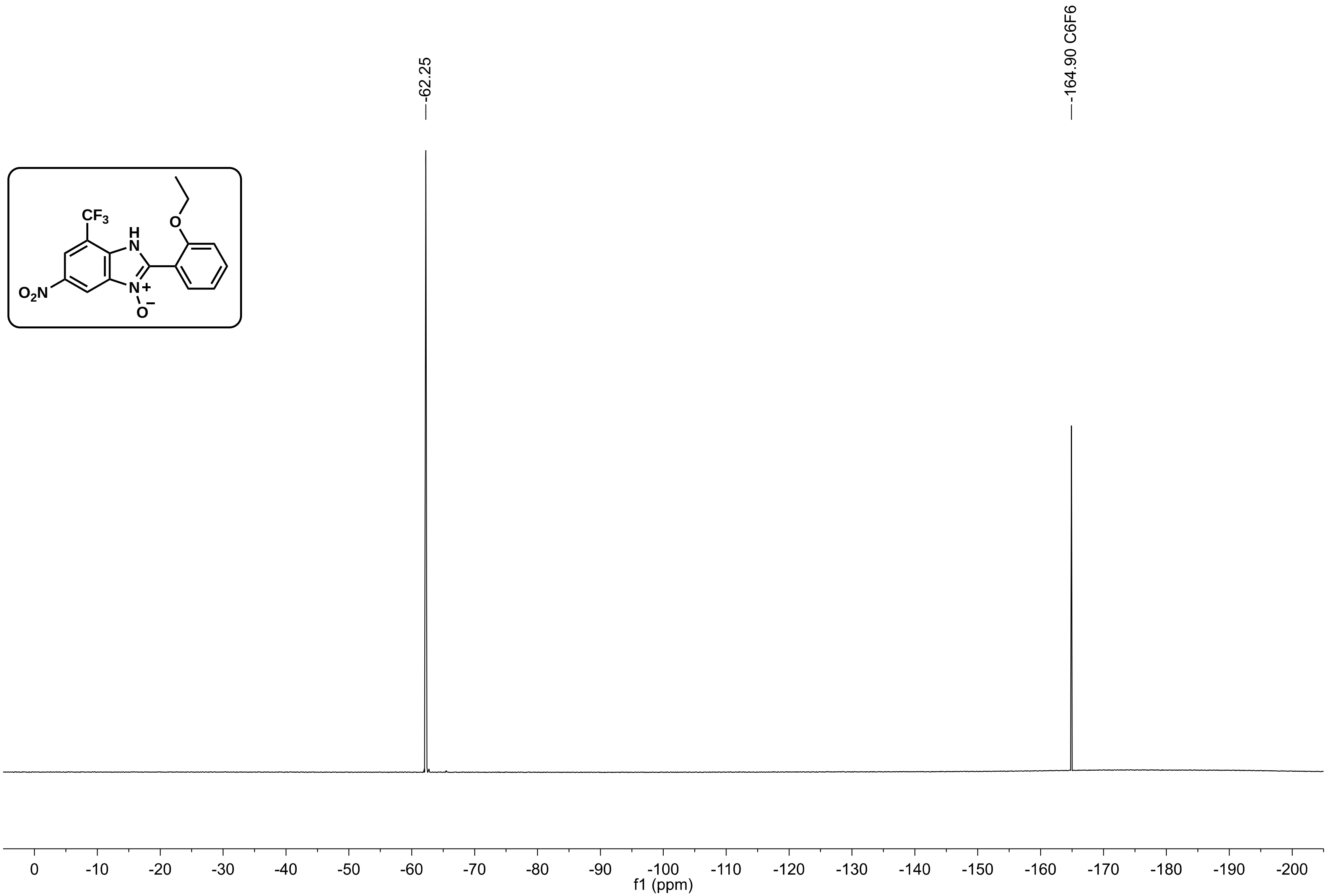

^{19}F-NMR (376 MHz $(CD_3)_2SO$). **2-(3-chlorophenyl)-5-nitro-7-trifluoromethyl-1*H*-benzimidazole 3-oxide (4ce)**

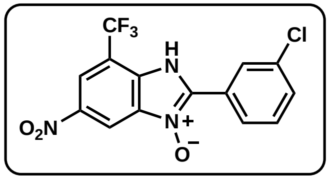

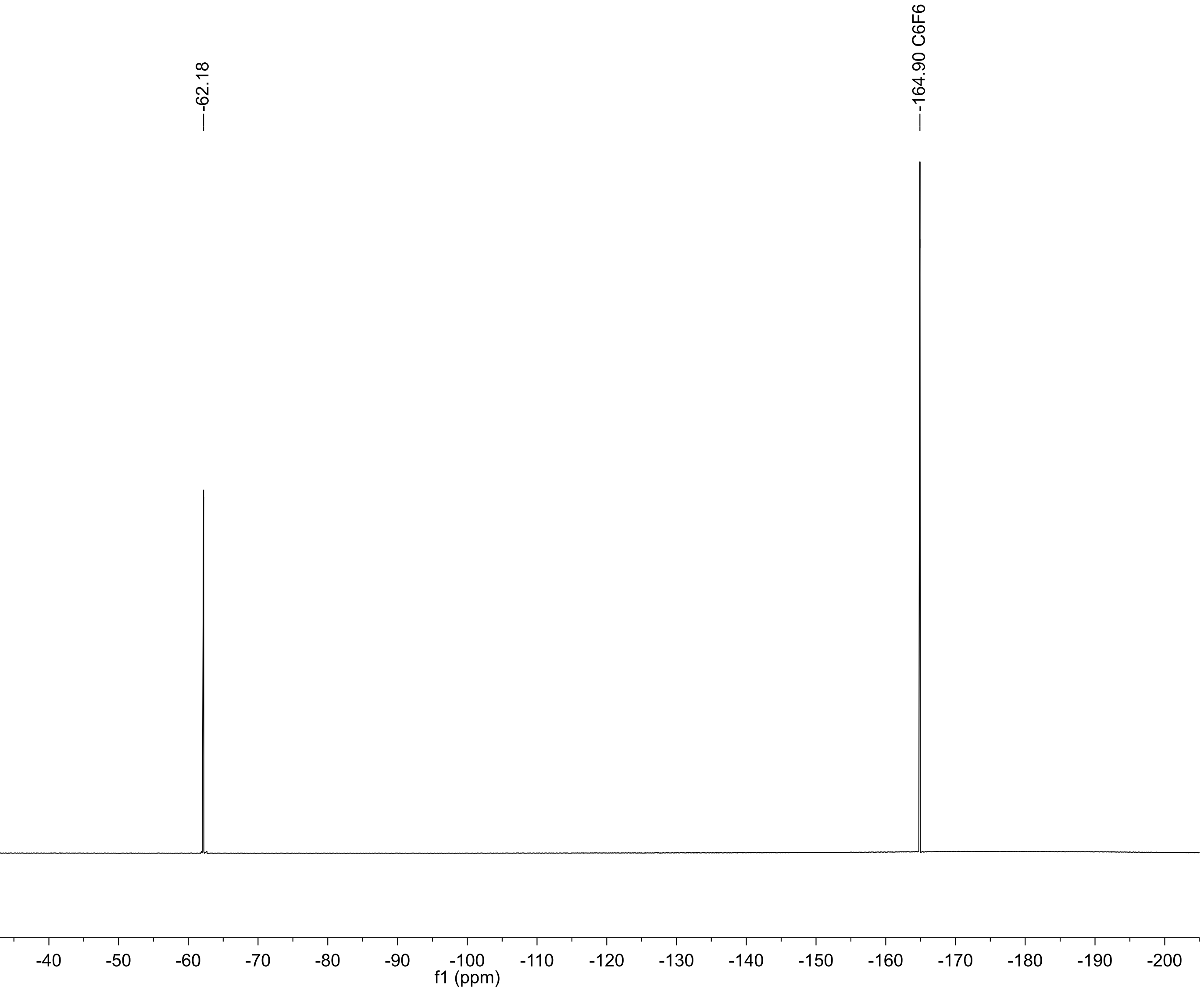

^{19}F-NMR (376 MHz $(CD_3)_2SO$). **2-(3-methoxyphenyl)-5-nitro-7-trifluoromethyl-1*H*-benzimidazole 3-oxide (4cf)**

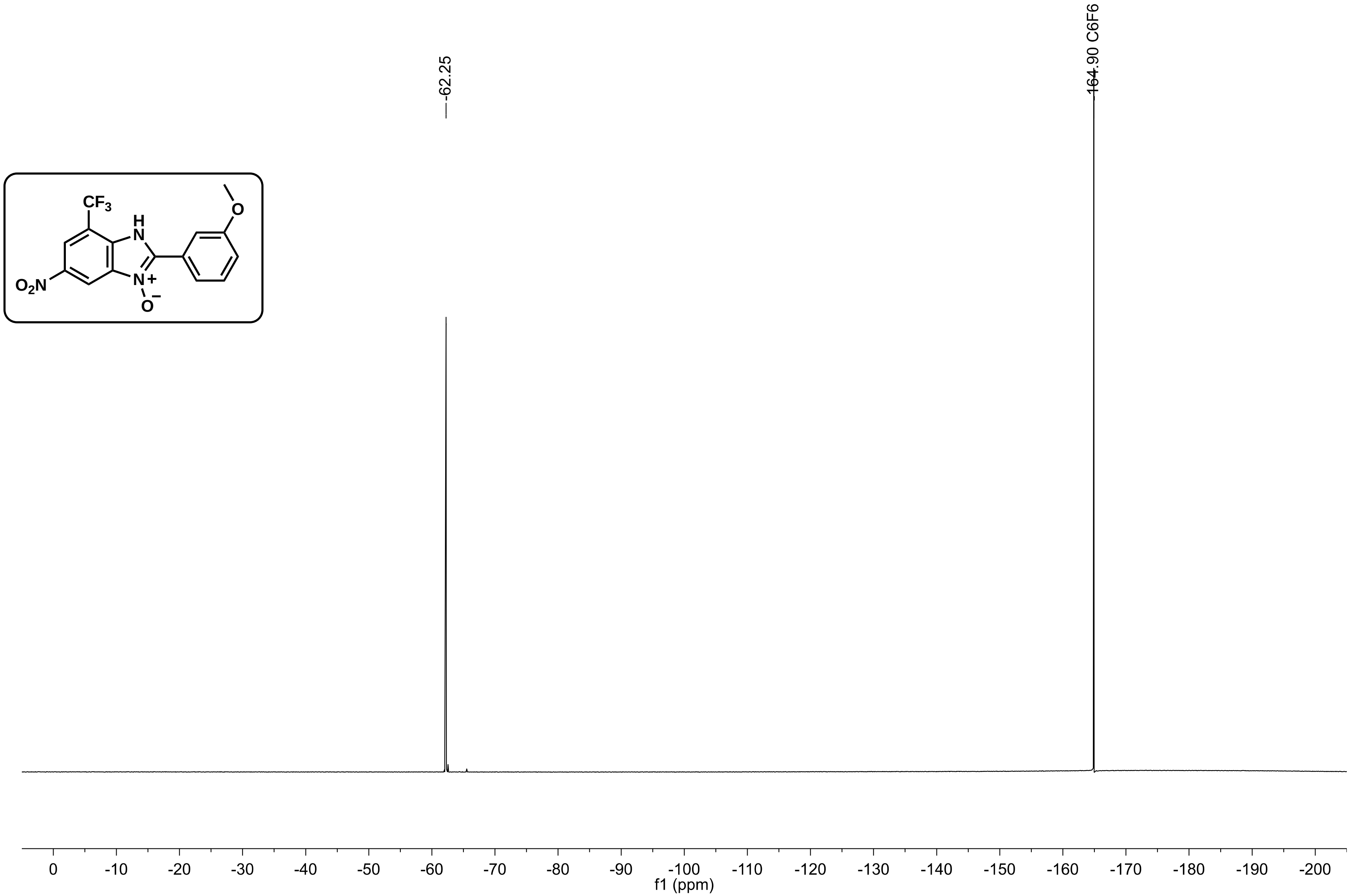

^{19}F-NMR (376 MHz $(CD_3)_2SO$). **5-nitro-2-(*p*-tolyl)-7-trifluoromethyl-1*H*-benzimidazole 3-oxide (4cg)**

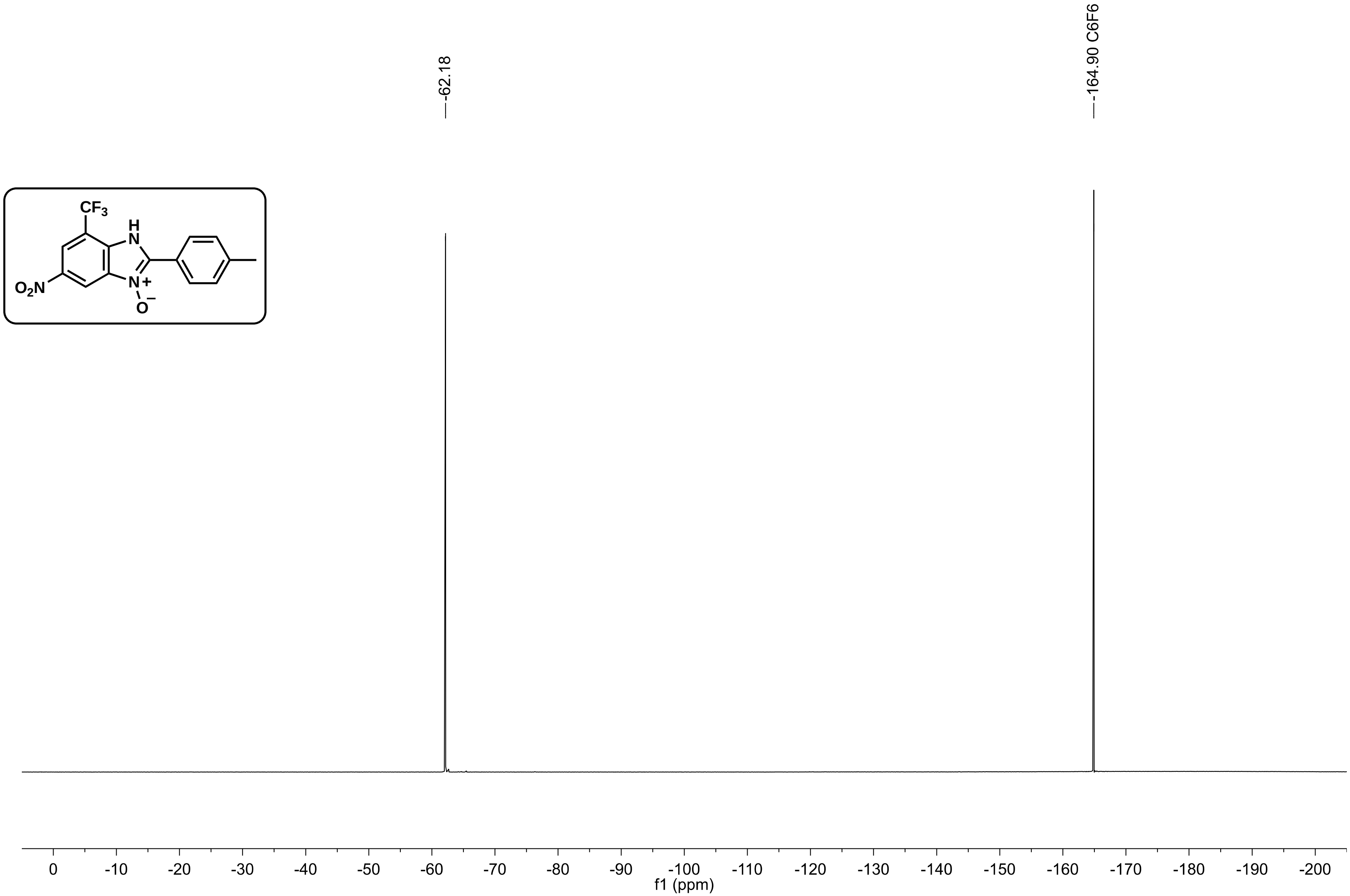

^{1F}F-NMR (376 MHz $(CD_3)_2SO$). **2-(4-fluorophenyl)-5-nitro-7-trifluoromethyl-1*H*-benzimidazole 3-oxide (4ch)**

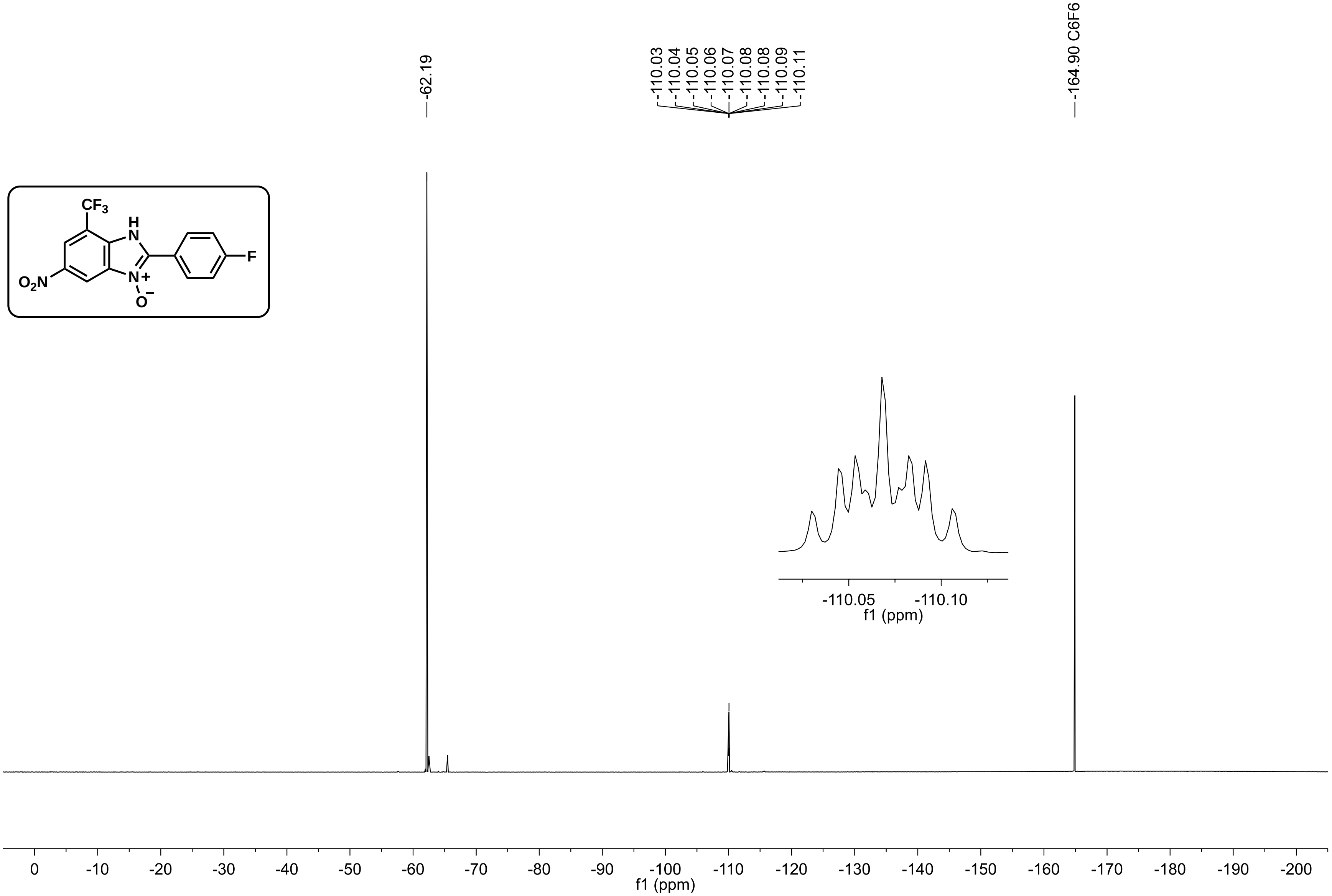

^{19}F-NMR (376 MHz $(CD_3)_2SO$). **2-(4-chlorophenyl)-5-nitro-7-trifluoromethyl-1*H*-benzimidazole 3-oxide (4ci)**

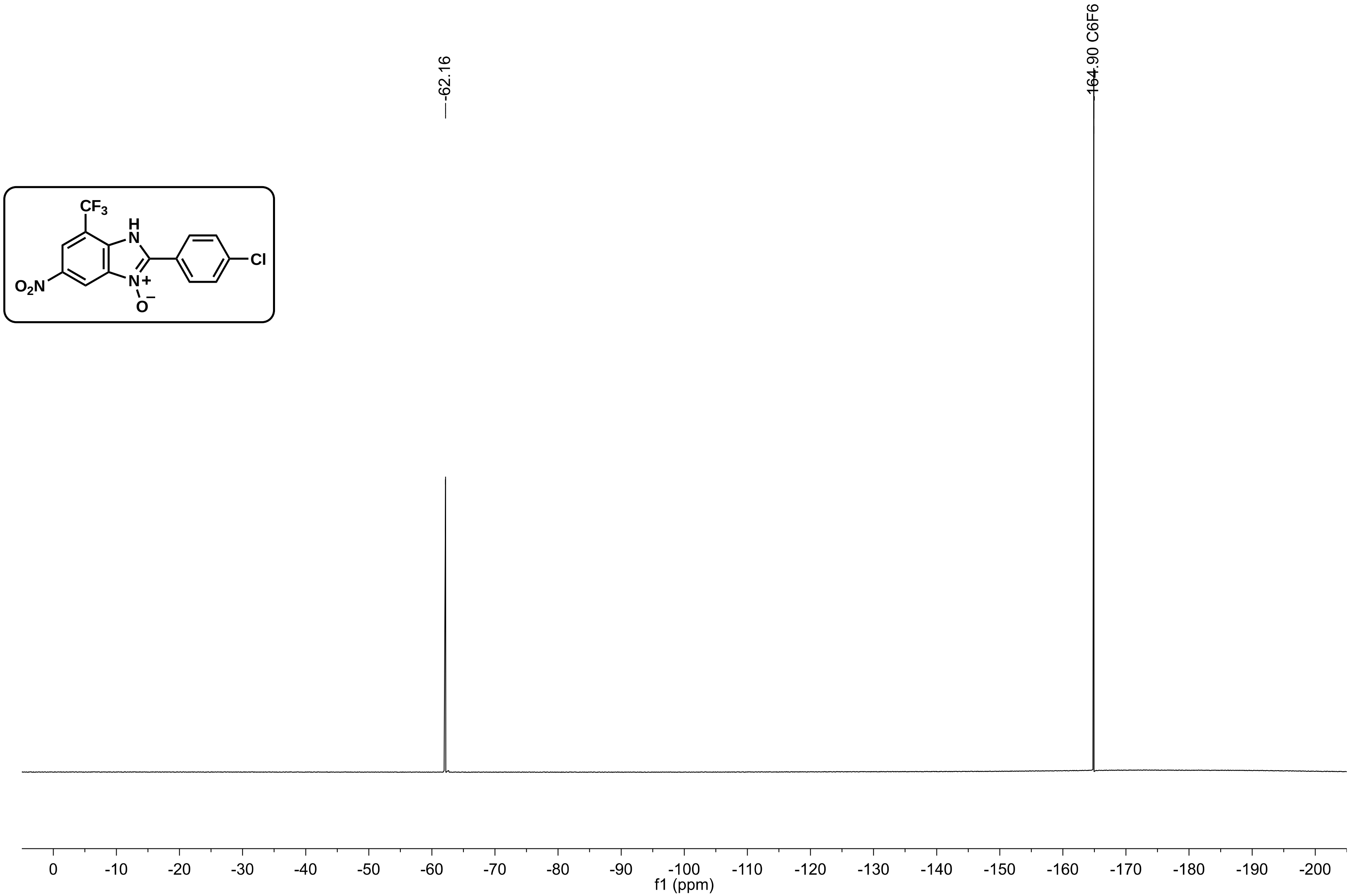

^{19}F-NMR (376 MHz $(CD_3)_2SO$). **2-(4-methoxyphenyl)-5-nitro-7-trifluoromethyl-1*H*-benzimidazole 3-oxide (4cj)**

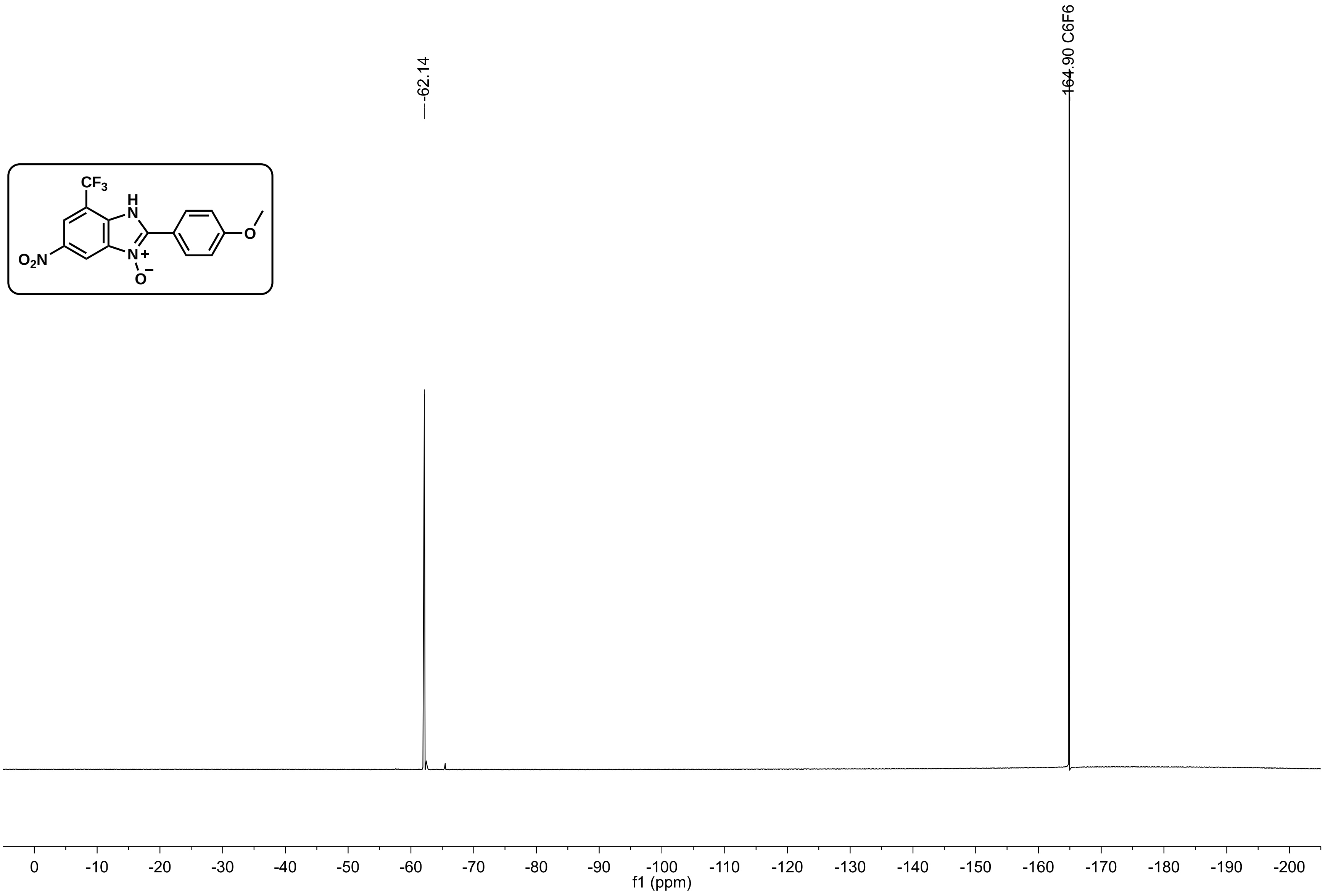

^{13}C-NMR (376 MHz $(CD_3)_2SO$). **2-(furan-2-yl)-5-nitro-7-trifluoromethyl-1*H*-benzimidazole 3-oxide (4ck)**

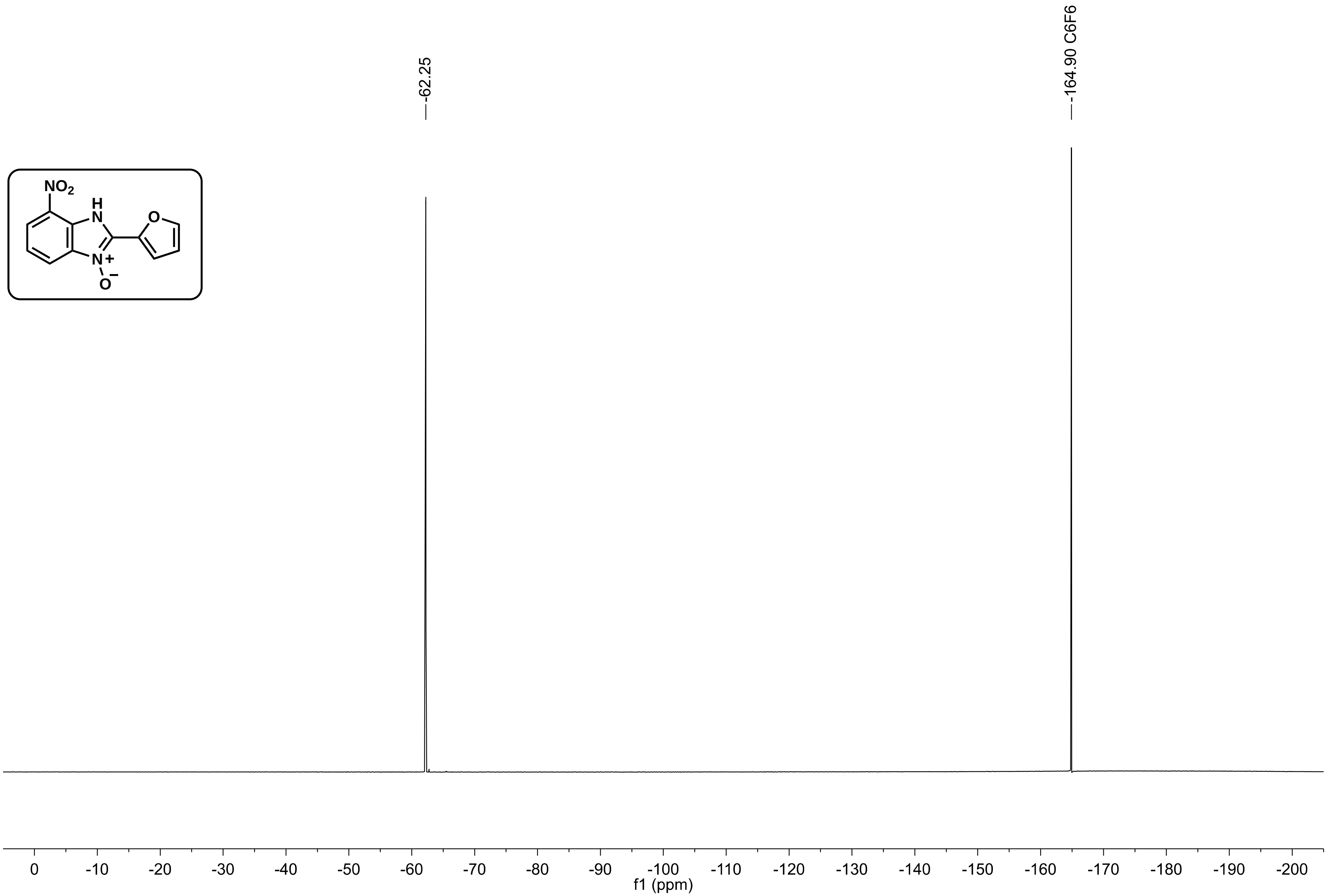

^{19}F-NMR (376 MHz $(CD_3)_2SO$). **5-nitro-2-(thiophen-2-yl)-7-trifluoromethyl-1*H*-benzimidazole 3-oxide (4cl)**

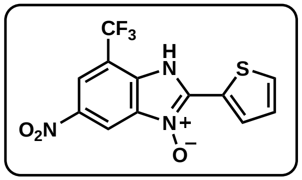

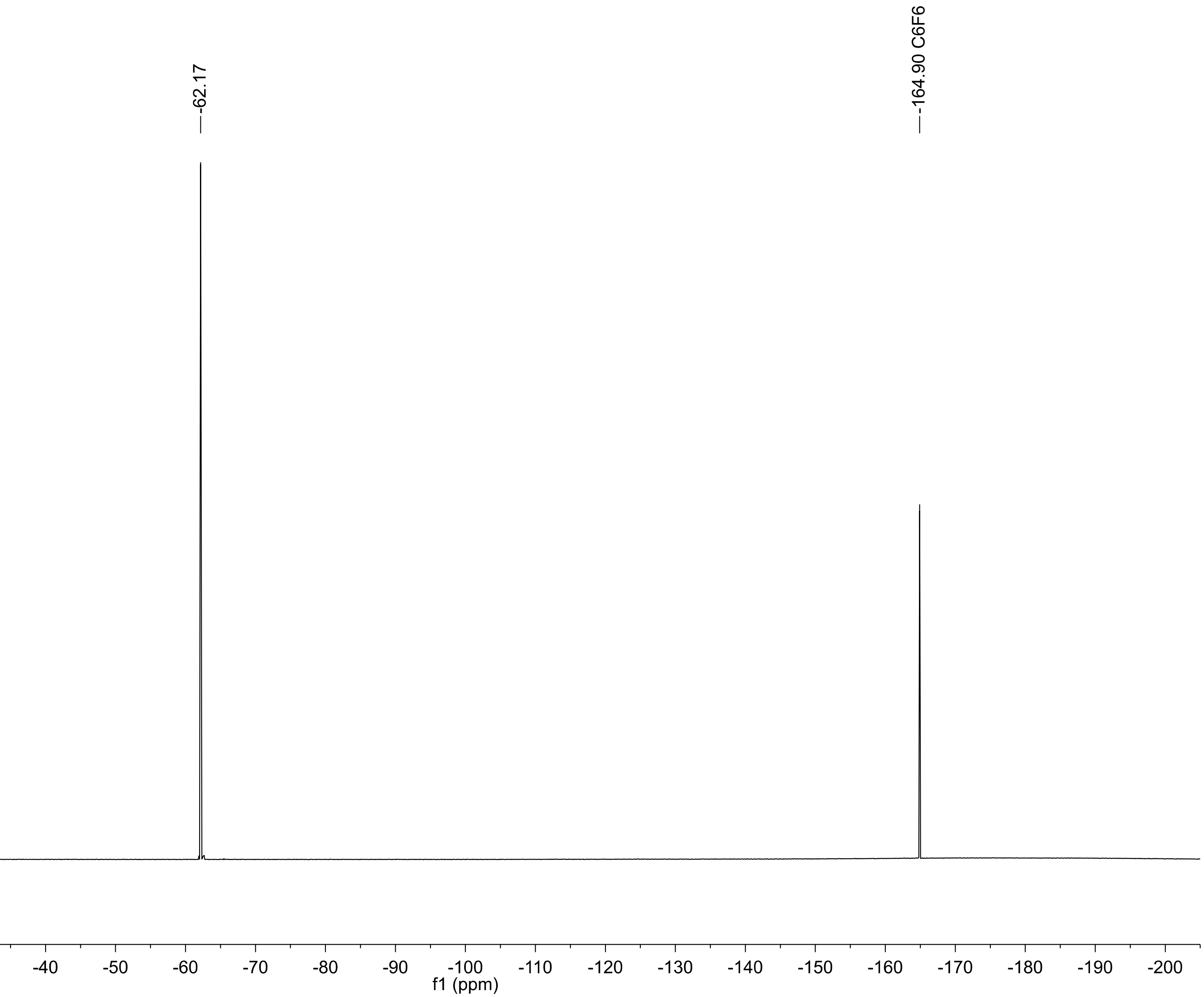

^{19}F-NMR (376 MHz $(CD_3)_2SO$). **5-nitro-2-(pyridin-3-yl)-7-trifluoromethyl-1*H*-benzimidazole 3-oxide (4cm)**

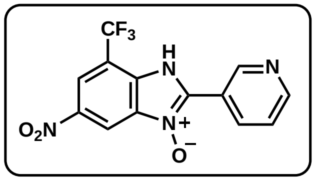

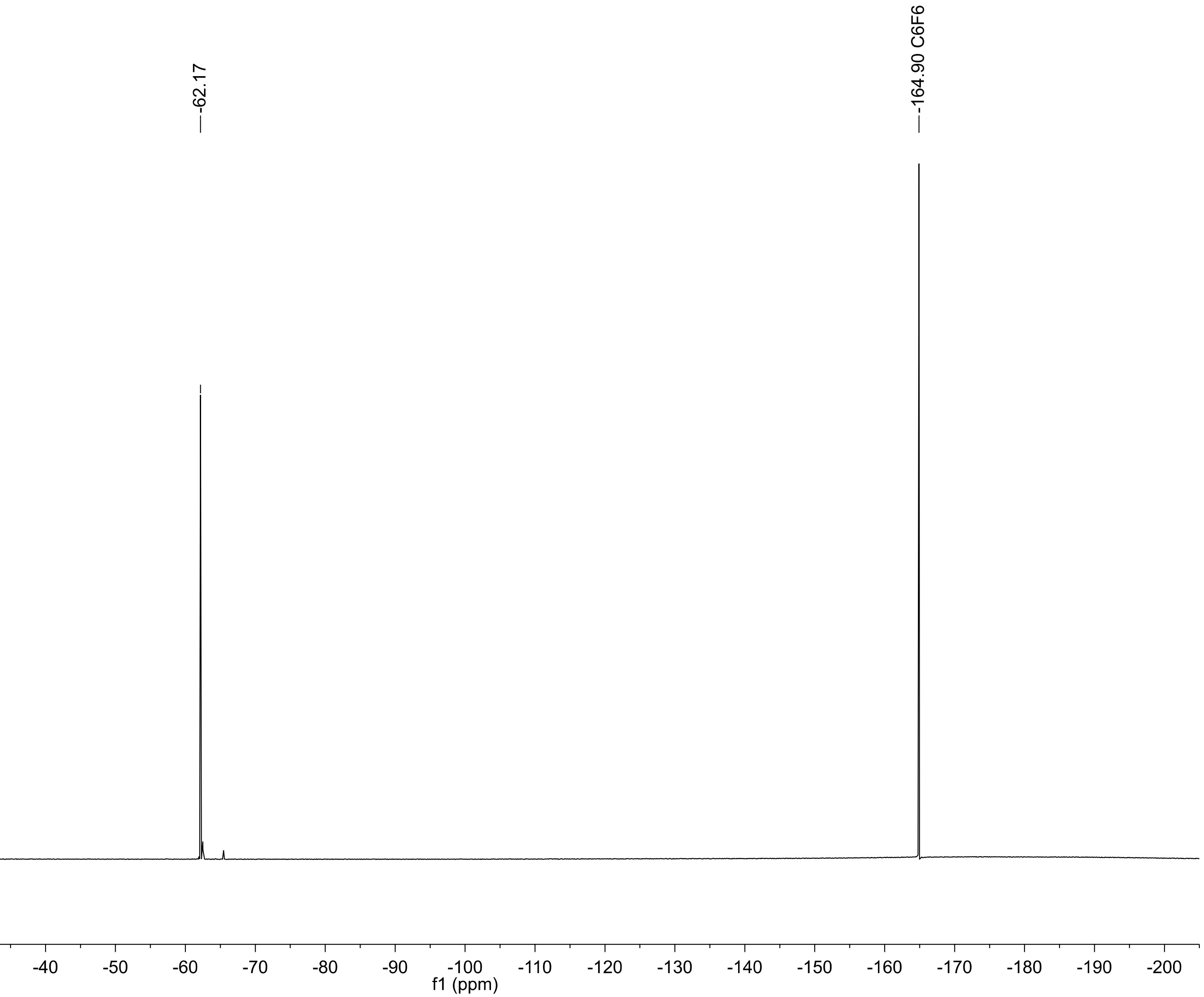

^{19}F-NMR (376 MHz $(CD_3)_2SO$). **2-benzyl-5-nitro-7-trifluoromethyl-1*H*-benzimidazole 3-oxide (4cn)**

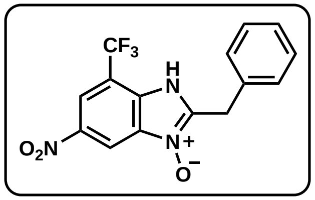

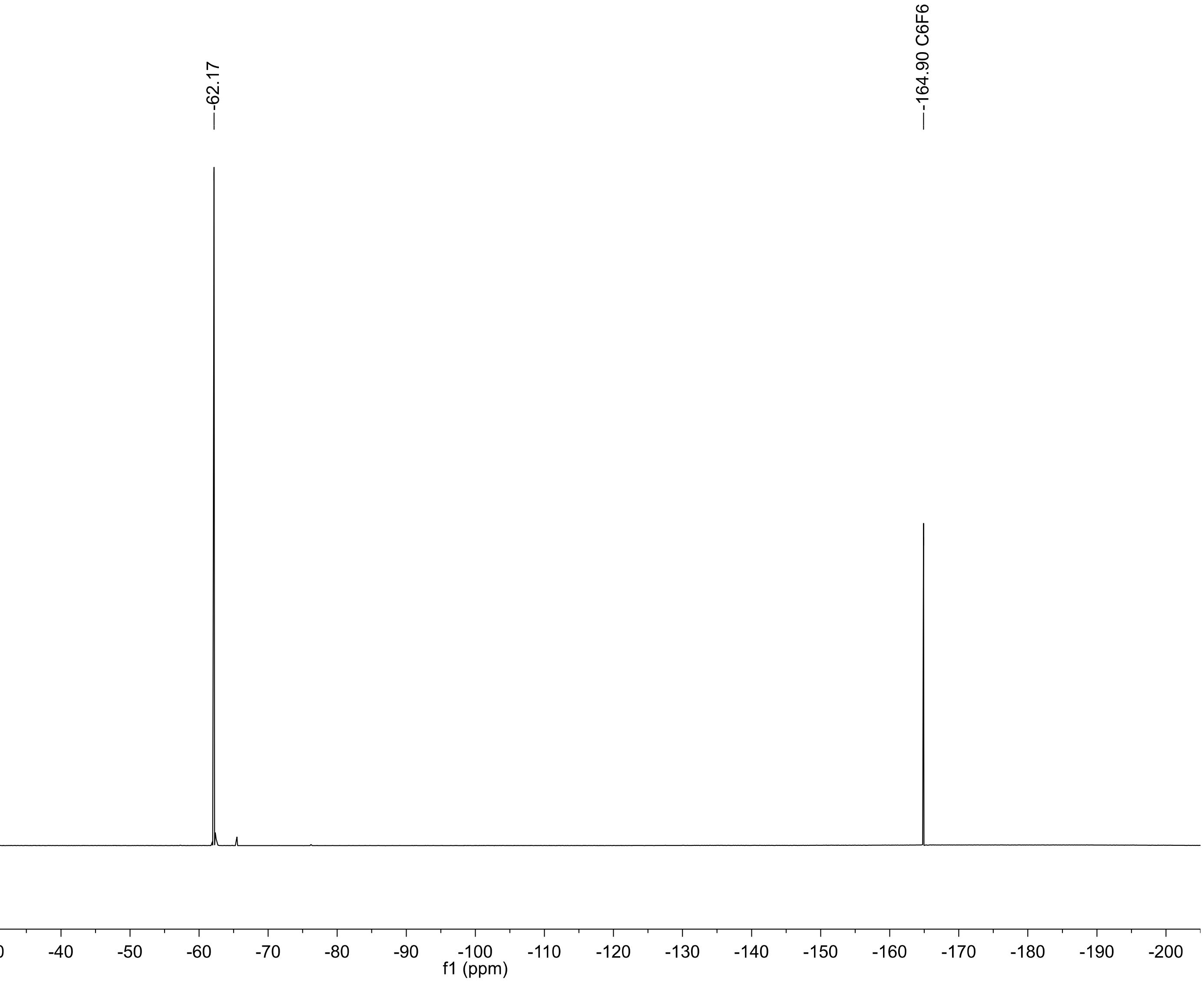

Supplement: Supplementary file 1 [file molecules-24-03639-s001.pdf]
